# Minimum service standards assessment tool and the hospital strengthening program: a novel first step towards the quality improvement of Nepal’s national hospital system

**DOI:** 10.1016/j.lansea.2025.100548

**Published:** 2025-02-22

**Authors:** Rita Pokhrel, Abigail Knoble, Pratibha Gautam, Mohammad Kashim Shah, Pravin Paudel, Archana Amatya, Madan Kumar Upadhyaya, Ruma Rajbhandari

**Affiliations:** a Nick Simons Institute, Kathmandu, Nepal; b Mass General Brigham, Boston, MA, USA; c Harvard School of Public Health, Boston, MA, USA; d Quality Standard and Regulation Division, Ministry of Health and Population, Government of Nepal, Kathmandu, Nepal; e Harvard Medical School, Boston, MA, USA

**Keywords:** Health system strengthening, Healthcare quality, Quality health systems, Nepal, Healthcare policy, Healthcare system assessment, Hospital strengthening, Curative services, Quality assessment tools, Health system quality monitoring

## Abstract

District hospitals in Nepal, as in other Low- and Lower - Middle Income Countries (LLMICs), struggle to provide quality care due to inadequate investments in equipment, human resources, and hospital infrastructure. To address these challenges, under the leadership of the Ministry of Health and Population (MoHP), Nick Simons Institute (NSI) developed and implemented the novel Minimum Service Standards (MSS) assessment tool in close partnership with the Government of Nepal. The MSS tool routinely assesses a hospital’s readiness to provide mandated care and identify gaps, which are then closed via a small annual grant to the health facility, together providing the knowledge and resources to improve hospital readiness and service availability. Since its inception in 2014, the program has expanded to 130 government hospitals as of April 2024. The program provides a blueprint for hospitals to pursue excellence and has tracked and motivated substantial improvements in services since 2014, such as basic laboratory investigations (+46%), cesarean sections (+40%), and spinal anesthesia (+32%). The program has impacted healthcare policy due to the close collaboration with the MoHP, influencing budget allocation, insurance payments, and hospital upgrade criteria, cementing its sustainability and long term impact.

**Funding:**

No external funding

## Introduction

Poor-quality healthcare, rather than access, is now the bigger barrier to reducing mortality and continues to be a persistent challenge in low- and middle-income countries (LMICs), leading to low patient satisfaction and poor health outcomes.^1^ The majority of services in rural communities of LMICs are delivered by government district hospitals. In Nepal, these rural district hospitals are relied on by nearly 80% of the 30 million population.^2^ Nepal’s mountainous terrain and difficult geography often make district hospitals the only option in an emergency and referrals to higher centers of care are difficult if not impossible.^3^ Despite their importance, the quality of care in these hospitals is suboptimal due to inadequate investments in critical areas such as medical equipment, human resources, and hospital infrastructure.^4^^,^^5^

In 2015, only 11% of assessed health facilities in Nepal met the World Health Organization’s quality of care standards, and only 13% had all seven basic equipment items, including an adult weighing scale, a thermometer, a stethoscope, and a light source for service provision, indicating a widespread problem of poor quality of care across the country.^6^ This was particularly acute in district hospitals, where only 20% could perform a Cesarean section when NSI, a non-governmental organization, began its work in 2006.^7^

While seeking a comprehensive tool to set a standard of quality at hospitals, we identified a significant gap: tools that routinely and reliably assess hospital management and overall curative service readiness are largely absent in LMIC settings. Some existing tools, such as the World Health Organization’s 1000 pediatric and neonatal quality indicators or Surgical Quality Indicators, are tailored for specific diseases, services, or populations and vertical programs, but they fail to provide the broader, hospital-wide assessment required.^8^, ^9^, ^10^, ^11^, ^12^, ^13^ Other hospital quality assessment tools, such as the Safety Assessment Framework, lack the detail needed to provide a clear path forward for hospital quality improvement with less than 60 indicators.^14^^,^^15^ Finally, while the Demographic and Health Survey (DHS) is invaluable for international comparisons and goal-setting, it is conducted infrequently and evaluates a subset of health facilities.^6^^,^^16^^,^^17^ As a result, hospitals lack frequent, standardized evaluations to track performance, identify areas for improvement, and establish a consistent standard of quality for hospital management.

This paper describes the development, implementation, and status of the novel Minimum Service Standards (MSS) assessment tool and the Hospital Strengthening Program (HSP), a collaborative initiative between the NSI and the MoHP to improve the quality of healthcare and curative services at district hospitals across Nepal.

## What we did

To address the poor quality of hospital care, NSI developed and launched the MSS/HSP program under the leadership of and in close partnership with the MoHP in 2014. The MSS/HSP program identifies gaps in hospital readiness and service availability through a novel comprehensive checklist tool paired with a funding mechanism to close gaps identified by the tool.^16^^,^^18^ MSS was strategically designed to assess governance, human resources, essential equipment, and basic infrastructure that are crucial for delivering quality curative care services at the hospital level in the Nepali context.^19^

The MSS assessment tool was developed by a Technical Working Group, composed of subject matter experts working through consultative workshops and meetings, utilizing key guiding documents like the National Health Policy 2014, Policy on Quality Assurance in Health Care Services, 2064 BS (2007 CE), and the Nepal Health Sector Strategy 2015–2020^19^ (Nepal follows the Vikram Samvat calendar and CE dates have been added for context). For more information regarding the development and implementation process, including engaging stakeholders and the endorsement process, field testing, and closed group workshops, see pages 1–6 of the Introduction from any of the MSS Tools included in the Supplementary files. Further, the Consolidated Framework for Implementation Research (CFIR) for the MSS/HSP program is in the Supplementary files. This produced the first version of the tool as an extensive checklist with 350 indicators and 15 annexes that allow for an itemized list of supplies, equipment, staff, and other items for district hospitals comprising three primary sections: 1) Governance and Management, 2) Clinical Services, and 3) Hospital Support Services weighted at 20%, 60%, and 20%, respectively.

Governance and Management subsections include Organizational Management, Medical Records and Information Management, and Human Resources Management and Development, detailing the different aspects of hospital governance. Example indicators include “Annual plan & budget is approved by the Hospital Management Committee before the fiscal year starts” (Indicator 1.1.6), “Hospital implements health insurance program” (Indicator 1.3.8.3), and “Dedicated Accounts Department of hospital with space and furniture” (Indicator 1.4.1.1).^19^

Clinical Services span the human resources, medical equipment, and hospital infrastructure needed to make services possible and is subdivided by department. Subsection examples include OPD Service, Delivery Service, Diagnostics and Laboratory Services, and Medico Legal Services. Additionally, best practices such as infection prevention, communication, or waste management are further detailed by department. Example indicators include “For 5 ER beds the ratio of Doctor on duty: Nurse: Paramedics: Office Assistant should be 1:1:1:1” (Indicator 2.3.2.1), “Hand washing facility with running water and soap is available for practitioners.” (Indicator 2.2.1.8.3), “Routine major surgeries available on scheduled days” (Indicator 2.8.1.1.2), “Separate space dedicated for pre-labor, labor and postnatal patients” (Indicator 2.7.2.1.4), and “The pharmacy is open 24 × 7” (Indicator 2.5.4).^19^

Lastly, Hospital Support Services evaluate the availability and functionality of the services that allow a hospital to function such as housekeeping and cleanliness, laundry, safety and security, and water supply. Example indicators include “There are separate rooms designated for dirty utility, cleaning, washing and drying and sterile area for sterilizing, packaging and storage” (Indicator 3.1.1.1), “Hospital has alternate power generator capable of running X-ray and other hospital equipment” (Indicator 3.4.3.2), and “Disaster preparedness orientation has been given to all staff at least every six months” (Indicator 3.7.6.4).^19^

Together, these three sections provide a comprehensive portrait of the human resources, equipment, practices, and infrastructure required for a high-functioning hospital to deliver quality health services. The subdivisions allow for areas of weakness and strengths to be easily analyzed and targeted for interventions. Indicators allow for specific, measurable, attainable, realistic, and time-bound goals to be set at the hospital, district, provincial, and federal level (See recent examples in Table 1). This is even explicitly encouraged as an indicator within the MSS assessment tool, “The hospital has developed specific plans to improve quality based on the MSS assessment” (1.6.5).^19^

**Table 1:** Excerpts from primary hospital 2024 action plans.

Prioritized gap	Action steps to be taken to fulfill gap	Responsible	Time frame	Managerial/Budget support
Hospital has trained security personnel round the clock. (3.7.1.1)	Proposed HDC to lobby with the Local Government for human resources.	HDC Chairperson	2–3 weeks	HDC, Local Government
Separate space allocated for breastfeeding for staffs/Separate space in duty room designated for breastfeeding (1.2.7.5)	Separate room allocation	Hospital Management Officer, Head of Inpatient	1–2 weeks	Hospital Chief, HDC
All staffs of hospital use electronic attendance (1.2.5)	Planning coordination and acquiring compatible software finalization	Hospital Chief, HDC, Accountant	4–6 months	HDC, Provincial Government
Hospital implements Robson’s classification (hospitals with CEONC services) (1.6.8.4)	Plan formulation and budgetary support	Provincial Health Ministry	As soon as possible	Provincial Health Ministry
Disaster area identified with adequate furniture to carry out Triage in case of disaster (2.3.10.2)	Plan formation	Head of Disaster Management	2 months	Administration, HDC

Small edits have been made for clarity and standard references have been added.

HDC = Hospital Development Committee.

After an initial pilot at four hospitals in 2013, NSI, in partnership with the MoHP, implemented a three-part workshop series to introduce the original MSS Assessment Tool across all 75 district hospitals from 2014 to 2017.^16^^,^^18^ The hospitals were grouped into 14 clusters based on geographic location, with each cluster attending three, two-to three-day workshops at a host hospital at roughly three month intervals. These workshops, attended by 10–15 participants per hospital and regional government officials, trained hospital staff to conduct MSS self-assessments to identify gaps in hospital readiness for quality curative care and to develop actionable plans to address these gaps, similar to those in Table 1.^19^

**Fig. 1:**
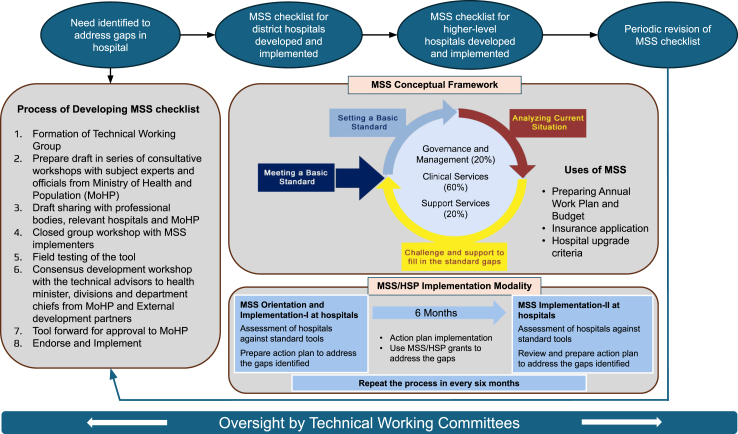
Conceptual framework for MSS/HSP.

Recognizing that identifying gaps was only a first step, NSI paired the MSS assessment tool with the HSP, an annual grant of 500,000 NPR (4870 USD) provided directly to the district hospital as a mechanism to follow up on the action plan.^20^ Hospital self-assessments were subsequently complemented by external follow-up assessments by NSI and the MoHP at a subset of hospitals three- and six-months after the last workshop to ensure a comprehensive evaluation process. Further, formal and informal qualitative interviews regarding MSS/HSP implementation over the past decade have informed MSS development and national scale up. Fig. 1 illustrates the conceptual framework for MSS/HSP.

Although initially developed for first referral district hospitals in 2013, the MSS Assessment Tool has been differentiated for Health Posts and Primary, Secondary A, Secondary B, and Tertiary Hospitals.^19^ Primary hospitals are 25–50 bed facilities, designated as first referral centers, and are expected to offer general medicine, general surgery, obstetric, and pediatric care and are currently assessed against 647 indicators. As hospital level increases, the mandated services and bed capacity grows. For example, Secondary A hospitals are assessed against 721 standards and are expected to additionally provide dental and orthopedic care, which are detailed in the corresponding MSS tool. Gaps between mandated services and the ground reality can be readily identified through the differentiated MSS tools, enabling targeted improvements to ensure hospitals can meet their expected standards of care.

These tools, along with relevant indicators and appendixes, are published by the Department of Health Services and can be found in the Supplementary files. Aggregate MSS data and MSS resources, including handbooks and implementation guidelines can be accessed at https://msshealth.org.np.

The paper utilizes existing hospital data and internal implementation assessments to analyze trends in hospital services and its impact on policy and management within the healthcare sector as a retrospective observational study. No human subjects were involved in data collection or analysis. Ethical approval was not required as the study utilized existing data for quality improvement purposes.

## Program impact

Before the MSS/HSP program, many hospitals, along with their Hospital Management Committee members, were unaware of the detailed standards a functional hospital should meet. The MSS assessment tool has closed this knowledge gap by providing an objective and measurable blueprint for success. Then, through the paired HSP financial support, the gaps identified can be addressed. As the Hospital Development Committee Chairperson of Beni Hospital said, “*[the] MSS program shows the way forward*”. Since the implementation of the MSS/HSP program, there has been a substantial improvement across various MSS hospital indicators nationally.

The average self-assessed MSS score across 45 district hospitals at the time of the first workshop in 2014–2015 was 46.9%. By the end of the three-part workshop, the average self-assessed MSS score had increased to 70.5% (n = 36). However, external evaluations by NSI and MoHP at a subset of hospitals likely revealed a more accurate increase to 55.8% (n = 18) at a three-month follow-up - still a substantial and sustained improvement. As of April 2024, the average scores for these 45 hospitals increased to 77.4%, reflecting a 30.5% overall increase. However, it should be noted that the novel MSS tool has been modified and underwent significant changes in 2018/19.^19^ Additionally, 38% of the 45 hospitals (n = 17) have been upgraded from a Primary hospital to a Secondary A hospital and are now evaluated using the corresponding Secondary A Hospital MSS tool. This tool includes more departments and corresponding indicators, potentially underestimating the true growth.

To account for these significant changes to the novel MSS Assessment tool in 2018/19 and hospital upgrades, specific baseline indicators measuring key services were paired with their current corresponding indicator across all MSS tools at each hospital level to accurately analyze the change in specific services over time.

**Fig. 2:**
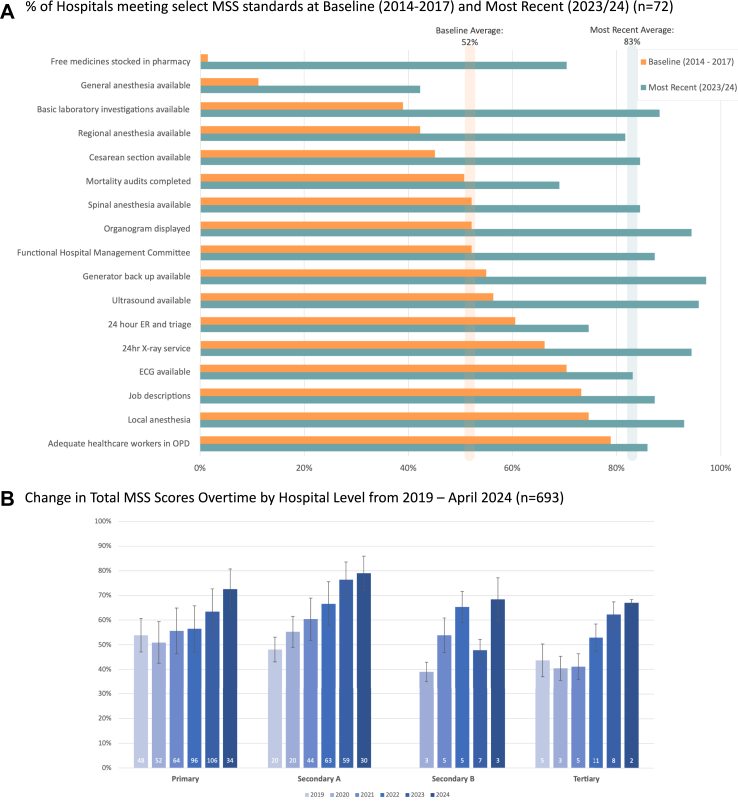
Change in MSS scores over time. A. % of Hospitals meeting select MSS standards at baseline (2014–2017) and Most Recent (2023/24) (n = 72). B. Change in total MSS scores overtime by hospital level from 2019–April 2024 (n = 693).

Fig. 2A compares the percent of hospitals meeting paired MSS standards at baseline during the pilot (2014–2017) and at their most recent MSS assessment (2023/24). Paired t-tests were used to determine significance. The percent of hospitals meeting these select standards increased from 51.8% to 83.1%, reflecting a 31.3% increase (p < 0.001). 15 of the 17 indicators showed a statistically significant increase (p < 0.05), with changes ranging from +7% to +69%. Generator back-up (97%), USG availability (96%), and 24 h X-ray service (94%) are now nearly universal. Cesarean section service access increased dramatically from 45% at baseline to 85%, shedding light on the range of services now available given the equipment, staff, and anesthesia needed to perform the procedure (p < 0.001).

Beyond the initial district hospitals, the novel MSS/HSP program has been expanded and is currently being implemented at 130 government hospitals nationally under the direction of the MoHP.^16^ The change in total MSS scores over time by hospital level in Fig. 2B was analyzed using t-tests, showing a significant and sustained increase in the average total MSS score from 51.6% in 2019 to 66.4% in 2023 (n = 180) and 75.0% as of April 2024 (n = 69) across all hospital levels under the current version of the novel MSS Assessment tools (p < 0.001; p < 0.001). The decrease in Secondary B’s average score during 2023 was due to the upgrade of two hospitals from Secondary A to Secondary B that year, thus being evaluated at a higher level for the first time.^18^

Because the MSS/HSP program was initiated under the Government of Nepal’s leadership, the MoHP successfully incorporated the MSS/HSP program into their Quality Standard and Regulation Division in 2019/20 and further digitized the MSS evaluation process in 2020/21. In 2015/16, the Management Division of the MoHP also began providing the Hospital Strengthening Grant, similar to the piloted HSP grant. Further, rather than being static, the novel MSS assessment tool has laid the foundation for quality improvement and continues to go through iterative development sessions led by the MoHP.^16^ The original MSS tool has been expanded to include specialized evaluation tools for health posts, primary hospitals, secondary hospitals, and specialized tertiary hospitals, such as Infectious Disease and Pediatrics Hospitals.^18^^,^^20^ This reflects the substantial investment that the MoHP continues to pour into this program, validating its position in the national infrastructure and impact. Further, it exemplifies both the utility and versatility of the MSS tool for use in diverse LMIC settings for different levels of care and its flexibility to meet needs in a changing context.

After COVID-19, MSS was utilized in a retrospective assessment, published in the Department of Health Services Annual Report 2077/78 BS (2020/21 CE), highlighted staffing shortages as the most critical resource gap during the pandemic.^16^ The lack of adequate manpower at the onset of COVID-19 left hospitals without a sufficient workforce to handle surges.^16^ These findings underscore the importance of adopting holistic approaches and prioritizing human resources strengthening in health system preparedness. Unlike external tools, such as the DHS Service Provision Assessment Survey—which are conducted infrequently representing a subset of health facilities^6^—the MSS tool provided timely assessments typically conducted two or more times a year across government hospitals.^21^ MSS data has the potential to inform future pandemics and provide timely responsiveness on the ground.

The program has had a substantial influence on policy and management within the healthcare sector, with notable outcomes including:

i. Inclusion within MoHP structure: Initially, MSS assessment of district hospitals were incorporated into annual review meetings of the Curative Division of MoHP. Now there are nine MSS Units, seven for each province, and two at the federal level - one within the Quality Standard and Regulation Division and one within the Curative Service Division which oversees the entire MSS program.^16^^,^^18^, ^20^, ^21^, ^22^
ii. Budget Allocation: The government utilizes the MSS score to determine budget allocations to government hospitals. This mechanism ensures that funds directly target detailed gaps in hospital readiness.^16^
iii. Insurance implications: Hospitals must maintain a minimum MSS score of 60% in order to receive payments from the National Health Insurance Program. This has been a significant motivating factor for hospitals to improve their quality of care but has yet to be fully implemented.^23^

Beyond policy implications, the HSP/MSS program has fostered a positive competitive spirit among hospitals across the country. A healthcare professional in Rukumkot, noted: “*The MSS program has instilled a newfound drive among hospitals to outdo each other. They are striving to excel not just for the sake of better scores, but to deliver quality healthcare services.*”

When hospitals receive feedback after a routine MSS assessment, photos of exemplary practices at other hospitals are shared with hospital staff during the feedback sessions. This directly “counters failures of imagination”, by both showing what is possible, sharing novel and creative ideas across the country, and rewarding excellence.^24^ A Medical Superintendent echoed these sentiments, “*With the MSS scores, our objectives have transformed. We no longer aim to merely ‘sustain’ our services but to be recognized for providing quality healthcare and setting benchmarks for others. Our clear goal is to offer the best patient care and strive for continuous improvement.*” Thus, the MSS/HSP program has not only operationalized standards but has also kindled a spirit of patient-centered excellence among Nepal’s healthcare institutions (Box 1).

Box 1 **Summary of main lessons learned.**

1. Quality improvement programs can succeed in LMIC hospital settings if assessment tools provide a measurable blueprint towards success and crucially, the resources to address identified gaps.
2. Systematic and transparent assessments, utilizing tools specifically designed for the context, can shift attitudes away from scarcity and develop a culture of excellence, continuous improvement, and healthy competition.
3. Close collaboration with the government secures support, legitimacy, and provides additional levers of motivation and enforcement, thereby ensuring sustainability and long-term impact of the program.

## Conclusion

To address the problem of poor-quality curative health services in Nepal, the novel MSS/HSP program was developed and implemented by NSI under the leadership and in close partnership with the MoHP to identify gaps in hospital readiness while supplying hospitals with financial support to address gaps. This has resulted in the development and national implementation of specialized hospital readiness assessment tools, providing the knowledge of what quality hospital readiness looks like and a blueprint towards that success, enabling hospitals to identify gaps where otherwise this vision was either vague or absent altogether. Crucially, the evaluation was accompanied with the resources to provide agency at the hospital level to implement change. Nationally, the regular assessments have motivated a significant cultural shift from focusing on limitations to an active pursuit of excellence and continuous improvement through the action plans formulated after each assessment. The novel MSS/HSP program is evidence that widespread quality improvement programs can succeed in the LMIC hospital setting if the knowledge of success and the resources to get there are made available.

The close collaboration between NSI and the MoHP at every step of the MSS/HSP program ensured programmatic support at all levels of government. What started as a small pilot in 2013, has now become standardized at the federal level and a key part of achieving Nepal’s Public Service Act of 2075 BS (2018 CE) goal of “*implementing the right to get free basic health service and emergency health service guaranteed by the Constitution of Nepal and establishing access of the citizens to health service by making it regular, effective, qualitative and easily available*”.^25^ This collaborative relationship was key not only for the program’s expansion, but also for its role as an advocacy tool and is instrumental for policy-level decisions and strategic and budgetary planning, highlighting the program’s broader influence. For example, hospitals must obtain a minimum 60% MSS score to receive payments from the National Health Insurance Program, although this is yet to be implemented systematically.^22^ Its integration into the healthcare landscape by the Government of Nepal has ensured its impact, sustainability, and longevity on hospital readiness and health care quality in Nepal.

It is essential to acknowledge that the MSS/HSP program is just the foundation in the journey towards quality health services. Currently, the MSS indicators predominantly focus on structural measures of readiness that a hospital needs to provide mandated curative services. True quality improvement will require a shift toward process and outcome measures. Looking forward, we envision expanding standards to explicitly measure quality and patient outcomes in addition to readiness in close collaboration with the MoHP. Beyond work in Nepal, we believe that the novel MSS/HSP program can be referenced for future work in resource constrained settings, to improve the quality of healthcare services.

## Contributors

RP, RR, and MKS conceptualized the article. PG carried out the original literature search and wrote the first draft with input from RR. AK carried out data curation and data analysis and made Fig. 2A with technical assistance from RR. Together, AK and RR interpreted the data. PP made Fig. 1. RP and AK made Fig. 2B. AK further revised and reviewed the content, with feedback from RR, RP, PP, AA, MKS, and MKU. All authors approved the final version of the manuscript.

## Data sharing statement

All MSS tools, along with relevant indicators and appendixes, are published by the Department of Health Services and can be found in the Supplementary files. Aggregate MSS data and MSS resources, including handbooks and implementation guidelines can be accessed at https://msshealth.org.np/User. Deidentified baseline pilot data collected by NSI from 2014 to 2017 can be made available upon request to the Nick Simons Institute.

## Declaration of interests

RP, MS, PP and AA are employed by the Nick Simons Institute and MU is employed by the Ministry of Health, which developed the program in partnership, but no additional financial support was provided by NSI or any other source. This affiliation may potentially create a competing interest, as the authors may have a vested interest in demonstrating the effectiveness of the program. However, steps were taken to ensure objectivity and impartiality of the study, including involving external researchers, AK, PG, and RR.

This research did not receive any external funding. Funding for the Nick Simons Institute is provided by Nick Simons Foundation. The research outcomes presented in this paper have no effect on the funding received. The authors did not receive grants directly supporting this work. The funders had no role in the study design, data collection and analysis, decision to publish, or preparation of the manuscript. None of the authors’ affiliations or organizations have any financial interest in the outcomes of the research publications. No external entities, apart from the Nick Simons Foundation, provided funding for this research.

## Appendix A Supplementary data

CFIR framework

MSS_Health Post Assessment

MSS_Primary Hospitals Assessment

MSS_Secondary B Hospitals Assessment

MSS_Tertiary Hospitals Assessment
