# Supplementary material for: Minimum service standards assessment tool and the hospital strengthening program: a novel first step towards the quality improvement of Nepal’s national hospital system
Source: Lancet Reg Health Southeast Asia. 2025 Feb 22;34:100548. doi: 10.1016/j.lansea.2025.100548 (PMC11904555; doi:10.1016/j.lansea.2025.100548)
Supplement: CFIR framework [file mmc1.pdf]

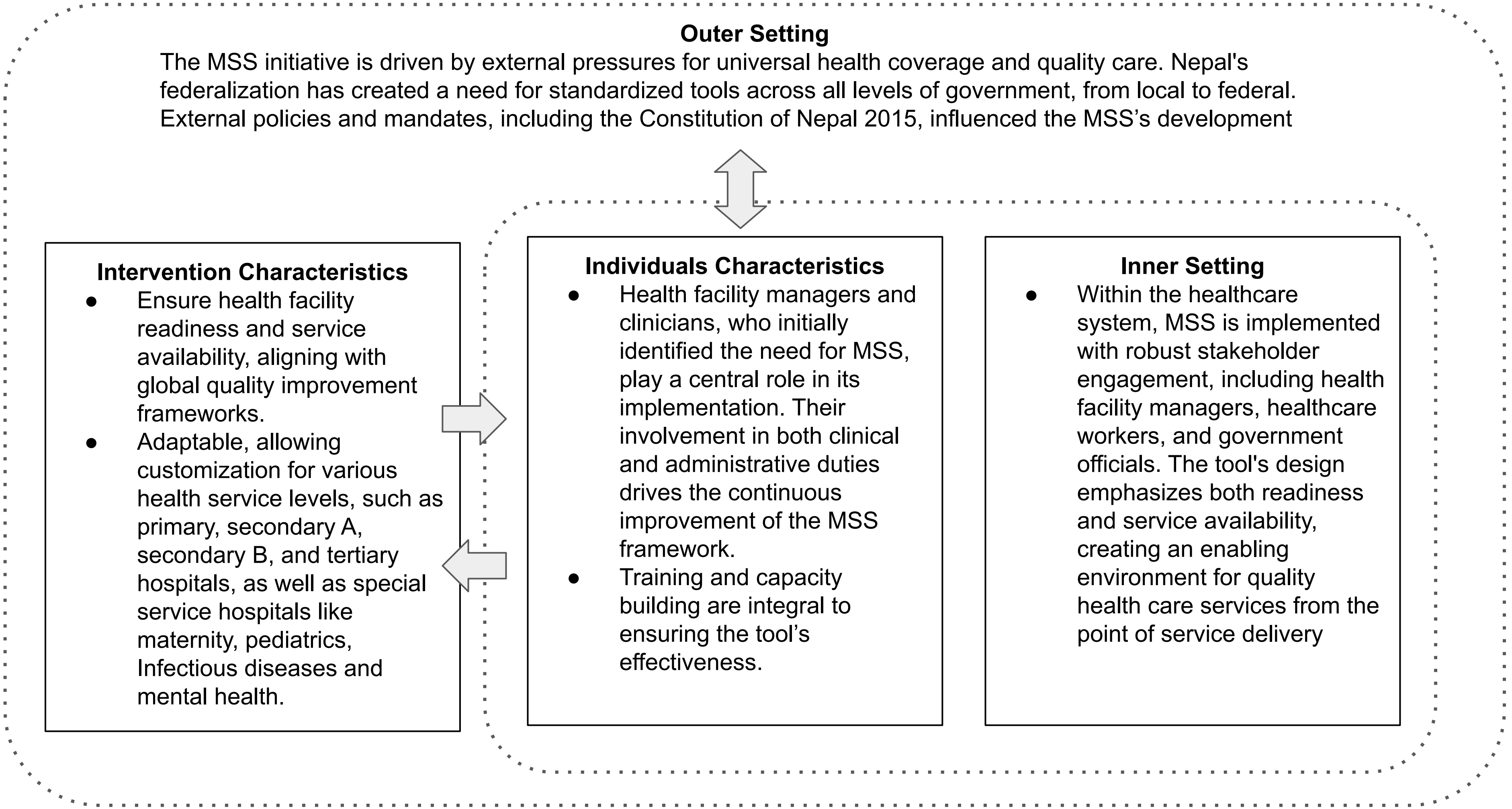
Outer Setting
The MSS initiative is driven by external pressures for universal health coverage and quality care. Nepal's federalization has created a need for standardized tools across all levels of government, from local to federal. External policies and mandates, including the Constitution of Nepal 2015, influenced the MSS's development
Intervention Characteristics
● Ensure health facility readiness and service availability, aligning with global quality improvement frameworks.
● Adaptable, allowing customization for various health service levels, such as primary, secondary A, secondary B, and tertiary hospitals, as well as special service hospitals like maternity, pediatrics, Infectious diseases and mental health.
Individuals Characteristics
● Health facility managers and clinicians, who initially identified the need for MSS, play a central role in its implementation. Their involvement in both clinical and administrative duties drives the continuous improvement of the MSS framework.
● Training and capacity building are integral to ensuring the tool's effectiveness.
Inner Setting
● Within the healthcare system, MSS is implemented with robust stakeholder engagement, including health facility managers, healthcare workers, and government officials. The tool's design emphasizes both readiness and service availability, creating an enabling environment for quality health care services from the point of service delivery
