# Supplementary material for: Minimum service standards assessment tool and the hospital strengthening program: a novel first step towards the quality improvement of Nepal’s national hospital system
Source: Lancet Reg Health Southeast Asia. 2025 Feb 22;34:100548. doi: 10.1016/j.lansea.2025.100548 (PMC11904555; doi:10.1016/j.lansea.2025.100548)
Supplement: MSS_Health Post Assessment [file mmc2.pdf]

न्यूनतम सेवा मापदण्ड
(Minimum Service Standard)

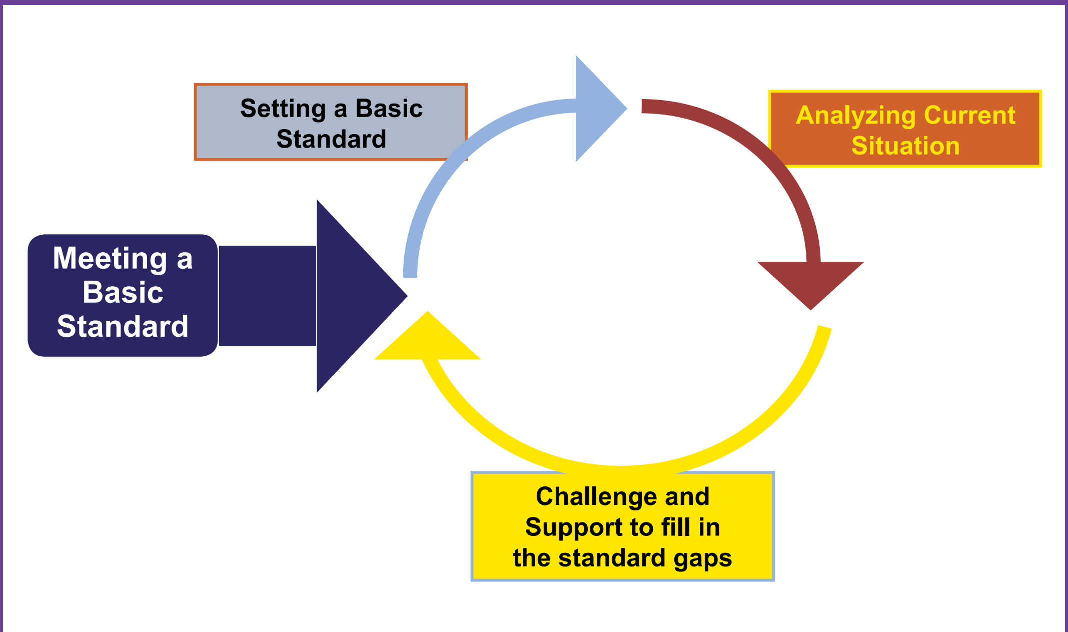

स्वास्थ्य चौकीमा गुणस्तरीय सुधार ल्याउनका लागि कमीकमजोरीहरुको पहिचान गर्ने चेकलिष्ट

नेपाल सरकार
स्वास्थ्य तथा जनसंख्या मन्त्रालय
स्वास्थ्य सेवा विभाग
उपचारात्मक सेवा महाशाखा
२०७६

Print supported by UKaid

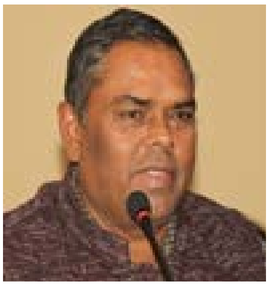

सन्देश

नेपाल सरकार सम्पूर्ण नेपाली जनतालाई न्यूनतम आधारभूत स्वास्थ्य सेवा निःशुल्क उपलब्ध गराउन प्रतिबद्ध छ । नेपालको संविधान २०७२ ले स्वास्थ्यलाई मौलिक हकको रूपमा स्थापित गरेको छ । राष्ट्रिय स्वास्थ्य नीति २०१४, नेपाल स्वास्थ्य क्षेत्र रणनीति २०१५-२०२० र जनस्वास्थ्य सेवा ऐन २०१८ ले सेवाग्राहीलाई गुणस्तरिय स्वास्थ्य सेवामा जोड दिएका छन् ।

स्वास्थ्य तथा जनसंख्या मन्त्रालयको प्रमुख लक्ष्य गुणस्तरीय स्वास्थ्य सेवा उपलब्ध गराउनु हो । सेवाको गुणस्तर बढाउन विगतमा विभिन्न कार्यहरु गरिएका छन् । स्वास्थ्य चौकीका लागि तयार गरिएको यस न्यूनतम सेवा मापदण्ड (MSS), स्वास्थ्य सेवाहरूको उपलब्धता र गुणस्तर सुधारका लागि प्रभावकारी माध्यम साबित हुनेछ । यसले -एकिकृत रुपमा सेवा सुधारको लागि सुशासन र व्यवस्थापनमा सुधारका साथै चिकित्सकीय सेवाको व्यवस्थापन गर्न सहयोग गर्नेछ । स्वास्थ्य तथा जनसंख्या मन्त्रालयले प्रदेश र स्थानीय तहसंग समन्वय गरेर यस -मापदण्डलाई सबै -सम्बन्धित स्वास्थ्य संस्थाहरुमा लागू गरेर सम्पूर्ण नेपाली जनतालाई गुणस्तरीय स्वास्थ्य सेवा सुनिश्चित गर्ने कार्यलाई अगाडि बढाउने छ ।

म यस 'स्वास्थ्य चौकीको लागि न्यूनतम सेवा मापदण्ड (MSS)' को विकासमा रचनात्मक भूमिका खेलेकोमा स्वास्थ्य सेवा विभाग, उपचारात्मक सेवा महाशाखा, गुणस्तर मापन तथा नियमन महाशाखा तथा विज्ञ र साझेदारहरुप्रति धन्यवाद प्रकट गर्दछु । स्वास्थ्य तथा जनसंख्या मन्त्रालय यस मापदण्डको सफलतापूर्वक कार्यान्वयन गर्न प्रतिबद्द रहनेछ ।

....

उपेन्द्र यादव

माननीय उपप्रधानमन्त्री - एवं स्वास्थ्य तथा जनसंख्या मन्त्री

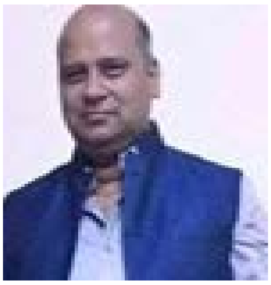

सन्देश

नेपालको स्वास्थ्य क्षेत्रको लागि नेपाल स्वास्थ्य क्षेत्र रणनीति २०१५-२०२० प्रमुख दस्तावेज हो, र यसले गुणस्तरलाई स्वास्थ्य सेवाप्रवाहको अभिन्न अंगको रुपमा लिएको छ । जनस्वास्थ्य सेवा ऐन २०१८ ले पनि सम्पूर्ण नेपाली जनतालाई न्यूनतम आधारभूत स्वास्थ्य सेवा निःशुल्क हुनुपर्ने व्यवस्था गरेको छ । तसर्थ सम्पूर्ण नेपाली जनतालाई गुणस्तरीय स्वास्थ्य सेवा प्रदान गर्नको लागि स्वास्थ्य चौकीको सुदृढीकरण स्वास्थ्य तथा जनसंख्या मन्त्रालयको प्रमुख प्राथमिकतामा पर्दछ ।

न्यूनतम सेवा मापदण्ड (MSS) अस्पताल तहमा सन् २०१४ देखि नै लागू हुँदै आएको छ । जिल्ला अस्पतालमा MSS कार्यान्वयनका क्रममा आर्जन गरिएको सिकाई र यसबाट भएका सकारात्मक परिवर्तनहरुका आधारमा स्वास्थ्य तथा जनसंख्या मन्त्रालय-स्वास्थ्य मन्त्रालयले सबै तहका स्वास्थ्य संस्थाहरुमा MSS लागू गर्ने कार्यलाई प्राथमिकता दिएको हो । -स्थानीय तहमा प्रवाह गरिने स्वास्थ्य सेवाको गुणस्तर सुधारका लागि 'स्वास्थ्य चौकीको MSS' कार्यक्रम सहयोगी हुनेछ । यो मापदण्ड स्थानीय तहमा स्वास्थ्य सेवाको गुणस्तरको अनुगमन गर्नका लागि समेत प्रमुख साधन पनि हुनेछ भन्ने अपेक्षा लिएकोछु ।

स्वास्थ्य चौकीको लागि न्यूनतम सेवा मापदण्ड तयार गर्न योगदान दिनुहुने सम्पूर्ण विज्ञ र साझेदारहरुप्रति म धन्यवाद ज्ञापन गर्न चाहन्छु । यो मापदण्ड कार्यान्वयनका लागि - स्वास्थ्य चौकी प्रमुखको प्रमुख भूमिका रहनेछ साथै -संघ प्रदेश र स्थानीय तहद्वारा स्रोत-साधनको व्यवस्था र MSS ले पहिचान गरेका समस्याहरुको सम्बोधनमा निरन्तर सहयोग जरुरी हुनेछ । अन्त्यमा, म यस MSS को सफल कार्यान्वयनको लागि सवै तहबाट निरन्तर प्रतिबद्धता र सहयोगको आशा गर्दछु ।

....

डा सुरेन्द्र कुमार यादव
माननीय राज्यमन्त्री स्वास्थ्य तथा जनसंख्या मन्त्रालय

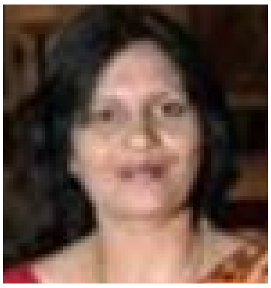

भूमिका

राष्ट्रिय स्वास्थ्य नीति २०१४, नेपाल स्वास्थ्य क्षेत्र रणनीति २०१५-२०२० र जनस्वास्थ्य सेवा ऐन २०१८ ले गुणस्तरिय स्वास्थ्य सेवा प्रवाहमा विशेष जोड दिएका छन् । स्वास्थ्य तथा जनसंख्या मन्त्रालयले स्वास्थ्य सेवाको उपलब्धता तथा गुणस्तर सुधारका लागि न्यूनतम सेवा मापदण्ड (MSS) जिल्ला तहका अस्पतालमा सन् २०१४ देखि नै लागू गर्दै आएको छ । न्यूनतम सेवा मापदण्डको (MSS) कार्यान्वियनबाट प्राप्त उपलब्धि - उत्साहजनक रहेका कारण स्वास्थ्य तथा जनसंख्या मन्त्रालयले यो न्यूनतम सेवा मापदण्ड सबै तहका स्वास्थ्य संस्थामा - कार्यान्वियन गर्न लागेको हो ।

विभिन्न विज्ञहरु र स्वास्थ्य सेवा प्रदायकसँग वृहत् छलफलपछि यो 'स्वास्थ्य चौकीको लागि MSS' को -निर्माण गरिएको हो । यसको कार्यान्वियनले स्वास्थ्य चौकीहरुको व्यवस्थापनमा सुधार आउनुका साथै त्यहाँबाट प्रवाहहुने सेवाको गुणस्तरमा सुधार ल्याउन विशेष भूमिका खेल्नेछ । स्वास्थ्य सेवाको मूल्यांकन गर्नका लगि समेत यो एउटा प्रभावकारी साधनको रुपमा प्रयोग हुने मेरो आशा छ । 'स्वास्थ्य चौकीको MSS प्रयोग गरी, नियमित स्वमूल्यांकन गरी पहिचान गरिएका समस्याहरुको समाधान गर्न सकिने छ । यस मापदण्डको कार्यान्वियनका लागि आवश्यक सहयोग प्रदान गर्न स्वास्थ्य तथा जनसंख्या मन्त्रालय कटिबद्ध छ र यसले पहिचान गरेका समस्याहरुको समाधानका लागि सबै तहहरुको सहयोग हुने अपेक्षा गर्दछु ।

यो दस्तावेज तयार पार्नका लागि सहयोग गर्नुहुने स्वास्थ्य सेवा विभाग र यस मन्त्रालय अन्तर्गतका महाशाखाहरूलाई म धन्यवाद दिन चाहन्छु । साथै यो मापदण्ड विकासमा योगदान दिनुहुने सम्पूर्ण विज्ञ र साझेदारहरुप्रति म धन्यवाद ज्ञापन गर्न चाहन्छु, एवं यो मापदण्डको सफल कार्यान्वियन गर्नको लागि निरन्तर सहयोगको आशा राख्दछु ।

....

डा. पुष्पा-चौधरी

सचिव

स्वास्थ्य तथा जनसंख्या मन्त्रालय

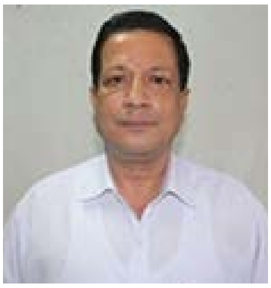

दुई शब्द

स्वास्थ्य संस्थाहरुले प्रदान गर्ने सेवाहरुको गुणस्तर सुधारका लागि आवश्यक तयारी तथा सो को सुनिश्चितताका लागि यो 'स्वास्थ्य चौकीको लागि न्यूनतम सेवा मापदण्ड, MSS' एउटा कोशेढुङ्गा हुनेछ । जनस्वास्थ्य सेवा ऐन २०१८ ले पनि सम्पूर्ण नेपाली जनतालाई न्यूनतम आधारभूत स्वास्थ्य सेवा निःशुल्क हुनुपर्ने व्यवस्था गरेअनुसारको गुणस्तरीय सेवा प्रत्याभूत गराउन स्वास्थ्य तथा जनसंख्या मन्त्रालय प्रतिबद्ध रहेको छ । स्वास्थ्य संस्थाहरुले प्रदान गर्ने सेवाहरुकालागि आवश्यक पर्ने उपकरण र तिनको भण्डारण र दक्ष जनशक्ति लगायतका न्यूनतम आवश्यकता परिपूर्ति गर्ने उदेश्यले यो मापदण्ड तयार गरिएको छ। नेपाल स्वास्थ्य क्षेत्र रणनीति २०१५-२०२० ले गुणस्तरलाई एक प्रमुख आयामको रुपमा निर्दिष्ट गरेअनुसार यस मापदण्डलाई सम्बन्धित सबै स्वास्थ्य संस्थाहरूमा लागू गर्न आवश्यक छ ।

MSS एउटा -स्वमूल्यांकन साधन हो जसले गुणस्तरीय स्वास्थ्य सेवा प्रदान गर्नमा देखिएका कमी कमजोरीहरूको पहिचान गर्छ । पत्ता लागेका कमी कमजोरीलाई सम्बोधन गरी सेवामा सुधारको लागि सम्बन्धीत स्वास्थ्य संस्थाले कार्ययोजना निर्माण गरी त्यसलाई कार्यान्वयन गर्दछ । स्वास्थ्य चौकीका लागि तयार गरिएको यस दस्तावेजलाई तीन भागमा विभाजन गरिएको छ- सुशासन र व्यवस्थापन, क्लिनिकल सेवा व्यवस्थापन, र सहयोग सेवा जसका लागि क्रमानुसार २०%, ६०%, र २०% अंकभार छुट्ट्याइएको छ ।

यो महत्वपूर्ण दस्तावेज तयार गरेकोमा स्वास्थ्य सेवा विभाग, उपचारात्मक सेवामहाशाखा, तथा साझेदारहरुलाई म बधाई दिन चाहन्छु । यस MSS को सबै स्वास्थ्य चौकीहरुमा सफल कार्यान्वयनको आशा गर्दछु र यसबाट आर्जित ज्ञानको अभिलेख राख्दै समय अन्तरालमा MSS को पुनरावलोकन गरिनेछ भन्ने आशा गर्दछु ।

....

डा सुशीलनाथ प्याकुरेल

प्रमुख विशेषज्ञ

स्वास्थ्य तथा जनसंख्या मन्त्रालय

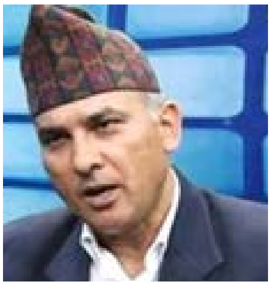

आभार

अस्पतालको लागि न्यूनतम सेवा मापदण्ड सन् २०१४ मा स्वास्थ्य तथा जनसंख्या मन्त्रालयको अस्पताल व्यवस्थापन सुधार कार्यक्रम अन्तर्गत शुरू गरिएको थियो। सोहि कार्यक्रमको सिकाईको आधारमा हामी न्यूनतम सेवा मापदण्ड तर्फ अग्रसर भएका छौं ।

न्यूनतम सेवा मापदण्डको सुरुवाती चरणदेखि नै संलग्न रहेको हुनाले, न्यूनतम सेवा मापदण्डको कारणले गर्दा आएका सकारात्मक परिवर्तनहरु यो कार्यक्रम लागू भएका स्वास्थ्य संस्थाहरुमा मैले देख्दै आएको छु । मैले यस स्वास्थ्य चौकीको लागि न्यूनतम सेवा मापदण्ड दस्तावेज स्वास्थ्य चौकीले प्रदान गर्ने गुणस्तरीय स्वास्थ्य सेवाको पूर्ण तयारीको लागि महत्वपूर्ण दस्तावेज साबित हुने विश्वास लिएको छु ।

यो दस्तावेज तयार गर्नमा प्रमुख भमिका निर्वाह गर्ने स्वास्थ्य सेवा विभाग अन्तर्गतका उपचारात्मक सेवा महाशाखाको म प्रशंसा गर्दछु। यस महत्वपूर्ण कार्यमा सहयोग गर्नुहुने साझेदारहरु विशेषतः निक साइमन्स ईन्टिच्यूट, DFID/ नेपाल स्वास्थ्य क्षेत्र सहयोग कार्यक्रम र WHO -प्रति पनि आभार व्यक्त गर्दछु, र भविष्यमा यस कार्यक्रमको सफल कार्यान्वयनमा समेत सहयोगको आशा गर्दछु ।

....

डा गुण राज लोहनी
महानिर्देशक
स्वास्थ्य सेवा विभाग
टेकु, काठमाण्डौ ।

विषयसूची

स्वास्थ्य चौकीको विवरण 1

पृष्ठभूमि 10

स्वास्थ्य चौकीको लागि न्यूनतम सेवा मापदण्डहरु 17

खण्ड १ सुसाशन र व्यवस्थापन 17

भाग १ का मापदण्डहरुको संख्या र प्राप्ताङ्कको सारांश 17

सुशासन 17

संस्थागत व्यवस्थापन 20

मानवश्रोत व्यवस्थापन र विकास 22

आर्थिक व्यवस्थापन 24

मेडिकल रेकर्ड र सूचना व्यवस्थापन 25

गुणस्तर व्यवस्थापन 26

खण्ड २: क्लिनिकल सेवा व्यवस्थापन 27

भाग २ का मापदण्डहरुको संख्या र प्राप्ताङ्कको सारांश 27

बहिरङ्ग सेवा (ओ.पि.डि) 28

खोप र वृद्धि अनुगमन 42

परिवार नियोजन सेवा 45

ANC र PNC सेवाहरु 48

DOTS क्लिनिक 52

ड्रेसिङ्ग, ईन्जेक्शन र दैनिक प्रक्रियाहरु (DIRP) 54

औषधि सेवा (डिस्पेन्सरि) 58

बर्थिङ्ग केन्द्र** 66

प्रयोगशाला सेवा (प्रयोगशाला भएका संस्थाको लागि) 79

खण्ड ३ स्वास्थ्य चौकी सहयोग सेवा व्यवस्थापन 81

खण्ड ३ का मापदण्डहरुको सारांश 81

उपकरण प्रशोधन र निर्मलीकरण 82

लन्ड्री सेवा (लुगा धुने व्यवस्था) 85

सरसफाई 87

विध्युत तथा अन्य उर्जाको प्रणाली 89
पानीको स्रोत 89
स्वास्थ्यसेवाजन्य फोहोर व्यवस्थापन 90
सुरक्षा र संरक्षण 91
यातायात साधन र संचार 94
भण्डार (मेडिकल र उपकरण) 94

परिशिष्ट १: 96

स्वास्थ्य चौकीको विवरण

हेल्थ पोस्टको नाम, ठेगाना	
मूल्याङ्कन मिति	
मूल्याङ्कनकर्ता	१.
	२.
	३.
परिच्छेद १ को अङ्कः सुशाशन र व्यवस्थापन	
परिच्छेद २ को अङ्कः क्लिनिकल सेवा व्यवस्थापन मापदण्ड	
परिच्छेद ३ को अङ्कः सहयोग सेवा (Support Services) व्यवस्थापन	
न्यूनतम सेवा मापदण्ड (MSS)को कूल अंक	
न्यूनतम सेवा मापदण्ड (MSS)को अङ्कको रङ समूह	

पृष्ठभूमि

परिचय

नेपालको संविधान २०७२ ले सम्पूर्ण नागरिकको लागि आधारभूत स्वास्थ्यलाई मौलिक अधिकारको रुपमा प्रत्याभूत गरेको छ । जनस्वास्थ्य सेवा ऐन २०७५ ले आधारभूत स्वास्थ्य सेवालाई निःशुल्क हुनुपर्ने व्यवस्था गरेको छ । आधारभूत स्वास्थ्य सेवा प्रदान गर्ने एकल अधिकार मूलतः अभिभारा स्थानीय सरकारलाई छ भने उपकरणको विकास, मापदण्डहरु र निर्देशिका निर्माण गरेर आधारभूत स्वास्थ्य सेवा प्रत्याभूत गराउने जिम्वेवारी स्वास्थ्य तथा जनसंख्या मन्त्रालयको रहेको छ ।

नेपालको स्वास्थ्य प्रणाली अनुसार स्वास्थ्य चौकी पहिलो सेवा प्रदायक संस्था हो । स्वास्थ्य सेवा प्रदान गर्नुको साथै स्वास्थ्य चौकीले महिला स्वास्थ्य स्वंयसेविकाहरुको गतिविधिको नियमन गर्ने र समुदायमा आधारित क्रियाकलापहरु जस्तै ORC, खोप कार्यक्रम समेत संचालन गर्दछ । हाल ३८०८ वटा स्वास्थ्य चौकीहरु रहेका छन् । रिक्त वडाहरूमा नयाँ स्वास्थ्य चौकी खोल्ने नीति रहेको छ।

स्वास्थ्य तथा जनसंख्या मन्त्रालय ले अस्पतालको लागि न्यूनतम सेवा मापदण्ड लागू गरिसकेको अवस्थामा यसबाट सिकेको अनुभवको आधारमा स्वास्थ्य चौकीको लागि न्यूनतम सेवा मापदण्ड विकास गरिएको हो । स्वास्थ्य चौकीको लागि न्यूनतम सेवा मापदण्ड एऊटा तयारी स्वमुल्याङ्कन साधन हो जसले गुणस्तरीय स्वास्थ्य सेवा प्रदान गर्न स्वास्थ्य चौकीले परिपर्ति गर्नुपर्ने न्यूनतम मापदण्डहरु तोकेको छ । स्वास्थ्य चौकीले MSS का मापदण्डहरु भन्दा बढी सेवा दिनेतर्फ उन्मुख हुनका लागि MSS को मापदण्ड पूरा गरेकै हुनुपर्ने छ । हाल भईआएका गुणस्तर सुधार साधनको परिपूरकको रुपमा MSS लाई लिन सकिन्छ किनकि यसले सेवा तयारीलाई परिपूर्णता दिन्छ । यसले कसरी सेवा प्रदान गरिनुपर्छ भन्ने कुरामा ध्यान दिदैन जुन प्रमुख रुपमा मापदण्ड उपचार प्रोटोकलअन्तर्गत पर्दछ ।

MSS को विकास गर्दा, तीनवटा आधारभूत विषयहरुको आधारमा यसको खाका तयार पारिएको छ- सुशासन र व्यवस्थापन, क्लिनकल सेवा व्यवस्थापन र सहयोग सेवा व्यवस्थापन । MSS को विकास मूलतः आधारभूत स्वास्थ्य सेवा प्याकेज २०७५ र नेपाल स्वास्थ्य पूर्वाधार विकास मापदण्ड २०७४ मा आधारित छ । MSS को विकासमा आधार मानिएका अन्य दस्तावेजहरु निम्नानुसार छन्-

- रास्ट्रिय स्वास्थ्य नीति २०७१
- जनस्वास्थ्य सेवा ऐन २०७५
- सुशासन (व्यवस्थापन र संचालन) २०६४
- वित्तिय प्रणाली नियमावली २०६४
- नेपाल स्वास्थ्य सेवा नियमावली २०५५
- लोक सेवा नियमावली २०५०

- नेपाल स्वास्थ्य क्षेत्र रणनीति २०१५-२०२०, स्वास्थ्य तथा जनसंख्या मन्त्रालय, नेपाल सरकार
- नेपाल एकिकृत स्वास्थ्य पूर्वाधार विकास मापदण्ड २०७३|७४
- स्वास्थ्य संस्थाको लागि गुणस्तर सुधार उपकरण २०७४
- स्वास्थ्य क्षेत्रमा सामाजिक लेखापरिक्षण निर्देशिका २०७० पुनरावलोकन २०७३
- अत्यावश्यक औषधीहरुको राष्ट्रिय सूची २०६६\६७ पुनरावलोकन २०७२\७३
- जिल्ला अस्पतालहरुको गुणस्तर सुधारका कमजोरी पहिचान गर्ने न्यूनतम सेवा मापदण्ड चेकलिस्ट, उपचारात्मक सेवा विभाग, स्वास्थ्य तथा जनसंख्या मन्त्रालय, नेपाल सरकार , २०७१\२०७२
- स्वास्थ्य व्यवस्थापन र सूचना प्रणाली, रेकर्डिङ र रिपोर्टिङ, २०७०
- रास्ट्रिय सुरक्षित मात्तृत्व तथा नवजात शिशु स्वास्थ्य कार्यक्रम, जिल्ला मात्तृत्व तथा नवजात शिशु स्वास्थ्य आवश्यकता मूल्यांकन उपकरण भाग ०१, अस्पताल, २०६३\२०६४
- स्वास्थ्य सेवा फोहोर व्यवस्थापन निर्देशिका २०१४, स्वास्थ्य तथा जनसंख्या मन्त्रालय, नेपाल सरकार

स्वास्थ्य चौकीको लागि MSS को औचित्यः

स्वास्थ्य चौकीको लागि MSS को विकासको प्रमुख कारण नै आधारभूत स्वास्थ्य सेवा प्रदान गर्नको लागि स्वास्थ्य चौकीले गर्नुपर्ने औपचारिक तयारीको आवश्यकता हो र यसले प्रमुख रुपमा स्वास्थ्य चौकीको वर्तमान अवस्था र कमजोरीहरुको मूल्यांकन, सूधारको लागि कार्ययोजनाको विकास र - लगानीको लागि प्रमाण संकलनको भूमिका निर्वाह गर्दछ। स्वास्थ्य तथा जनसंख्या मन्त्रालयको लागि स्वास्थ्य चौकीको मूल्यांकन गर्ने अनुगमनको साधनको रुपमा पनि यो प्रयोग हुनेछ।

न्यूनतम सेवा मापदण्डको निर्माण प्रक्रियाः

न्यूनतम सेवा मापदण्डको निर्माण तत्कालिन व्यवस्थापन स्वास्थ्य सेवा विभागले सुरुवात गरेको थियो र यो विभिन्न विभाग\केन्द्र र साझेदारहरुको संलग्रतामा तयार गरिएको हो। जीवनको लागि स्वास्थ्य, निक साइमन्स ईन्टिच्यूट, विश्व स्वास्थ्य संगठन र नेपाल स्वास्थ्य क्षेत्र सहयोग कार्यक्रमका सदस्यहरुको एउटा समिति बनाई यसको निर्माण कार्य थालिएको थियो। सो समितिको दुईवटा वैठकपश्चात् यसको खाका तयार पारिएको हो। पहिलो खाकाको आधारमा, MSS लाई पुनरावलोकन गरिएको थियो र सातवटै प्रदेशका सातवटा स्वास्थ्य चौकीका सेवाप्रदायकहरुसँग छलफल गरिएको थियो।

प्रदेश १:	भेडेटार स्वास्थ्य चौकी, धनकुटा
प्रदेश २:	हनुमाननगर स्वास्थ्य चौकी, सप्तरी
प्रदेश ३:	बुढानिलकण्ठ स्वास्थ्य चौकी, काठमाडौं
गण्डकी प्रदेश:	हेम्जा स्वास्थ्य चौकी, कास्की
प्रदेश ५:	पारसपुर स्वास्थ्य चौकी, बाँके
कर्णाली प्रदेश:	लाटीकोईली स्वास्थ्य चौकी, सुर्खेत
प्रदेश ७:	गेटा स्वास्थ्य चौकी, कैलाली

कोशीदेखा र खरेलथोक स्वास्थ्य चौकीका सेवाप्रदायकहरु, HFOMC सदस्यहरु, पाँचखाल नगरपालिका, काभ्रेपलान्चोकका स्थानीय शिक्षकहरु र स्थानीय जनप्रतिनिधिहरुसँग पनि थप परामर्श गरिएको थियो ।

तत्कालिन व्यवस्थापन महाशाखाले स्वास्थ्य सेवा विभागका विभिन्न महाशाखा र शाखाहरु, विज्ञहरु र प्रतिनिधीहरुको सहभागितामा जून २९, २०१८ मा अन्तिम चरणको कार्यशाला गोष्ठी काठमाडौंमा आयोजना गरेको थियो । साथै यो स्वास्थ्य चौकीको लागि MSS दस्तावेजलाई सुझाव तथा प्रतिकियाको लागि स्वास्थ्य सेवा विभाग को गुणस्तर मापन तथा सुधारसम्बन्धीको प्राविधिक कमिटि र स्वास्थ्य तथा जनसंख्या मन्त्रालयको गुणस्तर मापन अनुगमन कमिटिमा समेत छलफल गरि तयार गरिएको हो । उक्त अन्तिम दस्तावेजलाई स्वास्थ्य तथा जनसंख्या मन्त्रालयका सचिवको अध्यक्षतामा गठित उच्चस्तरीय व्यवस्थापन समितिमा पेश गरिएको थियो । सो समितिबाट आएका सुझाव समेटि अन्तिम रुपमा माननिय स्वास्थ्य तथा जनसंख्या मन्त्री ज्यु बाट स्वीकृत गरि कार्यान्वयनमा ल्याइएको हो ।

मापदण्डहरुको संरचनाः

स्वास्थ्य चौकीको लागि MSS का मापदण्डहरुलाई तीन भाग र त्यसभित्र यसरी राखिएको छ । (चित्र १ स्वास्थ्य चौकीको लागि MSS का मापदण्डहरुको संरचना)

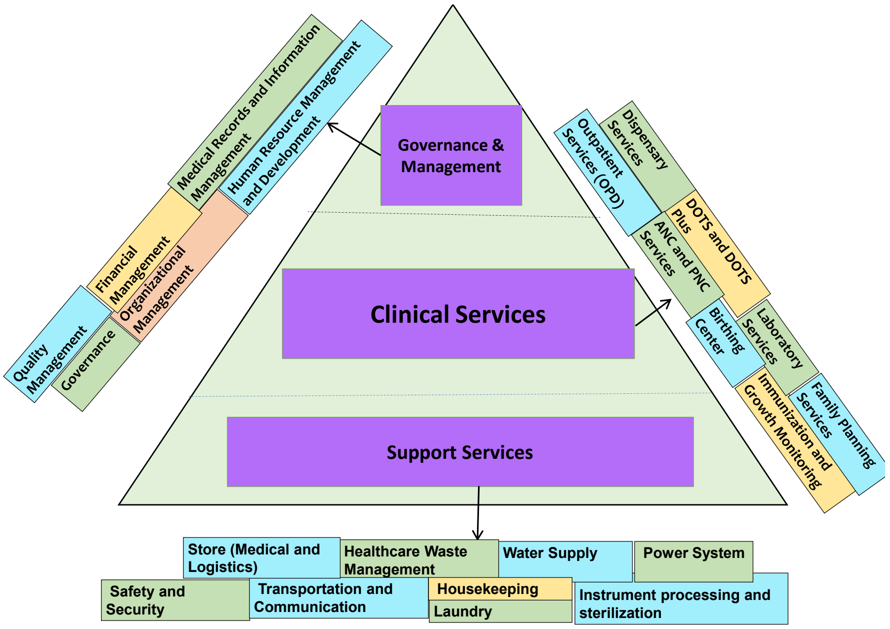

चित्र १ स्वास्थ्य चौकीको लागि MSS का मापदण्डहरुको संरचना

चेकलिष्टलाई कसरी प्रयोग गर्ने ?

स्वास्थ्य चौकीको लागि MSS मूलतः स्वमूल्यांकन को साधन हो । हरेक मापदण्डहरुको प्रमाणिकरणका एक वा सोभन्दा बढी मापदण्डहरुसहितका आ-आफ्नै क्षेत्रहरु छन् । यो चेकलिष्टले वर्तमान अवस्थालाई गणितिय हिसाबमा मापन गर्न सहयोग गर्छ र कमी कमजोरीहरु पहिचान गरी कार्ययोजना तयार पार्न मदत गर्छ । यो एउटा चक्रिय प्रक्रिया हो र यसको विस्तृत जानकारी सोसम्बन्धीको निर्देशिकामा रहेको छ । प्रमुख चरणहरु यसप्रकार छन्-

सामूहिक छलफलः

- तपाईको स्वास्थ्य चौकीले वास्तवमा दिइएका मापदण्डहरुको परिपूर्ति गर्छ कि गर्दैन भनेर तपाईको स्वास्थ्य चौकीमा सामूहिक छलफल गर्नुपर्दछ- ।

चेकलिष्ट भर्नेः

- हरेक भागहरुलाई राम्ररी पढ्ने र स्वास्थ्य चौकीले वास्तवमा दिइएका मापदण्डहरुको परिपूर्ति गर्छ कि गर्दैन हेरेर सोको हरफमा सो मापदण्डको उच्चतम अङ्कको आधारमा ० देखि ३ सम्मको अङ्क दिने ।
- जुन भागमा परिशिष्ट हेर्ने निर्देशन दिइएको छ, त्यसमा प्रतिशत निकालेर स्कोरिङ्ग तालिकाअनुसार गर्नुहोस् र ० देखि ३ सम्मको अङ्क दिने।
- संभव भएसम्म प्रत्येक भागको लागि फरक प्रतिलिपिको प्रयोग गर्ने ताकि मूल्यांकनमा मतभेद नहोस् ।
- सोही प्रक्रियाले सबै मापदण्डहरुको प्राप्ताङ्क (score) गर्नुपर्दछ ।

चेकलिष्टमा अङ्क दिनेः

- प्रत्येक खण्डको कुल अङ्क जोड्ने र प्रतिशतमा बदल्ने ।
- प्रत्येक खण्डको कुल अङ्क जोड्ने र सो भागको औषत निकाल्ने ।

भर्ने तरिकाको नमूनाः

क्षेत्र	कोड	प्रमाणिकरण		
उपकरण प्रशोधन र निर्मलीकरण	३.१			
उपक्षेत्र		मापदण्ड	प्राप्ताङ्क	उच्चतम अङ्क
३.१.१ ठाउँ/ स्थान				
	३.१.१	उपकरण प्रशोधन र निर्मलीकरणको लागि सफा पानीको सुविधासहितको ठाउँको व्यवस्था छ ।	१	१
३.१.२ कर्मचारी परिचालन	३.१.२	उपकरण प्रशोधन र निर्मलीकरणको लागि फरक कर्मचारीको व्यवस्था गरिएको छ ।	१	१
३.१.३ साधन र उपकरण	३.१.३	निर्मलीकरण गर्ने साधन र उपकरणहरु २४ सै घण्टा प्रयोगशील अवस्थामा उपलब्ध छन् । (हेर्नु परिशिष्ट ३.१ क निर्मलीकरण गर्ने साधन र उपकरणहरु)	२	३
३.१.४				
उपकरणको तयारी	३.१.४	न्यापर, गज, कटन बल र पट्टीहरु तयार गरिन्छ ।	१	१
मापदण्ड ३.१		कुल प्राप्ताङ्क	५	६
		प्रतिशत = कुल प्राप्ताङ्क / ६ x १००= ५\६ x १००	८३.३३%	

परिशिष्ट ३.१ क निर्मलीकरण गर्ने साधन र उपकरणहरु			
क्रम	उपकरण	आवश्यक संख्या	अङ्क
१.	काम गर्ने टेबल	१	१
२.	सर्जिकल ड्रम	२	०
३.	भण्डारण दराज	१	१
४.	बोइलर वा मःम पट	१	१
		कुल अङ्क	३
		कुल प्रतिशत = ३/४ X १००	७५

हरेक हरफले १ अङ्क पाउनेछ यदि सबै आवश्यक संख्या छ भने अन्यथा ०

स्कोरिङ तालिका	
कुल प्रतिशत	अङ्क
०-४९	०
५०-६९	१
७०-८४	२
८५-१००	३
मापदण्ड ३.१.३ को लागि अङ्क	२

प्रत्येक भागहरुको अङ्क र कुल MSS अङ्क

मापदण्डहरुको सबै भागहरुको मूल्याङ्कनपछि, कुल अङ्कको लागि सबै भागहरुको हिसाब गरिन्छ । सुशासन र व्यवस्थापनको भाग (भाग १) लाई २०%, क्लिनिकल सेवा व्यवस्थापन (भाग २) लाई ६०% र सहयोग सेवा व्यवस्थापन (भाग ३) लाई २०% दिइन्छ। उदाहरणः

यदि भाग १ को कुल अङ्क ८०%, भाग २ को ६०% र भाग ३ को ८०% छ भने, कुल MSS अङ्क यसरी निकालिन्छः

कुल MSS अङ्क = (०.२x भाग १ + ०.६ x भाग २+ ०.२ x भाग ३)%

कुल MSS अङ्क = (०.२x ८०% + ०.६ x ६०%+ ०.२ x ८०%)

कुल MSS अङ्क = ६८%

कुल MSS अङ्क र कलर कोडिङ्गः

कुल MSS अङ्कको आधारमा स्वास्थ्य संस्थाको कलर कोडिङ्ग यसरी गरिन्छ ।

कुल MSS अङ्क (%)	कलर कोडिङ्ग	
५० भन्दा कम	सेतो	
५०-६९	पहेँलो	
७०-८४	निलो	
८५-१००	हरियो	

माथिको उदाहरणमा कुल MSS अङ्क (%) ६८% छ, त्यसैले सो स्वास्थ्य चौकीलाई पहेँलो कलर कोडिङ्ग गरिन्छ र त्यसको MSS अङ्क जनाउन त्यसलाई पहेँलो -स्टिकर दिइन्छ।

स्वास्थ्य चौकीको लागि न्यूनतम सेवा मापदण्डहरु

खण्ड १ सुसाशन र व्यवस्थापन

भाग १ का मापदण्डहरुको संख्या र प्राप्ताङ्कको सारांश

क्षेत्र	कुल मापदण्ड संख्या	कुल अङ्क	कुल प्राप्ताङ्क (प्रतिशतमा)
सुसाशन	२४ (२५**)	२४ (२५**)	
संस्थागत व्यवस्थापन	१५	१५	
स्रोत मानव श्रोत व्यवस्थापन र विकास	११ (१२**)	१३ (१५**)	
आर्थिक व्यवस्थापन		८ (९**)	८ (९**)
मेडिकल रेकर्ड र सूचना व्यवस्थापन	८	८	
गुणस्तर व्यवस्थापन		८	१०
कुल	७४ (७७**)	७८ (८२**)	

* *बर्थिङ्ग सेन्टर भएका स्वास्थ्य चौकीका लागि

क्षेत्र	कोड	प्रमाणीकरण		
सुशासन	१.१			
उपक्षेत्र उपक्षेत्र		मापदण्डहरु	प्राप्ताङ्क	उच्चतम अङ्क
१.१.१ स्वास्थ्य संस्था संचालन र व्यवस्थापन समिति (HFOMC) को निर्माण	१.१.१	स्वास्थ्य तथा जनसङ्ख्या मन्त्रालय\ प्रदेश \स्थानीय सरकार को निर्देशिका अनुसार HFOMC को निर्माण गरिएको छ।		१
१.१.२ HFOMC को क्षमता विकास	१.१.२	सबै HFOMC सदस्यहरुले HFOMC को कार्य र आधारहरु बारेमा अभिमुखिकरण पाएका छन् ।		१
१.१.३ स्वास्थ्य चौकी प्रमुखको पदपुर्ति	१.१.३	स्वास्थ्य चौकी प्रमुखको पद संगठनिक संरचना अनुसार पदपूर्ति भएको छ ।		१
१.१.४ HFOMC को सन्चालन	१.१.४.१	सदस्य सचिव वा स्वास्थ्य चौकी प्रमुखले HFOMC को बैठक बोलाउने र त्यसको अध्यक्षता HFOMC का अध्यक्षले गर्ने गरेको (कम्तिमा ३ महिनामा १ पटक) र आवश्यकता अनुसार ।		१

	१.१.४.२	HFOMC का बैठकहरुले कम्तिमा निम्न एजेण्डा राखेको (पहिलेका बैठकहरुको माइन्युट हेर्ने।		
	१.१.४.२.१	स्वास्थ्य चौकीका सेवाहरुको उपलब्धता		१
	१.१.४.२.२	स्वास्थ्य चौकीका सेवाहरुको उपभोग र उद्देश्य जस्तैः सिमान्तकृत र पिछडिएका वर्गले उपभोग गरेका सेवाहरु, प्रेशण		१
	१.१.४.२.३	स्वास्थ्य चौकीको आन्तरिक स्रोतहरुको उपयोग		१
	१.१.४.२.४	बिरामीका अधिकारका विषयहरु जस्तैः बिरामीका सुविधा, प्राप्त उजूरीहरुको मुल्याङ्कन, बिरामीको सुरक्षा, संक्रमण रोकथाम।		१
	१.१.४.२.५	व्यवस्थापनका विषयहरुः मानव स्रोत, सुरक्षा		१
	१.१.४.२.६	पूर्वाधार \ औजारका विषयहरु		१
	१.१.४.२.७	स्थानीय सरकार \ गाऊँपालिका \ नगरपालिका \ प्रेषण अस्पताल \ सङ्घ \ प्रदेश \ DoHS \ स्वास्थ्य तथा जनसंख्या मन्त्रालय सँग समन्वय।		१
	१.१.४.२.८	स्टाफ मिटिङग् र गुणस्तर सम्वन्धिका बैठकका निर्णय र सुझावहरुको मुल्याङ्कन।		१
१.१.५ वार्षिक कार्य योजना	१.१.५	आर्थिक वर्षको सुरुवातसंगै वार्षिक योजना बनाई HFOMC बाट स्वीकृत गराइएको छ।		१
१.१.६ HFOMC कागजातको भण्डारण	१.१.६	HFOMC कागजातको भण्डारण गर्नको लागि छुट्टै दराजको व्यवस्था छ।		१
१.१.७ जवाफदेहीता	१.१.७.१	नागरिक वडापत्र राखिएको छ।		१
	१.१.७.२	सार्वजनिक सरोकारका सूचनाहरु सार्वजनिक गरिएको।		१
	१.१.७.३	देखिने गरि उजुरी पेटिका राखिएको र उजूरीहरुलाइ नियमित रुपमा सम्बोधन गरिएको।		१
	१.१.७.४	उपलब्ध सेवाहरु (सामुदायिक स्वास्थ्य ईकाईका संख्याहरु र खोप केन्द्रहरु, PHC-ORC)को विवरण देख्ने गरी राखिएको छ।		१

	१.१.७.५	परिस्कृत सामाजिक नक्सा जसमा कार्यक्षेत्र र सिमान्तकृत र पिछडिएका वर्गको जानकारी छ, सो नक्सा सबैले देख्नेगरी राखिएको।		१
	१.१.७.६	निशुल्क औशधिहरुको सूचि र त्यसको उपलब्धता सबैले देख्नेगरी राखिएको ।		१
	१.१.७.७	आमा सहुलियत पाएका आमाहरुको नामावली सूचना पाटीमा सबैले देख्नेगरी टाँस गरिएको।		१
१.१.८ आचरण र सामाजिक परिक्षण	१.१.८	पहिलेको वर्षको सामाजिक परीक्षण गरिएको छ ।		१
१.१.९ स्वास्थ्य संस्थाका पूर्वाधारहरु	१.१.९.१	निर्देशिका अनुसार स्वास्थ्य संस्था भएको जग्गा स्वास्थ्य संस्थाकै नाममा छ ।		१
	१.१.९.२	स्वास्थ्य संस्थाको आफ्नै भवन छ जुन निर्देशिकाअनुसार निर्माण भएको छ ।		१
१.१.१० स्वास्थ्य संस्थालाई सामुदायिक सहयोग	१.१.१०	स्वास्थ्य संस्थामा स्थानीय सरकारको सहयोगमा आवश्यकताअनुसार करारनामा गरेका कर्मचारीहरु राखिएका छन् ।		१
मापदण्ड १.१		कुल प्राप्ताङ्क		२५
		प्रतिशत = कुल प्राप्ताङ्क् \ २५ x १००		

क्षेत्र	कोड	प्रमाणीकरण		
संस्थागत व्यवस्थापन	१.२			
उपक्षेत्र उपक्षेत्र		मापदण्डहरु	प्राप्ताङ्क	उच्चतम अङ्क
१.२.१ संस्थागत संरचना	१.२.१.१	अध्यावधिक गरिएको स्वास्थ्य संस्थाका कर्मचारी र HFOMC सदस्यहरुको नामावलीसहितको संस्थागत संरचना सार्वजनिक गरिएको ।		१
	१.२.१.२	स्वास्थ्य चौकी अन्तर्गतका FCHVs[1] को अध्यावधिक गरिएको नामावली सार्वजनिक गरिएको ।		१
१.२.२ कार्य विभाजन र जिम्मेवारी बाँडफाड	१.२.२	जिम्मेवारी बाँडफाडको लिखित अभिलेख राखिएको छ ।		१
१.२.३ सेवाग्राहीको भीड व्यवस्थापन	१.२.३	दिशामूलक सूचनासँगै उपलब्ध सेवाहरु, कोठा नं र अधिकारीको नामसहितको जानकारीको उपलब्धता छ ।		१
१.२.४ पंङ्ति व्यवस्थापन	१.२.४	स्वास्थ्य चौकीले प्रथम आउनेलाई पहिला हेर्ने पद्दतिअनुसार सेवा दिन्छ र अत्यावश्यक, वृद्ध, फरक क्षमता भएका व्यक्ति र गर्भवतीलाई ग्राह्यता दिन्छ ।		१
१.२.५ हाजिरी	१.२.५	सबै कर्मचारीहरुले आफ्नो दैनिक हाजिरिको रेकर्ड राख्ने गर्छन् ।		१
१.२.६ सबै कर्मचारीको पोशाक	१.२.६.१	सबै कर्मचारीले ड्युटीको समयमा पोशाक वा एप्रोन लगाउँछन् ।		१
	१.२.६.२	सबै कर्मचारीले ड्युटीको समयमा आफ्नो परिचयपत्र लगाएका हुन्छन् ।		१
१.२.७ प्रवाभकारी सामूहिक काम गर्ने वातावरणको व्यवस्था	१.२.७.१	कर्मचारीहरुको बैठक मासिक रुपमा बस्छ ।		
	१.२.७.३	कर्मचारी बैठकले निम्न एजेण्डाहरु संलग्न गर्छ । (बैठक माइन्यूट हेर्ने)		१
	१.२.७.३.१	कर्मचारीको क्षमता विकास र कर्मचारीको कामको फाँट परिवर्तन ।		१
	१.२.७.३.२	सेवाको पहुँच र उपभोगसगै त्यसको अभिलेखिकरण र रिपोर्टिङ्ग ।		१
	१.२.७.३.३	FCHV को उपभोग र मूल्याङ्कन र त्यसको लागि आवश्यक सहयोग ।		१
	१.२.७.३.४	स्कुल स्वास्थ्य, स्वास्थ्य शिविर, आमा समूहजस्ता विशेष स्वास्थ्य सुधारात्मक कार्यक्रमहरु गरेको ।		१

1 Female Community Health Volunteers

	१.२.७.४	कर्मचारीहरुको लागि स्तनपानको छुट्टै स्थानको व्यवस्था गरिएको ।		१
१.२.८सडक संजाल	१.२.८	स्वास्थ्य चौकीको सडक संजालको लागि HFOMC ले समन्वय गर्ने ।		१
मापदण्ड		कुल प्राप्ताङ्क		१५
		प्रतिशत = कुल प्राप्ताङ्क् \ १५ x १००		

क्षेत्र	कोड	प्रमाणीकरण		
**मानवश्रोत व्यवस्थापन र विकास**	**१.३**			
**उपक्षेत्र उपक्षेत्र**		**मापदण्डहरु**	**प्राप्ताङ्क**	**उच्चतम अङ्क**
१.३.१ कर्मचारी प्रशासन	१.३.१	HFOMC को कर्मचारी प्रशासनको निर्देशिकाअनुसार कर्मचारी बिनिमय तयार छ । (सबै कर्मचारीहरु- स्थानीय करारका कर्मचारीसहित) ।		१
१.३.२ मानव स्रोतको अभिलेख	१.३.२	सबै कर्मचारीहरु - स्थानीय करारका कर्मचारीहरुसहितको व्यक्तिगत विवरणहरु राखिएको र अध्यावधिक गरिएको ।		१
१.३.३ कर्मचारी व्यवस्थापन	१.३.३.१	स्वास्थ्य चौकीको संरचनाअनुसार कर्मचारीको उपलब्धता । (हे. परिशिष्ट १.३क यस मापदण्डको अन्त्यमा रहेको दरबन्दी विवरण)		३
	१.३.३.२	संबन्धित निकायमा रिक्त दरबन्दी माग फाराम भरेर पठाएको ।		१
	१.३.३.३	लामो बिदा जस्तै अध्ययन बिदामा कर्मचारी जाँदा उनिहरुको रिक्त पदमा वैकल्पिक कर्मचारीको व्यवस्था भएको ।		१
	१.३.३.४	यदि स्वास्थ्य चौकी बर्थिङ्ग सेन्टर हो भने, प्रजनन् सेवा दिनको लागि कम्तिमा २ जना SBA तालिम लिएको अ.न.मी वा स्टाफ नर्सको उपलब्धता छ ।		१**
१.३.५ कामकोमुल्याङ्कन	१.३.५.१	सबै कर्मचारीहरुको कार्य सम्पादन मूल्याङ्कन गर्ने गरेको।		१
१.३.६ कर्मचारीलाई उत्प्रेरणा र व्यवसायिक सुरक्षा	१.३.६.१	कार्य सम्पादन मूल्याङ्कनको आधारमा कर्मचारीहरुको तालिम सम्बन्धीका आवश्यकता हेरेर स्वास्थ्य चौकीको लागि तालिम योजना बनाइन्छ ।		१

	१.३.६.३	वर्षमा कम्तिमा एकचोटि कर्मचारीलाई उत्प्रेरणा दिने कार्यक्रमहरु गरिन्छ (कर्मचारीलाई पुरस्कार, कार्यक्षमता पहिचान आदि)।		१
	१.३.६.४	स्वास्थ्य चौकीमा पेशागत खतरा (धारिला वस्तुको चोट) लाई समाधान गर्ने र खोपको व्यवस्था छ ।		१
१.३.७ बर्थिङ्ग केन्द्रमा कर्मचारीको लागि क्वार्टरको व्यवस्था	१.३.७	२४ घण्टा सेवा दिने बर्थिङ्ग केन्द्रमा नर्सिङ्ग कर्मचारीको लागि क्वार्टरको व्यवस्था भएको ।		१**
१.३.८ बैठकको लागि हल/कोठा	१.३.८	स्वास्थ्य चौकीमा बहुप्रयोगमा ल्याउन सकिने कोठा/हलको ब्यवस्था भएको।		१
मापदण्ड १.३		कुल अङ्क		१३ (१५**)
		प्रतिशत = कुल अङ्क / १३ वा १५**x १०० * *बर्थिङ्ग केन्द्रको लागि		

परिशिष्ट १.३ स्वास्थ्य चौकीको दरबन्दी विवरण

क्रमाङ्क	कर्मचारी दरबन्दी	आवश्यक संख्या	स्कोर	Maximum स्कोर
१.	स्वास्थ्य चौकी प्रमुख	१		१
२.	अ.न.मी[2]	२-३		१
३.	अ.हे.व [3]	२-३		१
४.	कार्यालय सहयोगी	१		१
	कुल स्कोर			४
	कुल प्रतिशत = कुल स्कोर/ ४ x१००			

हरेक हरफले १ अङ्क पाउनेछ यदि सबै आवश्यक संख्या छ भने अन्यथा 0

स्कोरिङ्ग तालिका	
कुल प्रतिशत	स्कोर
०-४९	०
५०-६९	१
७०-८४	२
८५-१००	३
मापदण्डको स्कोर १.३.३.१	

2 Hills & Mountain-२, Terai- 3
3 Hills & Mountain-२, Terai- 3

क्षेत्र	कोड	प्रमाणीकरण		
आर्थिक व्यवस्थापन	१.४			
उपक्षेत्र उपक्षेत्र		मापदण्डहरु	प्राप्ताङ्क	उच्चतम अङ्क
१.४.१ वार्षिक कार्ययोजनाको निर्माण र स्विकृति	१.४.१.१	सरकारी अनुदान र कार्यक्रमको अनुदानबाट भएको आम्दानीअनुसार एउटा वार्षिक कार्ययोजनाको निर्माण गरिन्छ ।		१
	१.४.१.२	वार्शिक कार्ययोजनामा अन्य संस्थाबाट उपलब्ध स्रोत र स्बास्थ्य संस्थाको आन्तरिक आम्दानी समेत समावेश गरिन्छ ।		१
१.४.२ सेवा शुल्क	१.४.२.१	स्वास्थ्य चौकीबाट आधारभूत स्वास्थ्य सेवा निशुल्क प्रदान गरिन्छ ।		१
	१.४.२.२	आधारभूत स्वास्थ्य सेवा बाहेकका अन्य स्वास्थ्य सेवाहरुको सेवा शुल्क HFOMC ले प्रत्येक वर्ष निर्धारण गर्नेछ ।		१
१.४.३ आमा सुरक्षा कार्यक्रम रकम र अन्य(बर्थिङ्ग सेन्टर भएका स्वास्थ्य चौकीका लागि)	१.४.३	आमा सुरक्षा कार्यक्रमको सदुपयोग गर्ने र अन्य अनुदानको प्रयोग गर्ने गर्छ ।		१
१.४.४ँ बैक खाता	१.४.४	स्वास्थ्य चौकीको बैंक खाता निर्देशिका अनुसार समितिको अध्यक्ष र स्वास्थ्य चौकी प्रमुखको संयुक्त हस्ताक्षरबाट संचालन गरिन्छ ।		१
१.४.५ बेरूजु फर्स्योट	१.४.५.१	बेरूजु फर्स्योटको बारे कारवाही ३५ दिनभित्र गरिन्छ ।		१
	१.४.५.२	बेरूजु फर्स्योटको नियमन राश्ट्रिय लक्ष्य अनुसार गरिन्छ ।		१
१.४.६ सूची निरीक्षण	१.४.६	सूची निरीक्षण वर्षमा एकपटक गरिन्छ।		१
मापदण्ड १.४		कुल अङ्क		९
		प्रतिशत = कुल अङ्क / ९ x १००		

क्षेत्र	कोड	प्रमाणीकरण		
मेडिकल रेकर्ड र सूचना व्यवस्थापन	१.५			
उपक्षेत्र उपक्षेत्र		मापदण्डहरु	प्राप्ताङ्क	उच्चतम अङ्क
१.५.१ मेडिकल रेकर्ड व्यवस्थापन	१.५.१.१	रजिस्टर प्रयोग गरेर सेवाग्राहीको नाम दर्ता गरिन्छ।		१
	१.५.१.२	प्रेषण रेकर्डहरु HMIS फाराममा र रजिस्टरमा राख्ने गरिन्छ।		१
	१.५.१.३	FCHV हरुको रेकर्ड र प्रतिवेदनलाई जम्मा गरिन्छ र मासिक रुपमा अध्यावधिक गरिन्छ।		१
१.५.२ सूचना व्यवस्थापन	१.५.२.१	मासिक प्रतिवेदनलाई (HMIS) स्थानीय सरकारको माध्यमबाट केन्द्रिय डाटावेसमा समावेश गरिन्छ।		१
	१.५.२.२	सेवा सदुपयोग तथ्याङ्कलाई चौमासिक रुपमा मूल्याङ्कन गरिन्छ र कर्मचारीहरु बीचमा छलफल गरिन्छ।		१
	१.५.२.३	स्वास्थ्य चौकी र यसको आउटरीच सेवाको सेवा सदुपयोगका प्रमुख तथ्याङ्कहरुलाई लक्षित तथ्याङ्कसँग तुलना गर्दै सार्वजनिक गरिएको छ।		१
१.५.३ सूचना व्यवस्थापनको स्रोतव्यक्ति	१.५.३.१	मेडिकल रिपोर्ट राख्न र त्यसको प्रतिवेदन बनाउन कर्मचारी तोकिएको छ र उक्त रिपोर्टको प्रमाणीकरण स्वास्थ्य चौकी प्रमुखले गरेको छ।		१
	१.५.३.२	स्वास्थ्य चौकी प्रमुखले सेवाग्राही \बिरामी \ बिरामीका आफन्त \ संचारकर्मी \ अन्य सरोकारवालाहरुसँग समन्वय गरेका छन्।		१
मापदण्ड १.५		कुल अङ्क		८
		प्रतिशत = कुल अङ्क / ८ x १००		

क्षेत्र	कोड	प्रमाणीकरण		
गुणस्तर व्यवस्थापन	१.६			
उपक्षेत्र उपक्षेत्र		मापदण्डहरु	प्राप्ताङ्क	उच्चतम अङ्क
१.४.१ वार्षिक कार्ययोजनाको निर्माण र स्विकृति	१.४.१.१	सरकारी अनुदान र कार्यक्रमको अनुदानबाट भएको आम्दानीअनुसार एउटा वार्षिक कार्ययोजनाको निर्माण गरिन्छ ।		१
१.६.२ सामाजिक परिक्षणको प्रतिवेदनका समस्याहरुको संबोधन	१.६.२	सामाजिक परिक्षणबाट उपलब्ध परिणाम जस्तै सेवाग्राहीको अनुभवसम्बन्धीको अन्तर्वाता लाई कर्मचारीको बैठकमा प्रस्तुत गरिन्छ ।		१
१.६.३ गुणस्तर मापन	१.६.३	MSS प्रकृयालाई स्वास्थ्य चौकीले कम्तिमा ४ महिनामा एक पटक प्रयोग गर्छ ।		१
१.६.४ गुणस्तर वृद्धिको योजना	१.६.४	स्वास्थ्य चौकीले MSS मूल्याङ्कनको आधारमा गुणस्तर वृद्धिको लागि विशिस्ट योजना निर्माण गर्छ ।		१
१.६.५ स्वास्थ्य चौकीद्वारा गुणस्तर निर्धारण समितिको सुचकको प्रयोग	१.६.५	स्वास्थ्य चौकीद्वारा सरकारका प्रमुख प्राथमिकताका कार्यक्रमको लागि गुणस्तर निर्धारण समितिले सुचकको प्रयोग गर्छ । (५०% भन्दा कम—०, ५०-७०%--१, ७०-८५%--२, ८५-१००—३)		३
१.६.६ गुणस्तर निर्धारण समिति योजनाको कार्यान्वयन	१.६.६.१	स्वास्थ्य चौकीले MSS योजनाअनुसार निर्दिस्ट कार्यक्रमहरु कार्यान्वयन गरेको छ ।		१
	१.६.६.२	स्वास्थ्य चौकीले गुणस्तर निर्धारण समिति सुचकको दूरी विश्लेशणअनुसार निर्दिष्ट कार्यक्रमहरु कार्यान्वयन गरेको छ ।		१
मापदण्ड १.६		कुल अङ्क		१०
		प्रतिशत = कुल अङ्क / १० x १००		

खण्ड २: क्लिनिकल सेवा व्यवस्थापन

भाग १ का मापदण्डहरुको संख्या र प्राप्ताङ्कको सारांश

क्षेत्र	कुल मापदण्ड संख्या	कुल अङ्क	कुल प्राप्ताङ्क (प्रतिशतमा)
बहिरङ्ग सेबा (ओ. पि. डि)	३९	५७	
खोप र वृद्धि मुल्याङ्कन	१६	१८	
परिवार नियोजन सेवा	१८	२०	
ANC र PNC सेवा	१९	२१	
DOTS क्लिनिक[4]	२२	२२	
औषधी सेवा (डिस्पेन्सरी)	२३ (२४*)	२३ (२४*)	
ड्रेसिङ्ग, ईन्जेक्शन र अन्य प्रक्रियाहरु	१५	२१	
प्रयोगशाला सेवा	२२	२६	
बर्थिङ्ग केन्द्र**	३५**	४१**	
कुल	१७५ (२०५**)	२०९ (२५०**)	

* औलो प्रभावित क्षेत्रमा मात्र (Applicable only for Malaria endemic areas)
** प्रसूतिसेवा सहितको स्वास्थ्य चौकीमा मात्र (Applicable only for health posts with birthing center)

क्षेत्र	कोड	प्रमाणीकरण		
बहिरङ्ग सेवा (ओ.पि.डि)	२.१			
उपक्षेत्र उपक्षेत्र		मापदण्डहरु	प्राप्ताङ्क	उच्चतम अङ्क
२.१.१ बिरामीको लागि समय	२.१.१.१	OPD बिहान १० बजेबाट बेलुका ४ बजेसम्म हुनेछ।		१
	२.१.१.२	स्वास्थ्य चौकीमा आकस्मिक सेवाहरु बिहान १० बजेबाट बेलुका ५ बजेसम्म उपलब्ध हुन्छ।		१
२.१.२ पर्याप्त कर्मचारी	२.१.२.१	OPD सेवाको लागि मध्यम तहका स्वास्थ्यकर्मीहरु परिचालन गरिनेछ।		१
२.१.३ रामीको गोपनीयता	२.१.३	अलग कोठाहरुको व्यवस्था, पर्दाहरु, पालो पर्खने व्यवस्था गरेर बिरामीको गोपनीयता राखिन्छ।		१

4 DOTS= Direct Observed Treatment Short-course

२.१.४ बिरामीको परामर्श	२.१.४.१	बिरामीहरुले कस्तो खालको उपचार पाईराखेका छन् र त्यसको परिणाम के हुन्छ भन्ने बारेमा बिरामीलाई परामर्श दिईन्छ ।		१
	२.१.४.२	OPD प्रतीक्षा कक्षमा IEC कर्नरको व्यवस्था गरेर उचित IEC सामाग्री (पोस्टर, आदि) उपलब्ध गराइन्छ ।		१
२.१.५ भौतिक सुविधाहरु	२.१.५.१	OPD सेवाको लागि कम्तिमा एउटा कोठा जसमा स्वास्थ्यकर्मी र बिरामीको लागि पर्याप्त ठाँऊको व्यवस्था छ ।		१
	२.१.५.२	पर्याप्त उज्यालो र हावा खेल्ने ठाँऊ भएको कोठाको व्यवस्था छ ।		१
	२.१.५.३.१	आवश्यक फर्निचर र साधनहरुको व्यवस्था गरिएको छ । (हेर्नु परिशिस्ट २.१ क OPD सेवाको लागि फर्निचर र साधनहरु)		३
२.१.६ औजार, साधन र उपकरण	२.१.६	OPD का कामहरुको लागि आवश्यक औजार, साधन र उपकरणहरु उपलब्ध छन् र प्रयोग योग्यछन् । (हेर्नु परिशिष्ट २.१ख OPD का कामहरुको लागि आवश्यक औजार, साधन र उपकरणहरु)		३
२.१.७ STP को उपलब्धता र प्रयोग	२.१.७	OPD मा स्तरिय उपचार प्रोटोकल(STP) उपलब्ध छ र प्रयोगमा पनि छ ।		१
२.१.८ ड्युटी तालिका	२.१.८	कर्मचारीहरुको ड्युटी तालिका हप्तैपिच्छे बनाईन्छ र उपयुक्त ठाऊँमा टासिन्छ ।		१
२.१.९ सामान्य सरुवा रोगको पहिचान, उपचार र प्रेषण	२.१.९.१	सामान्य सरुवा रोगको पहिचान, उपचार र आवश्यकताअनुसार प्रेषण गर्नको लागि कम्तिमा एउटा कर्मचारी तोकिएको छ र उसलाई त्यस सम्बन्धी तालिम दिईएको छ ।		१
	२.१.९.२	स्वास्थ्य चौकीमा पहिचान, उपचार र प्रेषण गरिएका सामान्य सरुवा रोगहरुको न्यूनतम सूची रहेको छ । (हेर्नु परिशिष्ट २.१ग सामान्य सरुवा रोगहरुको सूची)		३

२.१.१० सामान्य प्रजननसम्बन्धी रोगको पहिचान, उपचार र प्रेषण	२.१.१०.१	सामान्य प्रजननसम्बन्धी रोगको पहिचान, उपचार र आवश्यकताअनुसार प्रेषण गर्नको लागि कम्तिमा एउटा कर्मचारी तोकिएको छ र उसलाई त्यससम्बन्धी तालिम दिईएको छ ।		१
	२.१.१०.२	स्वास्थ्य चौकीमा पहिचान, उपचार र प्रेषण गरिएका सामान्य प्रजननसम्बन्धी रोगहरुको न्यूनतम सूची रहेको छ । (हेर्नु परिशिष्ट २.१घ सामान्य प्रजननसम्बन्धी रोगहरुको न्यूनतम सूची)		३
२.१.११ यौनजन्य हिंसाको पहिचान, विश्लेशण र व्यवस्थापन	२.१.११.५.१	यौनजन्य हिंसाको पहिचान, विश्लेशण र व्यवस्थापन गर्नको लागि कम्तिमा एउटा कर्मचारी तोकिएको छ र उसलाई त्यससम्बन्धी तालिम दिईएको छ ।		१
२.१.१२ NCD रोगको पहिचान, उपचार र प्रेषण	२.१.१२.१	नसर्ने रोग (NCD) को पहिचान, उपचार र आवश्यकताअनुसार प्रेषण गर्नको लागि कम्तिमा एउटा कर्मचारी तोकिएको छ र उसलाई त्यससम्बन्धी तालिम दिईएको छ । (PEN[5] प्रोटोकोल)		१
	२.१.१२.२	स्वास्थ्य चौकीमा पहिचान, उपचार र प्रेषण गरिएका सामान्य नसर्ने रोगहरुको न्यूनतम सूची रहेको छ । (हेर्नु परिशिष्ट २.१ङ सामान्य नसर्ने रोगहरुको न्यूनतम सूची)		३
२.१.१३ नवजात शिशु र बालबालिकाको रुग्णता, रोगको पहिचान, उपचार र प्रेषण	२.१.१३.१	नवजात शिशु र बालबालिकाको रुग्णता, रोगको पहिचान, उपचार र प्रेषण गर्नको लागि कम्तिमा एउटा कर्मचारी तोकिएको छ र उसलाई त्यससम्बन्धी तालिम दिईएको छ ।		१
	२.१.१३.२	IMNCI निर्देशनअनुसार विरामी नवजात शिशु, बालबालिका र असामयिक शिशुहरुको विश्लेशण, वर्गीकरण, उपचार र प्रेशण गरिन्छ ।		१

5 PEN= Package for Essential Non-communicable Disease

२.१.१४ नाक, कान, घाँटी , मुख र आँखासम्बन्धीका रोगहरुको पहिचान, उपचार र प्रेषण	२.१.१४.१	नाक, कान, घाँटी , मुख र आँखासम्बन्धीका रोगहरुको पहिचान, उपचार र प्रेषण गर्नको लागि कम्तिमा एउटा कर्मचारी तोकिएको छ र उसलाई त्यससम्बन्धी तालिम दिईएको छ ।		१
	२.१.१४.२	स्वास्थ्य चौकीमा पहिचान, उपचार र प्रेषण गरिएका सामान्य नाक, कान, घाँटी , मुख र आँखासम्बन्धीका रोगहरुको न्यूनतम सूची रहेको छ । (हेर्नु परिशिष्ट २.१च सामान्य नाक, कान, घाँटी , मुख र आँखासम्बन्धीका रोगहरुको न्यूनतम सूची)		३
२.१.१५ मानसिक स्वास्थ्यसम्बन्धीका समस्याहरुको पहिचान, उपचार र प्रेषण	२.१.१५.१	मानसिक स्वास्थ्यसम्बन्धीका समस्याहरुको पहिचान, उपचार र प्रेषण गर्नको लागि कम्तिमा एउटा कर्मचारी तोकिएको छ र उसलाई त्यससम्बन्धी तालिम दिईएको छ ।		१
	२.१.१५.२	स्वास्थ्य चौकीमा पहिचान, उपचार र प्रेषण गरिएका सामान्य मानसिक स्वास्थ्यसम्बन्धीका समस्याहरुको न्यूनतम सूची रहेको छ । (हेर्नु परिशिष्ट २.१छ सामान्य मानसिक स्वास्थ्यसम्बन्धीका समस्याहरुको न्यूनतम सूची)		३
२.१.१६ चिरफारसम्बन्धीका समस्याहरुको पहिचान, विश्लेशण, उपचार र प्रेषण	२.१.१६.१	चिरफारसम्बन्धीका समस्याहरुको पहिचान, विश्लेशण, उपचार र आवश्यकताअनुसार प्रेषण गर्नको लागि कम्तिमा एउटा कर्मचारी तोकिएको छ र उसलाई त्यससम्बन्धी तालिम दिईएको छ ।		१
	२.१.१६.२	स्वास्थ्य चौकीमा पहिचान, उपचार र प्रेषण गरिएका सामान्य चिरफारसम्बन्धीका समस्याहरुको न्यूनतम सूची रहेको छ । (हेर्नु परिशिष्ट २.१ज सामान्य चिरफारसम्बन्धीका समस्याहरुको न्यूनतम सूची)		३

२.१.१७ आकस्मिक समस्या र रोगहरुको पहिचान, स्थिरीकरण र प्रेषण	२.१.१७.१	स्वास्थ्य कर्मचारीहरु CPR (कार्डियो पल्मोनरी रिससिटेसन) गर्न सक्छन्।		१
	२.१.१७.२	स्वास्थ्य चौकीमा पहिचान, स्थिरीकरण, उपचार र प्रेषण गर्ने सामान्य आकस्मिक समस्या र रोगहरुको न्यूनतम सूची रहेको छ। (हेर्नु परिशिष्ट २.१झ पहिचान, स्थिरीकरण, उपचार र प्रेषण गर्ने सामान्य आकस्मिक समस्या र रोगहरुको न्यूनतम सूची)		३
२.१.१८ बिरामीको लागि सुविधा	२.१.१८.१	कम्तिमा ५-१० जनाको लागि प्रतीक्षा गर्ने र दर्ता गर्ने बस्ने व्यवस्था सहितको ठाउँ उपलब्ध छ।		१
	२.१.१८.२	प्रतीक्षा गर्ने र दर्ता गर्ने ठाउँमा शुद्ध खानेपानीको व्यवस्था छ।		१
	२.१.१८.३	हात धुने ठाउँ सहितको ३ वटा चर्पीको व्यवस्था छ। (एउटा पुरूषको लागि, एउटा महिलाको लागि र एउटा शारीरिक क्षमता फरक भएका)		१
	२.१.१८.४	बिरामीहरुको लागि हात धुने ठाउँको व्यवस्था छ।		१
२.१.१९ रेकर्डिङ्ग र रिपोर्टिङ्ग (HMIS 1.3 or appropriate tool to be used)	२.१.१९	मापदण्ड फारम र अनूसूचिहरुमा ICD-१० को वर्गीकरण अनुसार निदानहरु रेकर्ड गरिन्छ र OPD का कार्यहरु OPD रजिस्टरमा रेकर्ड गरिन्छ।		१
२.१.२० संक्रमण रोकथाम	२.१.२०.१	मास्क र पञ्जा उपलब्ध छन् र तिनको प्रयोग गरिन्छ।		१
	२.१.२०.२	कम्तिमा पनि तीन वटा फरक रङ्का फोहोर जम्मा गर्ने बाल्टीहरु (एक-एकवटा रातो, निलो र हरियो- HCWM सन् २०१४ को निर्देशिका (स्वास्थ्य तथा जनसंख्या मन्त्रालय) अनुसार उपलब्ध छन् र प्रयोगमा छन्।		१
	२.१.२०.३	स्वास्थ्यकर्मीको लागि सफा पानी र साबुन वा स्यानिटाईजरले हात धुने व्यवस्था छ।		१
	२.१.२०.४	निडिल कटर को प्रयोग गरिन्छ।		१
	२.१.२०.५	विशुद्दिकरण गर्नको लागि क्लोरिनको झोल बनाईन्छ र प्रयोग गरिन्छ।		१
मापदण्ड २.१.१		कुल अङ्क		५७
		प्रतिशत = कुल अङ्क / ५७ x १००		

परिशिस्ट २.१ क OPD सेवाको लागि फर्निचर र साधनहरु

क्रमाङ्क	कर्मचारी दरबन्दी	आवश्यक संख्या	स्कोर	Maximum स्कोर
१	काम गर्ने टेबल	१		१
२	कुर्सी\टुल	३		१
३	कुरुवाको लागि बेन्च\कुर्सी	कम्तिमा ५ जनाको लागि		१
४	परिक्षण बेड र डसना	१		१
५	सिरानी	१		
६	बेडमा चढ्ने सिढीँ	१		
७	तन्ना	२		
८	म्याकिन्टोश	२		
९	पर्दा	१		
१०	दराज	१		
			कुल स्कोर	
			कुल प्रतिशत = कुल स्कोर/ ४ x१००	

हरेक हरफले १ अङ्क पाउनेछ यदि सबै आवश्यक संख्या छ भने अन्यथा ०

स्कोरिङ्ग तालिका	
कुल प्रतिशत	स्कोर
०-४९	०
५०-६९	१
७०-८४	२
८५-१००	३
मापदण्डको स्कोर २.१.५.३.१	

परिशिष्ट २.१ख ओ.पि.डि सेवाको लागि उपकरण र औजारहरु

क्रमाङ्क	कर्मचारी दरबन्दी	आवश्यक संख्या	स्कोर	Maximum स्कोर
१	स्टेथेस्कोप	१		१
२	प्रेसर मापन यन्त्र (मर्करी मुक्त)	१		१
३	डिजिटल थर्मोमिटर (मर्करी मुक्त)	१		१
४	नि जर्क ह्याम्मर	१		१
५	ENT सेट	१		
६	टर्च लाइट\फ्ल्यासलाइट	१		
७	स्टप वाच\टाइमर	१		
८	ट्युनिङ्ग फर्क	१		
९	डक्स इस्पेकुलम	१		

१०	प्रोक्टोस्कोप	१		
११	हात धुने स्यानिटाइजर	१		
१२	जाँच पञ्जा	१ बाकस		
१३	निर्मलीकृत जाँच पञ्जा	२ प्रत्येक फरक साईजका		
१४	परिक्षण गर्ने वत्ती	१		
१५	डिस्पोजेबल काठको टङ्ग डिप्रेसर	आवश्यकतानुसार		
१६	उच्च क्षेमता मास्क	आवश्यकतानुसार		
१७	तौलने स्केल- वयस्क	१		
१८	तौलने स्केल- नवजात शिशु	१		
१९	तौलने स्केल- ५ वर्ष मुनिका बच्चा (Salter or Secca scale)	१		
२०	लंवाई\ऊचाई बोर्ड	१		
२१	स्नेलेन चार्ट	१		
२२	MUAC टेप	३		
२३	खकार जम्मा गर्ने भाँडो	आवश्यकतानुसार		
२४	खकारको नमूनाको लागी स्लाइड	आवश्यकतानुसार		
२५	खकारको नमूना राख्ने सिसाको निर्मलीकृत बट्टा	आवश्यकतानुसार		
२६	ल्याब रेकर्ड फाराम	आवश्यकतानुसार		
२७	क्लिनिकल रेकर्ड फाराम	आवश्यकतानुसार		
			कुल स्कोर	
			कुल प्रतिशत = कुल स्कोर/ ४ x१००	

हरेक हरफले १ अङ्क पाउनेछ यदि सबै आवश्यक संख्या छ भने अन्यथा ०

स्कोरिङ्ग तालिका	
कुल प्रतिशत	स्कोर
०-४९	०
५०-६९	१
७०-८४	२
८५-१००	३
मापदण्डको स्कोर २.१.६	

परिशिष्ट २.१ ग सरुवा रोगहरु तथा संक्रमणको सूची

क्रमाङ्क	सरुवा रोगहरुको सूची पहिचान गरिएको,निदान गरिएको र प्रेषण गरिएको	स्कोर
१	कुष्टरोग	
२	औंलो	
३	शिघ्र पखाला	
४	आँऊ र अन्य प्रोटोजुवल रोगहरु	
५	टाइफाइड र प्याराटाइफाइड	
६	प्यारासाइटिक संक्रमण	
७	कालाअजार	
८	फिलारियासिस	
९	लुतो	
१०	जुम्रा	
११	ढुसीको संक्रमण	
१२	हाडे	
१३	दादुरा	
१४	ठेऊला	
१५	रुबेला	
१६	सिजनल रुघाखोकी	
१७	पिनास	
१८	फ्यारिन्जाइटिस	
१९	टन्सिलाइटिस	
२०	ब्रोन्काइटिस	
२१	निमोनिया	
	कुल अङ्क	
	कुल प्रतिशत= कुल अङ्क/२१ x १००	

हरेक हरफले १ अङ्क पाउनेछ यदि सबै आवश्यक संख्या छ भने अन्यथा ०

स्कोरिङ्ग तालिका	
कुल प्रतिशत	स्कोर
०-४९	०
५०-६९	१
७०-८४	२
८५-१००	३
मापदण्डको स्कोर २.१.९.२	

परिशिष्ट २.१ घ प्रजनन स्वास्थ्य सम्वन्धि रोगहरुको सूची

क्रमाङ्क	रोगहरुको सूची पहिचान गरिएको,निदान गरिएको र प्रेषण गरिएको	स्कोर
१	एच्.आई. भी\एड्स	
२	भिरिङ्गी	
३	अप्थालमिया नियोन्याटोरम	
४	यूरेथ्रल डिस्चार्ज सिन्ड्रोम	
५	आङ खस्नु (Grade I, GradeII, Grade III)	
६	भजाइनल डिस्चार्ज सिन्ड्रोम	
७	जेनाइटल अल्सर सिन्ड्रोम	
८	तल्लो पेट दुख्ने सिन्ड्रोम	
९	स्क्रोटल सुन्निने सिन्ड्रोम	
१०	ईन्गुइनल बुबो सिन्ड्रोम	
११	प्रोल्याप्स सिन्ड्रोम (Grade IV)	
१२	अव्स्ट्रेटिक फिस्टुला	
१३	सर्भाइकल क्यान्सर	
१४	सुरक्षित गर्भपतन सेवा (१२ हप्तासम्मको)	
	कुल अङ्क	
	कुल प्रतिशत= कुल अङ्क/२१ x १००	

हरेक हरफले १ अङ्क पाउनेछ यदि सबै आवश्यक संख्या छ भने अन्यथा ०

स्कोरिङ्ग तालिका	
कुल प्रतिशत	स्कोर
०-४९	०
५०-६९	१
७०-८४	२
८५-१००	३
मापदण्डको स्कोर २.१.१०.२	

परिशिष्ट २.१ ङ नसर्ने रोगहरुको सूची

क्रमाङ्क	नसर्ने रोगहरुको न्यूनतम सूची पहिचान गरिएको,निदान गरिएको र प्रेषण गरिएको	स्कोर
१	मुटुरोग (उच्च रक्तचाप, हृदयघात)	
२	मष्तिस्क पक्षघात	
३	मधुमेह	
४	दम	
५	पाठेघरको मुखको क्यान्सर	
	कुल अङ्क	
	कुल प्रतिशत= कुल अङ्क/२१ x १००	

हरेक हरफले १ अङ्क पाउनेछ यदि सबै आवश्यक संख्या छ भने अन्यथा ०

स्कोरिङ्ग तालिका	
कुल प्रतिशत	स्कोर
०-४९	०
५०-६९	१
७०-८४	२
८५-१००	३
मापदण्डको स्कोर २.१.१२.२	

परिशिष्ट २.१ च नाक, कान, घाँटी, मुख तथा आँखाका रोगहरुको सूची

क्रमाङ्क	नाक, कान, घाँटी, मुख तथा आँखाका रोगहरुको सूची पहिचान गरिएको,निदान गरिएको र प्रेषण गरिएको	स्कोर
१	फूरनकुलोसिस	
२	ओटाइटिस इक्सटर्ना	
३	ओटोमाइकोसिस	
४	एक्यूट ओटाइटिस मिडिया	
५	बहिरोपना	
६	भेस्टिबुलाईटिस	
७	कानेगुजी	
८	गिजा सुनिन्नेे	
९	ग्लोसाइटिस	
१०	मुखको अल्सर	
११	स्टोमाटाइटिस	
१२	पिलो	
१३	दाँतमा किरा लाग्ने	
१४	कन्जङ्टिभाइटिस	

१५	आनो	
१६	ब्लेफराइटिस	
	कुल अङ्क	
	कुल प्रतिशत= कुल अङ्क/२१ x १००	

हरेक हरफले १ अङ्क पाउनेछ यदि सबै आवश्यक संख्या छ भने अन्यथा ०

स्कोरिङ्ग तालिका	
कुल प्रतिशत	स्कोर
०-४९	०
५०-६९	१
७०-८४	२
८५-१००	३
मापदण्डको स्कोर २.१.१४.२	

परिशिष्ट २.१ छ सामान्य मानसिक रोगहरुको सूची

क्रमाङ्क	सामान्य मानसिक रोगहरुको सूची पहिचान गरिएको,निदान गरिएको र प्रेषण गरिएको (BHS प्याकेजअनुसार)	स्कोर
१	डिप्रेशन	
२	एङ्जाइटि	
३	साइकोसिस(फलो अप र पूर्ति औषधिहरु)	
४	लागूपदार्थ दूर्व्यसन	
५	जड्याहापना	
६	छारेरोग (फलो अप र पूर्ति औषधिहरु)	
	कुल अङ्क	
	कुल प्रतिशत= कुल अङ्क/६x १००	

हरेक हरफले १ अङ्क पाउनेछ यदि सबै आवश्यक संख्या छ भने अन्यथा ०

स्कोरिङ्ग तालिका	
कुल प्रतिशत	स्कोर
०-४९	०
५०-६९	१
७०-८४	२
८५-१००	३
मापदण्डको स्कोर २.१.१५.२	

परिशिष्ट २.१ ज सामान्य चिरफारसम्बन्धीका रोगहरुको सूची

क्रमाङ्क	सामान्य चिरफारसम्बन्धीका रोगहरुको सूची पहिचान गरिएको र प्रेषण गरिएको (BHS प्याकेजअनुसार)	स्कोर
१	हर्निया	
२	हाइड्रोसिल	
३	सेलुलाइटिस	
४	पिलो	
	कुल अङ्क	
	कुल प्रतिशत= कुल अङ्क/२१ x १००	

हरेक हरफले १ अङ्क पाउनेछ यदि सबै आवश्यक संख्या छ भने अन्यथा ०

स्कोरिङ्ग तालिका	
कुल प्रतिशत	स्कोर
०-४९	०
५०-६९	१
७०-८४	२
८५-१००	३
मापदण्डको स्कोर २.१.१२.२	

परिशिष्ट २.१ झ सामान्यतया भेटिने आकस्मिक अवस्थाहरु

क्रमाङ्क	नाक, कान, घाँटी, मुख तथा आँखाका रोगहरुको सूची पहिचान गरिएको,निदान गरिएको र प्रेषण गरिएको	स्कोर
१	शीघ्र दुखाई	
२	बेहोस	
३	काम्रे रोग	
४	सडक दुर्घटना	
५	फ्रयाक्चर, जोर्नी फुत्कने\खस्कने	
६	जटिलता रहित पहिलो चरणको पोलाई	
७	विष सेवन	
८	पानीमा डुब्नु	
९	फरेन बडी	
१०	नाकवाट रगत बग्नु	
	कुल अङ्क	
	कुल प्रतिशत= कुल अङ्क/२१ x १००	

स्कोरिङ्ग तालिका	
कुल प्रतिशत	स्कोर
०-४९	०
५०-६९	१
७०-८४	२
८५-१००	३
मापदण्डको स्कोर २.१.१७.२	

क्षेत्र	कोड	प्रमाणीकरण		
खोप र वृद्धि अनुगमन	२.२.१			
उपक्षेत्र उपक्षेत्र		मापदण्डहरु	प्राप्ताङ्क	उच्चतम अङ्क
२.२.१.१ बिरामीको लागि समय	२.२.१.१	खोप र वृद्धि अनुगमन सेवा प्रत्येक कार्यदिनको विहान १० बजेदेखि बेलुका ४ बजेसम्म हुनेछ।		१
२.२.१.२ कर्मचारी परिचालन	२.२.१.२	कर्मचारीहरुमध्ये एक कर्मचारी खोप र वृद्धि अनुगमन सेवामा खटिनेछ।		१
२.२.१.३ बिरामीको गोपनीयता	२.२.१.३	बिरामीको गोपनीयताको लागि उपयुक्त उपायहरु अपनाईन्छ (फरक कोठा, पर्दाको प्रयोग, पंक्तिबद्दता)।		१
२.२.१.४ बिरामीलाई परामर्श	२.२.१.४.१	बिरामीको आफन्तलाई खोपको प्रकार, खोप दिने तरिका, बच्चाको पोषणको अवस्थाको बारेमा परामर्श दिइन्छ।		१
	२.२.१.४.२	खोपको प्रकार, खोप दिने तरिका, बच्चाको पोषणको बारेका उचित IEC सामाग्रीहरु (पोस्टर, आदि) खोप र वृद्धि अनुगमन सेवा दिइने ठाऊँमा उपलब्ध गराइन्छ।		१
२.२.१.५ औजार, साधन र उपकरणको उपलब्धता	२.२.१.५	खोप र वृद्धि अनुगमनसम्बन्धीका औजार, साधन र उपकरणहरु उपलब्ध छन्। (हेर्नु परिशिष्ट २.२.१क खोप र वृद्धिवृद्धि अनुगमनसम्बन्धीका औजार, साधन र उपकरणहरु)		३
२.२.१.६ STP को उपलब्धता र प्रयोग	२.२.१.६	खोप सेवाको लागि मापदण्ड उपचार प्रोटोकल(STP) उपलब्ध छ र प्रयोगमा पनि छ।		१

२.२.१.७ भौतिक सुविधा	२.२.१.७.१	स्वास्थ्यकर्मी र बिरामीको लागि कम्तिमा एउटा टेबल, स्वास्थ्यकर्मीको लागि कुर्सी र बिरामीको लागि दुईवटा कुर्सीसहितको पर्याप्त ठाउँ छ ।		१
	२.२.१.७.२	पर्याप्त उज्यालो र हावा खेल्ने ठाँऊ भएको कोठाको व्यवस्था छ ।		१
२.२.१.८ रेकर्डिङ्ग र रिपोर्टिङ्ग	२.२.१.८.१	बिरामी कार्ड (स्वास्थ्य कार्ड, वृद्धि तालिका) र खोप रजिस्टर उपलब्ध छन् र तिनमा सेवाहरु रेकर्ड गरिएको छ ।		१
	२.२.१.८.२	खोपसम्बन्धीका हानिहरु, जटिलता र अति शीघ्र कुपोषण र अन्यत्र प्रेषणहरु सबै रेकर्ड गरिएको छ र तिनको रिपोर्ट गरिएको छ ।		१
२.२.१.९ संक्रमण रोकथाम	२.२.१.९.१	मास्क र पञ्जा उपलब्ध छन् र तिनको प्रयोग गरिन्छ ।		१
	२.२.१.९.२	कम्तिमा पनि तीन वटा फरक रङका फोहोर जम्मा गर्ने बाल्टीहरु (एक-एकवटा रातो, निलो र हरियो- HCWM सन् २०१४ को निर्देशिका (स्वास्थ्य तथा जनसंख्या मन्त्रालय) अनुसार) उपलब्ध छन् र प्रयोगमा छन् ।		१
	२.२.१.९.३	स्वास्थ्यकर्मीको लागि सफा पानी र साबुन वा स्यानिटाईजरले हात धुने व्यवस्था छ ।		१
	२.२.१.९.४	निडिल कटर र सेफ्टी बाकसको प्रयोग गरिन्छ ।		१
	२.२.१.९.५	विशुद्दिकरण गर्नको लागि क्लोरिनको झोल बनाईन्छ र प्रयोग गरिन्छ ।		१
मापदण्ड २.२.१		कुल प्राप्ताङ्क		१८
		कुल प्रतिशत (कुल प्राप्ताङ्क / १८ x१००)		

परिशिष्ट २.२.१क खोप र वृद्धि अनुगमनसम्बनका औजार, साधन र उपकरणहरु

क्रमाङ्क	नाम	आवश्यक संख्या	स्कोर
१	जोख्ने यन्त्र (इन्फ्यान्टोमिटर र सेक्का स्केल) तौल मेसिन	कम्तिमा एउटा प्रत्येकमा	
२	स्टेडियोमिटर (उचाइ नाप्ने यन्त्र)	कम्तिमा एउटा	
३	MUAC टेप	२	
४	कोल्ड चेन बाकस\खोप बोक्ने आइस प्याकसहितको सेट	कम्तिमा एउटा set	
५	खोपको लागि विभिन्न साईजका सूईहरु (१,२,३,५,१० मि.लि)	कम्तिमा १० प्रत्येकमा	
६	कटन स्वाब राख्ने भाँडो	आवश्यकतानुसार	
७	सफा पानि राख्ने भाँडो	आवश्यकतानुसार	
राष्ट्रिय खोप कार्यक्रमअनुसारका खोपहरु वर्षभरि उपलब्ध रहेको			
८	Bacillus Calmatte Guerin (BCG)	वर्षभरि नै स्टक नसकिएको	
९	Pentavalent (DPT, HiB, Hep B), FIPV	वर्षभरि नै स्टक नसकिएको	
१०	Oral polio vaccine	वर्षभरि नै स्टक नसकिएको	
११	Pneumococcal conjugated vaccine	वर्षभरि नै स्टक नसकिएको	
१२	Measles/ rubella	वर्षभरि नै स्टक नसकिएको	
१३	Japanese encephalitis	वर्षभरि नै स्टक नसकिएको	
१४	Vitamin A	वर्षभरि नै स्टक नसकिएको	
		कुल अङ्क	
		प्रतिशत = कुल अङ्क/ १४ x १००	

हरेक हरफले १ अङ्क पाउनेछ यदि सबै आवश्यक संख्या छ भने अन्यथा ०

स्कोरिङ्ग तालिका	
कुल प्रतिशत	स्कोर
०-४९	०
५०-६९	१
७०-८४	२
८५-१००	३
मापदण्डको स्कोर २.२.१.५	

क्षेत्र	कोड	प्रमाणीकरण		
परिवार नियोजन सेवा	२.२.२			
उपक्षेत्र उपक्षेत्र		मापदण्डहरु	प्राप्ताङ्क	उच्चतम अङ्क
२.२.२.१ बिरामीको लागि समय	२.२.२.१	परिवार नियोजन सेवा प्रत्येक कार्यदिनको विहान १० बजेदेखि बेलुका ४ बजेसम्म हुनेछ ।		१
२.२.२.२ ठाऊँ	२.२.२.२	परिवार नियोजन सेवा र परामर्शको लागि छुट्टै ठाऊँको व्यवस्था गरिएको छ ।		१
२.२.२.३ कर्मचारी परिचालन	२.२.२.३	कर्मचारीहरुमध्य एक कर्मचारी परिवार नियोजन सेवा र परामर्शको सेवामा खटिनेछ ।		१
२.२.२.४ बिरामीको गोपनीयता	२.२.२.४	बिरामीको गोपनीयताको लागि उपयुक्त उपायहरु अपनाईन्छ (फरक कोठा, पर्दाको प्रयोग, पंक्तिबद्दता) ।		१
२.२.२.५ परामर्श	२.२.२.५.१	परिवार नियोजनका साधनको प्रयोगकर्तालाई परिवार नियोजनका साधनसम्बन्धी परामर्श दिइन्छ ।		१
	२.२.२.५.२	परिवार नियोजनसम्बन्धीका उपयुक्त IEC/BCC सामाग्रीहरु परामर्शमा प्रयोग गरिन्छ ।		१
२.२.२.६ उपलब्ध साधनहरु	२.२.२.६	परिवार नियोजन सेवाको लागि साधनहरु उपलब्ध छन् । (हेर्नु परिशिष्ट २.२.२क परिवार नियोजन सेवाको लागि साधनहरु)		३
२.२.२.७ उपकरण र स्रोतहरु	२.२.२.७	काम गरिरहेको BP मापन यन्त्र, स्तेथोस्कोप, थर्मोमिटर र वजन मापन यन्त्रहरु परिवार नियोजन सेवाको लागि उपलब्ध छन् ।		१
२.२.२.८ स्तरिय उपचार प्रोटोकल(STP) उपलब्धता र प्रयोग	२.२.२.८	परिवार नियोजन सेवाको लागि स्तरिय उपचार प्रोटोकल(STP) उपलब्ध छ र प्रयोगमा पनि छ ।		१
२.२.२.९ भौतिक सुविधा	२.२.२.९.१	स्वास्थ्यकर्मी र बिरामीको लागि कम्तिमा एउटा टेबल, एउटा परिक्षण गर्ने खाट, स्वास्थ्यकर्मीको लागि कुर्सी र बिरामीको लागि दुईवटा कुर्सीसहितको पर्याप्त ठाऊँ छ ।		१
	२.२.२.९.२	पर्याप्त उज्यालो र हावा खेल्ने ठाँऊ भएको कोठाको व्यवस्था छ ।		१

२.२.२.१० रेकर्डिङ्ग र रिपोर्टिङ्ग	२.२.२.१०.१	बिरामी कार्ड \स्वास्थ्य कार्ड र परिवार नियोजन रजिस्टर उपलब्ध छन् र तिनमा सेवाहरु रेकर्ड गरिएको छ ।		१
	२.२.२.१०.२	परिवार नियोजनसम्बन्धीका जटिलता, साधनहरुको असफलता र बिरामीको अनियमितताहरु सबै रेकर्डिङ्ग र रिपोर्टिङ्ग गरिन्छ ।		१
२.२.२.११ संक्रमण रोकथाम	२.२.२.११.१	मास्क र पञ्जा उपलब्ध छन् र तिनको प्रयोग गरिन्छ ।		१
	२.२.२.११.२	कम्तिमा पनि तीन वटा फरक रङ्का फोहोर जम्मा गर्ने बाल्टीहरु (एक-एकवटा रातो, निलो र हरियो- HCWM सन् २०१४ को निर्देशिका (स्वास्थ्य तथा जनसंख्या मन्त्रालय) अनुसार) उपलब्ध छन् र प्रयोगमा छन् ।		१
	२.२.२.११.३	स्वास्थ्यकर्मीको लागि सफा पानी र साबुन वा स्यानिटाईजरले हात धुने व्यवस्था छ ।		१
	२.२.२.११.४	निडिल कटर र सेफ्टी बाकसको प्रयोग गरिन्छ ।		१
	२.२.२.११.५	विशुद्दिकरण गर्नको लागि क्लोरिनको झोल बनाईन्छ र प्रयोग गरिन्छ ।		१
मापदण्ड २.२.२		कुल प्राप्ताङ्क		२०
		कुल प्रतिशत (कुल प्राप्ताङ्क / २० x१००)		

परिशिष्ट २.२.२ क परिवार नियोजनका उपकरण

क्रमाङ्क	नाम	आवश्यक संख्या	स्कोर
१	कण्डम	आवश्यकतानुसार	
२	परिवार नियोजनको चक्की	आवश्यकतानुसार	
३	आइ.यू.डी	आवश्यकतानुसार	
४	आइ.यू.डी राख्ने र निकाल्ने सेट	कम्तिमा २	
५	ईम्प्लान्ट	आवश्यकतानुसार	
६	ईम्प्लान्ट राख्ने र निकाल्ने सेट	कम्तिमा २	
७	डिपो प्रोभेरा सूई	आवश्यकतानुसार	
८	आकस्मिक गर्भनिरोधक चक्की	आवश्यकतानुसार	
९	सर्जिकल ग्लोभ्स (फरक साईजको)	२-३ जोर	
		कुल अङ्क	
		प्रतिशत = कुल अङ्क/ ९ x १००	

हरेक हरफले १ अङ्क पाउनेछ यदि सबै आवश्यक संख्या छ भने अन्यथा ०

स्कोरिङ्ग तालिका	
कुल प्रतिशत	स्कोर
०-४९	०
५०-६९	१
७०-८४	२
८५-१००	३
मापदण्डको स्कोर २.२.२.६	

क्षेत्र	कोड	प्रमाणीकरण		
ANC[6] र PNC[7] सेवाहरु	२.२.३			
उपक्षेत्र उपक्षेत्र		मापदण्डहरु	प्राप्ताङ्क	उच्चतम अङ्क
२.२.३.१ बिरामीको लागि समय	२.२.३.१	ANC र PNC सेवा प्रत्येक कार्यदिनको विहान १० बजेदेखि बेलुका ४ बजेसम्म हुनेछ।		१
	२.२.३.२	इमर्जेन्सी सेवा प्रत्येक कार्यदिनको विहान १० बजेदेखि बेलुका ५ बजेसम्म हुनेछ।		१
२.२.३.२ ठाऊँ	२.२.३.२	ANC and PNC सेवा, जाँच र परामर्शको लागि छुट्टै क्षेत्रको व्यवस्था गरिएको छ।		१
२.२.३.३ कर्मचारी परिचालन	२.२.३.३	कर्मचारीहरुमध्ये एक कर्मचारी ANC and PNC सेवा, जाँच र परामर्श सेवामा खटिनेछ।		१
२.२.३.४ बिरामीको गोपनीयता	२.२.३.४	बिरामीको गोपनीयताको लागि उपयुक्त उपायहरु अपनाईन्छ (फरक कोठा, पर्दाको प्रयोग, पंक्तिबद्दता)।		१
२.२.३.५ बिरामी परामर्श	२.२.३.५.१	महिला र उसको परिवारलाई गर्भवती हुँदा अपनाउनुपर्ने सावधानी, खतराका चिन्हहरु, प्रसूतिको तयारि, प्रसूतिपश्चात अपनाउनुपर्ने सावधानी, स्तनपान र खोपको बारेमा परामर्श दिइन्छ।		१
	२.२.३.५.२	गर्भवती हुँदा अपनाउनुपर्ने सावधानी, खतराका चिन्हहरु, प्रसूतिको तयारि, प्रसूतिपश्चात अपनाउनुपर्ने सावधानी, स्तनपान र खोपको बारेमा उचित IEC सामाग्रीहरु (पोस्टर, आदि) ANC and PNC सेवा, जाँच र परामर्श सेवा दिइने ठाऊँमा उपलब्ध गराइन्छ।		१

6 ANC= Antenatal Checkup (पुर्व प्रशुति जाँच)
7 PNC= Postnatal Checkup (उत्तर प्रशुति जाँच)

	२.२.३.५.३	गर्भावस्थासँगै आउनसक्ने अन्य रोगहरु जस्तै टी.बी, एच.आई.भी एड्स, भिरिङ्गी, हर्पिस, हेपाटाईटिस को बारेमा पनि परामर्श गरिन्छ र प्रेषण गरिन्छ।		१
२.२.३.६ औजार, साधन र उपकरणको उपलब्धता	२.२.३.६	ANC and PNC सेवा, जाँच र परामर्शसम्बन्धीका औजार, साधन र उपकरणहरु उपलब्ध छन्। (हेर्नु परिशिष्ट २.२.३क ANC and PNC सेवा, जाँच र परामर्शसम्बन्धीका औजार, साधन र उपकरणहरु)		३
२.२.३.७ STP उपलब्धता र प्रयोग	२.२.३.७	ANC and PNC सेवा, जाँच र परामर्शको मापदण्ड उपचार प्रोटोकल(STP) उपलब्ध छ र प्रयोगमा पनि छ।		१
२.२.३.८ भौतिक सुविधा	२.२.३.८.१	स्वास्थ्यकर्मी र बिरामीको लागि कम्तिमा एउटा टेबल, स्वास्थ्यकर्मीको लागि कुर्सी र बिरामीको लागि दुईवटा कुर्सीसहितको पर्याप्त ठाउँ छ।		१
	२.२.३.८.२	पर्याप्त उज्यालो र हावा खेल्ने ठाँऊ भएको कोठाको व्यवस्था छ।		१
२.२.३.९ रेकर्डिङ्ग र रिपोर्टिङ्ग	२.२.३.९.१	बिरामी कार्ड (स्वास्थ्य कार्ड, वृद्धि अनुगमन कार्ड) र सोहीअनुसारका रजिस्टर उपलब्ध छन् र तिनमा सेवाहरु रेकर्ड गरिएको छ।		१
	२.२.३.९.२	ANC and PNC सेवा, जाँच र परामर्शको रेकर्डिङ्ग र रिपोर्टिङ्ग गरिन्छ।		१
२.२.३.१० संक्रमण रोकथाम	२.२.३.१०.१	मास्क र पञ्जा उपलब्ध छन् र तिनको प्रयोग गरिन्छ।		१
	२.२.३.१०.२	कम्तिमा पनि तीन वटा फरक रङका फोहोर जम्मा गर्ने बाल्टीहरु (एक-एकवटा रातो, निलो र हरियो- HCWM सन् २०१४ को निर्देशिका (स्वास्थ्य तथा जनसंख्या मन्त्रालय) अनुसार) उपलब्ध छन् र प्रयोगमा छन्।		१
	२.२.३.१०.३	स्वास्थ्यकर्मीको लागि सफा पानी र साबुन वा स्यानिटाईजरले हात धुने व्यवस्था छ।		१
	२.२.३.१०.४	निडिल कटर र सेफ्टी बाकसको प्रयोग गरिन्छ।		१
	२.२.३.१०.५	विशुद्दिकरण गर्नको लागि क्लोरिनको झोल बनाईन्छ र प्रयोग गरिन्छ।		१
मापदण्ड २.२.३			कुल प्राप्ताङ्क	२१
			कुल प्रतिशत (कुल प्राप्ताङ्क / २१ x१००)	

परिशिष्ट २.२.३ क ANC सेवाको लागि सामाग्रीहरु

क्रमाङ्क	नाम	आवश्यक संख्या	स्कोर
१	डसनासहितको परीक्षण बेड	कम्तिमा १	
२	तौल मापन यन्त्र	कम्तिमा १	
३	फुटस्टेप	कम्तिमा १	
४	कुर्सी\टुल	कम्तिमा ३	
५	सिरानी	कम्तिमा १	
६	सिरानीको खोल	कम्तिमा १	
७	तन्ना	कम्तिमा २	
८	म्याकिनटोश	कम्तिमा २	
९	MUAC टेप- वयस्क	कम्तिमा २	
१०	ऊचाई बोर्ड (वयस्क)	कम्तिमा १	
११	फिटोस्कोप	कम्तिमा २	
१२	पाठेघरको ऊचाई नाप्ने टेप	कम्तिमा २	
१३	प्रेसर मापन यन्त्र	कम्तिमा १	
१४	स्टेथोस्कोप	कम्तिमा १	
१५	थर्मोमिटर	कम्तिमा १	
१६	स्पेकुलम	कम्तिमा २	
१७	आईरन\फोलिक एसिड	वर्षभरि नै स्टक नसकिएको	
१८	Td खोप	वर्षभरि नै स्टक नसकिएको	
१९	भिटामिन A	वर्षभरि नै स्टक नसकिएको	
२०	परीक्षण पञ्जा	कम्तिमा १ बाकस	
२१	क्यालेण्डर	कम्तिमा एउटा	
		कुल अङ्क	
		प्रतिशत = कुल अङ्क/ १४ x १००	

हरेक हरफले १ अङ्क पाउनेछ यदि सबै आवश्यक संख्या छ भने अन्यथा ०

स्कोरिङ्ग तालिका	
कुल प्रतिशत	स्कोर
०-४९	०
५०-६९	१
७०-८४	२
८५-१००	३
मापदण्डको स्कोर २.२.२.६	

क्षेत्र	कोड	प्रमाणीकरण		
DOTS[8] क्लिनिक	२.२.४			
उपक्षेत्र उपक्षेत्र		मापदण्डहरु	प्राप्ताङ्क	उच्चतम अङ्क
२.२.४.१ बिरामीको लागि समय	२.२.४.१	DOTS सेवा प्रत्येक कार्यदिनको विहान १० बजेदेखि बेलुका ४ बजेसम्म हुनेछ।		१
२.२.३.२ कर्मचारी परिचालन	२.२.४.२	कर्मचारीहरुमध्ये एक कर्मचारी DOTS सेवामा खटिनेछ।		१
२.२.३.३ बिरामीको गोपनीयता	२.२.४.३	बिरामीको गोपनीयताको लागि उपयुक्त उपायहरु अपनाईन्छ (फरक कोठा, पर्दाको प्रयोग, पंक्तिबद्दता)।		१
२.२.३.४ बिरामी परामर्श	२.२.४.४.१	बिरामीलाई आफुले के उपचार पाएको हो र त्यसको प्रतिफल के हुन्छ भन्ने कुराको परामर्श दिईन्छ।		१
	२.२.४.४.२	टि.बी रHIV/AIDS को सहसंक्रमणबारेका उचित IEC सामाग्रीहरु (पोस्टर, आदि) टि.बीको उपचार गरिने ठाऊँमा उपलब्ध गराइन्छ।		१
	२.२.४.४.३	शंका लागेका बिरामीहरुको रोगको बारेमा सोध्ने र खकारको नमूना लिने गरिएको छ।		१
	२.२.४.४.४	संकलित नमूनालाई यकिन निदान पत्ता लगाउन माथिल्लो स्तरको स्वास्थ्य संस्थामा पठाइन्छ वा बिरामीलाई नै प्रेषण गरिन्छ।		१
२.२.३.५ औषधीको उपलब्धता र प्रेषण	२.२.४.५	नेपाल सरकारको निर्देशिका अनुसार टि.बी का औषधीहरु क्याटगोरी १ र २ गरि खुवाईन्छ।		१
२.२.३.६ स्रोत र साधनको उपलब्धता	२.२.४.६.१	DOTS कर्नर वा कोठामा काम गर्ने (नबिग्रेको)BP मापन यन्त्र, स्टेथेस्कोप, थर्मोमिटर र वजन मापन यन्त्रहरु उपलब्ध छन्।		१
२.२.४.७ STP उपलब्धता र प्रयोग	२.२.४.७	टि.बी उपचार सेवाको लागि स्तरिय उपचार प्रोटोकल(STP) उपलब्ध छ र प्रयोगमा पनि छ।		१

8 DOTS= Direct Observed Treatment Shortcourse

२.२.४.७ भौतिक सुविधा	२.२.४.८.१	स्वास्थ्यकर्मी र बिरामीको लागि कम्तिमा एउटा टेबल, एउटा परिक्षण गर्ने खाट, स्वास्थ्यकर्मीको लागि कुर्सी र बिरामीको लागि दुईवटा कुर्सीसहितको पर्याप्त ठाउँ छ ।		१
	२.२.४.८.२	पर्याप्त उज्यालो र हावा खेल्ने ठाँऊ भएको कोठाको व्यवस्था छ ।		१
२.२.४.८ बिरामीका लागि सुविधा	२.२.४.९.१	औषधी सेवनको लागि मग वा गिलाससहितको सफा पिऊने पानीको व्यवस्था छ ।		१
	२.२.४.९.२	बिरामीको लागि हात धुने सुविधा छ ।		१
२.२.४.९ रेकर्डिङ्ग र रिपोर्टिङ्ग	२.२.४.१०.१	बिरामी कार्ड र रजिस्टरमा सेबाहरु रेकर्ड गरिन्छ ।		१
	२.२.४.१०.२	औषधीको अप्रभाबकारिता, जटिलता, बिरामीको अनियमितता र प्रेषणहरु सबै रेकर्डिङ्ग र रिपोर्टिङ्ग गरिन्छ ।		१
२.२.४.१० संक्रमण रोकथाम	२.२.४.११.१	सबै बहु-औषधी प्रभावहीन भएको टि.बीको शंका लागेका बिरामीहरुलाई एम्.डि. आर. केन्द्रमा प्रेसण गरिन्छ ।		१
	२.२.४.११.२	मास्क र पञ्जा उपलब्ध छन् र तिनको प्रयोग गरिन्छ ।		१
	२.२.४.११.३	कम्तिमा पनि तीन वटा फरक रङका फोहोर जम्मा गर्ने बाल्टीहरु (एक-एकवटा रातो, निलो र हरियो- HCWM सन् २०१४ को निर्देशिका (स्वास्थ्य तथा जनसंख्या मन्त्रालय) अनुसार) उपलब्ध छन् र प्रयोगमा छन् ।		१
	२.२.४.११.४	स्वास्थ्यकर्मीको लागि सफा पानी र साबुन वा स्यानिटाईजरले हात धुने व्यवस्था छ ।		१
	२.२.४.११.५	निडिल कटर र सेफ्टी बाकसको प्रयोग गरिन्छ ।		१
	२.२.४.११.६	विशुद्दिकरण गर्नको लागि क्लोरिनको झोल बनाईन्छ र प्रयोग गरिन्छ ।		१
मापदण्ड २.२.३		कुल प्राप्ताङ्क		
		कुल प्रतिशत (कुल प्राप्ताङ्क / २१ x१००)		

क्षेत्र	कोड	प्रमाणीकरण		
ड्रेसिङ्ग, ईन्जेक्शन र दैनिक प्रक्रियाहरु (DIRP)	२.३			
उपक्षेत्र उपक्षेत्र		मापदण्डहरु	प्राप्ताङ्क	उच्चतम अङ्क
२.३.१ काम गर्ने ठाऊँ	२.३.१	ड्रेसिङ्ग र ईन्जेक्सन लगाउने भिन्न ठाऊँको व्यवस्था छ।		१
२.३.२ फर्निचर र सामान्य उपकरण	२.३.२	पर्याप्त फर्निचर र सामान्य उपकरणको व्यवस्था छ। (हेर्नु परिशिष्ट २.३क DIRP को लागि फर्निचर र सामान्य उपकरण)		३
२.३.३ उपनब्ध सेवाहरु	२.३.३	न्यूनतम ड्रेसिङ्ग र ईन्जेक्सन सेवा र दैनिक प्रक्रियाहरु उपलब्ध छन्। (हेर्नु परिशिष्ट २.३ख DIRPका न्यूनतम सेवाहहरुको सूचि)		३
२.३.४ डिस्पोजेबल उपकरण	२.३.४	DIRP को लागि औषधीहरु र उपकरणहरु उपलब्ध छन्।(हेर्नु परिशिष्ट २.३ग DIRP को लागि औषधीहरु र उपकरणहरु)		३
२.३.५ निर्मलीकरणका उपकरण	२.३.५.१	पर्याप्त मात्रामा निर्मलीकरण गरिएका उपकरणहरु उपलब्ध छन्। (हेर्नु परिशिष्ट २.३घ DIRP का लागि निर्मलीकरण गरिएका उपकरणहरु)		३
	२.३.५.२	निर्मलीकृत गज र कटन बल्सको लागि फरक भाँडाहरु प्रयोग गरिन्छ।		१
२.३.६ मापदण्ड उपचार निर्देशिकाको प्रयोग र उपलब्धता		हेल्थ पोस्टमा स्तरिय उपचार पद्धति उपलब्ध छ र प्रयोग गरिन्छ।		१
२.३.७ संक्रमण रोकथाम र फोहोर व्यवस्थापन	२.३.७.१	मास्क, पञ्जा, प्लास्टिकको एप्रोन, बुट र चस्माहरु उपलब्ध छन् र आवश्यकताअनुसार प्रयोग गरिन्छ।		१
	२.३.७.२	कम्तिमा पनि तीन वटा फरक रङका फोहोर जम्मा गर्ने बाल्टीहरु (एक-एकवटा रातो, निलो र हरियो- HCWM सन् २०१४ को निर्देशिका (स्वास्थ्य तथा जनसंख्या मन्त्रालय) अनुसार) उपलब्ध छन् र प्रयोगमा छन्।		१
	२.३.७.३	निडल कटरसहितको ट्रलीको व्यवस्था छ।		१

	२.३.७.४	स्वास्थ्यकर्मीको लागि सफा पानी र साबुन वा स्यानिटाईजरले हात धुने व्यवस्था छ।		१
	२.३.७.५	विशुद्धिकरण गर्नको लागि क्लोरिनको झोल बनाईन्छ र प्रयोग गरिन्छ।		१
२.३.८ कागजात	२.३.८	सबै कार्यहरुको रेकर्ड गरिन्छ र रिपोर्ट गरिन्छ।		१
मापदण्ड २.३		कुल प्राप्ताङ्क		
		कुल प्रतिशत (कुल प्राप्ताङ्क / २१ x१००)		

परिशिष्ट २.३क DIRP का लागि फर्निचर र साधारण उपकरण

क्रमाङ्क	विवरण	आवश्यक संख्या	स्कोर
१	उपचारको लागि बेड (डसना\सिरानी)	१	
२	कुर्सी	१	
३	साबुन पानीसहितको बाल्टी	१	
४	निडिल कटर	१	
५	फ्ल्यास लाइट	१	
६	पोर्टेबल ल्याम्प	१	
७	भित्ते घडी	१	
कुल अङ्क			
कुल प्रतिशत = कुल अङ्क/७ X १००			

हरेक हरफले १ अङ्क पाउनेछ यदि सबै आवश्यक संख्या छ भने अन्यथा ०

स्कोरिङ्ग तालिका	
कुल प्रतिशत	स्कोर
०-४९	०
५०-६९	१
७०-८४	२
८५-१००	३
मापदण्डको स्कोर २.३.२	

परिशिष्ट २.३ख DIRP का लागि न्यूनतम सेवा

क्रमाङ्क	विवरण	आवश्यक संख्या	स्कोर
१	साधारण ड्रेसिङ्ग परिवर्तन	१	
२	छालाको टाँका निकाल्ने	१	
३	स्पिल्टिङ्ग	१	
४	घाऊको ड्रेसिङ्ग	१	
५	लोकल एनेस्थेसियामा ड्रेसिङ्ग परिवर्तन	१	
६	घाऊ चिरफार	१	
७	क्याथेटर (राख्ने र निकाल्ने)	१	
		कुल अङ्क	
		कुल प्रतिशत = कुल अङ्क/७ X १००	

हरेक हरफले १ अङ्क पाउनेछ यदि सबै आवश्यक संख्या छ भने अन्यथा ०

स्कोरिङ्ग तालिका	
कुल प्रतिशत	स्कोर
०-४९	०
५०-६९	१
७०-८४	२
८५-१००	३
मापदण्डको स्कोर २.३.३	

परिशिष्ट २.३ग DIRP का लागि औषधि र उपकरण

क्रमाङ्क	विवरण	आवश्यक संख्या	स्कोर
१	Lignocaine Hydrochloride १%	२	
२	Lignocaine Hydrochloride २%	२	
३	Povidine Iodine Solution (५००ml)	२	
४	Hydrogen Peroxide Solution	१	
५	कटन ब्याण्डेज	आवश्यकतानुसार	
६	सिल्क २-०	आवश्यकतानुसार	
७	प्रोलिन २-०, ३-०, ४-०	आवश्यकतानुसार (२-३)	
८	विभिन्न साइजका क्याथेटर	प्रत्येक साईजका २ वटा	
९	स्पिरिट (५००मि.लि)	१	
१०	एकल प्रयोग सूई १ मि.लि, ३ मि.लि, ५ मि.लि, १० मि.लि	आवश्यकतानुसार	
११	विभिन्न साइजका निर्मलीकृत पञ्जा	आवश्यकतानुसार	
१२	एकल प्रयोग पञ्जा	आवश्यकतानुसार	
१३	मास्क	आवश्यकतानुसार	
		कुल अङ्क	
		कुल प्रतिशत = कुल अङ्क/७ X १००	

हरेक हरफले १ अङ्क पाउनेछ यदि सबै आवश्यक संख्या छ भने अन्यथा ०

स्कोरिङ्ग तालिका	
कुल प्रतिशत	स्कोर
०-४९	०
५०-६९	१
७०-८४	२
८५-१००	३
मापदण्डको स्कोर २.३.४	

परिशिष्ट २.३घ DIRP का लागि निर्मलीकृत उपकरण

क्रमाङ्क	विवरण	आवश्यक संख्या	स्कोर
१	निर्मलीकृत ड्रेसिङ्ग सेट (र्यापरमा राखेको)	२-३	
२	निर्मलीकृत सुचर सेट (र्यापरमा राखेको)	२	
३	निर्मलीकृत सुचर हटाउने सेट (र्यापरमा राखेको)	१	
४	निर्मलीकृत क्याथेटर सेट (र्यापरमा राखेको)	२	
५	निर्मलीकृत चिटल फोरसेप (जारसहित)	२	
६	निर्मलीकृत कटन बल्स ड्रममा राखिएको	१ ड्रम	
७	निर्मलीकृत गज पिस र गज प्याड ड्रममा राखिएको	१ ड्रम	
		कुल अङ्क	
		कुल प्रतिशत = कुल अङ्क/७ X १००	

हरेक हरफले १ अङ्क पाउनेछ यदि सबै आवश्यक संख्या छ भने अन्यथा ०

स्कोरिङ्ग तालिका	
कुल प्रतिशत	स्कोर
०-४९	०
५०-६९	१
७०-८४	२
८५-१००	३
मापदण्डको स्कोर २.३.५.१	

क्षेत्र	कोड	प्रमाणीकरण		
औषधि सेवा (डिस्पेन्सरि)	२.३			
उपक्षेत्र उपक्षेत्र		मापदण्डहरु	प्राप्ताङ्क	उच्चतम अङ्क
२.४.१ औषधी सेवा	२.४.१	स्वास्थ्य चौकीमा औषधी सेवाको निश्चित क्षेत्र (डिस्पेन्सरि) छ।		१
२.४.२ नेशनल ड्रग फर्मुलरी	२.४.२	स्वास्थ्य चौकीको औषधी कक्षमा नेपाली नेशनल ड्रग फर्मुलरी (NNF) को प्रतिलिपि छ।		१
२.४.३ औषधी सेवा समय	२.४.३	औषधी कोठा OPD समयभरि खुला रहन्छ।		१
२.४.४ कर्मचारी परिचालन	२.४.४	औषधी सेवाको लागि मध्यम तहका स्वास्थ्यकर्मीहरुलाई तालिम दिइन्छ र परिचालन गरिन्छ।		१
२.४.५ आवश्यक निशुल्क औषधीहरुको उपलब्धता	२.४.५.१	देखिने ठाउँमा आवश्यक निशुल्क औषधीहरुको सूची रहेको छ।		१
	२.४.५.२	स्वास्थ्य चौकीमा आवश्यक आधारभुत सेवाका औषधीहरु वर्षभरि उपलब्ध छन् र स्टक आउट भएको छैन।		१
२.४.६ कार्यक्रमको लागि औषधी र उपकरणको उपलब्धता	२.४.६	वर्षभरि नै निर्देशिकाअनुसार कार्यक्रमको लागि औषधी र उपकरण उपलब्ध छन्		
	२.४.६.१	कुष्ठरोग		१
	२.४.६.२	मलेरिया (निश्चित जिल्लाहरुमा)		१*
	२.४.६.३	कालाजार		१
	२.४.६.४	हात्तीपाईले		१
	२.४.६.५	एच.आइ.भी\एड्स		१
	२.४.६.६	नसर्ने रोगहरु (आधारभूत स्वास्थ्य प्याकेज अनुसार)		१
	२.४.६.७	मानसिक स्वास्थ्यसम्बन्धी समस्याहरु (आधारभूत स्वास्थ्य प्याकेज अनुसार)		१
२.४.७ औषधीहरुको भण्डारण	२.४.७.१	सबै औषधीहरु र उपकरणहरु सफा र्‍याकमा वर्णानुक्रमानुसार वा समूहअनुसार राखिन्छ।		१
	२.४.७.२	औषधीहरुको तापक्रमलाइ(२५+/-२°C) निगरानी र रेकर्ड गरिन्छ।		१

	२.४.७.३	तापक्रम मिलाउनुपर्ने औषधीहरु र खोपहरु भण्डारण गर्न फ्रिजको(+/-४°C) व्यवस्था छ।		१
२.४.८ प्रेस्कृप्सनअनुसारको औषधी वितरण	२.४.८.१	बिरामीहरुलाई औषधीसम्बन्धीको जानकारी औषधी वितरणको समयमा दिइन्छ।		१
	२.४.८.२	औषधीसेवन कसरी गर्ने भन्नेबारे लिखित निर्देशन दिइन्छ।		१
	२.४.८.३	औषधी वितरण क्षेत्रमा औषधीको सही प्रयोग बारेमा IEC उपकरणहरु (पोस्टर, NNF) उपलब्ध छन्।		१
	२.४.८.४	प्रेस्कृप्सनमा औषधीको जेनेरिक नाम प्रयोग गरिन्छ।		१
२.४.९ STP उपलब्धता र प्रयोग	२.४.९	हेल्थ पोस्टमा मापदण्ड स्तरीय उपचार पद्धति निर्देशिका उपलब्ध छ र प्रयोग गरिन्छ।		१
२.४.१० पहिले म्याद सकिने औषधिलाई पहिले प्रयोग गर्ने (FEFO)	२.४.१०	पहिले म्याद सकिने औषधिलाई पहिले प्रयोग गर्ने (FEFO) प्रक्रियाको व्यवस्था गरिन्छ।		१
२.४.११ औषधीहरुको जाँचबुझ	२.४.११	सबै औषधीहरु र उपकरणहरु गिन्ति गरिन्छ, म्याद नाघेका औषधीहरु सही व्यवस्थापन भएको छ।		१
२.४.१२ औषधीजन्य फोहोरको व्यवस्थापन	२.४.१२	औषधीजन्य फोहोरको व्यवस्थापन औषधी व्यवस्थापन विभागको निर्देशिका अनुसार गरिन्छ।		१

* Malaria endemic districts

परिशिष्ट २.६क साधारण जाँचहरुको सूची

क्रमाङ्क	साधारण जाँचहरुको न्यूनतम सूची (BHS प्याकेजअनुसार)	उपलब्ध
१	रक्त समूह र आर्.एच् टाईपिङ्ग	
२	ब्लड सुगर	
३	हेमोग्लोबिन (%)	
४	पिसाबको रुटिन जाँच	
५	पिसाबद्वारा गर्भ जाँच	
६	दिसाको रुटिन जाँच	
७	किटोन बडिज	
८	प्रोटिनको लागि डिपस्टिक	
९	खकारको AFB जाँच	
१०	VIA (Visual Inspection with Acetic Acid) (भि.आई.ए)	
११	गर्भ जाँच	
१२	VDRL (भि.डि.आर.एल)	
१३	K-३९ RDT	
१४	ँऔलोको लागि RDT	
१५	डेङ्गूको लागि RDT	
१६	ँऔलोको लागि माइक्रोस्कोपी	
	कुल अङ्क	
	कुल प्रतिशत = कुल अङ्क / १६ x १००	

हरेक हरफले १ अङ्क पाउनेछ यदि सबै आवश्यक संख्या छ भने अन्यथा ०

स्कोरिङ्ग तालिका	
कुल प्रतिशत	स्कोर
०-४९	०
५०-६९	१
७०-८४	२
८५-१००	३
स्कोर मापदण्ड २.६.१.२	

परिशिष्ट २.६ख ल्याबरेटोरीको लागि फर्निचर र उपकरण

क्रमाङ्क	ल्याबरेटोरीको लागि फर्निचर र उपकरण	आवश्यक संख्या	स्कोर
१	काम गर्नको लागि टेबल	कम्तिमा ३	
२	बेन्च	कम्तिमा १	
३	कुर्सीहरु	कम्तिमा २	
४	दराज	कम्तिमा १	
५	ल्याबरेटोरीको लागि फ्रीज	कम्तिमा १	
६	डिटर्जेन्ट	आवश्यकतानुसार	
७	ह्याण्ड स्यानिटाइजर	आवश्यकतानुसार	
८	हात धुने साबुन	आवश्यकतानुसार	
९	एप्रोन	कम्तिमा २	
१०	तौलिया	आवश्यकतानुसार	
११	स्टेशनरी सामान (कलम, सिसाकलम, मार्कर, A४ कागज)	आवश्यकतानुसार	
१२	बिरामी रजिष्टर	कम्तिमा १	
१३	टेष्ट अनुसारका प्रेषण फारम र रिपोर्टिङ्ग फारम	आवश्यकतानुसार	
कुल अङ्क			
कुल प्रतिशत = कुल अङ्क / १३ x १००			

हरेक हरफले १ अङ्क पाउनेछ यदि सबै आवश्यक संख्या छ भने अन्यथा ०

स्कोरिङ्ग तालिका	
कुल प्रतिशत	स्कोर
०-४९	०
५०-६९	१
७०-८४	२
८५-१००	३
स्कोर Standard २.६.३.१	

परिशिष्ट २.६ ग ल्याबरेटोरीको लागि औजार, उपकरण र सामाग्रिको सूची

क्रमाङ्क	ल्याबरेटोरीको लागि औजार, उपकरण र सामाग्रि	आवश्यक संख्या	स्कोर
१	सेन्ट्रिफ्यूज	कम्तिमा १	
२	माइक्रोस्कोप	कम्तिमा १	
३	कलरिमिटर	कम्तिमा १	
४	वाटर बाथ	कम्तिमा १	
५	हट एयर ओभन	कम्तिमा १	
६	DC काउन्टर	कम्तिमा १	
७	ESR स्ट्याण्ड	कम्तिमा १	
८	ESR ट्यूब	आवश्यकतानुसार	
९	नेउबर च्याम्बर	कम्तिमा १	
१०	स्टप वाच\टाइमर	कम्तिमा १	
११	माइक्रोपिपेट	आवश्यकतानुसार	
१२	माइक्रोपिपेट स्ट्याण्ड	कम्तिमा १	
१३	रेफ्रिजिरेटर	१	
१४	रेफ्रिजिरेटरको तापक्रम मापन यन्त्र	१	
१५	अटोक्लेभ	१	
१६	स्टेनिङ्ग र्याक	१	
१७	HCWM निर्देशीका अनुसार फोहोर राख्ने कन्टेनर	१ सेट	
१८	ग्लासवेयरहरु (खानन ट्यूब, टेष्ट ट्यूब)	आवश्यकतानुसार	
१९	निर्दिष्ट जाँचहरुको लागि RDT किट	आवश्यकतानुसार	
२०	निर्दिष्ट जाँचहरुको लागि रियाजेन्ट	आवश्यकतानुसार	
२१	ड्राईड ब्लड स्पट (DBS)	आवश्यकतानुसार	
२२	जिपलक ब्याग	आवश्यकतानुसार	
२३	खाम	आवश्यकतानुसार	
२४	सिलिका जेल	आवश्यकतानुसार	
२५	माइक्रोस्कोपको लागि स्लाईड	आवश्यकतानुसार	
२६	ग्लास स्लाईड	आवश्यकतानुसार	
२७	कटन स्वाब	आवश्यकतानुसार	
२८	विभिन्न साईजका टिपहरु	आवश्यकतानुसार	
२९	विभिन्न साईजका एकल प्रयोग सूईहरु	आवश्यकतानुसार	
३०	भ्याकूटेनर	आवश्यकतानुसार	
३१	अल्कोहोल स्वाब	आवश्यकतानुसार	
३२	लानसेट	आवश्यकतानुसार	
३३	ब्लटिङ्ग पेपर	आवश्यकतानुसार	

३४	जाँच गर्ने पञ्जा	आवश्यकतानुसार	
३५	विभिन्न साईजका निर्मलीकृत पञ्जा	आवश्यकतानुसार	
३६	मास्कहरु	आवश्यकतानुसार	
३७	कोल्ड चेन बाकस	कम्तिमा १	
३८	आइस प्याक	कम्तिमा ३	
३९	स्पिरिट ल्याम्प	कम्तिमा १	
४०	टर्निकेट	कम्तिमा २	
४१	हात अड्याउने प्याड	कम्तिमा १	
४२	पिसाब र दिसाको लागि कन्टेनर	आवश्यकतानुसार	
४३	मापन सिलिन्डर (१००,१००० मि.लि)	कम्तिमा १ प्रत्येकमा	
४४	विकर (२००, ५०० मि.लि)	कम्तिमा १ प्रत्येकमा	
४५	बाँसको स्टिक	आवश्यकतानुसार	
४६	डिस्टिल पानी	आवश्यकतानुसार	
४७	Sodium hypochloride	आवश्यकतानुसार	
४८	ल्याब रियाजेन्ट	आवश्यकतानुसार	
४९	रक्त संकलनको ट्युब	आवश्यकतानुसार	
५०	रक्त नमूनाको लागि स्लाईड	आवश्यकतानुसार	
		कुल अङ्क	
		कुल प्रतिशत = कुल अङ्क / १३ x १००	

हरेक हरफले १ अङ्क पाउनेछ यदि सबै आवश्यक संख्या छ भने अन्यथा ०

स्कोरिङ्ग तालिका	
कुल प्रतिशत	स्कोर
०-४९	०
५०-६९	१
७०-८४	२
८५-१००	३
स्कोर Standard २.६.४.४	

क्षेत्र	कोड	प्रमाणीकरण (**बर्थिङ्ग केन्द्र भएका स्वास्थ्य चौकीको लागि)		
बर्थिङ्ग केन्द्र**	२.५			
उपक्षेत्र उपक्षेत्र		मापदण्डहरु	प्राप्ताङ्क	उच्चतम अङ्क
२.५.१ प्रसूति सेवाको उपलब्धता	२.५.१.१	गोपनीयता सहितको अलग पूर्व प्रसूति र प्रसूति कोठाको व्यवस्था गरिएको छ।		१
	२.५.१.२	२४ सै घण्टा प्रसूति सेवा उपलब्ध छ।		१
	२.५.१.३	इमर्जेन्सी सेवाको लागि सबै स्वास्थ्यकर्मीहरु अट्ने प्रसूति कोठाको व्यवस्था छ।		१
२.५.२ कर्मचारी परिचालन	२.५.२.१	चौबिसै घन्टा सेवाको लागि २ वटा SBA तालिमप्राप्त अ.न.मी वा स्टाफ नर्सको व्यवस्था छ।		१
	२.५.२.२	धेरै प्रसूति हुने ठाऊँमा अतिरिक्त सहयोगी कर्मचारीको व्यवस्था छ। (>=१० प्रसूति प्रति महिना)		१
	२.७.१.२.१.३	२४ सै घण्टा संक्रमण रोकथामबारेमा कम्तिमा तालिमप्राप्त कार्यालय सहयोगी उपलब्ध छ।		१
२.५.४ उचित तरिकाले पाटोग्राफ को प्रयोग र निर्णय प्रक्रिया	२.५.४.१	उचित तरिकाले पाटोग्राफ को प्रयोग गरिन्छ।		१
	२.५.४.२	जटिल समस्याहरुको सही पहिचान र सही समयमा प्रेसण गरिन्छ।		१
२.५.५ कम तौल भएका बच्चाहरुको लागि KMC (Kangaroo Mother Care)	२.५.५	कम तौल भएका बच्चाहरुको लागि KMC सेवा दिनलाई कम्तिमा एउटा KMC टेबलको व्यवस्था छ।		१
२.५.६ जन्मदर्ता प्रमाणपत्र	२.५.६	जन्मदर्ता प्रमाणपत्र दिईन्छ।		१
२.५.७ स्तरिय उपचार प्रोटोकल (STP) उपलब्धता र प्रयोग	२.५.७	स्तरिय उपचार प्रोटोकल(STP) उपलब्ध छ र प्रयोगमा पनि छ।		१
२.५.८ बिरामी परामर्श	२.५.८.१	पूर्व प्रसूति र प्रसूतिको बारेमा र सम्भावित जटिलताको विरामी र उसका आफन्तहरुलाई परामर्श गरिन्छ र लिखित अनुमति समेत लिईन्छ।		१

	२.५.८.२	प्रसूतिपश्चात्का आमा र बच्चामा देखिने खतराका चिन्हहरु, खोपसम्बन्धीका कामहरु, पोषण, सरसफाई र परिवार नियोजन सम्बन्धी स्वास्थ्य शिक्षा दिईन्छ।		१
	२.५.८.३	डिस्चार्ज हुनुपूर्व परिवार नियोजन र स्तनपानसम्बन्धी परामर्श दिइन्छ।		१
२.५.९ IEC/BCC[9] सामग्रिहरु	२.५.९	प्रसूतिपश्चात्को स्याहार, स्तनपान, पोषण, खोपसम्बन्धीका उपयुक्त IEC/BCC[10] सामग्रिहरु उपलब्ध छन्।		१
२.५.१० प्रसूति कोठाको लागि फर्निचर, उपकरण र औषधीहरुको व्यवस्था	२.५.१०.१	प्रसूति सेवा सम्बन्धी उपकरणहरु भण्डारणको लागि छुट्टै दराज छ।		१
	२.५.१०.२	प्रसूति सेवा सम्बन्धी उपकरणहरु र सामान्य साधनहरु पर्याप्त छन्। (हेर्नु परिशिष्ट २.५क प्रसूति कोठाको लागि फर्निचर, उपकरण र औषधीहरु)		३
	२.५.१०.३	प्रसूति कोठाको लागि उपकरण र औषधीहरुको व्यवस्था छ।(हेर्नु परिशिष्ट २.५ख प्रसूति कोठाको लागि उपकरण र औषधीहरु)		३
	२.५.१०.४	प्रसूति कोठाको लागि उपकरण र औषधीहरुको इमर्जेन्सी ट्रलीको व्यवस्था छ। (हेर्नु परिशिष्ट २.५ग प्रसूति कोठाको लागि उपकरण र औषधीहरु राख्ने इमर्जेन्सी ट्रली)		३
२.५.११ बिरामीका लागि सुविधा	२.५.१.१	२४ सै घण्टा सफा खानेपानीको व्यवस्था छ।		१
	२.५.१`.२	पूर्व प्रसूति कोठामा बिरामीको लागि फरक चर्पीको व्यवस्था छ।		१
२.५.१२ HIV संक्रमणको शंका भएका वा निदान भएका बिरामीहरुको व्यवस्थापन र प्रेसण	२.५.१२.१	सुकेका रगत का टाटा (Dried Blood Spot) लाई ART[11] केन्द्रमा पठाइन्छ।		१
	२.५.१२.२	एक्सपोजर पश्चात प्रोफाइलाक्सिसको लागि नवजातलाई दिनको लागि नेभिरापिन उपलब्ध छ र निर्देशिकाअनुसार दिईन्छ।		१
	२.५.१२.३	प्रेसण परामर्श गरेर ART केन्द्रमा प्रेसण गरिन्छ।		१

9 IEC/BCC= Information Education and Communication/ Behavior Change Communication
10 IEC/BCC= Information Education and Communication/ Behavior Change Communication
11 ART= Antiretroviral treatment

२.५.१३ संक्रमण रोकथाम	२.५.१३.१	व्यक्तिगत सुरक्षाका उपकरण, साधनहरु उपलब्ध छन् र आवश्यकताअनुसार प्रयोग गरिन्छ ।		१
	२.५.१३.२	प्रसूति कोठा धुनमिल्ने छ ।		१
	२.५.१३.३	प्रसूति कोठामा प्रयोगको लागि फरक चप्पल र हात धुने साबुनको व्यवस्था छ ।		१
	२.५.१३.४	कम्तिमा पनि तीन वटा फरक रङ्का फोहोर जम्मा गर्ने बाल्टीहरु (एक-एकवटा रातो, निलो र हरियो- HCWM सन् २०१४ को निर्देशिका (स्वास्थ्य तथा जनसंख्या मन्त्रालय अनुसार) उपलब्ध छन् र प्रयोगमा छन् ।		१
	२.५.१३.५	स्वास्थ्यकर्मीको लागि सफा पानी र साबुन वा स्यानिटाईजरले हात धुने व्यवस्था छ ।		१
	२.५.१३.६	निडिल कटर र सेफ्टी बाकसको प्रयोग गरिन्छ ।		१
	२.५.१३.७	विशुद्दिकरण गर्नको लागि सोडियम हाईड्रोक्लोराईडको झोल बनाईन्छ र प्रयोग गरिन्छ ।		१
	२.५.१३.८	सफा भाडामा सुख्खा गज र कटनहरु फरक ठाऊँमा राखिन्छ ।		१
	२.५.१३.९	प्लास्टिक र सालको लागि फरक कचौराको प्रयोग गरिन्छ ।		१
	२.५.१३.१०	साललाई खाल्टोमा पुरिन्छ वा placenta pit छ ।		१
मापदण्ड २.५		कुल प्राप्ताङ्क		४१
		कुल प्रतिशत = कुल प्राप्ताङ्क / ४१ x१००		

परिशिष्ट २.५क प्रसूति कोठाको लागि फर्निचर, उपकरण र औषधीहरु

क्रमाङ्क	विवरण	आवश्यक संख्या	स्कोर
१	प्रसूति बेड	कम्तिमा १	
२	सफा तन्ना	प्रत्येक बेडमा	
३	पर्दा	आवश्यकतानुसार	
४	सफा ठाँऊ (अर्को प्रसूतिको लागि)	Available	
५	नवजात शिशुको पुनर्जीवन टेबल	१	
६	बत्ती	१	
७	कोठाको हिटर	१	
८	बच्चाको हिटर	१ per delivery bed	
९	अक्सिटोसिन भण्डारणको लागि फ्रीज	१	
उपकरण र सामानहरु			
१	प्रेसर मापन सेट र स्टेथेस्कोप	१	
२	थर्मोमिटर (मर्करी रहित)	१	
३	कोठाको लागि थर्मोमिटर	१	
४	फिटोस्कोप	२	
५	भ्रुण स्टेथेस्कोप	१	
६	बच्चा जोख्ने यन्त्र	१	
७	नवजातको साईजको सेल्फ ईन्फ्लेटिङ्ग ब्याग र मास्क	१	
८	सक्सन सहितको म्यूकस ईक्सट्राक्टर (पेनगुईन)	२	
९	डप्लर	१	
१०	भजाईनल स्पेकुलम (सिम्स)	२	
११	नवजात शिशुको पुनर्जीवन किट	१	
१२	वयस्क पुनर्जीवन किट	१	
१३	निर्मलीकृत प्रसूति उपकरण (हरेक सेटलाई हेर्नुहोस्)	२ सेट डेलिभरी बेडको लागि	
१३.१	स्पोन्ज फोरसेप	२	
	आर्टरी फोरसेप	२	
	S/S बाउल (Galli pot)	१	
	S/S बाउल (receive placenta) (१-२ लि)	१	
	कर्ड कटिङ्ग कैची (बोधो खालको)	१	
	कर्ड टाई\ कर्ड क्ल्याम्प	२	
	प्लाष्टिक\रबर को पाता	१	
	गज स्वाब	४	
	चारपाटे कपडा	३	
	किड्नी ट्रे	१	

	पेरिप्याड\ ठूलो ड्रेसिङ्ग प्याड	२	
	लेगिङ्ग	२	
	पेरिनियल सिट	१	
	बच्चा रिसिभ गर्ने टावेल	१	
	निर्मलीकृत गाउन	१	
१४	टाँका लगाउने सेट (हरेक सेटलाई हेर्नुहोस्)	२सेट	
१४.१	निडिल होल्डर	१	
	स्पोन्ज होल्डर	१	
	टाँका काट्ने कैँची	१	
	डिसेक्टिङ्ग फोरसेप (टुथ र प्लेन)	२	
	आर्टरी फोरसेप	१	
	गलीपोर्ट	२	
१५	ईपिसियोटोमी सेट (हरेक सेटलाई हेर्नुहोस्)	२ सेट	
१५.१	ईपिसियोटोमी कैची	१	
	निडिल होल्डर	१	
	टाँका काट्ने कैँची	१	
	डिसेक्टिङ्ग फोरसेप (टुथ र प्लेन)	२	
	आर्टरी फोरसेप	१	
१६	भ्याकुम सेट	२	
१७	प्रसूतिको लागि फोरसेप सेट	१	
		कुल अङ्क	
		कुल प्रतिशत= कुल अङ्क/२६x१००	

हरेक हरफले १ अङ्क पाउनेछ यदि सबै आवश्यक संख्या छ भने अन्यथा ०

स्कोरिङ्ग तालिका	
कुल प्रतिशत	स्कोर
०-४९	०
५०-६९	१
७०-८४	२
८५-१००	३
मापदण्डको स्कोर २.५.१०.२	

परिशिष्ट २.५ ख प्रसूति कोठाको लागि औषधि र सामानहरु

क्रमाङ्क	औषधि र उपकरण	आवश्यक संख्या	स्कोर
**औषधि**			
१	Oxytocin injection (keep in २-८oC)	२० एम्पूल	
२	Tranexamic acetate injection	१० एम्पूल	
३	Ergometrine injection	१० एम्पूल	
४	Magnesium sulphate injection	५० एम्पूल	
५	Calcium gluconate injection	३ एम्पूल	
६	Diazepam injection	१०	
७	Labetolol injection	१०	
८	Ampicillin injection	१०	
९	Gentamycin injection	५	
१०	Metronidazole injection	५	
११	Lignocaine injection	२	
१२	Adrenaline injection	५	
१३	Ringers' lactate injection	१०	
१४	Normal saline injection	१०	
१५	Dextrose ५% injection	१०	
१६	Water for injection	५	
१७	Eye antimicrobial (१% silver nitrate or Tetracycline १% eye ointment)	२	
१८	Povidone iodine	५	
१९	Tetracycline १% eye ointment	२	
२०	Paracetamol Tablet	२०	
२१	Nefidipine SL Tablet ५ mg	४ ट्याब	
२२	Misoprostol Tablet	५ ट्याब	
**उपकरण**			
१	सूई र निडिल	२०	
२	IV सेट	१०	
३	स्पिरिट (७०% अल्कोहोल)	१ बोतल	
४	कटन सहितको स्टील ड्रम	१	
५	क्याथेटर (प्लेन र फोलिज)	५ प्रत्येकमा	
६	टियर वा इपिजियोटोमीको सिलाउन टाँका (२.० क्रोमिक क्याटगट)	५ पिस	
७	ब्लिच (क्लोरिनमा आधारित रसायन)	२ प्याकेट	

८	आमाको तल राख्नको लागि सफा (प्लाष्टिक) सिट	४	
९	स्यानिटरी प्याड	१ बाकस	
१०	पेरि-प्याड	आवश्यकतानुसार	
११	शिशुलाई सुख्खा राख्न र र्याप गर्नलाई सफा कपडा	५	
१२	कर्ड टाई- निर्मलीकृत	१०	
१३	बच्चाको लागि ब्ल्याङ्केट	५	
१४	बच्चालाई खुवाउने कप	३	
१५	औषधि मिसिएको झुल	२	
१६	सरसफाईको पञ्जा	२ जोर	
१७	निर्मलीकरण गरिएको पञ्जा	२ जोर प्रत्येकको फरक साईज	
१८	लामो प्लाष्टिकको एप्रोन	२	
१९	गगल्स	२	
२०	धारिला वस्तुको लागि कन्टेनर	१	
२१	निडिल कटर	१	
२२	फोहोर कपडा राख्ने कन्टेनर	१	
२३	फोहोर प्याड र स्वाबको लागि बाल्टी	२	
२४	सालको लागि बाल्टी (५ लि.)	२	
२५	HCWM निर्देशिकाअनुसार कलर कोड गरिएका बाल्टी	१ सेट	
२६	भित्ते घडी	१	
२७	ब्याट्री र चीमसहितको टर्च	१-२	
२८	मातृशिशु रजिष्टर	१-२	
२९	जन्म दर्ता प्रमाणपत्र	आवश्यकतानुसार	
३०	पार्टोग्राफ	आवश्यकतानुसार	
		कुल अङ्क	
		कुल प्रतिशत= कुल अङ्क/५२ x १००	

हरेक हरफले १ अङ्क पाउनेछ यदि सबै आवश्यक संख्या छ भने अन्यथा ०

स्कोरिङ्ग तालिका	
कुल प्रतिशत	स्कोर
०-४९	०
५०-६९	१
७०-८४	२
८५-१००	३
मापदण्डको स्कोर २.५.१०.३	

परिशिष्ट २.५ग प्रसूति कोठाको ER ट्रलीका औषधि र साधनहरु

क्रमाङ्क	नाम	आवश्यक संख्या	स्कोर
१	Atropine Injection		
२	Adrenaline Injection		
३	Xylocaine १% and २% Injections with Adrenaline		
४	Xylocaine १% and २ % Injections without Adrenaline		
५	Xylocaine Gel		
६	Diclofenac Injection		
७	Hyoscine Butylbromide Injection		
८	Diazepam injection		
९	Morphine Injection / Pethidine Injection		
१०	Hydrocortisone Injection		
११	Chlorpheniramine meliate Injection		
१२	Dexamethasone Injection		
१३	Ranitidine/Omeperazole Injection		
१४	Frusemide Injection		
१५	Dopamine injection		
१६	Noradrenaline injection		
१७	Digoxin injection		
१८	Verapamil injection		
१९	Amidarone injection		
२०	Glyceryl trinitrate/nitroglycerine injection		
२१	Labetolol injection		
२२	Magnesium sulphate injection		
२३	Calcium gluconate injection		
२४	Sodium bicarbonate injection		
२५	Ceftriaxone Injection		
२६	Metronidazole Injection		
२७	Dextrose २५%/ ५०% Injection		
२८	IV Infusion set (Adult/Pediatric)		
२९	IV Canula (१६, १८, २०, २२, २४, २६ Gz)		
३०	Disposable syringes १ ml, ३ ml, ५ ml, १० ml, २० ml, ५० ml		

३१	Disposable Gloves ६, ६.५, ७, ७.५		
३२	Water for injection १० ml		
३३	Sodium chloride-१५%w/v and Glycerin-१५% w/v (for enema)		
३४	PPH व्यवस्थापन सेट		
	• (IV canula: १६/१८G, IV fluids as per treatment protocol, IV set, Foley's catheter, Urobag)		
	• Condom tamponade set- Sponge holder:२, Sim's speculum:१, Foley's catheter: १, Condom:२, IV fluids: NS१, IV set, Thread, Cord Clamp),		
	• Inj Oxytocin, Tab Misoprostol,		
३५	Eclampsia व्यवस्थापन सेट		
	(Knee hammer, IV canula: १६/१८G, IV fluids, IV set, Foley's catheter, Urobag, ambu bag, Oxygen, Inj MgSO4: ४६ एम्पूल, Inj lignocaine २%, Inj Calcium gluconate, Distilled water, Disposable syringe २०ml-१, १०ml-८, Cap Nifedipin- ५mg ४ Cap)		
		कुल अङ्क	
		कुल प्रतिशत= कुल अङ्क/५२ x १००	

हरेक हरफले १ अङ्क पाउनेछ यदि सबै आवश्यक संख्या छ भने अन्यथा ०

स्कोरिङ्ग तालिका	
कुल प्रतिशत	स्कोर
०-४९	०
५०-६९	१
७०-८४	२
८५-१००	३
स्कोर Standard २.५.१०.४	

क्षेत्र	कोड	प्रमाणीकरण		
प्रयोगशाला सेवा (प्रयोगशाला भएका संस्थाको लागि)	२.६			
उपक्षेत्र उपक्षेत्र		मापदण्डहरु	प्राप्ताङ्क	उच्चतम अङ्क
२.६.१ बिरामीको लागि समय	२.६.१	प्रयोगशाला सेवा बिहान १० बजेबाट बेलुका ४ बजेसम्म दिइन्छ ।		१
	२.६.१.२	आधारभूत जाँचहरु गरिन्छ ।(हेर्नु परिशिष्ट २.६क आधारभूत जाँचहरुको सूची)		३
२.६.२ कर्मचारी परिचालन	२.६.२	प्रयोगशाला सेवाको लागि कम्तिमा एकजना तालिमप्राप्त ल्याब असिस्टेन्ट् वा टेक्निसियनको व्यवस्था छ ।		१
२.६.३ साधन र उपकरण	२.६.३.१	प्रयोगशाला सेवाको लागि प्रयोग गर्न सकिने साधन र उपकरणहरु उपलब्ध छन् । (हेर्नु परिशिष्ट २.६ख प्रयोगशाला सेवाको लागि साधन र उपकरणहरु)		३
	२.६.३.२	उत्पादकको मापदण्डअनुसार साधनहरु उपलब्ध छन् ।		१
२.६.४ भौतिक सुविधा	२.६.४.१	स्वास्थ्य चौकीमा प्रयोगशाला सेवाको लागि फरक ठाऊँ छ ।		१
	२.६.४.२	स्वास्थ्यकर्मी र कम्तिमा २ जना बिरामीको लागि आरामदायी बस्ने व्यवस्था छ ।		१
	२.६.४.३	पर्याप्त उज्यालो र हावा खेल्ने ठाऊँ छ।		१
	२.६.४.४	आवश्यक फर्निचर र उपकरणहरु उपलब्ध छन् । (हेर्नु परिशिष्ट २.६ग प्रयोगशाला सेवाको लागि आवश्यक फर्निचर र उपकरणहरु)		१
२.६.५ ड्युटी तालिका	२.६.५	ल्यावका कर्मचारीको ड्युटी तालिका नियमित रुपमा बनाईन्छ र देखिने गरि राखिन्छ ।		१
२.६.६ रेकर्डिङ्ग र रिपोर्टिङ्ग	२.६.६.१	विरामीको पूर्ण विवरणसहितको फारममा नमूनाहरुको रेकर्ड राखिन्छ ।		१
	२.६.६.२	सबै रिपोर्टहरु मापदण्ड रजिस्टरमा रेकर्ड गरिन्छ र मापदण्ड रिपोर्टिङ्ग फारमहरुको प्रयोग गरिन्छ ।		१

	२.६.६.३	रिपोर्ट दिनुपूर्व रिपोर्टमा पर्याप्त जानकारि छ कि छैन भनेर जिम्मेवार व्यक्तिले जाँच गर्छ ।		१
२.६.७ उपकरण भण्डारण र स्टक	२.६.७.१	कम्तिमा ३ महिनाको प्रयोगशाला उपकरणको बफर स्टक राखिएको छ ।		१
	२.६.७.२	रिएजेन्टहरुलाई उचित तापक्रममा भण्डारण गरिन्छ ।		१
२.६.८ संक्रमण रोकथाम	२.६.८.१	देखिने गरि जैविक हानिका चिन्हहरु राखिएको छ ।		१
	२.६.८.२	रसायन पोखिदा वा अरु घटनाहरु हुँदा के गर्ने भन्ने सबै कर्मचारीलाई जानकारी छ ।		१
	२.६.८.३	मास्क र पञ्जा उपलब्ध छन् र तिनको प्रयोग गरिन्छ ।		१
	२.६.८.४	कम्तिमा पनि तीन वटा फरक रङका फोहोर जम्मा गर्ने बाल्टीहरु (एक-एकवटा रातो, निलो र हरियो- HCWM सन् २०१४ को निर्देशिका (स्वास्थ्य तथा जनसंख्या मन्त्रालय) अनुसार) उपलब्ध छन् र प्रयोगमा छन् ।		१
	२.६.८.५	स्वास्थ्यकर्मीको लागि सफा पानी र साबुन वा स्यानिटाईजरले हात धुने व्यवस्था छ ।		१
	२.६.८.६	निडिल कटर र सेफ्टी बाकसको प्रयोग गरिन्छ ।		१
	२.६.८.७	विशुद्दिकरण गर्नको लागि क्लोरिनको झोल बनाईन्छ र प्रयोग गरिन्छ ।		१
मापदण्ड २.९.१		कुल स्कोर		२६
		कुल प्रतिशत (कुल स्कोर/ २६x१००)		

खण्ड ३ स्वास्थ्य चौकी सहयोग सेवा व्यवस्थापन

खण्ड ३ का मापदण्डहरुको सारांश

क्षेत्र	कुल मापदण्ड संख्या	कुल अङ्क	कुल प्राप्ताङ्क (प्रतिशतमा)
औजार प्रशोधन र निर्मलीकरण	१५	१७	
लुगा धुने ठाऊँ	१४	१६	
सरसफाई	११	१३	
उर्जा व्यवस्थापन	३	३	
पानी व्यवस्थापन	४	४	
स्वास्थ्य सम्बन्धी फोहोर व्यवस्थापन	१४	१४	
सुरक्षा र संरक्षण	१०	१२	
यातायात र संचार	५	५	
भण्डारण (मेडिकल र मसलन्द)	१०	१०	
कुल	८६	९४	

क्षेत्र	कोड	प्रमाणीकरण		
उपकरण प्रशोधन र निर्मलीकरण	३.१			
उपक्षेत्र उपक्षेत्र		मापदण्डहरु	प्राप्ताङ्क	उच्चतम अङ्क
३.१.१ ठाऊँ	३.१.१	उपकरण प्रशोधन र निर्मलीकरणको लागि सफा पानीको सुविधासहितको ठाऊँको व्यवस्था छ।		१
३.१.२ कर्मचारी परिचालन	३.१.२	उपकरण प्रशोधन र निर्मलीकरणको लागि फरक कर्मचारीको व्यवस्था गरिएको छ।		१
३.१.३ साधन र उपकरण	३.१.३	निर्मलीकरण गर्ने साधन र उपकरणहरु २४ सै घण्टा प्रयोगयोग्य अवस्थामा उपलब्ध छन्। (हेर्नु परिशिष्ट ३.१ क निर्मलीकरण गर्ने साधन र उपकरणहरु)		३
३.१.४ उपकरणको तयारी	३.१.४	र्यापर, गज, कटन बल र पट्टीहरु तयार गरिन्छ।		१
३.१.५ निर्मलिकरणको तयारी	३.१.५.१	रसायन र डिटर्जेन्टको प्रोग गरेर फरक कोठामा सबै प्रयोग गरिएको उपकरणहरु सफा गरिन्छ।		१
	३.१.५.२	फरक ठाऊँमा सबै उपकरण र साधनहरु सकाईन्छ।		१
	३.१.५.३	सबै उपकरणहरु दोहोरो र्यापरमा राखिन्छ।		१

३.१.६ निर्मलिकरण	३.१.६	सबै र्याप गरिएका उपकरणहरुलाई तापक्रमको ईन्डिकेटर राखेर अटोक्लेभ गरिन्छ ।		१
३.१.७ भण्डारण	३.१.७	निर्मलिकरण गरिएका प्याकेटहहरुलाई निर्मलिकरणको मिति हालेर दराजमा राखिन्छ ।		१
३.१.८ निरिक्षण र लग बुक	३.१.८.१	सबै उपकरणहरुको निरिक्षण गरेर रेकर्ड गरिन्छ ।		१
	३.१.८.२	लग बुकमा उपकरणको प्रशोधन र अटोक्लेभ गरेको मिति, उपकरणको प्रकारवारेमा सबै जानकारी राखिन्छ ।		१
३.१.९ संक्रमण रोकथाम	३.१.९.१	मास्क, पञ्जा, प्लास्टिकको एप्रोन, बुट र चस्माहरु उपलब्ध छन् र आवश्यकताअनुसार प्रयोग गरिन्छ ।		१
	३.१.९.२	कम्तिमा पनि तीन वटा फरक रङ्का फोहोर जम्मा गर्ने बाल्टीहरु (एक-एकवटा रातो, निलो र हरियो- HCWM सन् २०१४ को निर्देशिका (स्वास्थ्य तथा जनसंख्या मन्त्रालय) अनुसार) उपलब्ध छन् र प्रयोगमा छन् ।		१
	३.१.९.३	स्वास्थ्यकर्मीको लागि सफा पानी र साबुन वा स्यानिटाईजरले हात धुने व्यवस्था छ ।		१
	३.१.९.४	विशुद्दिकरण गर्नको लागि क्लोरिनको झोल बनाईन्छ र प्रयोग गरिन्छ ।		१
मापदण्ड ३.१		कुल प्राप्ताङ्क		१७
		प्रतिशत = कुल प्राप्ताङ्क / १७ x १००		

परिशिष्ट ३.१ क निर्मलीकरण गर्ने साधन र उपकरणहरु

क्रमाङ्क	नाम	आवश्यक संख्या	स्कोर
१	काम गर्ने टेबल	१	
२	ओसारपोसारको लागि बास्केट	२	
३	सर्जिकल ड्रम	२	
४	भण्डारण दराज	१	
५	बोइलर वा मःम पट	१	
६	अटोक्लेभ मेसिन	१	
७	डबल र्यापर	आवश्यकतानुसार	
८	टाईमर	१	
९	थर्मल सूचक टेप	आवश्यकतानुसार	
१०	क्याप, मास्क, गाउन, एप्रोन	आवश्यकतानुसार	
११	पञ्जाहरु	१ बाकस	
१२	कटन रोल	आवश्यकतानुसार	
१३	कटन गज	आवश्यकतानुसार	
१४	कैंची	२	
१५	बाल्टीहरु	३	
१६	स्कर्ब ब्रस	आवश्यकतानुसार	
		कुल अङ्क	
		कुल प्रतिशत = कुल अङ्क/१६ X १००	
		हरेक हरफले १ अङ्क पाउनेछ यदि सबै आवश्यक संख्या छ भने अन्यथा ०	

स्कोरिङ्ग तालिका	
कुल प्रतिशत	स्कोर
०-४९	०
५०-६९	१
७०-८४	२
८५-१००	३
मापदण्डको स्कोर ३.१.३	

क्षेत्र	कोड	प्रमाणीकरण		
लन्ड्री सेवा (लुगा धुने व्यवस्था)	३.२			
उपक्षेत्र उपक्षेत्र		मापदण्डहरु	प्राप्ताङ्क	उच्चतम अङ्क
३.२.१ ठाऊँ	३.२.१.१	उपकरण प्रशोधन र निर्मलीकरणको लागि सफा पानीको सुविधासहितको ठाऊँको व्यवस्था छ।		१
	३.२.१.१	लन्ड्रीको लागि फरक ठाऊँको व्यवस्था छ।		१
	३.२.१.२	सफा कपडाको लागि फरक दराजको व्यवस्था छ।		१
३.२.२ कर्मचारी परिचालन	३.२.२	संक्रमण रोकथामको तालिम लिएको कर्मचारीले लन्ड्री सेवा दिन्छ।		१
३.२.३ साधन र उपकरण	३.२.३	लन्ड्री सेवाको लागि साधन र उपकरणहरु उपलब्ध छन्। (हेर्नु परिशिष्ट ३.२ क लन्ड्री सेवाको लागि साधन र उपकरणहरु)		३
३.२.४ कपडाहरुको वर्गमा विभाजन र किटाणुहीन बनाइने	३.२.४.१	धुनुभन्दा पहिले कपडाहरुलाई रगत लागेको वा नलागेको जस्ता वर्गमा विभाजन गरिन्छ।		१
	३.२.४.२	विभाजित कपडाहारुलाई किटाणुरहित बनाईन्छ।		१
३.२.५ धुलाई	३.२.५	वासिङ्ग मेशिनमामात्र सवै कपडाहरु धोइन्छ।		१
३.२.६ सुकाई	३.२.६.१	सवै कपडाहरु घाममा सुकाईन्छ।		१
३.२.७ भण्डारण	३.२.७	सवै कपडाहरुलाई फरक दराजमा राखिन्छ।		१
३.२.८ निरीक्षण	३.२.८	सवै कपडाहरुलाई निरीक्षण र रेकर्ड गरिन्छ।		१
३.२.९ संक्रमण रोकथाम	३.२.९.१	मास्क, पञ्जा, प्लास्टिकको एप्रोन, बुट र चस्माहरु उपलब्ध छन् र आवश्यकताअनुसार प्रयोग गरिन्छ।		१
	३.२.९.२	कम्तिमा पनि तीन वटा फरक रङका फोहोर जम्मा गर्ने बाल्टीहरु (एक-एकवटा रातो, निलो र हरियो- HCWM सन् २०१४ को निर्देशिका (स्वास्थ्य तथा जनसंख्या मन्त्रालय) अनुसार) उपलब्ध छन् र प्रयोगमा छन्।		१

	३.२.९.३	स्वास्थ्यकर्मीको लागि सफा पानी र साबुन वा स्यानिटाईजरले हात धुने व्यवस्था छ ।		१
	३.२.९.४	विशुद्दिकरण गर्नको लागि क्लोरिनको झोल बनाईन्छ र प्रयोग गरिन्छ ।		१
मापदण्ड ३.२		कुल प्राप्ताङ्क		१६
		प्रतिशत = कुल प्राप्ताङ्क / १६ x १००		

परिशिष्ट ३.२ क लन्ड्री सेवाको लागि साधन र उपकरणहरु

क्रमाङ्क	नाम	आवश्यक संख्या	स्कोर
१	भण्डारण दराज	१	
२	बाल्टी \ बेसिन	३	
३	काठको स्टिरिरर	२	
४	बुट	२ जोर	
५	क्याप, मास्क, गाउन	आवश्यकतानुसार	
६	सुकाउने डोरी	आवश्यकतानुसार	
७	स्क्रब ब्रस	आवश्यकतानुसार	
८	सरसफाई पञ्जा	आवश्यकतानुसार	
९	धुलाई पाउडर	आवश्यकतानुसार	
१०	क्लोरिनको झोल\पाउडर	आवश्यकतानुसार	
		कुल अङ्क	
		कुल प्रतिशत = कुल अङ्क/१६ X १००	

हरेक हरफले १ अङ्क पाउनेछ यदि सबै आवश्यक संख्या छ भने अन्यथा ०

स्कोरिङ्ग तालिका	
कुल प्रतिशत	स्कोर
०-४९	०
५०-६९	१
७०-८४	२
८५-१००	३
मापदण्डको स्कोर ३.२.३	

क्षेत्र	कोड	प्रमाणीकरण		
सरसफाई	३.३			
उपक्षेत्र उपक्षेत्र		मापदण्डहरु	प्राप्ताङ्क	उच्चतम अङ्क
३.३.१ भण्डारणको ठाऊँ	३.३.१	सरसफाईका आधारभूत उपकरणहरु फरक दराजमा भण्डारण गरिन्छ ।		१
३.३.२ कर्मचारी परिचालन	३.३.२.१	सरसफाई गर्ने कर्मचारीलाई संक्रमण रोकथामको तालिम दिइन्छ ।		१
३.३.३ आधारभूत उपकरणहरु	३.३.३.१	आधारभूत उपकरणहरु उपलब्ध छन् ।(हेर्नु परिशिष्ट ३.३क सरसफाईका आधारभूत उपकरणहरु)		३
३.३.४ सरसफाई	३.३.४.१	स्वास्थ्य चौकी परिसर सफा र धूलोमुक्त छ ।		१
	३.३.४.२	सवै चर्पीहरु दुर्गन्धरहित र सफा छन् ।		१
	३.३.४.३	सवै चर्पीहरु कम्तिमा दिनको ३ पटक सफा गरिन्छ ।		१
	३.३.४.४	सबै ढोका र झ्यालहरु सफा र धूलोमुक्त छन् र दिनमा कम्तिमा एकपटक सफा गरिन्छ ।		१
	३.३.४.५	स्वास्थ्य चौकीका सवै भुईँहरु सफा छन् ।		१
	३.३.४.६	स्वास्थ्य चौकीका सवै भित्ताहरु सफा छन् र तिनमा टाईल लगाईएको छ वा ईनामेलले ४ फिटसम्म रङ्गाईएको छ ।		१
३.३.५ क्लोरिन झोलको व्यवस्थापन	३.३.५	क्लोरिन झोललाई छुट्टै नालीबाट बगाईन्छ वा छुट्टै फाल्डोमा पुरिन्छ।		१
३.३.६ हरियाली र खुला ठाऊँ	३.३.६	स्वास्थ्य चौकीमा हरियाली र खुला ठाऊँको व्यवस्था छ ।		१
मापदण्ड ३.३		कुल प्राप्ताङ्क		१३
		प्रतिशत = कुल प्राप्ताङ्क / १३ x १००		

परिशिष्ट ३.३क सरसफाईका आधारभूत उपकरणहरु

क्रमाङ्क	विवरण	आवश्यक संख्या	स्कोर
१	दराज	१	
२	रेकर्डको लग बुक	१	
३	हसिया	आवश्यकतानुसार	
४	कोदालो	आवश्यकतानुसार	
५	बेल्चा	आवश्यकतानुसार	
६	डोरी	आवश्यकतानुसार	
७	स्क्रब ब्रस	आवश्यकतानुसार	
८	कुचो	आवश्यकतानुसार	
९	बाल्टी	आवश्यकतानुसार	
१०	जार	आवश्यकतानुसार	
११	पानि छर्कने पाईप	आवश्यकतानुसार	
१२	साबुन	आवश्यकतानुसार	
१३	धुलाई पाउडर	आवश्यकतानुसार	
१४	फेरबदलका तन्ना	आवश्यकतानुसार	
१५	फेरबदलका सिरानी	आवश्यकतानुसार	
१६	सिरानिको खोल	आवश्यकतानुसार	
१७	ब्ल्याङ्केट	आवश्यकतानुसार	
१८	व्यक्तिगत सुरक्षाका साधन	आवश्यकतानुसार	
१९	झ्यालको जाली	सबै झ्यालहरुमा	
२०	मच्छड दानी	आवश्यकतानुसार	
२१	गमला	आवश्यकतानुसार	
		कुल अङ्क	
		कुल प्रतिशत = कुल अङ्क/२१ X १००	

हरेक हरफले १ अङ्क पाउनेछ यदि सबै आवश्यक संख्या छ भने अन्यथा ०

स्कोरिङ्ग तालिका	
कुल प्रतिशत	स्कोर
०-४९	०
५०-६९	१
७०-८४	२
८५-१००	३
स्कोर ३.३.३	

क्षेत्र	कोड	प्रमाणीकरण		
सरसफाई	३.३			
उपक्षेत्र उपक्षेत्र		मापदण्डहरु	प्राप्ताङ्क	उच्चतम अङ्क
३.४.३ उर्जा प्रणालीको व्यवस्था	३.४.३.१	स्वास्थ्य चौकीमा ३-फेज लाईनको विध्युत संचार छ ।		१
	३.४.३.२	स्वास्थ्य चौकीमा वैकल्पिक ऊर्जाको स्रोतहरु (जेनेरेटर, सोलार वा इन्भर्टर) छन् जसले उपकरणहरु चलाउन सकिन्छ ।		१
	३.४.३.३	आवश्यक डिजेल वा पेट्रोलको व्यवस्था छ ।		१
मापदण्ड ३.३		कुल प्राप्ताङ्क		३
		प्रतिशत = कुल प्राप्ताङ्क / १३ x १००		

क्षेत्र	कोड	प्रमाणीकरण		
पानीको स्रोत	३.५			
उपक्षेत्र उपक्षेत्र		मापदण्डहरु	प्राप्ताङ्क	उच्चतम अङ्क
३.५.१ पानीको स्रोत	३.५.१	स्वास्थ्य चौकीको लागि खानेपानी र अन्य पानीको (बोरिङ्ग वा ईनार) लगातार उपलब्धता छ ।		१
३.५.२ पानीको भण्डारण	३.५.२.१	पानीको भण्डारण गर्न टैङ्कीको प्रयोग गरिन्छ र संक्रमण रोक्न छोपेर राखिन्छ र नियमित सफा गरिन्छ ।		१
	३.५.२.२	पानीको आपूर्ति रोकिएको खण्डमा कम्तिमा २ दिनको लागि पुग्ने पानीको भण्डारण गर्ने टैङ्की छ ।		१
३.५.३ पानीको गुणस्तर	३.५.३	नेपाल खानेपानी गुणस्तर मापदण्ड २००५ अनुसार प्रत्येक वर्ष पानीको गुणस्तर जाँच गरिन्छ ।		१
मापदण्ड ३.५		कुल प्राप्ताङ्क		४
		प्रतिशत = कुल प्राप्ताङ्क / ४ x १००		

क्षेत्र	कोड	प्रमाणीकरण		
स्वास्थ्यसेवाजन्य फोहोर व्यवस्थापन	३.६			
उपक्षेत्र उपक्षेत्र		मापदण्डहरु	प्राप्ताङ्क	उच्चतम अङ्क
३.६.१ कर्मचारी परिचालन	३.६.१.१	स्वास्थ्यसेवाजन्य फोहोर व्यवस्थापनको लागि कर्मचारीको व्यवस्था गरिएको छ।		१
	३.६.१.२	स्वास्थ्यसेवाजन्य फोहोर व्यवस्थापनसम्वन्धिको तालिम वा अभिमुखिकरण गरिएको छ।		१
३.६.२ ठाऊँ	३.६.२	फोहोर जम्मा गर्न र जोखिमयुक्त फोहोरको अटोक्लेभ गर्नलाई फरक ठाऊँको व्यवस्था छ।		१
३.६.३ फोहोरलाई सुरुदेखि अन्तिमसम्म छुट्याउने	३.६.३	कम्तिमा पनि तीन वटा फरक रङका फोहोर जम्मा गर्ने बाल्टीहरु (एक-एकवटा रातो, निलो र हरियो- HCWM सन् २०१४ को निर्देशिका (स्वास्थ्य तथा जनसंख्या मन्त्रालय) अनुसार) उपलब्ध छन् र प्रयोगमा छन्।		१
३.६.४ व्यक्तिगत सुरक्षा	३.६.४	मास्क, पञ्जा, प्लास्टिकको एप्रोन, बुट र चस्माहरु उपलब्ध छन् र आवश्यकताअनुसार फोहोर जम्मा गर्न प्रयोग गरिन्छ।		१
३.६.५ सार्वजनिक जानकारी	३.६.५.	स्वास्थ्यसम्बन्धीका फोहोर व्यवस्थापनको आधारभूत जानकारी र फोहोर फाल्ने बाल्टीको प्रयोगबारेमा सार्वजनिक जानकारी दिईन्छ।		१
३.६.६ फोहोरको पुनः प्रयोग र व्यवस्थापन	३.६.६.१	संक्रमित फोहोरहरुलाई निर्मलीकरण गरेर मात्र विसर्जन गरिन्छ।		१
	३.६.६.२	पुनः प्रयोग गर्न मिल्ने फोहोरहरुलाई जम्मा गरिन्छ।		१
	३.६.६.३	जैविक फोहोरलाई कम्पोस्ट बनाईन्छ वा वायोग्याँस प्लान्ट बनाईन्छ।		१
	३.६.६.४	नगरपालिका वा गाऊँपालिकाले हानि नगर्ने फोहोर र अटोक्लेभ गरिएको फोहोर लैजान्छ।		१
	३.६.६.५	सालजस्ता मानवीय फोहोरहरुलाई प्लासेन्टा पिट मार्फत सही तरिकाले विसर्जन गरिन्छ।		१
३.६.७ औषधीजन्य फोहोर व्यवस्थापन	३.६.७	औषधीजन्य फोहोर व्यवस्थापन औषधी व्यवस्थापन विभागको निर्देशिकाअनुसार गरिन्छ।		१

३.६.८ झोलजन्य फोहोर व्यवस्थापन	३.६.८.१	झोलजन्य फोहोरको व्यवस्थापन गरिन्छ ।		१
	३.६.८.२	वर्षातको पानीमा स्वास्थ्यसम्बन्धीका फोहोर मिसिएर नजाने व्यवस्था गरिएको छ ।		१
मापदण्ड ३.६		कुल प्राप्ताङ्क		१४
		प्रतिशत = कुल प्राप्ताङ्क / १४ x १००		

**क्षेत्र**	**कोड**	**प्रमाणीकरण**		
**सुरक्षा र संरक्षण**	**३.७**			
**उपक्षेत्र उपक्षेत्र**		**मापदण्डहरु**	**प्राप्ताङ्क**	**उच्चतम अङ्क**
३.७.१ रक्षाकर्मीसँग सहयोग	३.७.१	स्वास्थ्य चौकीले नजिकको प्रहरी कार्यालयसँग समन्वय गर्छ ।		१
३.७.२ सुविधाहरु	३.७.२	सुरक्षा र संरक्षणको लागि आधारभूत सुविधाहरुको व्यवस्था छ । (हेर्नु परिशिष्ट ३.७क सुरक्षा र संरक्षणको लागि आधारभूत सुविधाहरु)		३
३.७.३ बिरामीको सुरक्षा	३.७.३	स्वास्थ्य चौकीले सबै पारोयुक्त उपकरणहरु फेरेर उपयुक्त प्रवधि प्रयोग भएको छ ।		१
३.७.४ प्रकोप न्यूनिकरण व्यवस्था	३.७.४.१	स्वास्थ्य चौकीमा आगो निभाउने यन्त्र र स्याण्ड ब्याग पहुँचयुक्त ठाऊँमा छ ।		१
	३.७.४.३	स्वास्थ्य चौकीमा चट्याङ्गबाट बच्न अर्थिङ्ग व्यवस्था छ ।		१
	३.७.४.४	प्रत्येक कर्मचारीलाई कम्तिमा ६ महिनामा एकपटक प्रकोप न्यूनिकरणको अभिमुखिकरण गरिएको छ ।		१
	३.७.४.५	प्रकोपको बेलामा उम्कनको लागि बाहिर निस्कने चिन्हहरु सबै विभागमा राखिएको छ ।		१
	३.७.४.६	प्रकोपको बेलामा जम्मा हुने ठाँउ (एसम्ब्लि जोन) तोकिएको छ।		१
	३.७.४.७	स्वास्थ्य चौकी र्यापिड रेस्पोन्स टोली बनाइएको छ ।		१
	३.७.४.८	प्रकोप पश्चातको रेस्पोन्स व्यवस्थापनको लागि औषधीहरु उपलब्ध छ ।		१
मापदण्ड ३.७		कुल प्राप्ताङ्क		१२
		प्रतिशत = कुल प्राप्ताङ्क /१२ x १००		

परिशिष्ट ३.७ क सुरक्षा र संरक्षणको लागि आधारभूत सुविधाहरु

क्रमाङ्क	विवरण	आवश्यक संख्या	स्कोर
१	फ्ल्यास लाईट	कम्तिमा १	
२	सिट्टी	कम्तिमा १	
३	महत्वपूर्ण टेलिफोन नंबर	देखिने गरि	
४	इमर्जेन्सी किट \बाकस	कम्तिमा १	
५	आगो नियन्त्रणको उपकरण	कम्तिमा १	
		स्कोर	
		कुल प्रतिशत = कुल अङ्क/५ X १००	

हरेक हरफले १ अङ्क पाउनेछ यदि सबै आवश्यक संख्या छ भने अन्यथा ०

स्कोरिङ्ग तालिका	
कुल प्रतिशत	स्कोर
०-४९	०
५०-६९	१
७०-८४	२
८५-१००	३
मापदण्डको स्कोर ३.७.४	

क्षेत्र	कोड	प्रमाणीकरण		
यातायात साधन र संचार	३.८			
उपक्षेत्र उपक्षेत्र		मापदण्डहरु	प्राप्ताङ्क	उच्चतम अङ्क
३.८.१ यातायात साधन	३.८.१.१	विरामीलाई ल्याउन र लैजानको लागि कम्तिमा एउटा स्ट्रेचरको व्यवस्था छ ।		१
	३.८.१.२	विरामीलाई स्वास्थ्य चौकीमा हिँडाउनकालागि कम्तिमा एउटा व्हीलचियरको व्यवस्था छ ।		१
३.८.२ संचार	३.८.२.१	स्वास्थ्य चौकीमा काम गर्ने टेलिफोन वा मोबाईल छ ।		१
	३.८.२.३	सूचना पाटीको व्यवस्था छ र प्रयोग पनि गरिन्छ ।		१
	३.८.२.४	जरुरी फोन नम्बरहरु (अत्यावश्यक फोन नम्बर, एम्बुलेन्स, वारुणयन्त्र, ब्लड बैँक, प्रशाशन, HFOMC सदस्यहरु, FCHV हरु) OPD, ईमर्जेन्सी र प्रशासनमा राखिएका छन् ।		१
मापदण्ड ३.५		कुल प्राप्ताङ्क		५
		प्रतिशत = कुल प्राप्ताङ्क / ५ x १००		

क्षेत्र	कोड	प्रमाणीकरण		
भण्डार (मेडिकल र उपकरण)	३.९			
उपक्षेत्र उपक्षेत्र		मापदण्डहरु	प्राप्ताङ्क	उच्चतम अङ्क
३.९.१ ठाऊँ	३.९.१	भण्डार (मेडिकल र उपकरण)को लागि फरक ठाऊँको व्यवस्था छ ।		१
३.९.२ मेडिकल स्टोर	३.९.२.१	आवश्यक औषधी र उपकरणहरुको कम्तिमा २ महिनाको स्टक र १ महिनाको ईमर्जेन्सी अर्डरको भण्डारण गरिन्छ ।		१
	३.९.२.२	स्टकहरुको न्यूनतम र उच्चतम परिमाणहरु हिसाब गरिन्छ र फेरि अर्डर गर्दा सोहि आधारमा गरिन्छ ।		१
३.९.३ निशुल्क आवश्यक औषधीहरुको व्यवस्था	३.९.३	निशुल्क आधारभुत औषधीहरुको व्यवस्था बर्षभरिनै उपलब्ध गरिएको छ ।		१

३.९.४ निरिक्षण र स्टक रेकर्डिङ्ग	३.९.४.१	स्टक बुकमा आम्दानी, खर्च, औषधी, खोपहरु र उपकरणहरु रेकर्ड गरिन्छ।		१
	३.९.४.२	स्वास्थ्य चौकीले चौमासिक रिपोर्ट बनाएर स्थानीय सरकारको माध्यमबाट LMIS लाई दिन्छ। (कागजी रिपोर्ट बा विद्युतीय प्रणालीबाट)		१
३.९.५ औषधी र उपकरणको भण्डारण	३.९.५.१	औषधी र उपकरणको भण्डारण चीसो र घामबाट जोगिने ठाऊँ दराजमा गरिन्छ।		१
	३.९.५.२	भण्डरण गरिएको ठाऊँमा हावा खेल्ने ठाऊँ छ।		१
३.९.६ म्याद नाघेका औषधीहरुको व्यवस्थापन	३.९.६	म्याद नाघेका औषधीहरुको व्यवस्थापन स्वास्थ्य मन्त्रालयको HCWM निर्देशिका २०१४ अनुसार प्रत्येक ६ महिनामा गरिन्छ।		१
३.९.७ उपकरणको लिलामी	३.९.७	वार्षिक रुपमा पुराना उपकरणहरुको लिलामी गरिन्छ।		१
मापदण्ड ३.५		कुल प्राप्ताङ्क		१०
		प्रतिशत = कुल प्राप्ताङ्क / १० x १००		

परिशिष्ट १:

सुझाव समिति

क्रमाङ्क	नाम	संस्था
१	केदार बहादुर अधिकारी	तत्कालिन सचिव, स्वास्थ्य तथा जनसंख्या मन्त्रालय
२	डा गुण राज लोहनी	महानिर्देशक, DoHS
३	नारायण ढकाल	महानिर्देशक, DoA
४	डा दिपेन्द्र रमन सिंह	प्रमुख, QSRD
५	महेन्द्र प्रसाद श्रेष्ठ	प्रमुख, HCD
६	डा विकास देवकोटा	प्रमुख, नीति योजना तथा व्यवस्थापन महाशाखा, MOHP
७	डा रमेश खरेल	व. जनस्वास्थ्य प्रशासक, स्वास्थ्य तथा जनसंख्या मन्त्रालय
८	डा मनिषा रावल	निर्देशक, उपचारात्मक सेवा शाखा, DoHS
९	डा मदन कुमार उपाध्याय	प्रमुख मेडिकल सुपरिटेण्डेण्ट, स्वास्थ्य तथा जनसंख्या मन्त्रालय
१०	रोशनि लक्ष्मी टुईटुई	निर्देशक, नर्सिङ तथा सामाजिक सुरक्षा शाखा, DoHS
११	मधुसुदन बुर्लाकोटी	सहसचिव, स्वास्थ्य तथा जनसंख्या मन्त्रालय
१२	सिर्जना श्रेष्ठ	प्रमुख जनस्वास्थ्य प्रशासक, व्यवस्थापन महाशाखा
१३	संगीता शाह	व. औषधि व्यवस्थापक, QARD
१४	विजय क्रान्ति शाक्य	व. जनस्वास्थ्य अधिकृत, QARD
१५	डा. किशोरी महत	सल्लाहकार, गुणस्तर मापन तथा नियमन, NHSSP
१६	डा अर्जुन सापकोटा	उपचारात्मक सेवा शाखा; तत्कालिन व्यवस्थापन शाखा, स्रोतव्यक्ति

पुनरावलोकानकर्ता र योगदान

क्रमाङ्क	नाम	संस्था
१	परशुराम श्रेष्ठ	तत्कालिन बाल स्वास्थ्य शाखा
२	मुक्ति नाथ खनाल	तत्कालिन व्यवस्थापन शाखा
३	रमेश कुमार खत्री	DPHO, ललितपुर
४	दिपक झा	तत्कालिन बाल स्वास्थ्य शाखा
५	ललन प्रसाद साह	तत्कालिन लोजिस्टिक व्यवस्थापन शाखा
६	रेखा साह	ईचङ्गुनारायण स्वास्थ्य चौकीs
७	डा राजेन्द्र भद्रा	ASRH विज्ञ
८	दिपक पौडेल	स्वास्थ्यको लागि सक्षम प्रणाली
९	ढोल बहादुर सिंजाली	चपालीभद्रकाली स्वास्थ्य चौकी
१०	बाल कुमार ओझा	दक्षिणकाली नगरपालिका
११	भोकन ढकाल	चिठुविहार स्वास्थ्य चौकी
१२	ग्यानेन्द्र चन्द	चल्नाखेल प्राथमिक उपचार केन्द्र
१३	रोमा बलामी	SIFPO-२/ FPAN
१४	हिमाल सिंह ठकुरी	गोदामचौर स्वास्थ्य चौकी
१५	सुदर्शन बजगाई	गोदावरी नगरपालिका
१६	डा शिलु अधिकारी	USAID
१७	डा माउरिन डार लाङ	DFID/NHSSP
१८	डा किशोरी महत	DFID/NHSSP
१९	डा रोसनी अमात्य	Jhpiego
२०	कृष्ण कुमार खत्री	
२१	डा बिना गुरुङ	SIFPO-२/ MSI
२२	डा सुमन पन्त	SIFPO-२/ MSI
२३	मदनराज भट्ट	SIFPO-२/ FPN
२४	किमत अधिकारी	WHO
२५	अन्जना राई	Consultant MSS, MD, DoHS
२६	अम्बिका थापा पाच्या	MSS का लागि प्राविधिक संयोजक, WHO नेपाल /NSI / NHSSP
