# Supplementary material for: Minimum service standards assessment tool and the hospital strengthening program: a novel first step towards the quality improvement of Nepal’s national hospital system
Source: Lancet Reg Health Southeast Asia. 2025 Feb 22;34:100548. doi: 10.1016/j.lansea.2025.100548 (PMC11904555; doi:10.1016/j.lansea.2025.100548)
Supplement: MSS_Tertiary Hospitals Assessment [file mmc5.pdf]

Minimum Service Standards (MSS)

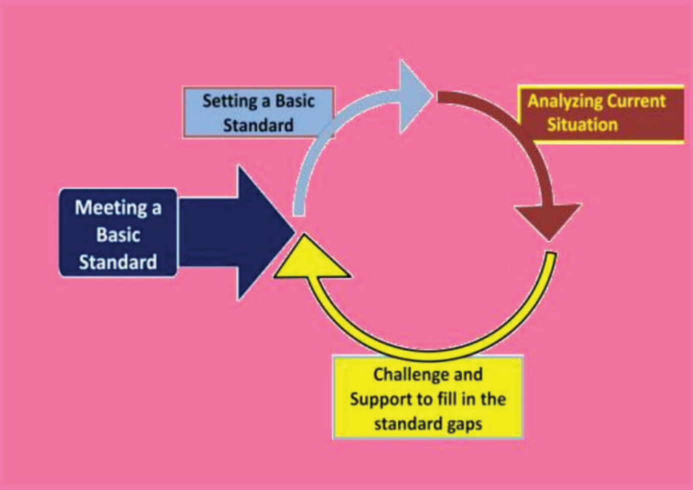

Checklist to Identify the Gaps in Quality Improvement of Tertiary Hospitals

Government of Nepal
Ministry of Health and Population
Quality Standards and Regulation Division

Government of Nepal
Ministry of Health and Population

Phone: 4. 262987 / 262590 / 262802 / 262706 / 262935 / 262862

Ramshahpath, Kathmandu
Nepal

Ref.: ....

Government of Nepal
Ministry of Health and Population
Ramshah Path, Kathmandu

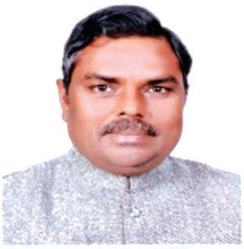

MESSAGE

The Constitution of Nepal 2072 has promulgated health as a fundamental human right. The Ministry of Health and Population (MoHP) is responsible for overall policy formulation, planning, organization and coordination in the health sector. The National Health Policy 2014, the Nepal Health Sector Strategy 2015-2020 and the Public Health Service Act 2018 provisions for quality health care delivery.

Ensuring quality health care is a major concern for the MoHP. Various initiatives have been implemented in the past to improve quality of care. The Minimum Service Standards (MSS), a self-assessment tool for hospitals will be an effective tool to ensure the enabling environment for both service providers and service users to achieve the goal of quality health care for all. The MSS focuses on the holistic aspect of hospital strengthening through improved Governance and Management, Clinical Service Management and Hospital Support Services. The MoHP in coordination with Provincial and Local governments including partners will implement MSS across all levels of health facilities for ensuring quality of health care to all citizens. The implementation of MSS demands integrated planning and budgeting in the components demanded for any service provision.

I acknowledge the leading role of Quality Assessment and Regulation Division in development of the MSS and the support provided by the partners.The MoHP remains committed in leading the successful implementation of MSS throughout the hospitals in the country.

....

Upendra Yadav
Deputy Prime Minister and
Minister of Health and Population

Date : 2075,Paush

Government of Nepal
Ministry of Health and Population

Phone: 4. 262987, 262590, 262802, 262706, 262935, 262862

Ramshahpath, Kathmandu
Nepal

Ref.: ....

Government of Nepal
Ministry of Health and Population
Ramshah Path, Kathmandu

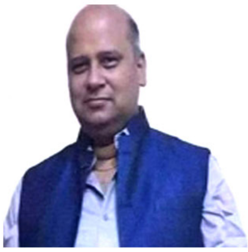

MESSAGE

The Constitution of Nepal 2015 has promulgated health as a fundamental human right. The Nepal Health Sector Strategy 2015-2020 is the guiding document for the health sector and emphasizes quality as an integral component of health service. The Public Health Service Act 2018 has also provisions of quality health care including delivery of quality Basic Health Care Services to all.

For the Ministry of Health and Population (MoHP), it is therefore essential to have the readiness standards to ensure availability for quality health care services at the point of delivery. The Minimum Service Standards (MSS) for health facilities has been instrumental in this regard. Since 2014, the MSS has been successfully implemented in all districts level hospitals which is now being planned for all levels of health facilities. Therefore, the updated standards for Primary hospitals and development of MSS for Secondary and Tertiary Hospitals will be helpful to improve the readiness for quality health services at all levels of health care. The MSS will also be an important monitoring tool for all three levels of government to plan and budget accordingly for improvement in quality of care.

I acknowledge the leading role of Quality Assessment and Regulation Division of MoHP and support of all the partners in developing the MSS. Implementing MSS and continuous monitoring will help to ensure enabling environment for quality service provision. Therefore, expansion of MSS at all levels of hospital is a priority of MoHP and I look forward for the continued support and commitment of partners in implementing the MSS.

....

Hon. Dr. Surendra Kumar Yadav
State Minister
Ministryof Health and Population

Date:2075,Paush

Government of Nepal
Ministry of Health and Population
Phone: 4. 262987 262590 262802 262706 262935 262862
Ramshahpath, Kathmandu
Nepal

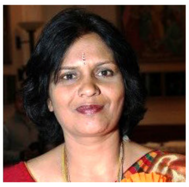

PREAMBLE

In line with the National Health Policy 2014, the Nepal Health Sector Strategy 2015-20 and its implementation plan and the Public Health Service Act 2018; the Minimum Service Standards (MSS) for Hospitals will be a milestone in improving the readiness of the hospitals to deliver quality health care service. The MSS tries to bring together the existing standards, protocols, guidelines and tools into a comprehensive document for assessment of all levels of hospitals in order to improve quality of care. The self-assessment through MSS provides information on existing gaps to be addressed in order to prepare the hospitals to deliver quality health services. It will also serve as a continuous monitoring tool for all levels of government which is crucial in ensuring quality health care in hospitals.

The standards are organized in three aspects, namely; Governance and Management, Clinical Service Management and Hospital Support Services. I hope our hospital management committees, medical superintendents, related staffs and partner organizations will use these standards for strengthening hospitals in order to improve the overall quality of care.

The contribution of Dr Dipendra Raman Singh, Chief Quality Assessment and Regulation Division and his team, and all staff involved in developing this important document is noteworthy. I would also like to acknowledge the support of the partners involved in finalizing these standards, particularly the Nick Simon's Institute, the DFID/Nepal Health Sector Support Program, and WHO Nepal for their constant effort to bring this document into its current form. I look forward for the successful implementation of MSS throughout the hospitals in the country.

Dr. Pushpa Chaudhary
Secretary

Date: 2075, Paush

Government of Nepal
Ministry of Health and Population
Phone: 4. 262987 262590 262802 262706 262935 262862
Ramshahpath, Kathmandu
Nepal

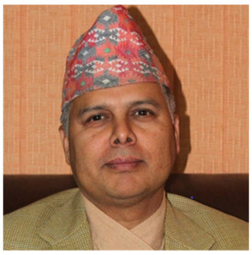

PREFACE

The National Health Policy, the Nepal Health Sector Strategy 2015-20 and the Public Health Service Act emphasizes delivery of quality health care. The learnings from the implementation of MSS since 2014 at district level hospitals have been encouraging and therefore, Ministry of Health and Population (MoHP) was committed to develop MSS for all levels of hospitals. The development of the MSS for hospitals has been done through extensive consultations with MSS implementing hospitals, experts and partners. I hope that implementation of MSS will not only help to strengthen the hospital management but will also be an effective tool for monitoring the hospitals by each level of government.

The implementation of MSS will take into account evidence based planning and budgeting for the identified gaps of the hospitals. These planned investments must be aligned with each other to make the hospitals ready for the services from holistic dimension of governance, clinical service and support service management. The MoHP stays committed in supporting hospitals as relevant in addressing the gap areas and urges the financial and technical support from all level of the government. And being a rolling document the revisions and update will be regulated as per need.

I extend my heartfelt gratitude to the contribution of Dr. Dipendra Raman Singh, Chief Quality Assessment and Regulation Division and his team, provincial health directorate and all the other divisions of MoHP and DoHS for their contribution. I would also like to acknowledge the contribution of experts and partners in supporting MoHP in developing this document and hope for the continued support of all in the implementation of MSS.

KAdhikari

Date:2075,Paush

Mr. Kedar Bahadur Adhikari
Secretary
Ministry of Health and Population

Government of Nepal
Ministry of Health and Population

Phone: 4. 262987, 262590, 262802, 262706, 262935, 262862

Ramshahpath, Kathmandu
Nepal

Ref.: ....

Government of Nepal
Ministry of Health and Population
Ramshah Path, Kathmandu

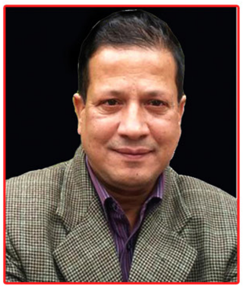

FOREWORD

The Minimum Service Standards for Hospitals is a milestone for overall health system that sets in the basics for the readiness and availability of services claimed to provide. The MSS tries to bring together the existing, guidelines, tools, standards, protocols and advocates for readiness in order to improve quality care in the hospitals and thus utilization of its services.

The Minimum Service Standards (MSS) for hospitals will be an important milestone for strengthening the hospital management and health services to realize the policy commitments of Ministry of Health and Population to ensure quality health care for all. The MSS for primary hospital is the revision of the MSS for District level hospitals, while those of Secondary and Tertiary have been developed in line with the commitment in the Nepal Health Sector Strategy 2015-20.

Minimum Service Standards is a self-assessment tool to identify the readiness gaps to deliver quality health services. Following the identification of gap the action plans is developed by the hospitals for improvement. Thus developed action plans demand both financial and technical support and require proper planning and budgeting. The investments made need to be integrated to see the overall dimensions of management as structured in MSS including the Governance and Management, Clinical Service Management and Hospital Support Services.

I would like to acknowledge the contribution of Chief Quality Assessment and Regulation Division, the members of the Technical Working Group, subject experts, reviewers and technical coordinator in developing this important document. I look forward for the successful implementation of MSS throughout the hospitals in the country and hope that the learnings will be documented to inform the revision of MSS in due course of time.

Date:2075,Paush

Dr. Sushil Nath Pyakurel
Chief Specialist
Ministry of Health and Population

Government of Nepal
Ministry of Health & Population
DEPARTMENT OF HEALTH SERVICES
(...)

Tel. : 4261436
: 4261712
Fax : 4262238

Pachali, Teku
Kathmandu, Nepal

Ref. No.

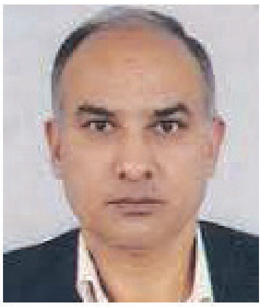

PREFACE

The Minimum Service Standards for Hospitals was pioneered in 2014 as Minimum Service Standards for District Hospital under the Hospital Management Strengthening Program of Ministry of Health and Population (MoHP) with support from Nick Simons Institute. During inception of Minimum Service Standards (MSS), it was taken up as a living document which will be updated and revised after being rolled out nationwide, till date the MSS for District Hospitals has been rolled successfully in 83 district level hospitals. With the revision envisioned from the very beginning, the available learnings from the implementation and the federal context, the MSS for District Hospitals has been given the present shape of MSS for Primary Hospitals, while MSS for Secondary and Tertiary Hospitals have been developed.

As a person involved since the inception of the Minimum Service Standards, I can see the positive changes that MSS has brought which is visible in the MSS implementing hospitals. The change was possible only through evidence informed action plans developed from assessments which were supported by holistic dimensions of governance, clinical services and support services management covered by budgeting and regular follow up. In the development of MSS these learnings have been incorporated through robust consultations with implementing hospitals, subject experts and Technical Working Group members. Their contribution in bringing the document to its current shape is admirable. MSS is based on the principle of evidence-based activities in itself, it will be revised as per required updates.

I would like to thank Chief of Quality Assessment and Regulation Division, all the other departments of DoHS who contributed their technical expertise in developing this document. I also acknowledge the support of partners particularly, the Nick Simons Institute, DFID/ Nepal Health Sector Support Program and WHO Nepal for their assistance in this important undertaking and look forward for their continued support in implementing the MSS at all levels of Hospitals.

....
Dr. Guna Raj Lohani
Director General
Department of Health Services

Date:2075,Paush

Executive Summary

Minimum Service Standards (MSS) for hospitals is the service readiness and availability of tool for optimal requirement of the hospitals to provide minimum services that are expected from them. This tool entails for preparation of service provision and elements of service utilization that are deterministic towards functionality of hospital to enable working environment for providers and provide resources for quality health service provision. MSS for hospitals reflect the optimally needed minimum criteria for services to be provide but in itself is not an "ideal" list of the maximum standards. This checklist of MSS is different than a program specific quality improvement tool as it will outline the equipment, supplies, furniture, human resource required for carrying out service but not detail out the standards operating procedures of any service.

The results of Nepal Health Facility Survey 2015 showed that among the health facilities that were assessed only 13 percent of them had all seven basic equipment items- adult weighing scale, child weighing scale, infant weighing scale, thermometer, stethoscope, blood pressure apparatus and a light source for service provision. The availability of all supplies and equipments defined for standard precaution control was as low as 0.2%, all basic laboratory services in 12% and only 3% facilities had client feedback mechanism in place. This was an alarming situation. During that period, minimum service standards was rolled out in 83 district level hospitals and was evident to contribute in quality of services provided by hospitals with instances of improved governance, management, clinical and support services. This encouraged MoHP to put on its efforts on setting the minimum service standards for hospitals secondary and tertiary levels and at the same time contextual revision of MSS for district hospitals to set MSS for primary level hospitals. The revision and development of the tool took into series of steps beginning with formulation of Technical Working Group and selection of subject experts and technical coordinator and consultative workshops and meetings (Figure: Process of MSS revision and development). The key guiding documents are Constitution of Nepal 2072, National Health Policy 2014, Policy on Quality Assurance in Health Care Services, 2064, Public Health Service Act 2075, Nepal Integrated Health Infrastructure Development Standards 2073/74, Nepal Health Sector Strategy 2015-2020 and Guideline on Health Institution Establishment, Operation and Upgrading Standards, 2070 but not limited to them.

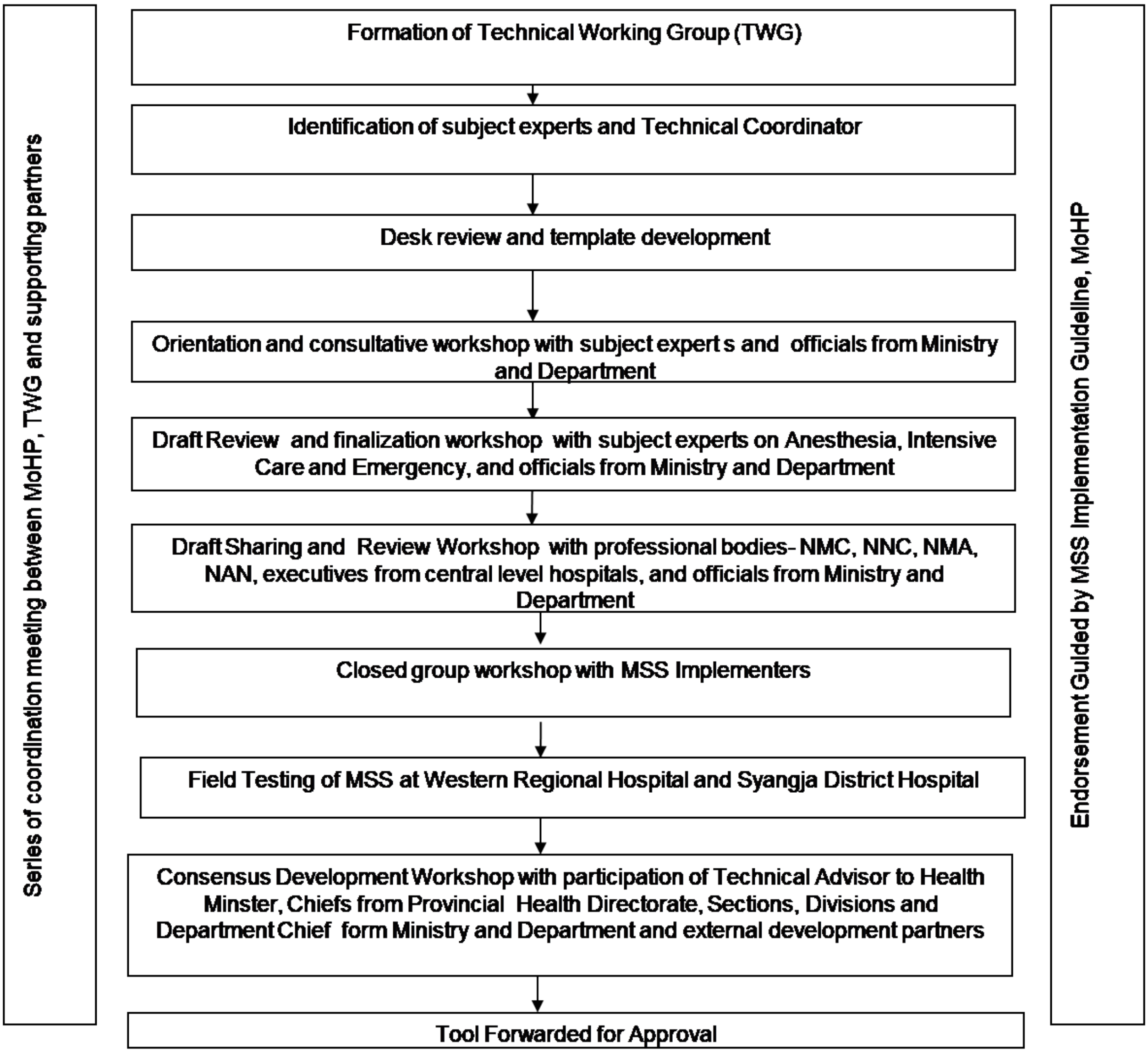

Figure Process of Revision and Development of Minimum Service Standards for Hospitals

Thus prepared MSS is a comprehensive tool for optimal preparation of the hospitals for the minimum services that are needed to be provided by these health facilities and has potential to bring a positive change. The health sector needs are dynamic and revision of the services and standards in due course is anticipated. The revision of MSS for hospitals is planned to be done every 2-3 years (completion of cycle of MSS in all targeted government hospitals) to incorporate the learning and adapt the documents to the emerging context.

The MSS tool has been organized in three major sections: Governance and Management, Clinical Service Management and Hospital Support Service Management. It has been prepared in the form of checklist that thrives for the preparedness and utilization that are fundamental to establish services towards quality. For tertiary hospitals, there are 1084 set of standards with total score of 1406, out of which- 106 standards for measuring governance and management and has weightage of 20%, 840 standards for measuring clinical service management and has weightage of 60%, and 138 standards for measuring support service management and has weightage of 20%. Governance and management section includes the minimum standards for six subsections, clinical service management has twenty one sub sections and hospital support service management has eleven subsections.

After assessment of all the sections of the standards, for overall scoring, each section is then weighed. The section of the governance and management (Section I) is weighed in 20%, that of clinical service management (Section II) is weighed in 60% and that of hospital support service management (Section III) is weighed in

20%. The sum of these weighed percentage of the subsections give the overall MSS score of the hospitals and based on it color code will be provided. This MSS Score for hospitals measure the existing situation and enables to identify the gap areas that are to be addressed through the development of the actions plan that demands both technical and financial inputs and managerial commitments. The overall process is guided by its implementation guideline that describes on sequences of self assessment and follow up workshops and gap identification for action plan development and striving for optimal MSS Score.

Ministry of Health and Population strives to implement MSS in hospitals for establishing enabling environment at service delivery point through preparedness and availability for quality service provision to the users. Not being an exhaustive list of facilities and services, hospitals are encouraged to strive for betterment and go beyond the defined set of minimum standards whenever their resources support.

Table of Contents

Executive Summary vii

Hospital Identification Sheet xiii

Background 1

Introduction 1

- Process of Minimum Service Standards Development 3
- Organization of the Standards 6
- How to use this Checklist? 7

Section I: Governance and Management Standards 10

- Summary Sheet for Standards and Scores 10
- Governance 11
- Organizational Management 12
- Human Resource Management and Development 13
- Financial management 18
- Medical Records and Information Management 19
- Quality Management 20

Section II: Clinical Service Management 22

- Summary Sheet for Standards and Scores 22
- OPD Service 23
- Special Clinics 36
- Emergency Service 43
- Emergency Minor OT 52
- Hospital Pharmacy Service 62
- Inpatient Service 64
- Maternity Services 78
- Surgery / Operation Services 97
- Hemodialysis service 117
- Intensive Care Services 123
- Diagnostics and laboratory 132
- Postmortem 154
- Medico-legal services 156
- One stop crisis management center 158
- Physiotherapy (Physical Rehabilitation) 162

Dietetics and Nutrition rehabilitation 165
Cardiac catheterization Laboratory 166
Dietetics and Nutrition rehabilitation 200
Section III: Hospital Support Services Standards 208
Summary Sheet for Standards and Scores 208
CSSD 209
Laundry 211
Housekeeping 213
Repair, Maintenance and Power system 215
Water supply 216
Hospital Waste Management 216
Safety and Security 217
Transportation and Communication 219
Store (Medical and Logistics) 219
Hospital Canteen and Dietetics 220
Social Service Unit 221
Annex I: List of Subject Experts 223
Annex II: List of Reviewers 224

Hospital Identification Sheet

Name of the Hospital	
Assessment Date	
Assessed By	1.
	2.
	3.
Score of Section I: Governance and Management	
Score of Section II: Clinical Service Management	
Score of Section III: Hospital Support Service Management	
Overall MSS Score	
MSS Score Color Category	

Background

Introduction

Minimum Service Standards (MSS) for hospitals is the service readiness and availability of tool for optimal requirement of the hospitals to provide minimum services that are expected from them. This tool entails for preparation of service provision and elements of service utilization that are deterministic towards functionality of hospital to enable working environment for providers and provide resources for quality health service provision. MSS for hospitals reflect the optimally needed minimum criteria for services to be provide but in itself is not an “ideal” list of the maximum standards. This checklist of MSS is different than a program specific quality improvement tool as it will outline the equipment, supplies, furniture, human resource required for carrying out service but not detail out the standards operating procedures of any service.

The results of Nepal Health Facility Survey 2015 showed that among the health facilities that were assessed only 13 percent of them had all seven basic equipment items- adult weighing scale, child weighing scale, infant weighing scale, thermometer, stethoscope, blood pressure apparatus and a light source for service provision. The inception of minimum service standards started with the realization to improve the curative services in rural hospitals with focus on hospital management. It began as piloting in 4-hospital in 2013 in partnership with Nick Simons Institute to support district hospital for assessment of minimum service standards using “MSS Checklist to Identify the Gaps in Quality Improvement of District Hospitals”. This has been rolled out successfully nationwide in 83 district level hospitals.

The availability of all supplies and equipments defined for standard precaution control was as low as 0.2%, all basic laboratory services in 12% and only 3% facilities had client feedback mechanism in place. This was an alarming situation. During that period, minimum service standards was rolled out in 83 district level hospitals and was evident to contribute in quality of services provided by hospitals with instances of improved governance, management, clinical and support services. This encouraged MoHP to put on its efforts on minimum service standards for hospitals.

Following the learning from the implementation of MSS and considering the current Federal context, MoHP has updated MSS for the district hospitals to make it applicable to Primary Hospitals. At the same time, MoHP has also developed the MSS for secondary and tertiary hospitals in line with the intervention planned in the NHSS-Implementation plan (2016-2021).

In developing the Minimum Service Standards for hospitals, following documents were key references:

1. National Health Policy 2071
2. Policy on Quality Assurance in Health Care Services, 2064
3. Public Health Service Act, 2075
4. Governance (Management and Operation) Act, 2064
5. Financial Procedure Regulation, 2064
6. Nepal Health Service Regulation, 2055
7. Civil Service Regulation, 2050
8. Basic Health Service Package 2075. Ministry of Health and Population, GoN
9. Nepal Health Sector strategy 2015 -2020. Ministry of Health and Population, GoN.
10. Nepal Integrated Health Infrastructure Development Standards 2073/74
11. Quality Improvement Tool for Health Facility, 2074

12. Guideline on Health Laboratory Establishment and Operation Standards, 2073

13. Implementation Guideline for Social Audit in Health Sector, 2070 Revised 2073

14. Hospital Pharmacy Service Guideline, 2070 Amended 2072

15. National List of Essential medicines 2066/67 Revised 2072/73

16. Minimum Service Standards (MSS) Checklist to Identify the Gaps in Quality Improvement of District Hospitals, Curative Service Division, MoHP, GoN, 2071/72

17. Guideline on Health Institution Establishment, Operation and Upgrading Standards, 2070

18. Transaction Accounting and Budget Control System (TABUCS) Users' Guide, 2070

19. Guideline for Heath Management Information System, Recording and Reporting, 2070

20. Job Description of Staffs of Regional Health Directorate and District Health and Public Health Offices, 2070

21. Operational Procedure of Department of Health Services, 2068

22. Implementation Guideline Quality Improvement of Health Services, 2066

23. Infection Prevention Reference Manual (District Hospital and Health Facility), 2066

24. National Medical Standard for Reproductive Health, Vol. III, 2063/64

25. National Safe Motherhood and Newborn Health Program District Maternal and Neonatal Health Need Assessment Toolkit Vo. 1, Hospital, 2063/64

26. Health care waste management guideline, 2014. Ministry of Health and Population. Government of Nepal

27. Hospital management practices observed in 83 District and upgraded hospitals.

28. Series of consultation Workshops, Technical Working Group meetings and consultation of subject experts.

Besides these, literature related to health facility readiness and availability, and quality of care were visited by the consultant, technical coordinator, subject experts and TWG members and contextual reality were given priority during the development of the MSS tools.

The Minimum Service Standards have been defined considering the existing organizational structure of the hospitals, provision of human resources and financing capacity. The defined standards basically focus on the readiness of hospitals towards ensuring the delivery of quality health services. However, hospitals are encouraged to strive for betterment and go beyond the defined set of minimum standards whenever their resources support. The health sector needs are dynamic and revision of the services and standards in due course is anticipated. The revision of MSS for hospitals is planned to be done every 2-3 years (completion of cycle of MSS in all targeted government hospitals) to incorporate the learning and adapt the documents to the emerging context.

Process of Minimum Service Standards Development

Formation of Technical Working Group (TWG)

A Technical Working Group was formed by MoHP to develop the Minimum Service Standards (MSS) for Hospitals with involvement of the then curative service division, related sections and departments and experts from the external development partners.

The TWG comprised of the following members as per previous MOHP structure and the members continued till finalization of the document:

- Chief, The then Curative Service Division, MoHP- Coordinator
- Chief, Public Health Administration, The then Monitoring and Evaluation Division, MoHP- Member
- Medical Generalist, MoHP- Member
- Executive Director, Health Insurance Board- Member
- Chief, The then Nursing Section, MoHP- Member
- Under Secretary, The then Curative Service Division, MoHP- Member
- Chief, The then Quality Section of Management Division- Member
- Representative, World Health Organization, Nepal- Member
- Representative, DFID/Nepal Health Sector Support Program- Member
- Representative, Nick Simons Institute- Member
- Section Chief, The then Curative Service Division, MoHP- Member Secretary

Identification of subject experts and technical coordinator

As per the agreement of the TWG members and the learning from MSS implementation in district hospitals, it was agreed to divide the MSS into three sections namely; Governance and Management, Clinical Service Management and Hospital Support Service Management. With series of meetings among the TWG members, subject experts (Annex I- List of Subject Experts) were identified for consultation in specific sections of the service standards under the leadership of the then Curative Service Division. They also developed term of reference for technical coordinator who would be a liaison among MoHP, partners and subject experts for the persecution of the task.

Desk review and template development

Technical coordinator (TC) for the work supported TWG for the development of the templates for revision of the minimum service standards for primary, secondary and tertiary hospitals with desk review of the key guiding documents (as listed in background) and service availability and readiness tool of World Health Organization (WHO). Thus developed templates were shared with the subject experts. All the subject experts were coordinated by TC for preparing the zero draft of the MSS and TWG moved on to the next step of consultative workshops.

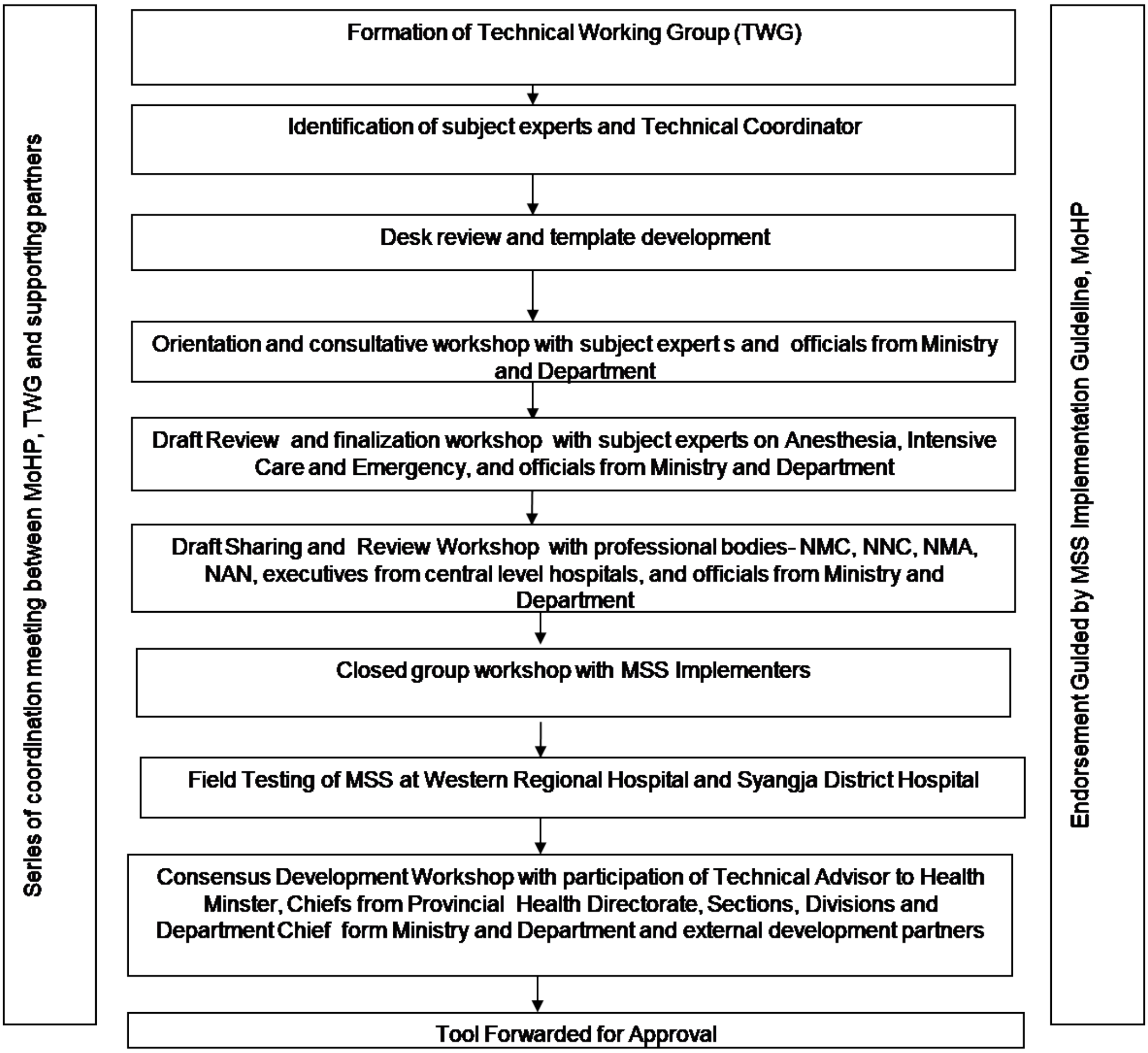

Figure Process of Revision and Development of Minimum Service Standards for Hospitals

Series of coordination meeting between MoHP, TWG and supporting partners

Series of coordination meeting were conducted by MoHP for the discussion of action points with TWG members and supporting partners for MSS. Being based on identified need the consultative workshops, their modality and participants were finalized.

Orientation and consultative workshop

For orientation and consultative workshop, participants were subject experts and consultant doctors, government officials from department, sections and divisions of MoHP, representatives from various academic institutions, different regional, zonal and district level hospitals and partner agencies. There were around 62 participants in the workshop addressed by Health Secretary, MoHP. Subject experts from tertiary center, regional, zonal and district hospitals of the country shared their valuable experience of understating and working for minimum service standards while being part of the district level hospitals during their service. From the workshop, revision of MSS for primary level and development of MSS for secondary and tertiary hospitals was guided to be service oriented leading to a comprehensive practical tool for institutionalizing readiness and service availability in the hospitals. Technical coordinator shared the draft one with the TWG team and critical areas for intensive work out identified were- intensive care units, anesthesia, emergency and public health perspective. Being based on the critical areas identified, modality for further steps was contextualized and Review Workshop I was planned with experts for working in identified critical areas.

Draft review and finalization workshop

For review and finalization workshop, there were around 25 participants including members of TWG, government officials from the then Curative Service Division, Management Division, subject experts on intensive care, anesthesia and emergency services from academic institutions, professional bodies and hospitals, and experts from partner agencies. The inputs from the workshop on was on format of tool to arrange the supporting annex immediately followed by standards and sequence of the annexes used along with standards to be uniformly allocated as furniture, equipment, instrument and supplies for making it user friendly. The technical inputs were received in all three thematic areas with special inputs on intensive care, anesthesia and emergency services; also contextualization of the ratio of health workforce for services was done during the workshop. The feedback were incorporated by TC to develop draft second of the MSS. Technical coordinator shared the draft second with the TWG team and draft sharing review workshop II with the professional councils and associations, representatives from central level hospital was planned.

Draft sharing and review workshop

For draft sharing and review workshop, there were around 35 participants who were representatives from Nepal Medical Council, Nepal Nursing Council, executives from central level hospitals, representatives from Nepal Medical Association, Nepal Nursing Association and Society of Public Health Physician Nepal. It was identified during the workshop that there is requirement of division of the clinical service components of primary hospitals at least into two sections of less than 50 beds and 50 beds to 100 beds for the ease of the relevancy of the services available. Also final format for the tool was decided to be addressing- area, its component, verification having the service standards. Being based on the inputs from workshop, separate MSS for primary hospitals with general service and primary hospitals with specialized services were drafted. And additional consultation was done with identified nephrologist, neurologist, gastrologist and burn/plastic surgeon for additional services in tertiary hospitals who were consulted by TC for technical inputs and thus draft third of the MSS was prepared. Closed group workshop with MSS implementers for proof reading and appraisal of practical aspects of the MSS was planned.

Closed group workshop with MSS Implementers

For closed group workshop with MSS implementers, participants were members of TWG, team of implementers working as district health support program officers of partner organization who were working closely with the district hospitals for MSS implementation. In the workshop, each of the standards were reviewed and their practical experiences were shared and documented. The tool was assessed for user-friendliness and inputs were incorporated related to directions of use of the tool was added in places where there were checklists and annexes followed by the standards. Feedback were incorporated by TC to develop draft fourth of the MSS which was presented to TWG. This draft was then used for field testing.

Field Testing of MSS

For field testing, the working team was composed of TWG members and coordinated by TC. Field testing was done in Western Regional Hospital, Pokhara and Syangja District Hospital. The hospital staffs of both the hospitals were given brief presentation by TWG member and further facilitated with help of Hospital Director at Western Regional Hospital and Medical Superintendent at Syangja District Hospital respectively. The department/unit heads were consulted and the tool was thoroughly read by them and marked as agreeable to them for assessing the readiness and availability of any services they are giving and asked about its practicable aspects. After incorporation of feedback, final draft were shared by TC with TWG and further shared with sections and department chiefs of MoHP and a consensus development workshop was planned.

Consensus Development Workshop

For consensus development workshop, the working team was composed of TWG members, TC and coordinated by Quality Assurance and Regulation Division (QARD). There were around 35 participants representing the different sections, Department of Health Service, Department of Drug Administration, Department of Ayurveda, and divisions of MoHP, representatives from province health directorate and representatives of partner organization. Consensus was developed in most of the content of the MSS and its implementation guideline was recommended to be in line with the federal context. Feedback received was focused on the practical aspect of the MSS and it was put forward as paving the road towards quality of care. After incorporation of feedback, final tools were shared by TC with TWG and further shared to Health Secretary of MoHP for further approval.

After approval from MoHP, the implementation of the MSS will be guided by "Minimum Service Standards Implementation Guideline 2075".

Organization of the Standards

The overall service standards are categorized in three major sections: governance and management, clinical service management and hospital support service management. There are total 1085 set of standards with **total score** of 1407; with 107 standards for measuring governance and management and score of 111 which has weightage of 20%, 840 standards for measuring clinical service management and score of 1148 which has weightage of 60%, and 138 standards for measuring support service management and score of 148 which has weightage of 20%.

Section I: Governance and management

Strengthening Governance and Management is a key to provide the enabling environment in hospitals for service providers and users. This section includes the minimum standards for the following subsections:

1.1. Governance

1.2. Organizational management

1.3. HR management and development

1.4. Financial management

1.5. Medical records and information management

1.6. Quality Improvement

Section II: Clinical Service Management

In order to ensure quality of care at the point of delivery, the implementation of clinical service standards is essential. This section comprises of minimum standards under the following sub sections:

2.1. Outpatient services (General Medicine, Obstetrics/Gynecology, Pediatrics, Surgery, Dental, Orthopedics, Pyschiatry, Ear, Neck and Throat (ENT), Cardiology, Dermatology, Urology, Neurosurgery, Neurology, Burn/Plastic, Nephrology, Gastrology)

2.2. Special Clinics (Immunization and growth monitoring; family planning; Anti-tubercular treatment (ATT), anti-retroviral treatment (ART), Safe Abortion Services)

2.3 Emergency Service

2.4 Emergency Minor OT

2.5 Hospital Pharmacy Service

2.6 Inpatient Service (Medicine Ward, Surgery Ward, Pediatrics Ward, Orthopedics Ward, Psychiatry Ward, ENT Ward, PNC and Gynecology Ward, Burn Ward and Geriatrics Ward)

2.7 Maternity Services (Delivery Service, Maternity Inpatient Ward)

2.8 Surgery/ Operation Service

2.9 Diagnostics and investigations (Laboratory, blood bank, X-ray, USG, ECG, Echocardiogram, Treadmill Test, CT Scan, Endoscopy, Audiometry)

2.10 Hemodailysis

2.11 Intensive Care Services- ICU, PICU, NICU

2.12.1 Postmortem Services

2.12.2 Medico-legal Services

2.13 One Stop Crisis Management Center (OCMC) Services

2.14 Physiotherapy

2.15 Nutritional Rehabilitation and Dietetics

2.16 Cardiac Catheterization Lab

Section III: Hospital Support Service Management

Implementing the standards for Hospital Support Services is equally important to avail and support delivery of quality clinical services. This section includes minimum standards under the following subsections:

3.1. CSSD

3.2. Laundry

3.3. Housekeeping

3.4. Repair and maintenance, power system

3.5. Water supply

3.6. Hospital Waste Management

3.7. Safety and Security

3.8. Transportation and Communication

3.9. Store (Medical and Logistics)

3.10. Hospital Canteen

3.11. Social service unit

How to use this Checklist?

The MSS for hospitals is a self- assessment tool. Each standard has set of dimensions with one or more verification criteria which is assessed. The checklist enables hospitals to measure the existing situation in through scoring and helps to identify the gap areas to be addressed through the development of the actions plans. This is a cyclical process the details of which is in the implementation guideline. The key steps are as follows:

Group discussion

- Conduct a group discussion in your hospital to see if the hospital really meets the given standards under each section.

Filling the checklist

- Read each section carefully and if your hospital meets the given standards, please score from 0 to 3 in the column of the score based on the maximum score for that standard
- For areas where there is indication of checking annex, please calculate the percentage and follow the scoring chart for scoring from 0 to 3
- For areas where there is indication of checking the checklist, please use both the standard and checklist for getting the clear picture of that standard
- Please use individual copies for each department/unit wherever applicable so that there is least biasness in the assessment
- Complete this process for all the standards

Scoring the checklist

- In each sub-section, add the **total score** and convert it into percentage.
- Calculate the average of the percentages obtained in its sub-section to obtain the score of a section
- Do this to all three sections to obtain the scores of each section and then take the weightage for calculating the overall MSS score of the hospital

Sample of filling the tool

Area	Code	Verification		
**CSSD**	**3.1**			
**Components**		**Service Standards**	**Obtained Score**	**Maximum Score**
3.1.1 Space	3.1.1.1	Separate central supply sterile department (CSSD) is available. with running water facility	1	1
	3.1.1.2	There are separate rooms/ space allocated for dirty and clean utility.	1	1
3.1.2 Staffing	3.1.2	Separate staffs assigned for CSSD under leadership of trained nursing staff	1	1
3.1.3 Equipment and supplies for CSSD	3.1.3	Equipment and supplies for sterilization available and functional round the clock (See Annex 3.1a CSSD Equipment and Supplies At the end of this standard)	2	3
3.1.4 Preparing consumables	3.1.4	Wrapper, gauze, cotton balls, bandages are prepared.	1	1
Standard 3.1		**Total Score**	6	7
		**Percentage = Total Score / 7 x 100= 6/7x100**	85.7%	

Annex3.1a CSSD Equipment and Supplies

SN	Items	Required No.	Score
1.	Working Table	3	1
2.	Trolley for Transportation	2	0
3.	Steel Drums	10	0
4.	Autoclave Machine (250 liter, pre-vacuum, with horizontal outlet)	2	1
		**Total Score**	3
		**Total Percentage = Total Score/4 X 100**	75

Each row gets a score of 1 if all the required number is available otherwise 0.

**Scoring Chart**	
**Total Percentage**	**Score**
0-50	0
50-70	1
70-85	2
85-100	3
**Score for Standard 3.1.3**	2

Weightage of the sections and Overall MSS Score

After assessment of all the sections of the standards, for overall scoring, each section is then weighed. The section of the governance and management (Section I) is weighed in 20%, that of clinical service management (Section II) is weighed in 60% and that of hospital support service management (Section III) is weighed in 20%. For example:

If Section I has the overall score of 80%, Section II has 60% and Section III has 80%; the overall score of the hospital for MSS assessment is calculated as:

Overall MSS Score = (0.2xSectionI + 0.6X Section II + 0.2x Section III)%

Overall MSS Score = (0.2x80%+ 0.6x60%+0.2x 80%)

Overall MSS Score= 68%

Interpretation of the MSS scores

The overall idea of the MSS score is that it is the minimally required optimal readiness of the hospitals to provide the available services. And moving towards the obtaining 100% in all individual sub-sections and overall MSS score is the requirement to thrive a step ahead towards quality service provision. The scores for any sub-sections being less than fifty (<50) is taken as very poor and alarming and needs to be addressed first. The scores from fifty to less than seventy (50 to<70) are taken as the state of improving status that needs specific targeted areas support. The scores from seventy to less than eighty five (70 to <85) indicate acceptance level that requires careful specific interventions. And from eighty five percentages onwards is optimal level of the readiness of the hospitals to provide the available services that requires sustained efforts to maintain the level and move towards 100%.

Overall MSS Score and Color Coding

Being based on the overall MSS score (%) obtained, the color coding of the health facilities will be done as follows:

MSS overall score (%)	Color Code	
Less than 50	White	
50-70	Yellow	
70-85	Blue	
85-100	Green	

In the above example the overall MSS score is 68%, thus health post will be categorized in yellow color zone. It will be provided with the yellow flag as its color code for MSS score.

Section I: Governance and Management Standards

Summary Sheet for Standards and Scores

Area	Total Number of Standards	Total Score	Total Obtained Score (Percentage)
Governance	27	27	
Organizational Management	16	16	
Human Resource Management and Development	18	20	
Financial Management	17	17	
Medical Records and Information Management	14	14	
Quality Management	15	17	
Total	107	111	
Score of Section I<br><br>(Average of the percentage obtained = Sum of percentage obtained in each sub-section/ Number of sub-section (6))			

Area	Code	Verification		
**Governance**	**1.1**			
**Components**		**Standards**	**Obtained Score**	**Maximum Score**
1.1.1 Formation of Hospital Management Committee (HMC)	1.1.1	Hospital Management Committee is formed		1
1.1.2 Capacity building of HMC	1.1.2	All HMC members have received an orientation on HMC functions		1
1.1.3 Availability of Medical Superintendent/ Director	1.1.3	Medical Superintendent/ Director is fulfill as per organogram		1
1.1.4 Functional HMC	1.1.4.1	HMC meetings called upon by member secretary / Medical Superintendent headed by chairperson conducted at least 3 times per year or as per need		1
	1.1.4.2	HMC meetings have covered at least following agenda (See minutes of last meetings):		
	1.1.4.2.1	Hospital services and utilization		1
	1.1.4.2.2	Hospital's financial issues		1
	1.1.4.2.3	Patient rights issues e.g. patient facilities, analysis of complaints received, patient security		1
	1.1.4.2.4	Management issues- HR issues, security issues		1
	1.1.4.2.5	Infrastructure/ Equipment issues		1
	1.1.4.2.6	Coordination issues with local governance- rural municipality/ municipality, provincial, federal, DoHS, MoHP		1
	1.1.4.2.7	Review of decisions and recommendations of staff meeting and QI Committee meetings discussions		1
1.1.5 Support in health financing	1.1.5.1	Hospital implements health insurance program		1
	1.1.5.2	All targeted women receive Aama Surakhsya program incentives on time (in last quarter)		1
1.1.6 Annual plan & budget	1.1.6	Annual plan & budget is approved by HMC before the fiscal year starts		1
1.1.7Storage of HMC documents	1.1.7	There is a separate locker for HMC documents.		1

1.1.8 Accountability	1.1.8.1	Updated citizens charter is displayed		1
	1.1.8.2	Notices of public concern are displayed publicly		1
	1.1.8.3	Complaint boxes are kept in a visible place		1
	1.1.8.4	Information officer opens complaint box at least once a week and issues are timely addressed		1
	1.1.8.5	Hospital has a website or social media account like- Facebook, Viber or Twitter-available and functional with latest information		1
	1.1.8.6	Hospital has geriatrics friendly infrastructure (like side rails for mobilization and support)		1
	1.1.8.7	Hospital has friendly environment for people with disability		1
1.1.9 Grievance and complain handling	1.1.9.1	Mechanism for grievance and complain handling institutionalized		1
	1.1.9.2	Grievance and complains are effectively addressed		1
1.1.10 Hospital has operational manual	1.1.10	Hospital its own operational manual with clear information on how the hospital operates its' services		1
1.1.11 Hospital produces an Annual Report	1.1.11	Hospital Annual Report is available in website		1
1.1.12 Conduct social audit	1.1.12	Social audit is conducted for last year		1
**Standard 1.1**		**Total Obtained Score**		**27**
		**Percentage = Total Obtained Score / 27 x 100**		

**Area**	**Code**	**Verification**		
**Organizational Management**	**1.2**			
**Components**		**Standards**	**Obtained Score**	**Maximum Score**
1.2.1 Organizational structure	1.2.1.1	Organogram of hospital showing departments/units with number of staffs is displayed		1
	1.2.1.2	Organogram of hospital is reviewed every 2 years and forwarded to higher authority		1
1.2.2 Work division and delegation of authorities	1.2.2	Written delegation of authorities is maintained		1
1.2.3 Maintaining client flow system	1.2.3	Navigation chart with services and departments guiding clients to access services		1
1.2.4 Queue system	1.2.4	Hospital implements token and / or queue system for users (separate for elderly, disable and pregnant)		1

1.2.5 E-Attendance	1.2.5	All staffs of hospital use electronic attendance		1
1.2.6 Dress code for all staffs	1.2.6.1	All clinical, technical and administrative staffs have apron / uniform which is worn on duty		1
	1.2.6.2	All hospital staffs carry personal ID cards when on duty		1
1.2.7 Maintaining effective team work environment	1.2.7.1	Hand-over meetings are conducted daily and also in concerned department		1
	1.2.7.2	Morning conference is conducted everyday		1
	1.2.7.3	Regular meetings are conducted as follows (see meeting minutes):		
	1.2.7.3.1	Intra- departmental meeting every two weeks		1
	1.2.7.3.2	Inter-departmental meeting once a month		1
	1.2.7.3.3	Staff meeting once a month		1
	1.2.7.4	Staff quarters are provided and adequate for the staffs		1
	1.2.7.5	Separate space allocated for breast feeding for staffs/ Separate space in duty room designated for breast feeding		1
	1.2.7.6	Transport services for hospital staffs covering all shifts		1
**Standard 1.2**		**Total Obtained Score**		**16**
		**Percentage = Total Obtained Score / 16 x 100**		

Area	Code	Verification		
**Human Resource Management and Development**	**1.3**			
**Components**		**Standards**	**Obtained Score**	**Maximum Score**
1.3.1 Personnel administration policy of hospital	1.3.1	Personnel administration guideline of HMC is available (for all staffs including locally hired staff) and practiced accordingly		1
1.3.2 Human resource records	1.3.2	Individual records of all staffs including contract staffs are maintained and updated.		1
1.3.3 Staffing	1.3.3.1	Staffs available for service in hospital as per organogram (See Annex 1.3a Functional Organogram At the end of this standard)		3
	1.3.3.2	*Maaga Akriti* form (माग आकृति फाराम) correspondence to fulfill vacant positions to concerned authority as per guideline		1

1.3.4 Job description	1.3.4	All staffs including HMC staffs are given a job description when they are recruited/ posted to the hospital (permanent and contract staff)		1
1.3.5 Review of performance	1.3.5.1	Performance appraisal (का.स.मु.) of all staffs is done as per guideline		1
1.3.6 Motivating staff and occupational safety	1.3.6.1	A training plan for the hospital is developed based on the training needs of the staff identified at the performance appraisal		1
	1.3.6.2	For training and related activities, at any point of time, the acceptable work absenteeism is <10% of staff		1
	1.3.6.3	There is activity conducted to motivate staff (staff retreat, rewards, recognition of performances, etc.) at least once a year.		1
	1.3.6.4	Hospital has system for addressing occupational hazard like needle stick injury, radiation exposure, vaccination		1
1.3.7 Continuous professional development (CPD)/ Continuous medical education (CME)	1.3.7.1	Hospital conducts CPD / CME classes to technical staff weekly		1
	1.3.7.2	Written record of attendance, subjects presented and discussed during CPD/ CME		1
	1.3.7.3	Separate space with furniture, audio-visual aids and internet for CPD/CME/ meeting are available.		1
	1.3.7.4	If hospital is a training site, training guideline of National Health Training Centre is followed		1* (optional)
1.3.8 Library facility available	1.3.8.1	Hospital has its own library with sitting arrangement for readers		1
	1.3.8.2	A list of national health guidelines and treatment protocols available and inventory managed for readers accessing it		1
	1.3.8.3	Computers with printing and photocopy facility available		1
	1.3.8.6	Access to internet facility with institutional access to at least one of the international health related domain like HINARI		1
**Standard 1.3**		**Total Score**		**20**
		**Percentage = Total Obtained Score / 20 x 100**		

Annex 1.3a Functional Organogram for Tertiary Hospitals

Functional Organogram for Tertiary Hospitals			Obtained Score	Maximum Score
For Governance and Management				
1.	Medical Superintendent	1		1
2.	Hospital Management officer	1		1
3.	Information management officer	1		1
4.	Information Technology Officer	1		1
5.	Accountant for hospital	2		1
6.	Medical recorder	3		1
7.	Health Insurance Team	As per Health Insurance Board		1
For Clinical Services				
8.	Doctor: OPD Patients per OPD (Dental service)	1:35-50 (1:20)		1
9.	Screening counter	1 paramedics: 4 OPDs and for psychiatry OPD, psychological counselor at least 2, at least one nurse in gynae/obs OPD		1
10.	Special clinics	2 mid-level health workers: 1 Special Clinic*<br>*For safe abortion services, at least one trained and certified medical officer/ MDGP/ ObGyn for first trimester and second trimester safe abortion services		1
11.	ER beds: Health Workers	5 ER Beds: Doctor (1): Nurse (1): Paramedics (1): Office Assistant (1)<br>There should be 1:1 nurse patient ratio in red area, 1:3 in yellow area and 1:6 in green area.		1
12.	Pharmacy staffs as per pharmacy service guideline 2072	Pharmacy department is lead by at least one clinical pharmacist<br>Pharmacy has at least 3 pharmacist, 6 assistant pharmacist and 2 office assistants		1
13.	Nursing staff in inpatient per shift per ward	Nurse patient ratio 1:6 in general ward, 1:4 in pediatric ward, 1:2 in high dependency or intermediate ward or post-operative or burn/ plastic ward) and at least one trained office assistant/ward attendant per shift in each ward		1
14.	Nursing staff in labor and maternity per shift	Nurse /SBA trained/midwife mother ratio 1:2 in pre labor; 2:1 per delivery table and 1:6 in post natal ward) with at least one ASBA trained medical officer on duty and one office assistant are available per shift		1

15.	Nursing staff in haemodialysis per shift	One haemodialysis trained nurse per two Haemodialysis machine		1
16.	Intensive care service team	One coordinator each for ICU, NICU, PICU with at least MD Anesthesiology , MD Pediatrics respectively with at least one admitting consultant on duty each for ICU, NICU, PICU		1
		One trained medical officer for each five bed		
		Nurse-in-charge one each for ICU, NICU. PICU with nurse patient ratio:<br><br>1:1 for ventilated and multi organ failure, 2:3 for ventilated or multi-organ failure and 1:2 for other		
		IP trained office assistant 1 for every five bed and on call biomedical engineer in each shift		
17.	Nursing supervisor/ administrator	At least three nursing supervisor/administrator (one for IPD, OPD and emergency, one for maternity services, one for intensive care service and Operation Theatre)		1
18.	Surgery team per surgery	For one surgery, at least a team is composed of: MS with one trained medical officer, two OT trained nurse (one scrub and one circulating), one Anesthesiologist, one anesthesia assistant and one office assistant (for cleaning and helping)		1
		For overall management of operation theatre, there is one OT nurse (with minimum bachelors degree) assigned as OT in-charge		
		At least two nurses in pre-anesthesia area for receiving and transferring of the patient		
		At least two ICU trained nurses for post anesthesia care for receiving patient after OT		
19.	Laboratory	Laboratory team is lead by pathologist (at least) 3 (one for histocytopathology and hematology, 1 for biochemistry and 1 for microbiology) with at least 12 lab staffs (3 medical technologists, 3 technicians, 2 assistants and 2 helpers are available during routine working hours and on call biomedical engineer.		1
20.	X-ray	Adequate numbers of trained healthcare workers are available in x-ray (at least 2 staffs to cover shifts including ER)		1
21.	USG	USG trained medical practitioner and mid-level health worker in each USG room		1
22.	Echocardiogram	MD internal medicine with echo training or Cardiologist is available for Echo service with at least one mid-level health worker assigned in Echo is available for Echo service with at least one mid-level health worker assigned in Echo		1
23.	Treadmill test	At least one trained medical officer / cardiologist and one mid-level health worker is allocated for TMT service		1

24.	Endoscopy	Physician/ surgeons having endoscopic training or Gastroenterologist or hepatologist or gastrointestinal surgeons with at least 2 Trained endoscopic nurse/paramedic designated for endoscopy room and one trained record keeping staff nurse/paramedics		1
25.	Medico legal and forensics services	Team led by MD Forensic Medicine with at least one trained medical officer for mortuary service and medico-legal services		1
26.	OCMC	2 Staff nurse working in the hospital and 1 trained psycho social counselor)		1
27.	Physiotherapy	At least 3 physiotherapist trained in Masters in Physiotherapy (MPT) one each for orthopedics, cardiology and neurology, 6 Bachelors' in Physiotherapy (BPT) and 3 Certificate in physiotherapy (CPT) or Diploma in physiotherapy (DPT) and 3 office assistants		1
28.	Inpatient and outpatient dietetics and nutritional rehabilitation	one Senior Dietitian (Masters qualification in Nutrition & Dietetics including hospital internship or Bachelors in Nutrition & Dietetics with 1 years hospital experience) plus one dietetic assistant per hundred general beds, plus one office assistant<br><br>Additionally, 1 senior dietitian per 25 beds for all specialized services, including ICU, NICU, PICU, nephrology/hemodialysis		1
29.	Stabilization center	Inpatient bed: Nurses trained in stabilization of severely undernourished children with complications – 2:1		1
30.	Cath Lab	For one cardiac intervention, at least a team is composed of: MD Internal Medicine trained in cardiac intervention or cardiologist with one trained medical officer, two trained nursing/ paramedics, and one trained office assistant		1
For Support Services				
31.	CSSD	Separate staffs assigned for CSSD under leadership of trained nursing staff		1
32.	Laundry and housekeeping	There is a special schedule for collection and distribution of linens with visible duty roster for staffs laundry and housekeeping		1
33.	BMET	Human resource trained in biomedical engineering is designated for repair and maintenance		1
34.	Security	The hospital has trained security personnel round the clock.		1
35.	SSU	Facilitators at least 2 to 10 facilitators led by unit chief		1
		**Total Obtained Score**		**35**
		**Total Percentage= Total Obtained Score/ 35 x100**		

Each row gets a score of 1 in each row if it is available otherwise 0.

Scoring Chart	
**Total percentage**	**Score**
0-50	0
50-70	1
70-85	2
85-100	3
**Score for Standard 1.3.3**	

Area	Code	Verification		
**Financial management**	**1.4**			
**Components**		**Standards**	**Obtained Score**	**Maximum Score**
1.4.1 Account department of hospital	1.4.1.1	Dedicated account department of hospital with space and furniture		1
	1.4.1.2	At least one accountant available for hospital financial management		1
1.4.2 Formulation and approval of Annual Hospital Budget	1.4.2.1	An annual hospital budget is developed incorporating the revenue from services, government grants, and support provided by other organizations.		1
	1.4.2.2	Internal income is reviewed during budgeting every year.		1
1.4.3 Service fees	1.4.3	The service fees of the hospital are fixed by HMC every year.		1
1.4.4 Daily income	1.4.4	Daily income is deposited in the bank every day.		1
1.4.5 Financial review and audit	1.4.5.1	Budget absorption rate of last fiscal year is as per national target		1
	1.4.5.2	Internal audit, financial and physical progress review is done at least once each trimester (once in every 4 months).		1
	1.4.5.3	Final audit/ external audited accounts are available for last year.		1
1.4.6 Electronic database	1.4.6.1	The hospital uses central electronic billing system		1
	1.4.6.2	The hospital uses TABUCS/ LMBIS for accounting including local income and expenses by HMC.		1
1.4.7 Hospital prepares financial reports	1.4.7.1	The hospital prepares and keeps monthly financial report.		1
	1.4.7.2	Trimester financial report is produced (every 4 months) and financial status tracked and discussed in meetings		1
	1.4.7.3	Annual financial report is submitted to HMC.		1

1.4.8 Clearing financial irregularities	1.4.8.1	Financial irregularities are responded within 35 days		1
	1.4.8.2	Clearance of financial irregularities is done as per national target		1
1.4.9 Inventory inspection	1.4.9	Inventory inspection is done once in a year and managed accordingly		1
**Standard 1.4**		**Total Score**		**17**
		**Percentage = Total Obtained Score / 17 x 100**		

Area	Code	Verification		
**Medical Records and Information Management**	**1.5**			
**Components**		**Standards**	**Obtained Score**	**Maximum Score**
1.5.1 Managing medical records and use of electronic database	1.5.1.1	Client registration is digitized using standard software		1
	1.5.1.2	Referral in and out records are kept using the standard form (HMIS 1.4) and register.		1
	1.5.1.3	Electronic health record system that generates the HMIS monthly report (HMIS 9.4)is in place		1
1.5.2 Infrastructure and supplies for information management	1.5.2.1	There is a functional Medical Record Section		1
	1.5.2.2	All patients' records are kept in individual folders in racks or held digitally.		1
	1.5.2.3	There is a set of functional computer and printer available for maintaining medical records.		1
1.5.3 Evidence generation and utilization	1.5.3.1	Hospital monthly reports (HMIS 9.4) of the last three months are shared to the national database		1
	1.5.3.2	Hospital services utilization statistics are analyzed at least every month and shared with all the HODs and in-charge via email, paper and/or dashboard. (Check last three months status)		1
	1.5.3.3	Statistics including OPD morbidity pattern data, IPD data, surveillance data are analyzed and discussed in staff meeting and CPD/CME (Check the status in the last meeting)		1
	1.5.3.4	Key statistics of service utilization is displayed in respective Departments/ Wards		1
	1.5.3.5	Medico-legal incidents and services are recorded		1

1.5.4 Focal person for information management	1.5.4.1	Medical recorder is trained on ICD and DHIS2		1
	1.5.4.2	An information officer is specified to communicate with patients/clients, their relatives, media and other stakeholders.		1
	1.5.4.3	Contact details of information officer is displayed in hospital premises with photo and phone number.		1
**Standard 1.5**		**Total Score**		**14**
		**Percentage = Total Obtained Score / 14x 100**		

Area	Code	Verification		
**Quality Management**	**1.6**			
**Components**		**Standards**	**Obtained Score**	**Maximum Score**
1.6.1 Hospital Quality Health Service Delivery and Management Strengthening (QHSDMS) Committee	1.6.1.1	Hospital QHSDMS committee is formed according to MSS Implementation Guideline.		1
	1.6.1.2	Hospital QHSDMS committee meetings are held at least every 4 months.		1
1.6.2 Display of patients' rights and responsibilities	1.6.2	The hospital has a statement of patient rights and responsibilities, which is posted in public places in the hospital.		1
1.6.3 Addressing issues in report of social audit	1.6.3	The findings of social audit like client exit interview are shared in whole staff meeting		1
1.6.4 Assessing hospital quality	1.6.4	The hospital has assessed the hospital quality using the MSS tool at least every 4 months		1
1.6.5 Planning to improving quality	1.6.5	The hospital has developed specific plans to improve quality based on the MSS assessment.		1
1.6.6 Hospital uses QI tools	1.6.6	Hospital uses QI tools for assessment of the major priority government programs (less than 50%=0, 50-70% =1, 70-85% = 2, 85-100% =3)		3
1.6.7Implementing QI plan	1.6.7.1	Hospital has implemented the specific activities based on the MSS plan.		1
	1.6.7.2	Hospital has implemented specific activities based on gap analysis of QI tools		1

1.6.8 Clinical Audit	1.6.8.1	The hospital has functional MPDSR committee (in program district)		1
	1.6.8.2	There are regular reviews, reporting and dissemination of morbidity and mortality (M&M) including		
	1.6.8.2.1	Investigations and complications of treatment including medication error		1
	1.6.8.2.2	Hospital acquired infections (HAI)		1
	1.6.8.3	Mortality audit of every death in the hospital is done and reported		1
	1.6.8.4	Hospital implement Robson's classification (hospitals with CEONC services)		1
	1.6.8.5	Hospital implement baby friendly initiative		1
**Standard 1.6**		**Total Obtained Score**		**17**
		**Percentage = Total Obtained Score/17 x 100**		

Section II: Clinical Service Management

Summary Sheet for Standards and Scores

Area	Total Number of Standards	Total Score	Total Obtained Score (Percentage)
OPD Service	57	145	
Special Clinic	67	73	
Emergency Services	38	46	
Emergency Operation Theatre (OT)	15	27	
Hospital Pharmacy Service	36	40	
Inpatient Service	58	154	
Delivery Service	34	40	
Maternity Inpatient Service (General Ward)	27	33	
Birthing Center	31	37	
Surgery/Operation Service	47	73	
Hemodialysis	38	42	
Intensive Care Unit (ICU)	34	40	
NICU	35	39	
PICU	34	38	
Diagnostics and Laboratory Services	172	184	
Post-mortem and morturay service	12	14	
Medico-legal Services	12	14	
One stop crisis management services	23	29	
Physiotherapy Services	20	22	
Nutrition Rehabilitation and Dietetics	22	22	
Cardiac Catheterization Laboratory	28	36	
Total	840	1148	
Score of Section II<br>(Average of the percentage obtained = Sum of percentage obtained in each sub-section/ Number of sub-section (21))			

Area	Code	Verification		
OPD Service [1]	2.1			
**Standards**		**Service Standards**	**Obtained Score**	**Maximum Score**
2.1.1 Time for patients	2.1.1.1	OPD is open from 10 AM to 3 PM (See Checklist 2.1 At the end of this standard for scoring).		3
	2.1.1.2	Tickets for routine OPD are available till 2 pm		1
	2.1.1.3	EHS services from 3PM onwards and tickets available from 2PM onwards		1
2.1.2 Adequate Staffing	2.1.2.1	There should be one administrator to manage all OPDs and procedure room		1
	2.1.2.2.	Doctor: OPD Patients- 1:35-50 per day for quality of care (*for dental services this ratio is 1:20)		1
	2.1.1.2.3	One screening counter with 1 paramedics for every four OPDs and there should be one nurse in OBGYN OPD		1
2.1.3 Maintaining patient privacy	2.1.3	Patient privacy maintained with separate rooms, curtains hung, maintaining queuing of patients with paging system in OPD (See Checklist 2.1 At the end of this standard for scoring).		3
2.1.4 Patient counseling	2.1.4.1	Counseling is provided to patients about the type of treatment being given and its consequences (See Checklist 2.1 At the end of this standard for scoring).		3
	2.1.4.2	Appropriate IEC materials (posters, leaflets etc.) as an IEC corner available in the OPD waiting area.		1

1 Separate set of sheets for standards, checklist and annexes should be used for assessment of each OPD and cumulative scoring is done after the assessment of all OPDs

2.1.5 Physical facilities	2.1.5.1	Adequate rooms and space for the practitioners and patients are available (See Checklist 2.1 At the end of this standard for scoring).		3
	2.1.5.2	Light and ventilation are adequately maintained. (See Checklist 2.1 At the end of this standard for scoring)		3
	2.1.5.3	Required furniture, supplies and space are available		
	2.1.5.3.1	**General Medicine OPD** (See Annex 2.1a Furniture and supplies for OPD At the end of this standard)		3
	2.1.5.3.2	**Obstetrics and Gynecology OPD** (See Annex 2.1a Furniture and supplies for OPD At the end of this standard)		3
	2.1.5.3.3	**Pediatrics OPD** (See Annex 2.1a Furniture and supplies for OPD At the end of this standard)		3
	2.1.5.3.4	**Surgical OPD** (See Annex 2.1a Furniture and supplies for OPD At the end of this standard)		3
	2.1.5.3.5	**Dental OPD** (See Annex 2.1a Furniture and supplies for OPD At the end of this standard)		3
	2.1.5.3.6	**Orthopedics OPD** (See Annex 2.1a Furniture and supplies for OPD At the end of this standard)		3
	2.1.5.3.7	**Psychiatry OPD** (See Annex 2.1a Furniture and supplies for OPD At the end of this standard)		3
	2.1.5.3.8	**ENT OPD** (See Annex 2.1a Furniture and supplies for OPD At the end of this standard)		3
	2.1.5.3.9	**Cardiology OPD (**See Annex 2.1a Furniture and supplies for OPD At the end of this standard)		3
	2.1.5.3.10	**Dermatology OPD** (See Annex 2.1a Furniture and supplies for OPD At the end of this standard)		3
	2.1.5.3.11	**Urology OPD** (See Annex 2.1a Furniture and supplies for OPD At the end of this standard)		3
	2.1.5.3.12	**Neurosurgery OPD (**See Annex 2.1a Furniture and supplies for OPD At the end of this standard)		3
	2.1.5.3.13	**Neurology OPD** (See Annex 2.1a Furniture and supplies for OPD At the end of this standard)		3
	2.1.5.3.14	**Burn/Plastic OPD** (See Annex 2.1a Furniture and supplies for OPD At the end of this standard)		3
	2.1.5.3.15	**Nephrology OPD** (See Annex 2.1a Furniture and supplies for OPD At the end of this standard)		3
	2.1.5.33.16	**Gastrology OPD** (See Annex 2.1a Furniture and supplies for OPD At the end of this standard)		3
	2.1.5.4	Each OPD has designated space/room for procedure room/area with basic supplies of dressing, injection and procedures (specific to OPD like PV for Obstetrics /gynecology, PR for surgery) and hand washing facility (See Checklist 2.1 At the end of this standard for scoring)		3

2.1.6 Equipment, instrument and supplies	2.1.6	Equipment, instrument and supplies to carry out the OPD works are available and functioning		
	2.1.6.1	General OPD (See Annex 2.1b Basic Equipment and Instrument for OPD At the end of this standard)		3
	2.1.6.2	Obstetrics and Gynecology OPD (See Annex 2.1b Basic Equipment and Instrument for OPD At the end of this standard)		3
	2.1.6.3	Pediatrics OPD (See Annex 2.1b Basic Equipment and Instrument for OPD At the end of this standard)		3
	2.1.6.4	Surgical OPD(See Annex 2.1b Basic Equipment and Instrument for OPD At the end of this standard)		3
	2.1.6.5.1	Dental OPD (See Annex 2.1cBasic Equipment and Instrument for Dental OPD At the end of this standard)		3
	2.1.6.5.2	Dental OPD has fully functioning electric dental chair with adequate light, water supply and drainage system with suction machine		1
	2.1.6.5.3	Dental OPD has iopa x-ray machine (along with lead apron and thyroid collar		1
	2.1.6.6	Orthopedics OPD (See Annex 2.1b Basic Equipment and Instrument for OPD At the end of this standard)		3
	2.1.6.7	Psychiatry OPD (See Annex 2.1b Basic Equipment and Instrument for OPD At the end of this standard)		3
	2.1.6.8	ENT OPD (See Annex 2.1b Basic Equipment and Instrument for OPD At the end of this standard)		3
	2.1.6.9	Cardiology OPD (See Annex 2.1b Basic Equipment and Instrument for OPD At the end of this standard)		3
	2.1.6.10	Dermatology OPD (See Annex 2.1b Basic Equipment and Instrument for OPD At the end of this standard)		3
	2.1.6.11	Urology OPD (See Annex 2.1b Basic Equipment and Instrument for OPD At the end of this standard)		3
	2.1.6.12	Neurosurgery OPD (See Annex 2.1b Basic Equipment and Instrument for OPD At the end of this standard)		3
	2.1.6.13	Neurology OPD (See Annex 2.1b Basic Equipment and Instrument for OPD At the end of this standard)		3
	2.1.6.14	Burn/Plastic OPD (See Annex 2.1b Basic Equipment and Instrument for OPD At the end of this standard)		3
	2.1.6.15	Nephrology OPD (See Annex 2.1b Basic Equipment and Instrument for OPD At the end of this standard)		3
	2.1.6.16	Gastrology OPD (See Annex 2.1b Basic Equipment and Instrument for OPD At the end of this standard)		3

2.1.7 Duty rosters	2.1.7	Duty rosters of all OPDs are developed regularly and available in appropriate location.		1
2.1.8 Facilities for patients	2.1.8.1	Comfortable waiting space with sitting arrangement is available for at least 100 persons in waiting lobby (for total OPDs)		1
	2.1.8.2	Safe drinking water is available in the waiting lobby throughout the day.		1
	2.1.8.3	There are four toilets with hand-washing facilities (2 for males and 2 for females separate, one each universal toilet)		1
	2.1.8.4	Hand-washing facilities are available for patients		1
2.1.9 Recording and reporting	2.1.9	OPD register available in every OPD with ICD 10 classification for diagnosis recorded (electronic health recording system) (See checklist 2.1 At the end of this standard for scoring)		3
2.1.10 Infection prevention	2.1.10.1	Masks and gloves are available and used (See Checklist 2.1 At the end of this standard for scoring)		3
	2.1.10.2	There are well labeled colored bins for waste segregation and disposal as per HCWM guideline 2014 (MoHP) (See Checklist 2.1 At the end of this standard for scoring)		3
	2.1.10.3	Hand-washing facility with running water and soap or hand sanitizer is available for practitioners (See Checklist 2.1 At the end of this standard for scoring)		3
	2.1.10.4	Needle cutter is used (See Checklist 2.1 At the end of this standard for scoring)		3
	2.1.10.5	Chlorine solution is available and utilized for decontamination (See Checklist 2.1 At the end of this standard for scoring).		3
**Standard 2.1**		**Total Score**		145
		**Total Percentage (Total Score/ 145 x100)**		

Checklist 2.1 OPD Services
(1= General Medicine, 2= Obs/Gyne, 3= Pediatrics, 4= General Surgery, 5= Dental, 6= Orthopedics, 7=Psychiatry, 8= ENT, 9= Cardiology, 10= Dermatology, 11= Urology, 12= Neurosurgery, 13= Neurology, 14=Burn/Plastic, 15=Nephrology, 16= Gastrology)

Code	Service Standards	Score																		
		1	2	3	4	5	6	7	8	9	10	11	12	13	14	15	16	Total Score	Percentage	Direction to use
2.1.1.1	OPD is open from 10 AM to 3 PM *																			Go to Standard 2.1.1.2
2.1.3	Appropriate techniques have been used to ensure the patient privacy (separate rooms, curtains hung, maintaining queuing of patients)																			Go to Standard 2.1.4
2.1.4.1	Counseling is provided to patients about the type of treatment being given and its consequences																			Go to Standard 2.1.5
2.1.5.1	Adequate rooms and space for the practitioners and patients are available																			Go to Standard 2.1.5.2
2.1.5.2	Light and ventilation are adequately maintained																			Go to Standard 2.1.5.3
2.1.5.4	Each OPD has designated space/ room for procedure room/area with basic supplies of dressing, injection and procedures and hand washing facility																			Go to Standard 2.1.6
2.1.9	OPD register available in every OPD and ICD 10 classification for diagnosis recorded (electronic health recording system)																			Go to Standard 2.1.10
2.1.10.1	Masks and gloves are available and used																			Go to Standard 2.1.10.2
2.1.10.2	There are well labelled colored bins for waste segregation and disposal as per HCWM guideline 2014 (MoHP)																			Go to Standard 2.1.10.3

2.1.10.3	Hand-washing facility with running water and soap or hand sanitizer is available for practitioners																			Go to Standard 2.1.10.4
2.1.10.4	Needle cutter is used																			Go to Standard 2.1.10.5
2.1.10.5	Chlorine solution is available and utilized for decontamination																			Score Standard 2.1

Total percentage = Total score / No. of OPD x 100;
Each row gets a score of 1 in each row if is available otherwise 0

Scoring Chart	
**Total Percentage**	**Score**
0-50	0
50-70	1
70-85	2
85-100	3

Plot score based on scoring chart in obtained marks of respective standards
*Specialized OPDs can be scheduled 2-3 days a week based on available human resource and patients' flow

Annex 2.1a Furniture and Supplies for OPD

(1= General Medicine, 2= Obs/Gyne, 3= Pediatrics, 4= General Surgery, 5= Dental, 6= Orthopedics, 7=Psychiatry, 8= ENT, 9= Cardiology, 10= Dermatology, 11= Urology, 12= Neurosurgeon, 13= Neurology, 14= Burn/Plastic, 15=Nephrology, 16= Gastrology)

SN	General Items	Required No.	Score															
			1	2	3	4	5	6	7	8	9	10	11	12	13	14	15	16
1	Working desk	1 for each practitioner																
2	Working Chairs	1 for each practitioner																
3	Patient chairs	2 for each working desk																
4	Examination table	1 in each OPD room																
	Foot Steps	1 in each OPD room																
5	Curtain separator for examination beds	In each examination bed																
6	Shelves for papers	As per need																
7	Weighing scale	Adult and Child																
		**Total Score**																
	**Total Percentage = Total Score/7 X 100**																	
*For psychiatry OPD, furniture should be fixed to prevent harm from violent patients																		

Each row gets a score of 1 in each row if is available otherwise 0

**Scoring chart**	
**Total percentage**	**Score**
0-50	0
50-70	1
70-85	2
85-100	3
**Score for Standard 2.1.5.3.1**	
**Score for Standard 2.1.5.3.2**	
**Score for Standard 2.1.5.3.3**	
**Score for Standard 2.1.5.3.4**	
**Score for Standard 2.1.5.3.5**	
**Score for Standard 2.1.5.3.6**	
**Score for Standard 2.1.5.3.7**	
**Score for Standard 2.1.5.3.8**	
**Score for Standard 2.1.5.3.9**	
**Score for Standard 2.1.5.3.10**	
**Score for Standard 2.1.5.3.11**	
**Score for Standard 2.1.5.3.12**	
**Score for Standard 2.1.5.3.13**	
**Score for Standard 2.1.5.3.14**	
**Score for Standard 2.1.5.3.15**	
**Score for Standard 2.1.5.3.16**	

Annex 2.1b Basic Equipment and Instruments for OPD

(1= General Medicine, 2= Obs/Gyne, 3= Pediatrics, 4= General Surgery, 6= Orthopedics, 7=Psychiatry, 8= ENT, 9= Cardiology, 10= Dermatology, 11= Urology, 12= Neurosurgery, 13= Neurology, 14= Burn/Plastic, 15= Nephrology, 16=Gastrology)

S.No.	Basic equipment and instruments	Required No.	Score														
			1	2	3	4	6	7	8	9	10	11	12	13	14	15	16
1.	Stethoscope*	1 for each practitioner															
2.	Sphygmomanometer* (non-mercury) (*Pediatric size in pediatric OPD)	1 for each practitioner															
3.	Thermometer (digital)	2 in each table															
4.	Jerk hammer	1 for each practitioner															
5.	Flash light	1 for each practitioner															
6.	Disposable wooden tongue depressor	As per need															
7.	Hand sanitizer	1 in each table															
8.	Examination Gloves	As per need															
9.	X-Ray View Box	1 in each OPD															
10.	Measuring tape	1 in each table															
11.	Tuning fork	1 in each table															
12.	Proctoscope	1															
13.	Otoscope	1															
14.	Duck's Speculum	1															
15.	Aeyer's Spatula/ Slides (Pap Smear/ VIA materials)	1															
16.	Betadine/Swab	1															
17.	Fetoscope	1															
18.	Pediatric Paracetamol	At least one syrup															
19.	ORS	At least one sachet															
20.	Goniometer	1 in each table															
21.	Plaster cutter	1															
22.	Diagnostic tools for psychiatry	1 set															

S.No.	Basic equipment and instruments	Required No.	Score														
			1	2	3	4	6	7	8	9	10	11	12	13	14	15	16
23.	Nasal speculum of different size	1 set															
24.	Bull's eye lamp	1															
25.	Head mirror	1 for each practitioner															
26.	ENT Forceps	1 set															
27.	Nasopharyngolaryngoscpe with monitor	1															
28.	Indirect Laryngoscopy mirrors	1															
29.	Posterior rhinoscopy mirrors	1															
30.	Magnifying glass	1															
31.	Table lamp with good light source	1															
32.	Glass slides	As per need															
33.	Wood's Lamp	1															
34.	Cystoscope	1															
35.	Ophthalmoscope	1															
36.	EEG (24 channel)	1															
37.	EMG machine also supporting MNCV, SMC, VEP	1															
38.	Dressing trolley with drum with gauze pad	1 set															
**Total score**																	
Maximum Score			13	17	13	14	15	12	18	11	16	12	11	14	12	11	12
**Total percentage= Total Score/ Maximum Score x 100**																	

* For pediatrics OPD, pediatric size

(1= General Medicine, 2= Obs/Gyne, 3= Pediatrics, 4= General Surgery, 6= Orthopedics, 7=Psychiatry, 8= ENT, 9= Cardiology, 10= Dermatology, 11= Urology, 12= Neurosurgery, 13= Neurology, 14= Burn/Plastic, 15= Nephrology, 16=Gastrology)

Each row gets a score of 1 in each row if is available otherwise 0

Scoring chart	
**Total percentage**	**Score**
0-50	0
50-70	1
70-85	2
85-100	3
**Score for Standard 2.1.6.1**	
**Score for Standard 2.1.6.2**	
**Score for Standard 2.1.6.3**	
**Score for Standard 2.1.6.4**	
**Score for Standard 2.1.6.6**	
**Score for Standard 2.1.6.7**	
**Score for Standard 2.1.6.8**	
**Score for Standard 2.1.6.9**	
**Score for Standard 2.1.6.10**	
**Score for Standard 2.1.6.11**	
**Score for Standard 2.1.6.12**	
**Score for Standard 2.1.6.13**	
**Score for Standard 2.1.6.14**	
**Score for Standard 2.1.6.15**	
**Score for Standard 2.1.6.16**	

Annex 2.1c Basic Equipment and Instrument for Dental OPD

S.No.	Instruments and Equipment for Dental OPD	Required numbers	Score
Diagnostic			
1.	Mouth mirror	10	
2.	Explorer	10	
3.	St. Probe	5	
4.	Tweezers	10	
5.	Periodontal probe	2	
6.	Kidney tray small and large	5	
7.	Plastic tray	10	
Extraction forceps			
8.	Upper premolar	1	
9.	Upper molar (right)	2	
10.	Upper molar (left)	1	
11.	Upper third molar	1	
12.	Lower cowhorn forceps	3	
13.	Lower third molar	1	
14.	Lower root forceps	1	
Elevators			
15.	Compland elevators (small and large)	10	
16.	Cryers	1 set	
17.	Pointed elevator	2	
18.	Apexoelevator	2	
Surgical			
19.	Bp handle	2	
20.	Needle holder	3	
21.	Artery forceps	2	
22.	Toothed forceps	2	
23.	Scissors (suture cutting)	1	
24.	21 no wire	2 packets	
25.	Wire cutter	1	
Restorative			
26.	Airotor handpiece	2	

Burs			
27.	Round burs (smalland large)	5	
28.	Straight bur	2	
29.	Inverted cone bur	2	
30.	Composite finishing bur	1	
31.	Cement spatula	1	
32.	Plastic spatula	1	
33.	Glass slab	1	
34.	Mixing paper pad	1	
35.	Cement carrier	5	
36.	Condenser (round)	5	
37.	Ball burnisher	2	
38.	Spoon excavators	5	
39.	Toffle wire matrix retainer	1	
40.	Matrix band (steel)	2 packets	
41.	Matrix band (plastic)	1 packets	
42.	Wedge	1 packets	
43.	Dycal tip	2	
Dental materials			
44.	Gic (restorative)	1 set	
45.	Miracle mix	1 set	
46.	Composite filling set		
47.	Etchant	1	
48.	Bonding agent	1	
49.	Composite = shades a_1 $a_2a_3b_1b_2$	1 each	
50.	Bonding agent applicator	1 packet	
51.	Dycal	1 set	
52.	Cavit(temporary restorative)	1	
53.	Zinc phosphate (restorative)	1 set	
54.	Vaseline	1	
Scaling			
55.	Suction tips	2 packets	
56.	Curette (universal curette)	3	

Pedo forceps			
57.	Upper anterior	2	
58.	Upper root	1	
59.	Upper molar	2	
60.	Lower anterior	2	
61.	Lower molar	2	
Additional instruments/supplies			
62.	Local anesthesia (2% lidocane with adrenaline)	1 box	
63.	Syringe 1ml 2ml 3ml	1 packet each	
64.	Gauge	1 packet	
65.	Cotton roll	1 packet	
66.	Normal sline	1 bottle	
67.	Betadine	1 bottle	
68.	Micromotor (slow speed round bur)	1(2)	
69.	H_2o_2	1 bottle	
70.	Dental floss	1 packet	
71.	Surgical gloves	As per need	
72.	Loose gloves	As per need	
		**Total score**	
		**Percentage= Total score/ 72 x 100**	

Each row gets a score of 1 in each row if is available otherwise 0

**Scoring Chart**	
**Total Percentage**	**Score**
0-50	0
50-70	1
70-85	2
85-100	3
**Score 2.1.6.5.1**	

Area	Code	Verification		
**Special Clinics**	**2.2**			
**Immunization and Growth Monitoring Clinic**	**2.2.1**			
**Components**		**Standards**	**Obtained Score**	**Maximum Score**
2.2.1.1 Time for patients	2.2.1.1	Immunization and growth monitoring service is available from 10 AM to 3 PM.		1
2.2.1.2 Staffing	2.2.1.2	Adequate numbers of healthcare workers are available (at least 2 mid-level health workers are assigned)		1
2.2.1.3 Maintaining patient privacy	2.2.1.3	Appropriate techniques have been used to ensure the patient privacy (separate rooms, curtains hung, maintaining queuing of patients).		1
2.2.1.4 Patient counseling	2.2.1.4.1	Counseling is provided to caretaker about the type of vaccine, its schedule, nutritional status of child.		1
	2.2.1.4.2	Appropriate IEC/BCC materials on vaccine, schedule and child growth and nutrition are available in clinic		1
2.2.1.5 Instrument, equipment and supplies available	2.2.1.5	Immunization and growth monitoring instrument, equipment and supplies are available (See Annex 2.2.1a Immunization and growth monitoring At the end of this standard)		3
2.2.1.6 Physical facilities	2.2.1.6.1	Adequate rooms and space for health worker and patients are available with at least one working table, chair for health worker and two patients' chair		1
	2.2.1.6.2	Light and ventilation are adequately maintained.		1
2.2.1.7 Recording and reporting	2.2.1.7.1	Patient's card (Health card, growth chart) and register available and services recorded		1
	2.2.1.7.2	Adverse immunization reactions, complication, severe under-nutrition and referral to other sites recorded and reported		1
2.2.1.8 Infection prevention	2.2.1.8.1	Masks and gloves are available and used		1
	2.2.1.8.2	There are well labelled colored bins for waste segregation and disposal as per HCWM guideline 2014 (MoHP)		1
	2.2.1.8.3	Hand-washing facility with running water and soap is available for practitioners.		1
	2.2.1.8.4	Needle cutter is used		1
	2.2.1.8.5	Chlorine solution is available and utilized.		1
**Standard 2.2.1**		**Total Score**		17
		**Total Percentage (Total Score/ 17 x100)**		

Annex 2.2.1a Instruments, equipment and Supplies for Immunization and Growth Monitoring

SN	Name	Required Quantity	Score
1	Weighing scale (Infantometer and Secca Scale)	At least one each	
2	Stadiometer	At least one	
3	MUAC tape	2	
4	Cold chain box set	At least one set	
5	Immunization as per national protocol	At least two vial/ampule each	
6	Different size syringe for immunization (1,2,3,5,10 ml)	At least 10 each	
7	Cotton in swab container	As per needed	
8	Container for clean water	As per needed	
		**Total score**	
		**Percentage = Total score/ 8 x 100**	

Each row gets a score of 1 in each row if is available otherwise 0

**Scoring Chart**	
**Total Percentage**	**Score**
0-50	0
50-70	1
70-85	2
85-100	3
**Score for Standard 2.2.1.5**	

**Area**	**Code**	**Verification**		
**Family planning Clinic**	**2.2.2**			
**Components**		**Service Standards**	**Obtained Score**	**Maximum Score**
2.2.2.1 Time for patients	2.2.2.1	Family planning service is available from 10 AM to 3 PM.		1
2.2.2.2 Space	2.2.2.2	A separate area dedicated for FP counseling and Services		1
2.2.2.3 Staffing	2.2.2.3	Adequate numbers of healthcare workers are available (at least 2 mid-level health workers are assigned)		1
2.2.2.4 Maintaining patient privacy	2.2.2.4	Appropriate techniques have been used to ensure the patient privacy (separate rooms, curtains hung, maintaining queuing of patients).		1
2.2.2.5 Patient counseling	2.2.2.5.1	Counseling is provided to users for family planning methods		1
	2.2.2.5.2	Appropriate IEC/BCC materials on family planning including DMT tool used for counseling		1

2.2.2.6 Supplies available	2.2.2.6	Supplies for Family Planning Services available (See Annex 2.2.2a Supplies for FP services At the end of this standard)		3
2.2.2.7 Equipment and supplies available	2.2.2.7	Functional BP set, stethoscope, thermometer, and weighing scale available		1
2.2.2.8 Physical facilities	2.2.2.8.1	Adequate rooms and space for health worker and patients are available with at least one working table, chair for health worker and two patients' chair and one examination bed		1
	2.2.2.8.2	Light and ventilation are adequately maintained.		1
2.2.2.9 Recording and reporting	2.2.2.9.1	Patient's health card and register available and services recorded		1
	2.2.2.9.2	FP related complication, defaulter and contraceptive failure are recorded and reported		1
2.2.2.10 Infection prevention	2.2.2.10.1	Masks and gloves are available and used		1
	2.2.2.10.2	There are well labelled colored bins for waste segregation and disposal as per HCWM guideline 2014 (MoHP)		1
	2.2.2.10.3	Hand-washing facility with running water and soap is available for practitioners.		1
	2.2.2.10.4	Needle cutter is used		1
	2.2.2.10.5	Chlorine solution is available and utilized.		1
**Standard 2.2.2**		**Total Score**		19
		**Total Percentage (Total Score/ 19 x100)**		

Annex 2.2.2a Supplies for Family Planning

SN	Name	Required Quantity	Score
1.	Condoms	As per needed	
2.	Combined oral contraceptive pills	As per needed	
3.	IUD	As per needed	
4.	IUD Insertion and removal Set	At least 2	
5.	Implants	As per needed	
6.	Implants insertion and removal set	At least 2	
7.	Injection Depo provera	As per needed	
8.	Emergency contraceptive pills	As per need	
9.	Sterile surgical gloves (different sizes)	2-3	
		**Total score**	
		**Percentage = Total score/ 9 x 100**	

Each row gets a score of 1 in each row if is available otherwise 0

Scoring Chart	
**Total Percentage**	**Score**
0-50	0
50-70	1
70-85	2
85-100	3
**Score for Standard 2.2.2.6**	

**Area**	**Code**	**Verification**		
**ATT, ART clinic**	**2.2.3**			
**Components**		**Service Standards**	**Obtained Score**	**Maximum Score**
2.2.3.1 Time for patients	2.2.3.1	Clinic is open from 10 AM to 3 PM.		1
2.2.3.2 Staffing	2.2.3.2	Adequate numbers of healthcare workers are available in OPD (at least 2 mid-level health workers are assigned)		1
2.2.3.3 Maintaining patient privacy	2.2.3.3	Appropriate techniques have been used to ensure the patient privacy (separate rooms, curtains hung, maintaining queuing of patients).		1
2.2.3.4 Patient counseling	2.2.3.4.1	Counseling is provided to patients about the type of treatment being given and its consequences.		1
	2.2.3.4.2	Appropriate IEC/BCC materials on TB, HIV/ AIDS (posters, leaflets) are available in the OPD waiting area.		1
2.2.3.5 Medicine available	2.2.3.5	Medicines for TB, HIV/AIDS as per government treatment protocol available in OPD		1
2.2.3.6 Equipment and supplies available	2.2.3.6	OPD has functional BP set, stethoscope, thermometer and weighing scale		1
2.2.3.7 Physical facilities	2.2.3.7.1	Adequate rooms and space for health worker and patients are available with at least one working table, chair for health worker and two patients' chair		1
	2.2.3.7.2	Light and ventilation are adequately maintained.		1
2.2.3.8 Facilities for patients	2.2.3.8.1	Safe drinking water with mug or glass is available for taking medicine		1
	2.2.3.8.2	Hand-washing facilities are available for patients.		1

2.2.3.9 Recording and reporting	2.2.3.9.1	Patient's card (TB, ART) and register available and services recorded		1
	2.2.3.9.2	Drug resistance, complication and referral to other sites recorded and reported		1
2.2.3.10 Infection prevention	2.2.3.10.1	Masks and gloves are available and used		1
	2.2.3.10.2	There are well labelled colored bins for waste segregation and disposal as per HCWM guideline 2014 (MoHP)		1
	2.2.3.10.3	Hand-washing facility with running water and soap is available for practitioners.		1
	2.2.3.10.4	Needle cutter is used		1
	2.2.3.10.5	Chlorine solution is available and utilized.		1
**Standard 2.2.3**		**Total Score**		18
		**Total Percentage (Total Score/ 18 x100)**		

**Area**	**Code**	**Verification**		
**Special Clinics**	**2.2**			
**Safe Abortion Services**	**2.2.4**			
**Components**		**Standards**	**Obtained Score**	**Maximum Score**
2.2.4.1 Time for patients	2.2.4.1	Safe abortion services is available from 10 AM to 3 PM.		1
2.2.4.2 Space	2.2.4.2	A separate area dedicated for Safe Abortion counseling and services, area is washable and has separate instrument processing space for decontamination		1
2.2.4.3 Staffing	2.2.4.3.1	At least one medical officer or gynecologist trained and certified in first trimester SAS is available		1
	2.2.4.3.1	For surgical abortion, at least one medical officer or gynecologist or MDGP trained and certified in second trimester SAS is available		
2.2.4.4 Maintaining patient privacy	2.2.4.4	Appropriate techniques have been used to ensure the patient privacy (separate rooms, curtains hung, maintaining queuing of patients).		1
2.2.4.5 Patient counseling	2.2.4.5.1	Counseling is provided to users on Safe Abortion Services, complication and family planning post abortion along with clear discharge instructions		1
	2.2.4.5.2	Appropriate IEC/BCC materials on safe abortion services and post abortion family planning services –Medical Abortion Chart, CAC counseling flip chart, second trimester counseling flipchart, DMT Tools used for counseling		1
2.2.4.6 WHO Safe Surgery Checklist available	2.2.4.6	WHO safe surgery checklist is available and used for safe abortion services including written informed consent		

2.2.4.7 Instruments, equipments and Supplies available	2.2.4.7.1	Instruments, equipments and supplies for Safe Abortion Services available (See Annex 2.2.2a Instruments, equipments and supplies for Safe Abortion services At the end of this standard)		3
	2.2.4.7.2	Functional BP set, stethoscope, thermometer, and weighing scale available		1
2.2.4.8Physical facilities	2.2.4.8.1	Adequate rooms and space for health worker and patients are available with at least one working table, chair for health worker and two patients' chair, one examination bed, one procedure table and one foot step		1
	2.2.4.8.2	Light and ventilation are adequately maintained.		1
2.2.4.9 Recording, reporting and histological examination	2.2.4.9.1	Patient's health card and register available and services recorded along with complications if any		1
	2.2.4.9.2	Product of conception is sent for histopathlogical examination and reports followed up		1
2.2.4.10 Infection prevention	2.2.4.10.1	Utility Gloves, Gumboot, Mask, Plastic Apron, Caps are available and used		1
	2.2.4.10.2	There are well labelled colored bins for waste segregation and disposal as per HCWM guideline 2014 (MoHP)		1
	2.2.4.10.3	Hand-washing facility with running water and soap is available for practitioners.		1
	2.2.4.10.4	Needle cutter is used.		1
	2.2.4.10.5	Chlorine solution is available and utilized.		1
**Standard 2.2.4**		**Total Obtained Score**		19
		**Total Percentage (Total Obtained Score/ 19 x100)**		

Annex 2.2.4a Instruments, equipments and supplies for Safe Abortion services

SN	Name	Required Quantity	Score
1.	Shelf for storage	At least 1	
2.	Reliable Light source (goose neck light)	At least 1	
3.	Oxygen concentrater/ Oxygen filled cylinder with flow meter and mask	At least 1 Set	
4.	Light view box with glass/ plastic container and sieve for POC check	At least 1 each	
5.	Intubation set adult	1 set	
6.	IV stand	At least 1	
7.	Surgical drum (2)	As per needed	
8.	Sterilized Chettle forceps with jar	At least 2	
9.	Bivalve Speculum (3 sized- small, medium and large)	At least 3 each	
10.	Stainless steel container with cover for storing instruments	At least 2	

11.	Cheatle's forceps with jar	At least 2	
12.	Instrument trolley	At least 2	
13.	Abdominal drapes	As per need	
14.	MVA aspirator	At least 2	
15.	MVA cannula sizes 4-12	At least 2 each	
16.	MVA cannula number (14 & 16)	At least 2 each	
17.	MVA set	At least 2 Set	
18.	D&E set	At least 2 Set	
19.	Suture set with Long needle holder	At least 2	
20.	Combi-pack (Mifepristone and Misoprostol)		
21.	Misoprostol only to treat incomplete abortion		
22.	Antibiotics (Injection Metronidazole 500mg/100ml, Tab Azithromycin 500mg)	As per need	
23.	Uterotonics (Injection Oxytocin, Tablet Misoprotol, Injection ergometrine)	As per need	
24.	Injection Xylocaine 1% /2% without adrenaline	2 vail each	
25.	Injection Atropine	10 ampules	
26.	Injection Adrenaline	10 ampules	
27.	Injection Hydrocortisone	At least 3 vail	
28.	Injection Dexamethasone	At least 3 vail	
29.	Distilled Water (100ml)	At least 2 bottles	
30.	Gloves (disposable) for P/V examination	At least 2 box	
31.	Surgical gloves different size	At least 2 each	
32.	Betadine Solution	At least 1 bottle	
33.	Disposable syringes 2 ml, 5 ml, 10 ml, 20 ml	At least 5 each	
34.	ET tubes of different size	At least 2 of each size	
35.	IV fluids (Normal Saline 0.9%, Ringers; Lactate, Dextrose 5% Normal Saline 0.9%)	At least 5 each	
36.	IV Infusion set	At least 5	
37.	IV canula (18 Gz, 20Gz)	At least 2 each	
38.	Foley's catheter and Urobag,	At least 2 set	
39.	Sutures of different size	At least 5 each	
40.	Soft brush for cleaning equipments	At least 2	
41.	Bucket or Basin	2-3 each of different size	
42.	IP flex available for processing MVA aspirator and cannula	One	
		**Total score**	
		**Percentage = Total score/ 42 x 100**	

Each row gets a score of 1 in each row if is available otherwise 0

Scoring Chart	
**Total Percentage**	**Score**
0-50	0
50-70	1
70-85	2
85-100	3
**Score for Standard 2.2.3.7.1**	

Area	Code	Verification		
**Emergency Service**	**2.3.1**			
**Components**		**Service Standards**	**Obtained score**	**Maximum score**
2.3.1 Time for patients	2.3.1	Emergency room/ward is open 24 hours		1
2.3.2 Staffing (per shift in ER)	2.3.2.1.1	For 5-10 ER beds (Doctor: Nurse: Paramedics: Office Assistant = 1:1:1:1)		1
	2.3.2.1.2	For every increased 5 ER beds, proportionate additional health workers is done (Doctor: Nurse: Paramedics: Office Assistant = 1:1:1:1)		1
	2.3.2.2	There should be 1:1 nurse patient ration in red area, 1:3 in yellow area and 1:6 in green area.		1
	2.3.2.3	The doctor, nurse and paramedics are trained in PTC, ETM, BLS and ACLS[2] training		1
2.3.3 Physical facilities	2.3.3.1	10% of the total hospital beds are allocated for ER of which 1% for red, 2% for yellow, 3% for green and 1 % for black color coded		1
	2.3.3.2	Adequate furniture and supplies (See Annex 2.3a Furniture and General Supplies for ER At the end of this standard)		3
	2.3.3.3	Light and ventilation are adequately maintained.		1
	2.3.3.4	Designated area for nursing station centrally placed in ER and all beds visible from nursing station		1
	2.3.3.5	Space allocated for duty room and changing room separate for male and female staffs with facilities of tea room		1
	2.3.3.6	Separate toilets for staffs at least one each-male, female and universal		1
	2.3.3.7	Separate land line/ mobile phone for emergency		1
2.3.4 Instruments/ equipment	2.3.4	Instruments and equipment to carry out the ER works are available and functioning (See Annex 2.3b ER Instruments and Equipment At the end of this standard)		3

2 PTC- Primary Trauma Care, ETM- Emergency Trauma Management, BLS- Basic Life Support, ACLS- Advanced Cardiac Life Support

2.3.5 Medicines and supplies	2.3.5.1	Medicines and supplies to carry out the ER works are available (See Annex 2.3c Medicines and Supplies for ER At the end of this standard)		3
	2.3.5.2	Emergency stock of medicines and supplies for mass casualty management		1
2.3.6 Triage	2.3.6.1	Hospital maintains a triage system in the ER with 24 hours triage service		1
	2.3.6.2	Availability of at least two sets of triaging equipment- thermometer, stethoscope, BP instrument, Pulse Oxy-meter, glucometer		1
	2.3.6.3	Triage category board and information to the public (Red, Yellow, Green Board)		1
2.3.7 Emergency protocol in place	2.3.7.1	In red area one of the bed is Resuscitation bed with availability of emergency crash trolley with emergency lifesaving drugs, cardiac monitor, non-invasive ventilator, oxygen concentrator		1
	2.3.7.2	Development of 001 or Blue code call system whenever any patient visited in Emergency collapses and need immediate and urgent emergency care		1
	2.3.7.3	Emergency disposition of the patient either in observation ward or definite care ward or referral or discharge within 3-6 hours		1
	2.3.7.4	Critical patient transfer from emergency to OT or Inter-hospital transfer is accompanied at least by paramedics or Nurse for handover of patient		1
2.3.8 Maintaining patient privacy	2.3.8	Appropriate methods have been used to ensure patient privacy (separate rooms, curtains hung)		1
2.3.9 Security	2.3.9	The hospital has maintained security system for ER for 24 hours with CCTV coverage		1
2.3.10 Mass casualty/ disaster preparedness	2.3.10.1	The hospital has mass casualty management protocol, and all staffs are updated with well labelled direction, prepositioning clipboards		1
	2.3.10.2	Disaster area identified with adequate furniture to carry out Triage in case of disaster		1
	2.3.10.3	There must be disaster store in ER with required medicines, supplies and equipment (See Annex- List of medicine, supplies and equipment for Disaster Store)		3
	2.3.10.4	Hospital carries out at least one mock drill and disaster preparedness once a year		1
2.3.11 Duty rosters	2.3.11	Duty rosters of the ER are developed regularly and available in appropriate location		1
2.3.12 Maintaining inventory	2.3.12	Separate inventories for emergency lifesaving drugs/equipment and narcotics are maintained		1
2.3.13 Securing narcotic drugs	2.3.13	Narcotic drugs are kept separately and securely with mandatory recording system		1

2.3.14 Facilities for patients	2.3.14.1	Safe drinking water is available 24 hours		1
	2.3.14.2	Hand-washing facility with running water and liquid soap		1
	2.3.14.3	There are at least 3 toilets with hand-washing facilities (1 for males, 1 for females, and 1 universal) for every 10 ER beds and for additional beds increase proportionately for male and female		1
2.3.15 Decontamination area	2.3.15	Decontamination area specified and practiced		1
2.3.16 Infection prevention	2.3.16.1	Staff wear mask and gloves at work		1
	2.3.16.2	There are clearly labelled colored bins for waste segregation and disposal as per HCWM Guideline 2014 (MoHP)		1
	2.3.16.3	Needle cutter is used		1
	2.3.16.4	Chlorine solution is available and utilized for decontamination		1
**Standard 2.3**		**Total Score**		47
		**Total Percentage (Total Score/ 47 x100)**		

Annex 2.3a Furniture and General Supplies for ER

S.No.	Furniture and General Supplies	Required Quantity	Score
1.	Wheel chair	2 for every 5 ER beds	
2.	Trolley	1 for every 5 ER beds	
3.	Stretcher	1 for every 5 ER beds	
4.	Information board	1	
5.	Foot Step	2 for every 5 ER beds	
6.	Working Table/Station with 2 chairs	1	
7.	Stool (for visitor) each bed	1	
8.	Medicine Rack	1	
9.	Supplies Rack	1	
10.	Waste Bins (color coded and labelled as per guideline 2014 (MoHP))	1 set for every 5 ER beds	
11.	Poisoning Chart	1	
12.	Telephone set/Mobile	1	
13.	Reference Books with cupboard	1	
14.	Cup Board	1	
15.	Screen	As per need	
16.	Cart/Trolley with medicines for emergency procedures	1	
17.	IV stand	At least one per bed	
18.	Bed Pan	2 for every 5 bed	
19.	Urinal	2 for every 5 bed	
		**Total Score**	
		**Total Percentage = Total Score/19 X 100**	

Each row gets a score of 1 if all the required number is available otherwise 0.

Scoring chart	
**Total percentage**	**Score**
0-50	0
50-70	1
70-85	2
85-100	3
**Score for Standard 2.3.3.2**	

Annex 2.3b ER Equipment and Instrument

SN	Equipment /Instruments	Required No.	Score
1.	ECG machine (12 Leads)	1	
2.	Defibrillator	1	
3.	Foot / Electric Suction Machine	2	
4.	Portable ventilator/ Non-invasive ventilator	1	
5.	Positive Airway Pressure machine with accessories	1	
6.	Nebulizer set	1	
7.	Cardiac monitors with non-invasive BP cuffs	1 (For 5 beds)	
8.	BP set and Stethoscope (each treatment room)	2	
9.	Pulse oximeter	1	
10.	Glucometer with strips	1	
11.	Duck Speculum	2	
12.	Protoscope	2	
13.	Otoscope set	1	
14.	Nasal Speculum	1	
15.	Laryngoscope with batteries and blades	2	
16.	ET tubes of different sizes	At least 2 each	
17.	Torch Light	2	
18.	Geudel Airway	2	

19.	Ambu Bag (Adult and Pediatric)	2	
20.	Bougie	2	
21.	Endotracheal tube of different sizes	6	
22.	Different size mask	6	
23.	Laryngeal mask airway (Adult and Peadiatric)	1 each	
24.	Oxygen tubes and masks	10 each	
25.	Suture Set	4	
26.	Catheterization set	2	
27.	Dressing set	2	
28.	Water sealed drainage set	1	
29.	N/G tube Aspiration set	1	
30.	Ear Irrigation Set	1	
31.	Cervical collar	4	
32.	Spinal backboard	1	
33.	Splints	3	
34.	Arm Slings	3	
35.	Portable Light	2	
		**Total Score**	
		**Total Percentage = Total Score/34X 100**	

Each row gets a score of 1 if all the required number is available otherwise 0.

**Scoring chart**	
**Total percentage**	**Score**
0-50	0
50-70	1
70-85	2
85-100	3
**Score for Standard 2.3.4**	

Annex 2.3c Medicines and supplies for ER (number proportionate to ER beds 1:2)

SN	Name	Score
1.	Atropine Injection	
2.	Adrenaline Injection	
3.	Xylocaine 1% and 2% Injections with Adrenaline	
4.	Xylocaine 1% and 2 % Injections without Adrenaline	
5.	Xylocaine Gel	
6.	Diclofenac Injection	
7.	Hyoscine Butylbromide Injection	
8.	Diazepam injection	
9.	Morphine Injection / Pethidine Injection	
10.	Hydrocortisone Injection	
11.	Antihistamine Injection	
12.	Dexamethasone Injection	
13.	Ranitidine/Omeperazole Injection	
14.	Frusemide Injection	
15.	Dopamine injection	
16.	Noradrenaline injection	
17.	Digoxin injection	
18.	Verapamil injection	
19.	Amidarone injection	
20.	Glyceryl trinitrate injection/ tab	
21.	Labetolol injection	
22.	Magnesium Sulphate injection (loading dose)	
23.	Sodium bicarbonate injection	
24.	Calcium Gluconate injection	
25.	Ceftriaxone Injection	
26.	Metronidazole Injection	
27.	Charcoal Power	
28.	Normal Saline Injection	
29.	Ringers' Lactate Injection	

30.	Dextrose 5% Normal Saline Injection	
31.	Dextrose 5% Injection	
32.	Dextrose 25%/50% Injection (ampoule)	
33.	IV Infusion set (Adult/Pediatric)	
34.	IV Canula (16, 18, 20, 22, 24, 26 Gz)	
35.	Foley's Catheter (different French)	
36.	Disposable syringes (1 ml, 3 ml, 5 ml, 10 ml, 20 ml, 50 ml)	
37.	Disposable Gloves (Size- 6, 6.5, 7, 7.5)	
38.	Distilled Water	
39.	Sodium chloride-15%w/v and Glycerin-15% w/v (for enema)	
	**Total Score**	
	**Total Percentage = Total Score/39 X100**	

Each row gets a score of 1 if all the required number is available otherwise 0.

**Scoring chart**	
**Total percentage**	**Score**
0-50	0
50-70	1
70-85	2
85-100	3
**Score for Standard 2.3.5.1**	

Annex 2.3d List of equipment, instrument, medicine and supplies for Disaster Store

**S.No.**	**Equipment and Instruments**	**Required Number**	**Score**
1.	Stretcher/ trolley	5	
2.	Spinal boards	5	
3.	Wheel chairs	5	
4.	Medicine trolley	2	
5.	Portable suction machine	2	
6.	Nebulizer machine	2	

7.	Fluid warmer	2	
8.	Bp instrument	10	
9.	Stethoscope	10	
10.	Saturation probe	5	
11.	Thermometer	5	
12.	Suture sets	5	
13.	Dressing sets	5	
**MEDICINES**			
14.	Tetanus Toxoid Injection	50	
15.	Diclofenac Paracetamol Injection	50	
16.	Tramadol Injection	50	
17.	Ondensterone Injection	50	
18.	Cefazoline Injection	20	
19.	Metronidazole Injection	20	
20.	Ketorolac Injection	20	
21.	Transemic Acetate Injection	20	
22.	Atropine Injection	50	
23.	Adrenaline Injection	50	
24.	Midazolam Injection	20	
25.	Xylocaine 2% Injection	20	
26.	Vitamin B-complex Injection	20	
27.	Succinylcholine Injection	20	
28.	Normal Saline /Ringers' Lactate/ Dextrose 5% Normal Saline Injection	20 each	
29.	Hemaecel Injection	5	
**Intubation articles**			
30.	Ambu bag (adult,paed.)	10	
31.	Resuscitation masks (adult, pediatric, newborn)	10 each	
32.	ET tubes different size	5 each	
33.	Airways	5	
34.	Laryngoscope	5	
35.	ECG Leads	30	

SUPPLIES			
36.	Triage tags	100	
37.	Extra I/V stand	20	
38.	Portable oxygen cylinder	5	
39.	Plastic aprons	10	
40.	Gowns	10	
41.	Extra Mattress	50	
42.	Blankets	50	
43.	Screens	3	
44.	Scissors	5	
45.	Splints	15	
46.	Cervical collar(hard/soft)	20	
47.	Arm slings	20	
48.	Pelvic binder	5	
49.	Bandages	50	
50.	Crepe bandage	20	
51.	Elastoplast	20	
52.	Leuckoplast	20	
53.	Nebulizer kit set	2	
54.	Oxygen masks	10	
55.	PMO line	10	
56.	Chest tube set (No.28,32)	10	
57.	Drainage bag	20	
58.	Foleys catheter/ urobag	20 each	
59.	Surgical gloves Different Size	30 of each size	
60.	Examination gloves	2 boxes	
61.	Utility gloves	10	
62.	Betadine /spirit	10	
63.	IVCannulas of all size, IV set and Buret Set	20 each	
64.	Syringes of different size (3ml,5ml, and 10ml) / (20ml, 50ml)	50 each/ 20 each	
65.	Suction tubes different size	5 each	

66.	Yanker suction	1	
67.	Sterile gauze, cotton, dressing pads.	1 medium size steel drum each	
68.	Hand Sanitizer	20	
69.	Torch lights	5	
70.	Note book	10	
71.	Ballpens	10	
**Total score**			
**Total percentage = Total Score/ 71 x100**			

Each row gets a score of **1** if all the required number is available otherwise **0**.

**Scoring chart**	
**Total percentage**	**Score**
0-50	0
50-70	1
70-85	2
85-100	3
**Score for Standard 2.3.14.4**	

**Area**	**Code**	**Verification**		
**Emergency Minor OT**	**2.4**			
**Components**		**Service Standards**	**Obtained Score**	**Maximum Score**
2.4.1 Working space	2.4.1	A separate space dedicated for emergency minor operative procedures		1
2.4.2 Furniture & general supplies	2.4.2	Adequate furniture and general supplies are available (See Annex 2.4a Furniture and General Supplies for ER Minor OT At the end of this standard).		3
2.4.3 Services available	2.4.3	Minimum dressing services and routine procedures are available (See Annex 2.4b List of Minimum Services from ER Minor OT At the end of this standard).		3
2.4.4 Staffing	2.4.4	Duty roster prepared to assign staffs for emergency minor OT		1
2.4.5Disposable supplies	2.4.5	Medicines and supplies needed for surgical procedures available (SeeAnnex 2.4c Medicine and Supplies for Minor OT At the end of this standard).		3

2.4.6 Sterile supplies	2.4.6.1	Adequate quantity of sterilized packs for Minor OT are available (See Annex 2.4d Sterile Supplies for Minor OT At the end of this standard).		3
	2.4.6.2	Separate containers for sterile gauze and cotton balls are available.		1
2.4.7 Anesthesia services available	2.4.7.1	Minor OT has equipment, instrument and supplies for anesthesia services (See Annex 2.4e Equipment, Instrument and Supplies for Anesthesia for Minor OT At the end of this standard)		3
	2.4.7.2	Minor OT has medicines and supplies for anesthesia services (See Annex 2.4f Medicine and Supplies for Anesthesia for Minor OT At the end of this standard)		3
2.4.8 Infection prevention and waste disposal	2.4.8.1	Mask, gloves, plastic apron, boots and goggles are available and used whenever required.		1
	2.4.8.2	At least three color coded waste bins as per HCWM guideline 2014 (MoHP) are available and used		1
	2.4.8.3	Supplies trolley with needle cutter is available and used		1
	2.4.8.4	Hand-washing facility with running water and soap		1
	2.4.8.5	Chlorine solution is available and utilized for decontamination		1
2.4.9 Documentation	2.4.9	Proper records of all procedures are kept and reported.		1
**Standard 2.4**		**Total Score**		27
		**Total Percentage (Total Score/ 27 x100)**		

Annex 2.4a Furniture, Equipment, Instruments and Supplies for Minor OT

SN	General Equipment and Instruments for OT	Standard Quantity	Score
1.	Wheel chair foldable, adult size	As per need	
2.	Stretcher	As per need	
3.	Patient trolley	As per need	
4.	Cupboards and cabinets for store	As per need	
5.	Working desk for anesthesia, nursing station, gowning	1 each	
6.	OT Table- universal type/ with wedge to position patient	At least 1	
7.	Examining table	1	
8.	Mayo Stand with tray	2	
9.	Operation theatre lights	1	
10.	Ultra violet light source	1	
11.	Electronic suction machine/ Foot-operated suction machine	1/1	
12.	Refrigerator / cold box	1	

13.	Boiler/ Autoclave	1/1	
14.	Anesthesia machine with cardiac monitor	1	
15.	Cautery/Diathermy machine	1	
16.	Oxygen concentrator/ Oxygen Cylinder	1	
17.	Baby warmer	1	
18.	Baby weight machine	1	
19.	Anesthesia trolley	2	
20.	Instrument trolley	2	
21.	BP instrument with stethoscope	1	
22.	Thermometer	1	
23.	Steel Drum for gloves	1	
24.	Steel Drum for Cotton	1	
25.	Tourniquet, latex rubber, 75 cm	2	
26.	Kidney tray (600cc)	2	
27.	Covered instrument trays	4	
28.	Mackintosh sheet	1	
29.	Lead gown	2 sets	
30.	Bowl stand	2	
31.	Cheatle forceps in jar	2	
32.	Drapes for abdominal site<br>(laparotomy sheet, table cover, hook towel, mayo cover, plastic sheet, tetra)	As per need	
33.	Drapes for perineal region<br>(Laparotomy sheet, table cover, hook towel, mayo cover, plastic sheet, tetra, leggings)	As per need	
34.	Packing towel double wrapper	As per need	
35.	Sterile gloves (6,6.5,7,7.5,8)	5/5/5/5/5/5	
36.	Towels/ eye hole	As per need	
37.	Masks and caps	As per need	
38.	Torch light and batteries	1 set	
39.	Foot steps	2	
40.	Wall clock	1	
41.	Waste bucket for scrub nurse	1	

42.	IV stand	2	
43.	Leak proof sharp container	1	
44.	Generator back up for OT	1	
45.	Gas/ Kerosene stove- 4 burners	1	
46.	Color coded waste bins (based on HCWM guideline 2014 (MoHP))	1 set	
		**Total Score**	
		**Total percentage= Total/ 46 x 100**	

Each row gets a score of 1 if the mentioned test is available otherwise 0.

**Scoring Chart**	
**Total percentage**	**Score**
0-50	0
50-70	1
70-85	2
85-100	3
**Score for Standard 2.4.2**	

Annex 2.4b List of Minimum Services from ER Minor OT

**S.No.**	**List of Minimum Services from ER Minor OT**	**Score**
Minor		
1.	I & D under LA	
2.	Excision of cysts, ganglion, lump, lymhnode, lipoma, skin papilloma, corn under LA	
3.	Excision of ingrowing toe nail under digital block	
4.	Breast Abscess aspiration	
5.	Wound debridement	
6.	Skin suturing < 5cm size	
7.	Foreign Body removal under LA	
8.	Repair split ear	
9.	True cut biopsy	
10.	Chest tube insertion under LA	
11.	Circumcision Under LA	

12.	Eversion of sac for hydrocele (EVS)	
13.	Haemorrhoid banding	
Intermediate		
14.	Herniotomy under IVA	
15.	Mesh Repair / Darn Repair (under LA/SA)	
16.	Amputation	
17.	Large wound dressing / debridement under IVA/SA	
18.	Chest tube insertion under IVA	
19.	Circumcision under IVA	
20.	I & D under IVA eg. Breast abscess, perineal abscess	
21.	Haemorrhoidectomy	
**Total score**		
**Total Percentage= Total score/21 x 100**		

Each row gets a score of 1 if all the required number is available otherwise 0.

**Scoring chart**	
**Total percentage**	**Score**
0-50	0
50-70	1
70-85	2
85-100	3
**Score for Standard 2.4.3**	

Annex 2.4c Medicine and Supplies for Minor OT

SN	Emergency Drugs (including neonates) for OT	Standard Quantity for 1 patient	Score
1.	Midazolam Injection	5 vials	
2.	Hydrocortisone Powder for Injection	100ml 2 vial	
3.	Frusemide Injection	2 ampules	
4.	Dopamine Injection	5 vials	
5.	Transemic Acetate Injection	2 ampules	
6.	Hydralizine Injection	5 vials	
7.	Calcium Gluconate Injection	10ml X 2 ampules	

8.	Magnesium sulphate Injection	0.5 gms X 28	
9.	Oxytocin Injection	10 Ampules	
10.	Dextrose (25%) / (50%) Injection	2 ampules	
11.	Naloxone Injection	1 ampule	
12.	Aminophyline Injection	2 ampules	
13.	Chloropheniramine Injection	2 ampules	
14.	Mephentine Injection	1 vial	
15.	IV Fluids- Ringers Lactate / Normal Saline/ Dextrose 5% Normal Saline/ Dextrose 5%	6 bottles each	
16.	IV infusion Set	4	
17.	IV Canula 22G/20G/18G	4 each	
		**Total Score**	
		**Total Percentage = Total Score/17 X 100**	

Each row gets a score of 1 if all the required number is available otherwise 0

**Scoring Chart**	
**Total percentage**	**Score**
0-50	0
50-70	1
70-85	2
85-100	3
**Score for Standard 2.4.5**	

Annex 2.4d Sterile Supplies for Minor OT

**S.No.**	**Items**	**Required number**	**Score**
1.	Catheter set	At least 5	
2.	Suture set	At least 5	
3.	Dressing set of different size (small, medium, large)	At least 2 each	
4.	Incision and drainage set	At least 5	
5.	Laparotomy set	At least 2	
		**Total Score**	
		**Total Percentage= Total Score/5x100**	

Each row gets a score of 1 if all the required number is available otherwise 0

Scoring Chart	
**Total percentage**	**Score**
0-50	0
50-70	1
70-85	2
85-100	3
**Score for Standard 2.4.6.1**	

Annex 2.4e Equipment, Instrument and Supplies for Anesthesia for Minor OT

S.No.	List of equipment, instruments and supplies for anesthesia	Required Number	Score
1.	Supply of oxygen<br>(e.g., oxygen concentrator, cylinders or pipeline) with regulator and flow meter	At least 2 oxygen concentrator	
2.	Oropharyngeal airways<br>(Size 000, 00, 0, 1, 2, 3, 4)	At least 2 each	
3.	Anesthesia face masks<br>(Size 0, 1, 2, 3, 4)	At least 2 each	
4.	Laryngoscope, Mc Coy's curved blade and Miller's straight blade<br>(small, medium and large sizes for both adult and pediatric patients)	At least two	
5.	Endotracheal tubes, cuffed, uncuffed, different sizes (Sizes 2.5 - 8.0 ID)	At least two of each size	
6.	Intubation aids<br>(Magillsforcep of small and large size, bougie, stylets of small and large size)	As per need	
7.	Suction device and suction catheters of different sizes (Size 8 -16 Fr)	As per need	
8.	Adult and pediatric self-inflating bags<br>(Size 2L, 1L, 0.5L)	As per need	
9.	Equipment for intravenous infusions and injection of medications for adult and pediatric patients<br>(IV stand, IV canula, fixing tapes, infusion sets, blood transfusion sets, burette sets, syringes, three-way stop cocks)	As per need	
10.	Examination (non-sterile) gloves	As per need	
11.	Sterile gloves	As per need	
12.	Pulse oximeter	At least 2	
13.	Access to a defibrillator	At least 1	
14.	Stethoscope	At least 2	

15.	Sphygmomanometer with appropriate sized cuffs for adult and pediatric patients	As per need	
16.	Non-invasive blood pressure monitor with appropriate sized cuffs for adult and pediatric patients	As per need	
17.	Electrocardiogram - three leads	As per need	
18.	Temperature monitor (intermittent)	As per need	
		**Total Score**	
		**Total percentage = Total score/ 18 x 100**	

Each row gets a score of 1 if the mentioned test is available otherwise 0.

**Scoring chart**	
**Total percentage**	**Score**
0-50	0
50-70	1
70-85	2
85-100	3
**Score for Standard 2.4.7.1**	

Annex 2.4f Medicines and Supplies for Anesthesia for Minor OT

**S.No.**	**List of Medicines**	**Required Number**	**Score**
Preoperative medications			
1.	Ranitidine Injection	5	
2.	Metoclopramide Injection	5	
3.	Aluminium hydroxide or magnesium trisilicate suspension	5	
4.	Atropine Injection	10	
5.	Diazepam Tablet	5	
Intraoperative medications			
6.	Ketamine Injection	3	
7.	Midazolam Injection	3	
8.	Opioid analgesics injections (Morphine, Pethidine, Fentanyl)	2 each	
9.	Lignocaine 2% Injection for IV infusion	2	
10.	Lignocaine Inj 1%, 2% with or without Adrenaline 1:200000	2	

11.	Thiopental Powder 500mg	As per need	
12.	Propofol Injection	As per need	
13.	Appropriate inhalational anesthetic (Halothane, Isoflurane, Sevoflurane)	As per need	
14.	Succinylcholine Injection	As per need	
15.	Appropriate non-depolarizing muscle relaxant (Pancuronium, Vecuronium, Rocuronium, Atracurium Injections)	As per need	
16.	Neostigmine Injection	As per need	
17.	Atropine Injection / Glycopyrolate Injection	10/10	
18.	Bupivacaine Heavy 0.5%	2	
Intravenous fluids			
19.	Water for injection	As per need	
20.	Normal saline / Ringer's lactate	As per need	
21.	5% Dextrose / Dextrose normal saline	As per need	
22.	1/5Dextrose 1/3Normal saline	As per need	
23.	Mannitol Inj 20%	As per need	
24.	Haemaccel Injection / Gelafusine Injection / Voluven Injection	As per need	
Resuscitative medications			
25.	Dextrose 25%/ 50% Injection	5 each	
26.	Mephenteramine or Ephedrine Injection	5	
27.	Dopamine Injection	5	
28.	Noradrenaline Injection	5	
29.	Amiodarone Injection	5	
30.	Hydrocortisone Injection	5	
31.	Dexomethasone Injection	5	
32.	Chlorpheniramine Injection	5	
33.	Calcium gluconate Injection	5	
34.	Beta-blockers (Metoprolol, Labetolol, Esmolol) Injection	As per need	
35.	Naloxone Injection	5	
Post-operative medications		Post-operative medications	
36.	Morphine Injection	As per need	
37.	Pethidine Injection	As per need	

38.	Tramadol Injection	As per need	
39.	Pentazocine Injection	As per need	
40.	Paracetamol Injection 1gm, Suppository 125mg	As per need	
41.	Diclofenac Injection	As per need	
42.	Ketorolac Injection	As per need	
43.	Promethazine Injection	As per need	
44.	Ondansetron Injection	As per need	
45.	Gabapentin Injection	As per need	
Other medications		Other medications	
46.	Magnesium Injection	As per need	
47.	Salbutamol Injection (for inhalation)	As per need	
48.	Ipratropium bromide Injection (for inhalation)	As per need	
49.	Furosemide Injection	As per need	
50.	Glyceryl trinitrate/nitroglycerine Injection	As per need	
51.	Sodium nitroprusside Injection	As per need	
52.	Heparin Injection	As per need	
53.	Aminophylline Injection	As per need	
		**Total Score**	
		**Total percentage = Total score/ 53x 100**	

Each row gets a score of 1 if the mentioned test is available otherwise 0.

**Scoring chart**	
**Total percentage**	**Score**
0-50	0
50-70	1
70-85	2
85-100	3
**Score for Standard 2.4.7.2**	

Area	Code	Verification		
**Hospital Pharmacy Service**	**2.5**			
**Components**		**Service Standards**	**Obtained Score**	**Maximum Score**
2.5.1 Pharmacy department available	2.5.1	Hospital has designated pharmacy department		1
2.5.2 Governance committee for hospital pharmacy services	2.5.2.	Governance committee for hospital are formed based on hospital pharmacy-service guideline:		
	2.5.2.1	Drug and Therapeutic committee (DTC)		1
	2.5.2.2	Hospital pharmacy operation committee		1
2.5.3 Hospital formulary Heading: Availability of medicines and supplies	2.5.3.1	Hospital has hospital formulary based on Nepalese National Formulary (NNF) approved by DTC		1
	2.5.3.2	Hospital formulary includes all medicines and supplies as per services provided by hospital		1
	2.5.3.3	Hospital has all ,medicines and supplies available as per approved hospital formulary list		1
2.5.4 Good procurement practice	2.5.4.1	Annual procurement plan for medicines and supplies for pharmacy services is available		1
	2.5.4.2	Procurement is done based on public procurement guideline		1
	2.5.4.3	Product specification for each medicine and related supplies of approved formulary list is available		1
	2.5.4.4	Technical criteria on quality assurance of procured medicines is included in standard bidding document		1
	2.5.4.5	Certificate of analysis (CoA) from manufacturer of each batch of procured medicine is available		1
	2.5.3.4	Selling price of the drugs does not exceed 20% of the procurement price		1
2.5.4 Pharmacy service hours	2.5.4	The pharmacy is open 24x7		1
2.5.5 Staffing as per hospital pharmacy service guideline 2072	2.5.5.1	Pharmacy department is led by at least one clinical pharmacist		1
	2.5.5.2	Pharmacy has at least 3 pharmacist, 6 assistant pharmacist and 2 office assistants		1
	2.5.5.3	Duty roster of pharmacy to cover 24 hours service is prepared and visibly placed		1
2.5.7 Display of list of free medicines	2.5.7	The list of free medicines is displayed in a clearly visible place.		1
2.5.8 Availability of medicines for specific programs	2.5.8	All of the required medicines and supplies for specific programs are available in pharmacy (less than 50%= 0; 50-70 =1, 70-85=2 85-100= 3)		3

2.5.9 Inpatient pharmacy services available	2.5.9	Hospital pharmacy directly supplies inpatient medicine and supplies to wards and OT		1
2.5.10 Electronic record keeping	2.5.10	Pharmacy uses computer with software for inventory management and medicine use		1
2.5.12 Pharmacy stock available	2.5.12	Number of items of hospital formulary stocked in pharmacy(less than 50%= 0; 50-70 =1, 70-85=2, 85-100= 3)		3
2.5.13 Display and storage of medicines	2.5.13.1	All the medicines and supplies are displayed in clean racks following either alphabetical orders and generic names or grouping as use		1
	2.5.13.2	Temperature of pharmacy is monitored and recorded and is maintained in range of (25+/-2°C)		1
	2.5.13.3	Functional freeze +/-4°C for thermolabile medicine		1
2.5.14 Information to patients	2.5.14.1	Pharmacy department has its allocated separate information and counseling unit with reference books or e-books		1
	2.5.14.2	Information regarding the medicines is provided to the patients.		1
	2.5.14.3	IEC materials (posters, leaflets, national hospital formulary) about the appropriate use for medicines are available in the pharmacy area.		1
2.5.15 Generic prescription	2.5.15	Hospital has pre-printed list of medicines for generic prescription available		1
2.5.16 Dispensing medicines	2.5.16.1	Medicine is dispensed using electronic billing with barcode system		1
	2.5.16.2	Each medicine is given with written instructions on how to take		1
2.5.17 First Expiry First Out (FEFO)	2.5.17	FEFO system is maintained using standard stock book/cards.		1
2.5.18 Pharmacy Inventory	2.5.18	Every month, all medicines and supplies are counted, out- of-date discarded, and tallied with the medical store.		1
2.5.19 Drug utilization review and quantification of data	2.5.19.1	Pharmacy department operates pharmacovigilance activities and adverse drug reaction (ADR) Reporting		1
	2.5.19.2	Pharmacy department conducts studies on drug utilization and quantification		1
	2.5.19.3	Antimicrobial stewardship programme , proper antimicrobial utilization review and provide data on antimicrobial use		1
2.5.20 Pharmaceutical waste disposal	2.5.20	Pharmaceutical waste (expired or unused pharmaceutical products, spilled contaminated pharmaceutical products surplus drugs, vaccines or sera, etc) management is done based on HCWM guideline 2014 (MoHP) or returned to the supplier on time		1
**Standard 2.5**		**Total Score**		40
		**Total Percentage (Total Score/40 x100)**		

Area	Code	Verification		
**Inpatient Service**[3]	**2.6**			
**Components**		**Service Standards**	**Obtained Score**	**Maximum Score**
2.6.1 Space for work	2.6.1.1	Separate space for nursing station is available in each ward (See Checklist 2.6 At the end of this standard for scoring)		3
	2.6.1.2	Separate changing room available for male and female staffs (See Checklist 2.6 At the end of this standard for scoring)		3
	2.6.1.3	Separate store room is available (See Checklist 2.6 At the end of this standard for scoring)		3
	2.6.1.4	One ward should not exceed 25 beds for general ward		1

3 Separate Set of Sheets for Assessment of Service Standards including - Checklist and Annexes, should be used for Each Ward Allocated for Inpatient Service and scoring of cumulative standards is done at end of all inpatient wards' assessment

	2.6.2	Furniture and supplies to carry out the inpatient services are available and functioning		
	2.6.2.1	**Medicine Ward** (See Annex 2.6a Furniture and supplies for inpatient wards At the end of this standard)		3
	2.6.2.2	**Surgery Ward**(See Annex 2.6a Furniture and supplies for inpatient wards At the end of this standard)		3
	2.6.2.3.1	**Pediatrics Ward** (See Annex 2.6a Furniture and supplies for inpatient wards At the end of this standard)		3
	2.6.2.3.2	Separate area dedicated for play room with play materials for different pediatric age groups		1
	2.6.2.4	**Orthopedics ward** (See Annex 2.6a Furniture and supplies for inpatient wards At the end of this standard)		3
	2.6.2.5.1	**Psychiatry ward** (See Annex 2.6a Furniture and supplies for inpatient wards At the end of this standard)		3
	2.6.2.5.2	Separate area dedicated for recreational activities for psychiatry patients		1
2.6.2 Furniture and supplies available and functioning	2.6.2.5.3	Separate space designated for ECT procedure with treatment bed, ECT machine, emergency trolley with medicines and supplies		1
	2.6.2.6	**ENT Ward** (See Annex 2.6a Furniture and supplies for inpatient wards At the end of this standard)		3
	2.6.2.7	**PNC and Gynecology Ward** (See Annex 2.6a Furniture and supplies for inpatient wards At the end of this standard)		3
	2.6.2.8	**Burn/Plastic Ward** (See Annex 2.6a Furniture and supplies for inpatient wards At the end of this standard)		3
	2.6.2.8.1	Separate space designated for physiotherapy with walker, weight lifting and cycle exercise facility for burn inpatients		1
	2.6.2.8.2	Separate OT is designated for burn		1
	2.6.2.8.3	Separate ICU is designated for burn patient		1
	2.6.2.8.4	Separate space designated for shower (bathing) for burn patient		1
	2.6.2.9	**Geriatrics Ward** (See Annex 2.6a Furniture and supplies for inpatient wards At the end of this standard)		3

2.6.3 Medicine and supplies available	2.6.3	Medicine and supplies to carry out the inpatient services are available in wards		
	2.6.3.1	Medicine Ward (See Annex 2.6b medicine and supplies for inpatient wards At the end of this standard)		3
	2.6.3.2	Surgery Ward (See Annex 2.6b medicine and supplies for inpatient wards At the end of this standard)		3
	2.6.3.3	Pediatrics Ward (See Annex 2.6b medicine and supplies for inpatient wards At the end of this standard)		3
	2.6.3.4	Orthopedics ward (See Annex 2.6b medicine and supplies for inpatient wards At the end of this standard)		3
	2.6.3.5	Psychiatry ward (See Annex 2.6b medicine and supplies for inpatient wards At the end of this standard)		3
	2.6.3.6	ENT Ward (See Annex 2.6b medicine and supplies for inpatient wards At the end of this standard)		3
	2.6.3.7	PNC and Gynecology Ward (See Annex 2.6b medicine and supplies for inpatient wards At the end of this standard)		3
	2.6.3.8	Burn/Plastic Ward(See Annex 2.6b medicine and supplies for inpatient wards At the end of this standard)		3
	2.6.3.9	Geriatrics Ward (See Annex 2.6b medicine and supplies for inpatient wards At the end of this standard)		3
2.6.4 Nursing station	2.6.4	There is a set of at least one table and two chairs in the nursing station and shelves for storage of charts and inpatient forms and formats (See Checklist 2.6 At the end of this standard for scoring)		3
2.6.5 Nursing staff for inpatient service	2.6.5	Adequate numbers of nursing staff are available in ward per shift (nurse patient ratio 1:6 in general ward, 1:4 in pediatric ward, 1:2 in high dependency or intermediate ward or post-operative ward or burn/plastic) and at least one trained office assistant/ward attendant per shift in each ward (See Checklist 2.6 At the end of this standard for scoring)		3
2.6.6 Duty rosters	2.6.6	Duty roster of doctors, nurses, paramedics and support staffs kept visibly in nursing station (See Checklist 2.6 At the end of this standard for scoring)		3
2.6.7 Communication	2.6.7	Telephone facility is available with list of important contact numbers and hospital codes visibly kept (See Checklist 2.6 At the end of this standard for scoring)		3

2.6.8 Emergency management of inpatients	2.6.8.1	All staffs in wards are trained for BLS and oriented about emergency code 001 or blue code (See Checklist 2.6 At the end of this standard for scoring)		3
	2.6.8.2	At least one emergency trolley with emergency medicine available in ward		
	2.6.8.2.1	Medicine Ward (See Annex 2.6c Emergency Trolley for Inpatient At the end of this standard)		3
	2.6.8.2.2	Surgery Ward (See Annex 2.6c Emergency Trolley for Inpatient At the end of this standard)		3
	2.6.8.2.3	Pediatrics Ward (See Annex 2.6c Emergency Trolley for Inpatient At the end of this standard)		3
	2.6.8.2.4	Orthopedics ward (See Annex 2.6c Emergency Trolley for Inpatient At the end of this standard)		3
	2.6.8.2.5.1	Psychiatry ward (See Annex 2.6c Emergency Trolley for Inpatient At the end of this standard)		3
	2.6.8.2.5.2	ECT room (See Annex 2.6c Emergency Trolley for Inpatient At the end of this standard)		3
	2.6.8.2.6	ENT Ward (See Annex 2.6c Emergency Trolley for Inpatient At the end of this standard)		3
	2.6.8.2.7	PNC and Gynecology Ward(See Annex 2.6c Emergency Trolley for Inpatient At the end of this standard)		3
	2.6.8.2.8	Burn/plastic Ward (See Annex 2.6c Emergency Trolley for Inpatient At the end of this standard)		3
	2.6.8.2.9	Geriatrics Ward (See Annex 2.6c Emergency Trolley for Inpatient At the end of this standard)		3
	2.6.8.3	At least one defibrillator in immediate accessible area (See Checklist 2.6 At the end of this standard for scoring)		3
2.6.9 Safe Abortion Service (SAS) available	2.6.9	Safe abortion service (SAS) is available as per National SAS Implementation Guideline		1

2.6.10 Physical facilities for patient	2.6.10.1	Separate area designated for admission of male and female inpatients in wards (See Checklist At the end of this standard for scoring)		3
	2.6.10.2	There are adequate separate toilets for male and female patients in each ward (1 for 6 female bed and 1 for 8 male beds) and also adequate wash basins/sinks for the patients. (See Checklist 2.6 At the end of this standard for scoring)		3
	2.6.10.3	Patient safety in taken care of in all inpatient wards including proper fixation of the furniture and equipment		1
	2.6.10.4	Separate waiting area for visitors.		1
	2.6.10.5	For psychiatry ward, ensure that there is special arrangements for securing all furniture, equipment and instrument; all doors with no internal laches or locks, all the windows have grills and half doors in toilets and bathrooms for visible head and foot parts from outside		1
	2.6.10.6	Safe drinking water is available 24 hours for inpatients(See Checklist 2.6 At the end of this standard for scoring)		3
	2.6.10.7	Hours/ Time allocated for visitors to meet the inpatients and controlled traffic to prevent cross infection (See Checklist 2.6 At the end of this standard for scoring)		3
	2.6.10.8	Separate space is available for patients' visitors (KuruwaGhar).		1
2.6.11 Communication and counselling	2.6.11.1	Basic information regarding admitted patients is displayed in a separate board (See Checklist 2.6 At the end of this standard for scoring)		3
	2.6.11.2	Separate space with privacy dedicated for regular counselling is done for patient and patient party on condition and disease of patient (See Checklist 2.6 At the end of this standard for scoring)		3
2.6.12 IEC/BCC Materials	2.6.12	Appropriate IEC/BCC materials (posters, leaflets etc.) are available in the inpatient ward with focus on infection prevention (See Checklist 2.6 At the end of this standard for scoring)		3
2.6.13 Recording and reporting	2.6.113	Admission and discharge registers are available and are being filled completely (HMIS 8.1 and 8.2) (See Checklist 2.6 At the end of this standard for scoring)		3

2.6.14 Infection prevention	2.6.141	PPE are available and used whenever required (See Checklist 2.6 At the end of this standard for scoring)		3
	2.6.14. 2	Each ward has hand sanitizer in visible place for health workers to use before and after touching patients (See Checklist 2.6 At the end of this standard for scoring)		3
	2.6.14.3	There are well labelled color coded bins for waste segregation and disposal as per HCWM guideline 2014 (MoHP) (See Checklist 2.6 At the end of this standard for scoring)		3
	2.6.14.4	Hand-washing facility with running water and liquid soap is available and being practiced (See Checklist 2.6 At the end of this standard for scoring)		3
	2.6.14.5	Needle cutter is used (See Checklist 2.6 At the end of this standard for scoring)		3
	2.6.14..6	Chlorine solution is available and utilized for decontamination (See Checklist 2.6 At the end of this standard for scoring)		3
	2.6.14.7	Separate isolation room for any communicable disease patients		1
**Standard 2.6**		**Total Score**		165
		**Total Percentage (Total Score/ 165x100)**		

Checklist 2.6 Inpatient Services

(1= Medicine Ward 2= Surgery ward 3= Pediatrics Ward 4= Orthopedics ward 5=Psychiatry Ward 6= ENT ward 7= PNC Ward, 8= Burn/ plastic Ward, 9= Geriatrics Ward)

Code	Service Standards	1	2	3	4	5	6	7	8	9	Total Score	Percentage	Scoring	Direction to Use
2.6.1.1	Separate space for nursing station is available in each ward													Go to Standard 2.6.1.2
2.6.1.2	Separate changing room available for male and female staffs													Go to Standard 2.6.1.3
2.6.1.3	Separate store room is available													Go to Standard 2.6.2
2.6.4	There is a set of at least one table and two chairs in the nursing station and shelves for storage of charts and inpatient forms and formats													Go to Standard 2.6.5

2.6.5	Adequate numbers of nursing staff are available in ward per shift (nurse patient ratio 1:6 in general ward, 1:4 in pediatric ward, 1:2 in high dependency or intermediate ward or post operative or burn/plastic ward) and at least one trained office assistant/ward attendant per shift in each ward												Go to Standard 2.6.6
2.6.6	Duty roster of doctors, nurses, paramedics and support staffs kept visibly in nursing station												Go to Standard 2.6.7
2.6.7	Telephone facility is available with list of important contact numbers and hospital codes visibly kept												Go to Standard 2.6.8
2.6.8.1	All staffs in wards are trained for BLCS and oriented about emergency code 001 or blue code												Go to Standard 2.6.8.2
2.6.8.3	At least one defibrillator in immediate accessible area												Go to Standard 2.6.9
2.6.10.1	Separate area designated for admission of male and female inpatients in ward												Go to Standard 2.6.10.2
2.6.10.2	There are adequate separate toilets for male and female patients in each ward (1 for 6 female bed and 1 for 8 male beds) with wash basins/ sinks for the patients.*												Go to Standard 2.6.10.3
2.6.10.3	Safe drinking water is available 24 hours for inpatients												Go to Standard 2.6.10.4

2.6.10.5	Hours/ Time allocated for visitors to meet the inpatients and controlled traffic to prevent cross infection													Go to Standard 2.6.10.6
2.6.10.6	Separate space is available for patients' visitors (KuruwaGhar).													Go to Standard 2.6.11
2.6.11.1	Basic information regarding admitted patients is displayed in a separate board													Go to Standard 2.6.11.2
2.6.11.2	Separate space with privacy dedicated for regular counselling is done for patient and patient party on condition and disease of patient. (See Checklist 2.6).													Go to Standard 2.6.12
2.6.12	Appropriate IEC/ BCC materials (posters, leaflets etc.) are available in the inpatient ward with focus on infection prevention													Go to Standard 2.6.13
2.6.13	Admission and discharge registers are available and are being filled completely (HMIS 8.1 and 8.2)													Go to Standard 2.6.14
2.6.14.1	PPE are available and used whenever required													Go to Standard 2.6.14.2
2.6.14.2	Each ward has hand sanitizer in visible place for health workers to use before and after touching patients													Go to Standard 2.6.14.3
2.6.14.3	There are well labelled color-coded bins for segregation and disposal of waste as per HCWM guideline 2014 (MoHP)													Go to Standard 2.6.14.4

2.6.14.4	Hand-washing facility with running water and liquid soap is available and being practiced													Go to Standard 2.6.14.5
2.6.14.5	Needle cutter is used													Go to Standard 2.6.14.6
2.6.14.6	Chlorine solution is available and utilized for decontamination													Go to Standard 2.6.14.7
2.6.14.7	Separate isolation room for any communicable disease patients													Score Standard 2.6

Each row gets a score of 1 if the mentioned medicines are available otherwise 0.

Total Percentage = Total Score/ No of wards (8) x100

Plot the scoring based on the scoring chart and fill in the respective standards in tool and checklist

Scoring Chart	
**Total Percentage**	**Score**
0-50	0
50-70	1
70-85	2
85-100	3

* For geriatrics ward, commode in toilet and railing on wall in both toilet and bathroom must be kept.

Annex 2.6a Furniture and Supplies for inpatient wards
(1= Medicine Ward 2= Surgery ward 3= Pediatrics Ward 4= Orthopedics Ward
5=Psychiatry Ward 6= ENT Ward 7= PNC Ward 8= Burn/Plastic Ward 9= Geriatrics Ward)

SN	General Items	Required Number	Score								
			1	2	3	4	5	6	7	8	9
1.	Working table	1-2									
2.	Chairs	2									
3.	Cup board	2									
4.	Shelves	1									
5.	Bed side table	per bed-1									
6.	Stools (for visitor)	per bed 1									
7.	Patient Beds (Metal bed / adjustable head/ mechanical ratchet, 3 X 6 ft.)	As per sanctioned bed (fixed (immobile) for psychiatry ward) (for geriatrics, pneumatic beds/ geriatrics friendly bed)									
8.	Bed cradle	As per sanctioned bed									
9.	Cardiac monitor	As per sanctioned bed									
10.	Hot air blower	At least 1									
11.	Hair dryer	At least 1									
12.	Radio or Television for patients	At least 1 audible/visible from beds									
13.	Pneumatic tourniquet with different cuff size	At least 1									
14.	IV stand	As per bed									
15.	Medicine trolley	1									
16.	Dressing trolley	1									
17.	Wall Clock	2									
18.	OxygenConcentrator	1 per 5 bed									
19.	Suction machine (foot/ electric)	1									
20.	Refrigerator	1									
21.	Laryngoscope with blade and batteries	1									
22.	ET tubes of different sizes	At least 2 each									
23.	Self-inflating bag air mask – adult, child, neonate size	1 set									
24.	BP set and stethoscope (Non-Mercury)	2 sets									
25.	Thermometer	3-5									
26.	Baby and adult weighing scale	1 each									

27.	Nasal speculum set and otoscope	1 each									
28.	Plaster cutter	At least 1									
29.	Steel drum with sterile cotton	1									
30.	Steel drum with sterile gauze and pad	1									
31.	Scissors	2									
32.	Cheatle Forceps with Jar	2									
33.	Catheter set	2									
34.	Dressing set	At least 10									
35.	Minor-set for burn	At least 2									
36.	Mattress with bedcover, pillow with pillow cover, blanket with cover	1 set per bed									
37.	Torch with extra batteries and bulb	2-3									
38.	Inpatient register/ entered per ICD code	As per need (1)									
39.	Inventory Records/ entered per ICD code	As per need (1)									
40.	Cardex files	As per bed									
41.	Waste bins color coded based on HCWM guideline 2014 (MoHP)	1 set per room									
**Total Score**											
Maximum Score			31	31	31	32	31	32	31	38	31
**Total percentage= Total Score/Maximum Score x 100**											

Each row gets a score of 1 if the mentioned medicines are available otherwise 0.

**Scoring Chart**	
**Total Percentage**	**Score**
0-50	0
50-70	1
70-85	2
85-100	3
**Score for Standard 2.6.2.1**	
**Score for Standard 2.6.2.2**	
**Score for Standard 2.6.2.3.1**	
**Score for Standard 2.6.2.4**	
**Score for Standard 2.6.2.5**	
**Score for Standard 2.6.2.6**	
**Score for Standard 2.6.2.7**	
**Score for Standard 2.6.2.8**	
**Score for Standard 2.6.2.9**	

Annex 2.6b Medicine and Supplies for Inpatient Ward
(1= Medicine Ward 2= Surgery ward 3= Pediatrics Ward 4= Orthopedics ward, 5= Psychiatry ward, 6= ENT ward 7= PNC Ward 8=Burn/plastic Ward, 9 = Geriatrics Ward)

**S.No.**	**Medicine and supplies**	**Required No.**	**Score**								
			**1**	**2**	**3**	**4**	**5**	**6**	**7**	**8**	**9**
1.	NS	15									
2.	D5%	15									
3.	RL	15									
4.	DNS	15									
5.	Distilled Water	10									
6.	IV Infusion Set	10									
7.	Blood Transfusion set	5									
8.	IV Canula (16,18,20,22,24,26Gz)	5 each									
9.	Gloves (Utility)	As per need									
10.	Mask, Cap, Gowns	1 box									
11.	Mask, Cap, Gowns	As per need									
12.	Disposable syringes 1 ml, 3 ml, 5 ml, 10 ml, 30 ml, 50 ml	As per need									
13.	Needle 18-25	As per need									
14.	Traction set with different weights	As per need									
15.	Restrain set including belts (magnetic preferred)	As per need									
16.	Epistaxis management set	At least 2									
17.	Condom tamponed set	At least 2									
18.	Ophthalmoscope*	1									
19.	Negative pressure vacuum machine										
20.	Silver dressing										
21.	Collagen dressing										
		**Total Score**	**14**	**14**	**13**	**14**	**14**	**14**	**14**	**16**	**14**
		Maximum Score									
**Total Percentage = Total Score/ Maximum Score x 100**											

Each row gets a score of 1 if the mentioned medicines are available otherwise 0.
*Required for neurosurgery and neurology

Scoring Chart	
**Total Percentage**	**Score**
0-50	0
50-70	1
70-85	2
85-100	3
**Score for Standard 2.6.3.1**	
**Score for Standard 2.6.3.2**	
**Score for Standard 2.6.3.3**	
**Score for Standard 2.6.3.4**	
**Score for Standard 2.6.3.5**	
**Score for Standard 2.6.3.6**	
**Score for Standard 2.6.3.7**	
**Score for Standard 2.6.3.8**	
**Score for Standard 2.6.3.9**	

Annex 2.6c Medicines and Supplies for ER Trolley for Inpatient Ward
1= Medicine Ward, 2= Surgery Ward, 3= Pediatrics Ward 4= Orthopedics Ward, 5= Psychiatry Ward, 6= ECT Room 7= ENT ward 8 = PMC Ward 9= Burn/plastic ward, 10 = Geriatrics Ward

SN	Name	Required No	Score									
			1	2	3	4	5	6	7	8	9	10
1.	Atropine Injection	10										
2.	Adrenaline Injection	3										
3.	Xylocaine 1% and 2% Injections with Adrenaline	2										
4.	Xylocaine 1% and 2 % Injections without Adrenaline	2										
5.	Xylocaine Gel	2										
6.	Diclofenac Injection	5										
7.	Hyoscine Butylbromide Injection	5										
8.	Diazepam injection	2										
9.	Morphine Injection / Pethidine Injection	2										

10.	Hydrocortisone Injection	4										
11.	Antihistamine Injection	4										
12.	Dexamethasone Injection	4										
13.	Ranitidine/Omeperazole Injection	4										
14.	Frusemide Injection	5										
15.	Dopamine injection	2										
16.	Noradrenaline injection	2										
17.	Digoxin injection	2										
18.	Verapamil injection	2										
19.	Amidarone injection	2										
20.	Glyceryltrinitrate injection	1										
21.	Labetolol injection	1										
22.	Sodium bicarbonate injection	2										
23.	Tablet Ketamine	5-10										
24.	Phenytoin* Injection	5-10										
25.	Sodium Valporate* Injection	5-10										
26.	Phenobarbitone* Injection	5-10										
27.	Levetiraceta* Injection	2-5										
28.	Ceftriaxone Injection	4										
29.	Metronidazole Injection	4										
30.	Dextrose 25%/50% ampoule	2										
31.	IV Infusion set (Adult/Pediatric)	2										
32.	IV Canula (16, 18, 20, 22, 24, 26 Gz)	2 each										
33.	Disposable syringes 1 ml, 3 ml, 5 ml, 10 ml, 20 ml, 50 ml	5 each										
34.	Disposable Gloves (Size 6, 6.5, 7, 7.5)	3 each										
35.	Distilled Water	3										
36.	Free needles (18-24Gz)	As per need										
37.	Sodium chloride-15%w/v and Glycerin-15% w/v (for enema)	5										
**Total Score**												
Maximum Score			36	36	32	32	32	32	32	32	33	32
**Total Percentage = Total Score/Maximum ScoreX100**												

Each row gets a score of 1 if all the required number is available otherwise 0.
*For neurology and neurosurgery inpatients

Scoring chart	
**Total percentage**	**Score**
0-50	0
50-70	1
70-85	2
85-100	3
**Score for Standard 2.6.8.2.1**	
**Score for Standard 2.6.8.2.2**	
**Score for Standard 2.6.8.2.3**	
**Score for Standard 2.6.8.2.4**	
**Score for Standard 2.6.8.2.5.1**	
**Score for Standard 2.6.8.2.5.2**	
**Score for Standard 2.6.8.2.6**	
**Score for Standard 2.6.8.2.7**	
**Score for Standard 2.6.8.2.8**	
**Score for Standard 2.6.8.2.9**	

**Area**	**Code**	**Verification**		
**Maternity Services**	**2.7**			
**Delivery Services**	**2.7.1**			
**Components**		**Standards**	**Obtained Score**	**Maximum Score**
2.7.1.1 Availability of delivery service	2.7.1.1.1	Separate pre-labor room/ labor room with privacy is available.		1
	2.7.1.1.2	Delivery service is available round the clock		1
	2.7.1.1.3	At least one delivery bed is assigned for every 15 maternity beds		1
	2.7.1.1.4	Labor room has adequate space for accommodating team of health workers during emergencies and easy access to OT		1
	2.7.1.1.5	Separate OT for Obstetric Emergencies is available		1

2.7.1.2 Trained Human Resource for Delivery Services	2.7.1.2.1 Hospital delivery service has adequate and trained staffing			
	2.7.1.2.1.1	Nurse: pregnant women ratio 1:2 in pre-labor; 2:1 per delivery table and 1:6 in post-natal ward		1
	2.7.1.2.1.2	At least one ASBA trained medical officer on duty		1
	2.7.1.2.1.3	At least one office assistant is available per shift		1
	2.7.1.2.2	All staffs- nursing, medical practitioner designated for delivery services are trained skilled birth attendants		1
2.7.1.3 Duty rosters	2.7.1.3	Duty roster to cover 24 hours shift is developed and placed in visible place		1
2.7.1.4 Appropriate use of partograph for decision making	2.7.1.4	Partograph available and being used rationally		1
2.7.1.5 KMC done for low birth weight babies	2.7.1.5	At least 2 KMC chairs available for providing KMC to premature and preterm babies		1
2.7.1.6 Birth certificate prepared and released	2.7.1.6	A formally signed standard birth certificate is issued.		1
2.7.1.7 Patient counseling	2.7.1.7.1	Pre-labor/ during labor patient and patients' family are adequately given counseling on labor, possible complications and written consent taken		1
	2.7.1.7.2	Health education on PNC, danger signs of mother and child, Immunization, nutrition, hygiene and family planning is given		1
	2.7.1.7.3	Postpartum family planning and breastfeeding- early, exclusive and extended counseling is done prior to discharge.		1
2.7.1.8 IEC/BCC [4] materials	2.7.1.8	Appropriate IEC/BCC materials (posters, leaflets etc.) on postnatal care, breastfeeding-early, exclusive and extended, nutrition, immunization are used and available for users		1
2.7.1.9 Furniture, equipment, instrument, medicine and supplies for labor room	2.7.1.9.1	Separate store room for delivery service related logistics		1
	2.7.1.9.2	The facility has adequate equipment, instrument and general supplies for delivery services (See Annex 2.7.1a Furniture, equipment, instrument and general supplies for labor room At the end of this standard)		3
	2.7.1.9.3	Labor room has medicines and supplies available for delivery services (See Annex 2.7.1bmedicines and supplies for Labor Room At the end of this standard)		3
	2.7.1.9.4	Labor room has emergency cart with medicines and supplies available (See Annex 2.7.1c Medicines and Supplies for ER[5] Trolley Labor Room At the end of this standard)		3

4 IEC/BCC= Information Education and Communication/ Behavior Change Communication

5 ER= Emergency

2.7.1.10 Facilities for patients	2.7.1.10.1	Safe drinking water is available 24 hours.		1
	2.7.1.10.2	Separate toilet for patient is available in pre-labor room and accessible to patient after delivery		1
	2.7.1.10.3	There should be maternity waiting homes[6] where there is more than 20 deliveries per day and the waiting home must be taken round by every shift with at least one visit (by nurse)		1
2.7.1.11 Infection prevention	2.7.1.11.1	Personal protective equipment are available and used whenever required.		1
	2.7.1.11.2	Washable labor room		1
	2.7.1.11.3	Separate slipper designated for labor room and hand sanitizer placed in visible place for use		1
	2.7.1.11.4	There are at well labelled color-coded bins for waste segregation and disposal as per HCWM guideline 2014 (MoHP)		1
	2.7.1.11.5	Hand-washing facility with running water and liquid soap is available		1
	2.7.1.11.6	Needle cutter is used		1
	2.7.1.11.7	Liquid sodium hypochoride (0.5% Chlorine solution) is available and utilized for decontamination.		1
	2.7.1.11.8	Dry gauze and cotton are stored separately in clean containers.		1
	2.7.1.11.9	Separate bowls/ bucket for placenta and plastic		1
	2.7.1.11.10	Placenta pit is used to dispose placenta.		1
**Standard 2.7.1**		**Total Obtained Score**		40
		**Total Percentage = Total Obtained Score/ 40 x100**		

Annex 2.7.1a Furniture, equipment, instrument and general supplies for labor room

**S.No.**	**Items**	**Required Number**	**Score**
1.	Delivery bed	At least 1 for every 15 beds	
2.	Clean bed linen	Each bed	
3.	Curtains	As per need	
4.	Clean surface (for alternative delivery position)	Available	
5.	Newborn Resuscitation table	1	
6.	Light source	1	
7.	Room Heater	1	
8.	Baby heater	1 per delivery bed	
9.	Refrigerator for labor room	1	

6 Only for selected remote mountainous area as defined by government

Equipment and Instruments			
10.	BP Set and Stethoscope	1	
11.	Body Thermometer (Non- mercury)	1	
12.	Room thermometer	1	
13.	Fetoscope	2	
14.	Fetal stethoscope	1	
15.	Baby weighing scale	1	
16.	Self-inflating bag air mask - neonatal size	1	
17.	Mucus extractor with suction tube/ (Penguin)	2	
18.	Doppler	1	
19.	Vaginal speculum (Sims)	2	
20.	Neonatal resuscitation kit	1	
21.	Adult resuscitation kit	1	
22.	Sterile Delivery Instrument Set (Check each set)	4 sets per delivery beds	
22.1	Sponge forceps	2	
	Artery forceps	2	
	S/S bowl (Galli pot)	1	
	S/S bowl (receive placenta) (1-2 litre)	1	
	Cord cutting Scissors (blunt end)	1	
	Cord ties/ cord clamp	2	
	Plastic sheet/ rubber sheet	1	
	Gauze swabs	4	
	Cloth squared	3	
	Kidney tray	1	
	Peripad/ big dressing pad	2	
	Leggings	2	
	Perineal sheet	1	
	Baby receiving towel	1	
	Sterile gown	1	
23.	Suture set (Check each set)	2 sets per delivery beds	
23.1	Needle holder	1	
	Sponge holder	1	
	Suture cutting scissors	1	
	Dissecting forceps (tooth and plain)	2	
	Artery forceps	1	
	Galliport	2	
24	Episiotomy set (Check each set)	2 sets per delivery beds	

24.1	Episiotomy scissors	1	
	Needle holder	1	
	Suture cutting scissor	1	
	Dissecting forceps(tooth and plain)	2	
	Artery forceps	1	
25.	Vacuum set	2	
	Forceps set for delivery	1	
		**Total Score**	
		**Total percentage= Total Score/26x100**	

Each row gets a score of 1 if the mentioned medicines are available otherwise 0.

**Scoring Chart**	
**Total percentage**	**Score**
0-50	0
50-70	1
70-85	2
85-100	3
**Score for Standard2.7.9.2**	

Annex 2.7.1b Medicines and Supplies for Labor Room

S.No.	Medicines and supplies	Required No.	Score
Medicines			
1.	Oxytocin injection (keep in 2-8°C)	20 amp	
2.	Tranexamic acetate injection	10 amp	
3.	Ergometrine injection	10 amp	
4.	Magnesium sulphate injection	50 amp	
5.	Calcium gluconate injection	10 amp	
6.	Diazepam injection	10	
7.	Labetolol injection	10	
8.	Ampicillin injection	10	
9.	Gentamycin injection	5	
10.	Metronidazole injection	5	
11.	Lignocaine injection	2	
12.	Adrenaline injection	5	
13.	Ringers' lactate injection	10	
14.	Normal saline injection	10	
15.	Dextrose 5% injection	10	

16.	Water for injection	5	
17.	Eye antimicrobial (1% silver nitrate or Tetracycline 1% eye ointment)	2	
18.	Povidone iodine	5	
19.	Tetracycline 1% eye ointment	2	
20.	Paracetamol Tablet	20	
21.	Nefidipine SL Tablet 5 mg	4 tab	
22.	Misoprostol Tablet	5 tabs	
Supplies			
23.	Syringes and needles	20	
24.	IV set	10	
25.	Spirit (70% alcohol)	1 bottle	
26.	Steel drum with cotton	1	
27.	Urinary catheter(plain and folys)	5 each	
28.	Sutures for tear or episiotomy repair (2.0 chromic catgut)	12 PC	
29.	Bleach (chlorine-base compound)	2 packets	
30.	Clean (plastic) sheet to place under mother	4	
31.	Sanitary pads	1 box	
32.	Peri-pads Sterile	As per need	
33.	Clean towels for drying and wrapping the baby	5	
34.	Cord ties (sterile)	50	
35.	Blanket for the baby	5	
36.	Baby feeding cup	3	
37.	Impregnated bed net	2	
38.	Utility Gloves	10 pairs	
39.	Sterile Gloves	50 pairs	
40.	Long plastic apron	2	
41.	Goggles	2	
42.	Container for sharps disposal	1	
43.	Needle cutter	1	
44.	Receptacle for soiled linens	1	
45.	Bucket for soiled pads and swabs	2	
46.	Bucket for placenta (5 ltr.)	2	
47.	Well labelled color coded bins as per HCWM guideline 2014 (MoHP)	1 set	
48.	Wall Clock	1	
49.	Torch with extra batteries and bulb	1-2	

50.	Maternity register	1-2	
51.	Birth certificate	as per need	
52.	Partograph	As per need	
		**Total Score**	
		**Total percentage= Total Score/52 x 100**	

Each row gets a score of 1 if the mentioned medicines are available otherwise 0.

**Scoring Chart**	
**Total percentage**	**Score**
0-50	0
50-70	1
70-85	2
85-100	3
Score for standard 2.7.1.9.3	

Annex 2.7.1c Medicines and Supplies for ER Trolley Labor Room

SN	Name	Required No	Score
1.	Atropine Injection	10 amp	
2.	Adrenaline Injection	3vial	
3.	Xylocaine 1% and 2% Injections with Adrenaline	2vial	
4.	Xylocaine 1% and 2 % Injections without Adrenaline	2vial	
5.	Xylocaine Gel	2 tube	
6.	Diclofenac Injection	5 amp	
7.	Hyoscine Butylbromide Injection	5amp	
8.	Diazepam injection	2 amp	
9.	Morphine Injection / Pethidine Injection	2 amp	
10.	Hydrocortisone Injection	4vial	
11.	Chlorpheniramine meliate Injection	4amp	
12.	Dexamethasone Injection	4vial	
13.	Ranitidine/Omeperazole Injection	4 amp	
14.	Frusemide Injection	5 amp	
15.	Dopamine injection	2 amp	
16.	Noradrenaline injection	2 amp	
17.	Digoxin injection	2 amp	
18.	Verapamil injection	2 amp	

19.	Amidarone injection	2 amp	
20.	Glyceryl trinitrate/nitroglycerine injection	10 tab/ 5amp	
21.	Labetolol injection	5 amp	
22.	Magnesium sulphate injection	30 amp	
23.	Calcium gluconate injection	2 amp	
24.	Sodium bicarbonate injection	2 amp	
25.	Ceftriaxone Injection	4 vials	
26.	Metronidazole Injection	4 bottles	
27.	Dextrose 25%/ 50% Injection	2	
28.	IV Infusion set (Adult/Pediatric)	2	
29.	IV Canula (16, 18, 20, 22, 24, 26 Gz)	2 each	
30.	Disposable syringes 1 ml, 3 ml, 5 ml, 10 ml, 20 ml, 50 ml	5 each	
31.	Disposable Gloves (Size 6, 6.5, 7, 7.5)	3 each	
32.	Water for injection 10 ml	10 amp	
33.	Sodium chloride-15%w/v and Glycerin-15% w/v (for enema)	5	
34.	PPH management Set<br>• (IV canula: 16/18G, IV fluids as per treatment protocol, IV set, Foley's catheter, Urobag)<br>• Condom tamponade set- Sponge holder:2, Sim's speculum:1, Foley's catheter:1, Condom:2, IV fluids: NS1, IV set, Thread, Cord Clamp),<br>• Inj Oxytocin, Tab Misoprostol,	At least 1	
35.	Eclampsia management Set<br>(Knee hammer, IV canula: 16/18G, IV fluids, IV set, Foley's catheter, Urobag, ambu bag, Oxygen, Inj MgSO4: 46 ampoules, Inj lignocaine 2%, Inj Calcium gluconate, Distilled water, Disposable syringe 20ml-1, 10ml-8, Cap Nifedipin- 5mg 4 Cap)	At least 1	
		**Total Score**	
		**Total Percentage =Total Score/35X100**	

Each row gets a score of 1 if all the required number is available otherwise 0.

**Scoring chart**	
**Total percentage**	**Score**
0-50	0
50-70	1
70-85	2
85-100	3
**Score for Standard 2.7.1.9.4**	

Area	Code	Verification		
**Maternity Services**	**2.7**			
**Maternity Inpatient Service**	**2.7.2**			
**Components**		**Standards**	**Obtained Score**	**Maximum Score**
2.7.2.1 Space for work	2.7.2.1.1	Separate space for nursing station is available		1
	2.7.2.1.2	Separate changing room available for male and female staffs		1
	2.7.2.1.3	Separate store room is available		1
	2.7.2.1.4	Separate space dedicated for pre-labor, labor and post-labor patients		1
2.7.2.2 Furniture and supplies available and functioning	2.7.2.2	Furniture and supplies to carry out the inpatient services are available and functioning (See annex 2.7.2a Furniture and supplies for maternity inpatient wards At the end of this standard) (including nursing station)		3
2.7.2.3 Medicine and supplies available	2.7.2.3	Medicine and supplies to carry out the inpatient services are available General Ward (See Annex 2.7.2bmedicine and supplies for maternity inpatient wards At the end of this standard)		3
2.7.2.4 Nursing and support staff for inpatient service	2.7.2.4.1	Adequate numbers of nursing staff are available in ward per shift (nurse patient ratio 1:6 in general ward)		1
	2.7.2.4.2	At least one trained office assistant per shift in each ward		1
2.7.2.5 Duty rosters	2.7.2.5	Duty roster of doctors, nurses, paramedics and support staffs kept visibly in nursing station		1
2.7.2.6 Communication	2.7.2.6	Telephone facility is available with list of important contact numbers and hospital codes visibly kept		1
2.7.2.7 Emergency management of inpatients	2.7.2.7.1	All staffs in wards are trained for BLS and oriented about emergency code 001 or blue code		1
	2.7.2.7.2	At least one emergency trolley with emergency medicine available in ward (Annex 2.7.2c Medicine and Supplies for ER Trolley for Maternity In patient Ward At the end of this standard)		3
	2.7.2.7.3	At least one defibrillator in immediate accessible area		1
2.7.2.8 Physical facilities for patient	2.7.2.8.1	Separate area designated for admission of male and female inpatients in general ward		1
	2.7.2.8.2	There are adequate toilets for male and female patients in each ward (1 for 6 female bed)		1
	2.7.2.8.3	Safe drinking water is available 24 hours for inpatients		1
	2.7.2.8.4	Hours/ Time allocated for visitors to meet the inpatients and controlled traffic to prevent cross infection		1
	2.7.2.8.5	Separate space is available for patients' visitors (Kuruwa Ghar).		1

2.7.2.9 Communication	2.7.2.9	Basic information regarding admitted patients is displayed in a separate board		1
2.7.2.10 IEC/ BCC Materials	2.7.2.10	Appropriate IEC materials (posters, leaflets etc.) are available in the inpatient ward with focus on infection prevention		1
2.7.2.11 Recording and reporting	2.7.2.11	Admission and discharge registers are available and are being filled completely (HMIS 8.1 and 8.2)		1
2.7.2.12 Infection prevention	2.7.2.12.1	Personal Protective equipment are available and used whenever required		1
	2.7.2.12.2	Each ward has hand sanitizer in visible place for health workers to use before and after touching patients		1
	2.7.2.12.3	There are well labelled color-coded bins for waste segregation and disposal as per HCWM guideline 2014 (MoHP)		1
	2.7.2.12.4	Hand washing facility with running water and liquid soap is available and being practiced		1
	2.7.2.12.5	Needle cutter is used		1
	2.7.2.12.6	Chlorine solution is available and utilized for decontamination		1
**Standard 2.7.2**		**Total Obtained Score**		33
		**Total Percentage (Total Obtained Score/ 33 x100)**		

Annex 2.7.2a Furniture and Supplies for Maternity Inpatient wards

SN	General Items	Required No.	Score
1.	Working table	1-2	
2.	Chairs	2	
3.	Cup board	2	
4.	Shelves	1	
5.	Bed side table	per bed-1	
6.	Stools (for visitor)	per bed 1	
7.	Patient Beds (Metal bed / adjustable head/ mechanical ratchet, 3 X 6 ft.)	As per sanctioned bed	
8.	IV stand	As per bed	
9.	Medicine trolley	1	
10.	Dressing trolley	1	
11.	Wall Clock	2	
12.	Oxygen Concentrator	1 per 5 bed	
13.	Suction machine (foot/electric)	1	
14.	Laryngoscope with blade and batteries	1	

15.	ET tubes of different sizes	At least 2 each	
16.	Self-inflating bag air mask – adult, child, neonate size	1 set	
17.	BP set and stethoscope (Non-Mercury)	2 sets	
18.	Thermometer	3-5	
19.	Baby and adult weighing scale	1 each	
20.	Steel drum with sterile cotton	1	
21.	Steel drum with sterile gauze and pad	1	
22.	Scissors	2	
23.	Cheatle Forceps with Jar	2	
24.	Catheter set	2	
25.	Dressing set	2	
26.	Mattress with bedcover, pillow with pillow cover, blanket with cover	2-3 set per bed	
27.	Torch with extra batteries and bulb	2-3	
28.	Inpatient register as per ICD code	As per need	
29.	Inventory Records	As per need	
30.	Cardex files	As per bed	
31.	Waste bins color coded based on HCWM guideline 2014 (MoHP)	1 set per room	
		**Total Score**	
		**Total percentage= Total Score/31x 100**	

Each row gets a score of 1 if the mentioned medicines are available otherwise 0.

**Scoring Chart**	
**Total Percentage**	**Score**
0-50	0
50-70	1
70-85	2
85-100	3
**Score for Standard 2.7.2.2**	

Annex 2.7.2b Medicine and Supplies for Maternity Inpatient Ward

S.No.	Medicine and supplies	Required No.	Score
1.	Normal Saline Injection	15	
2.	Dextrose 5% Injection	15	
3.	Ringers' Lactate Injection	15	
4.	Dextrose 5% Normal Saline Injection	15	
5.	Distilled Water	10	
6.	IV Infusion Set	10	
7.	IV set	5	
8.	IV Catheter 18G,20G,22G,24G,26G	5 each	
9.	Gloves (Utility)	1 box	
10.	Mask, Cap, Gowns	As per need	
11.	Disposable syringes 1 ml, 3 ml, 5 ml, 10 ml, 30 ml, 50 ml	As per need	
		**Total Score**	
		**Total Percentage = Total Score/ 11 x 100**	

Each row gets a score of 1 if the mentioned medicines are available otherwise 0.

**Scoring Chart**	
**Total Percentage**	**Score**
0-50	0
50-70	1
70-85	2
85-100	3
**Score for Standard 2.7.2.3**	

Annex 2.7.2c Medicines and Supplies for ER Trolley Maternity Inpatient Ward

SN	Name	Required No	Score
1.	Atropine Injection	10	
2.	Adrenaline Injection	3	
3.	Xylocaine 1% and 2% Injections with Adrenaline	2	
4.	Xylocaine 1% and 2% Injections without Adrenaline	2	
5.	Xylocaine Gel	2	
6.	Diclofenac Injection	5	
7.	Hyoscine Butylbromide Injection	5	

8.	MetharginInjecyion	5	
9.	Diazepam injection	2	
10.	Morphine Injection / Injection Pethidine	2	
11.	Hydrocortisone Injection	4	
12.	Antihistamine Injection	4	
13.	Dexamethasone Injection	4	
14.	Ranitidine/Omeperazole Injection	4	
15.	Frusemide Injection	5	
16.	Dopamine injection	2	
17.	Noradrenaline injection	2	
18.	Digoxin injection	2	
19.	Verapamil injection	2	
20.	Amidarone injection	2	
21.	Glyceryl trinitrate/ notroglycerine	1o tab/ 5 amp	
22.	Labetolol injection	1	
23.	Magnesium sulphate injection	30	
24.	Calcium gluconate injection	2	
25.	Sodium bicarbonate injection	2	
26.	Ceftriaxone Injection	4	
27.	Metronidazole Injection	4	
28.	Dextrose 25% / 50% Injection	2	
29.	IV Infusion set (Adult/Pediatric)	5 each	
30.	IV Canula (16, 18, 20, 22, 24, 26 Gz)	2	
31.	Disposable syringes 1 ml, 3 ml, 5 ml, 10 ml, 20 ml, 50 ml	2 each	
32.	Disposable Gloves 6, 6.5, 7, 7.5	5 each	
33.	Distilled Water	3	
34.	Sodium chloride-15%w/v and Glycerin-15% w/v (for enema)	5	
		**Total Score**	
		**Total Percentage = Total Score/34 X100**	

Each row gets a score of 1 if all the required number is available otherwise 0.

**Scoring chart**	
**Total percentage**	**Score**
0-50	0
50-70	1
70-85	2
85-100	3
**Score for Standard 2.7.2.8.2**	

Area	Code	Verification		
**Maternity Service**	**2.7**			
**Birthing center service**	**2.7.3**			
**Components**		**Standards**	**Obtained Score**	**Maximum Score**
2.7.3.1 Birthing center available	2.7.3.1	Dedicated block for birthing center with health facility having more than 500 deliveries per month		1
2.7.3.2 Space for work	2.7.3.2.1	Separate space for nursing station is available		1
	2.7.3.2.2	Separate changing room available for male and female staffs		1
	2.7.3.2.3	Separate store room is available		1
	2.7.3.2.4	Separate space dedicated for pre-labor, labor and post-labor patients		1
2.7.3.3 Furniture and supplies available and functioning	2.7.3.3	Furniture and supplies to carry out the inpatient services are available and functioning (See Annex 2.7.3aFurniture and supplies for birthing center At the end of this standard)		3
2.7.3.4 Medicine and supplies available	2.7.3.4	Medicine and supplies to carry out the inpatient services are available General Ward (See Annex 2.7.3b Medicine and supplies for Birthing Center At the end of this standard)		3
2.7.3.5 Nursing station	2.7.3.5	There is a set of at least one table and two chairs in the nursing station and shelves for storage of charts and inpatient forms and formats		1
2.7.3.6 Nursing staff for inpatient service	2.7.3.6	Hospital delivery service has adequate and trained staffing		
	2.7.3.6.1	Nurse/Midwife: pregnant women ratio 1:2 in pre-labor; 2:1 per delivery table and 1:6 in post-natal ward		1
	2.7.3.6.2	At least one ASBA trained medical officer on duty		1
	2.7.3.6.3	At least one office assistant is available per shift		1
	2.7.3.6.4	All staffs- nursing, medical practitioner designated for delivery services are trained skilled birth attendants/Midwife		1
2.7.3.7 Duty rosters	2.7.3.7	Duty roster to cover 24 hours shift is developed and placed in visible place		1
2.7.3.8 Communication	2.7.3.8	Telephone facility is available with list of important contact numbers and hospital codes visibly kept		1

2.7.3.9 Emergency management of inpatients	2.7.3.9.1	All staffs in wards are trained for BLS and oriented about emergency code 001 or blue code		1
	2.7.3.9.2	At least one emergency trolley with emergency medicine available in ward (See Annex 2.7.3c Medicine and Supplies for ER Trolley for Maternity Inpatient Ward At the end of this standard)		3
	2.7.3.9.3	At least one defibrillator in immediate accessible area		1
2.7.3.10 Physical facilities for patient	2.7.3.10.1	Separate area designated for admission of male and female inpatients in general ward		1
	2.7.3.10.2	There are adequate toilets for male and female patients in each ward (1 for 6 female bed)		1
	2.7.3.10.3	Safe drinking water is available 24 hours for inpatients		1
	2.7.3.10.4	Hours/ Time allocated for visitors to meet the inpatients and controlled traffic to prevent cross infection		1
	2.7.3.10.5	Separate space is available for patients' visitors (Kuruwa Ghar).		1
2.7.3.11 Communication	2.7.3.11	Basic information regarding admitted patients is displayed in a separate board		1
2.7.3.12 IEC/BCC Materials	2.7.3.12	Appropriate IEC materials (posters, leaflets etc.) are available in the inpatient ward with focus on infection prevention		1
2.7.3.13 Recording and reporting	2.7.3.13	Admission and discharge registers are available and are being filled completely (HMIS 8.1 and 8.2)		1
2.7.3.14 Infection prevention	2.7.3.14.1	PPE are available and used whenever required		1
	2.7.3.14.2	Each ward has hand sanitizer in visible place for health workers to use before and after touching patients		1
	2.7.3.14.3	There are well labelled color-coded bins for waste segregation and disposal as per HCWM guideline 2014 (MoHP)		1
	2.7.3.14.4	Hand-washing facility with running water and liquid soap is available and being practiced		1
	2.7.3.14.5	Needle cutter is used		1
	2.7.3.14.6	Chlorine solution is available and utilized for decontamination		1
**Standard 2.7.3**		**Total Score**		37
		**Total Percentage (Total Score/ 37 x100)**		

Annex 2.7.3a Furniture and Supplies for Birthing Center

SN	General Items	Required No.	Score
1.	Working table	1-2	
2.	Chairs	2	
3.	Cup board	2	
4.	Shelves	1	
5.	Bed side table	per bed-1	
6.	Stools (for visitor)	per bed 1	
7.	Patient Beds (Metal bed / adjustable head/ mechanical ratchet, 3 X 6 ft.)	As per sanctioned bed	
8.	IV stand	As per bed	
9.	Medicine trolley	1	
10.	Dressing trolley	1	
11.	Wall Clock	2	
12.	OxygenConcentrator	1 per 5 bed	
13.	Suction machine (foot/electric)	1	
14.	Laryngoscope with blade and batteries	1	
15.	ET tubes of different sizes	At least 2 each	
16.	Self-inflating bag air mask – adult, child, neonate size	1 set	
17.	BP set and stethoscope (Non-Mercury)	2 sets	
18.	Thermometer	3-5	
19.	Baby and adult weighing scale	1 each	
20.	Steel drum with sterile cotton	1	
21.	Steel drum with sterile gauze and pad	1	
22.	Scissors	2	
23.	Cheatle Forceps with Jar	2	
24.	Catheter set	2	
25.	Dressing set	2	
26.	Delivery set	At least 5	
27.	Delivery forceps set	At least 2	

28.	Delivery vacuum set	At least 2	
29.	Mattress with bedcover, birthing pillow with pillow cover, blanket with cover	1 set per bed	
30.	Birthing balls	At least 3	
31.	Torch with extra batteries and bulb	2-3	
32.	Inpatient register/entered per ICD code	As per need (1)	
33.	Inventory Records/ entered per ICD code	As per need (1)	
34.	Cardex files	As per bed	
35.	Waste bins color coded based on HCWM guideline 2014 (MoHP)	1 set per room	
		**Total Score**	
		**Total percentage= Total Score/35 x 100**	

Each row gets a score of 1 if the mentioned medicines are available otherwise 0.

**Scoring Chart**	
**Total Percentage**	**Score**
0-50	0
50-70	1
70-85	2
85-100	3
**Score for Standard 2.7.3.3**	

Annex 2.7.3b Medicine and Supplies for Birthing Center

S.No.	Medicine and supplies	Required No.	Score
1.	Normal Saline Injection	15	
2.	Dextrose 5% Injection	15	
3.	Ringers' Lactate Injection	15	
4.	Dextrose 5% Normal Saline Injection	15	
5.	Distilled Water	10	
6.	IV Infusion Set	10	
7.	IV set	5	
8.	IV Canula (16G,18G,20G,22G,24G,26G)	5 each	

9.	Gloves (Utility)	1 box	
10.	Mask, Cap, Gowns	As per need	
11.	Disposable syringes 1 ml, 3 ml, 5 ml, 10 ml, 30 ml, 50 ml	As per need	
		**Total Score**	
		**Total Percentage = Total Score/ 11 x 100**	

Each row gets a score of 1 if the mentioned medicines are available otherwise 0.

**Scoring Chart**	
**Total Percentage**	**Score**
0-50	0
50-70	1
70-85	2
85-100	3
**Score for Standard 2.7.3.4**	

Annex 2.7.3c Medicines and Supplies for ER Trolley Labor Room

SN	Name	Required No	Score
1.	Atropine Injection	10 amp	
2.	Adrenaline Injection	3vial	
3.	Xylocaine 1% and 2% Injections with Adrenaline	2vial	
4.	Xylocaine 1% and 2% Injections without Adrenaline	2vial	
5.	Xylocaine Gel	2 tube	
6.	Diclofenac Injection	5 amp	
7.	Hyoscine Butylbromide Injection	5amp	
8.	Diazepam injection	2 amp	
9.	Morphine Injection / Pethidine Injection	2 amp	
10.	Hydrocortisone Injection	4vial	
11.	Antihistamine Injection	4amp	
12.	Dexamethasone Injection	4vial	

13.	Ranitidine/Omeperazole Injection	4 amp	
14.	Frusemide Injection	5 amp	
15.	Dopamine injection	2 amp	
16.	Noradrenaline injection	2 amp	
17.	Digoxin injection	2 amp	
18.	Verapamil injection	2 amp	
19.	Amidarone injection	2 amp	
20.	Glyceryl trinitrate/ nitroglycerine injection	10 tab/ 5amp	
21.	Labetolol injection	5 amp	
22.	Magnesium sulphate injection	30 amp	
23.	Calcium gluconate injection	2 amp	
24.	Sodium bicarbonate injection	2 amp	
25.	Ceftriaxone Injection	4 vials	
26.	Metronidazole Injection	4 bottles	
27.	Dextrose 25% / 50% Injection	2	
28.	IV Infusion set (Adult/Pediatric)	2	
29.	IV Canula (16, 18, 20, 22, 24, 26 Gz)	2 each	
30.	Disposable syringes 1 ml, 3 ml, 5 ml, 10 ml, 20 ml, 50 ml	5 each	
31.	Disposable Gloves 6, 6.5, 7, 7.5	3 each	
32.	Distilled Water	10 amp	
33.	Sodium chloride-15%w/v and Glycerin-15% w/v (for enema)	5	
34.	PPH management Set<br>• (IV canula: 16/18G, IV fluids as per treatment protocol, IV set, Foley's catheter, Urobag)<br>• Condom tamponade set- Sponge holder:2, Sim's speculum:1, Foley's catheter:1, Condom:2, IV fluids: NS1, IV set, Thread, Cord Clamp),<br>• Inj Oxytocin, Tab Misoprostol,	At least 1	
35.	Eclampsia management Set<br>(Knee hammer, IV canula: 16/18G, IV fluids, IV set, Foley's catheter, Urobag, ambu bag, Oxygen, Inj MgSO4: 46 ampoules, Inj lignocaine 2%, Inj Calcium gluconate, Distilled water, Disposable syringe 20ml-1, 10ml-8, Cap Nifedipin- 5mg 4 Cap)	At least 1	
		**Total Score**	
		**Total Percentage =Total Score/35X100**	

Each row gets a score of 1 if all the required number is available otherwise 0.

Scoring chart	
**Total percentage**	**Score**
0-50	0
50-70	1
70-85	2
85-100	3
**Score for Standard 2.7.3.9.2**	

**Area**	**Code**	**Verification**		
**Surgery / Operation Services**	**2.8**			
**Components**		**Service Standards**	**Obtained Score**	**Maximum Score**
2.8.1 Time for surgical services/ operations	2.8.1.1.1	Routine minor and intermediate surgeries available on scheduled days		1
	2.8.1.1.2	Routine major surgeries available on scheduled days		1
	2.8.1.2	Emergency surgeries available round the clock		1
	2.8.1.3	At least six functional operating rooms/ theater		1
2.8.2 Staffing	2.8.2.1	For one surgery, at least a team is composed of: MS with one trained medical officer, two OT trained nurse (one scrub and one circulating), one Anesthesiologist, one anesthesia assistant and one office assistant (for cleaning and helping)		1
	2.8.2.2	For overall management of operation theatre, there is one OT nurse (with minimum bachelors degree) assigned as OT in-charge		1
	2.8.2.3	At least two nurses in pre-anesthesia area for receiving and transferring of the patient		1
	2.8.2.4	At least two ICU trained nurses for post anesthesia care for receiving patient after OT		1

2.8.3 Surgical services available	2.8.3.1	General Surgeries (See Annex 2.8a List of Minimum Surgeries Available At the end of this standard)		3
	2.8.3.2	Obstetrics and gynecology surgeries (See Annex 2.8b List of Minimum Obstetrics and gynecology surgeries Available At the end of this standard)		3
	2.8.3.3	Orthopedic Surgeries (See Annex 2.8c List of Minimum Orthopedics Surgeries Available At the end of this standard)		3
	2.8.3.4	ENT surgeries available (Annex 2.8d Types of ENT Surgeries Available At the end of this standard)		3
	2.8.3.5	Neurosurgeries available (Annex 2.8e Types of Neurology Surgeries Available At the end of this standard)		3
	2.8.3.6	Urology Surgeries available (Annex 2.8f Types of Urology Surgery Available At the end of this standard)		3
	2.8.3.7	Burn/plastic Surgeries available (Annex Types of Burn/Plastic 2.8g Surgery Available At the end of this standard)		3
2.8.4 Patient counseling and	2.8.4.1	Indications and reviews the clinical history and physical examination is documented		1
	2.8.4.2	Pre-anesthesia checkup done for routine surgeries and documented		1
	2.8.4.3	Informed consent is taken before surgery; patients and caretakers are given appropriate counseling about the surgery		1
2.8.5 WHO safe surgery checklist	2.8.5	The WHO Safe Surgery Checklist is available in OT and used		1
2.8.6 Patient preparation	2.8.6	Preoperative instructions for patient preparation are given and practiced with routine pre-anesthesia check up		1
2.8.7 Operation Theatre/Room	2.8.7.1	OT has appropriate physical set up (See Annex 2.8h Physical Set Up for OT At the end of this standard).		3
	2.8.7.2	Each operating room has general equipment, instruments and supplies available (See Annex 2.8i Furniture, Equipment, Instruments and Supplies for OT At the end of this standard)		3
	2.8.7.3	Each operating room has medicines and supplies available (See Annex 2.8j General Medicine and Supplies for OT At the end of this standard)		3
	2.8.7.4	Surgical sets for minimum list of the surgical services available (See Annex 2.8k Surgical sets for Minimum list of the surgical procedures At the end of this standard)		3

2.8.8.1 Availability of anesthesia Services	2.8.8.1	Anesthesia service is provided following the standards operating procedure		
	2.8.8.1.1	Local anesthesia		1
	2.8.8.1.2	Regional anesthesia		1
	2.8.8.1.3	Spinal anesthesia		1
	2.8.8.1.4	General anesthesia		1
2.8.8.2Equipment, instruments and supplies for anesthesia	2.8.8.2	Equipment, instrument and supplies for anesthesia available (See Annex 2.8l Equipment, Instrument and Supplies for Anesthesia At the end of this standard)		3
2.8.8.3 Medicine and supplies for anesthesia	2.8.8.3	Medicine and supplies for anesthesia available (See Annex 2.8m Medicine and Supplies for Anesthesia At the end of this standard)		3
2.8.8.4 Staffing and supervision	2.8.8.4.1	Anesthesia should be provided, led, or overseen by an anesthesiologist		1
2.8.9 Pre anesthesia and post-operative care	2.8.9.1	Dedicated space for pre-anesthesia assessment and post-anesthesia recovery with patient bed, IV stand, IV cannula, fixing tapes, infusion sets, burette sets, syringes, three-way stop cocks and at least one cardiac monitor		1
	2.8.9.2	Separate area designated for post-operative care to stabilize the patient after surgery		1
	2.8.9.3	Staffs are specified for the post-operative care including close monitoring of the vital signs and observation of patient		1
	2.8.9.4	Patients' pain management is prioritized, measures well documented and analgesic effect followed up		1
	2.8.9.5	Patient undergoing surgical procedure is done pre- anesthetic check-up, continuously monitored during and at least 2 hours post- anesthesia		1
	2.8.9.6	Adequate information shared for patient care and patient followed by at least one nurse/doctor for hand over or transfer of patient within or outside the hospital		1
2.8.10 Recording	2.8.10.1	Recording is done for all surgeries procedure including observation, management and complications if any		1
	2.8.10.2	Records of all anesthetic procedures are kept and reported		1
2.8.11 Infection prevention protocol is strictly followed by all staffs in operation theatre/room				
2.8.11.1 Hand hygiene	2.8.11.1	Hand-washing and scrubbing facility with running water and soap is available and being practiced with elbow tap		1

2.8.11.2 Appropriate PPE	2.8.11.2	Gowns and sterile drapes for patients and sterile gown for surgery team are available and used whenever required.		1
2.8.11.3 Fumigation	2.8.11.3	Fumigation is done at least once a week in the OT on Saturdays and as per need.		1
2.8.11.4 Disinfection of instruments	2.8.11.4	High Level Disinfection (e.g. Cidex) facility is available and being practiced.		1
2.8.11.5 High Wash	2.8.11.5	High wash is done once a month in OT		1
2.8.11.5 Appropriate segregation of waste	2.8.11.6	Separate colored waste bins based on HCWM guideline 2014 (MoHP) are available and used		1
2.8.11.7 Disposal of sharps	2.8.11.7	Needle cutter is used		1
2.8.11.8 Cleaning	2.8.11.8	Chlorine solution is available and utilized for decontamination.		1
**Standard 2.8**		**Total Score**		73
		**Total Percentage= Total Score/ 69 x 100**		

Annex 2.8a General Surgeries Available

**S.No.**	**List of the surgeries available (minimum)**	**Score**
Minor		
1.	I & D under LA	
2.	Excision of cysts, ganglion, lump, lymhnode, lipoma, skin papilloma, corn under LA	
3.	Excision of ingrowing toe nail under digital block	
4.	Wound debridement	
5.	Skin suturing < 5cm size	
6.	Foreign Body removal under LA	
7.	Repair split ear	
8.	True cut biopsy	
9.	Circumcision Under LA	
10.	Haemorrhoid banding	
Intermediate		
11.	Chest tube insertion under LA	
12.	Eversion of sac for hydrocele (EVS)	
13.	Herniotomy under IVA	

14.	Mesh Repair / Darn Repair (under LA/SA)	
15.	Amputation	
16.	Split Skin Graft(SSG) (< 1% TBSA)	
17.	Large wound dressing / debridement under IVA/SA	
18.	Chest tube insertion under IVA	
19.	Circumcision under IVA	
20.	I & D under IVA eg. Breast abscess, perineal abscess	
21.	Release of tongue tie	
22.	Fistulotomy	
23.	Haemorrhoidectomy	
24.	Vasectomy	
Major		
25.	Exploratory laparotomy	
26.	Appendectomy	
27.	Exploration for obstructed hernia	
28.	Mesh repair incisional hernia	
29.	Open cholecystectomy	
30.	Ileostomy / colostomy formation	
31.	Open pyelolithotomy	
32.	Open ureterolithotomy	
33.	Open suprapubic cystolithotomy	
34.	Oophorectomy	
35.	Extensive wound debridement (Necrotising Fascitis)	
**Total score**		
**Total Percentage= Total score/38 x 100**		

Each row gets a score of 1 if all the required number is available otherwise 0.

**Scoring chart**	
**Total percentage**	**Score**
0-50	0
50-70	1
70-85	2
85-100	3
**Score for Standard 2.8.3.1**	

Annex 2.8b Obstetrics and Gynecology Surgeries Available

S.No.	List of the surgeries available (minimum)	Score
Intermediate		
1.	I & D under IVA eg. Breast abscess	
2.	Removal of product of conception and surgical Abortion	
3.	Cervical Tear Repair	
Major		
4.	Caesarean Section	
5.	Minilap	
6.	Vaginal hysterectomy	
7.	Abdominal hysterectomy	
8.	Oophorectomy	
9.	Tumor removal (Obstetrics and gynaecological)	
10.	Exploratory laparotomy	
	**Total score**	
	**Total Percentage= Total score/ 10x 100**	

Each row gets a score of 1 if all the required number is available otherwise 0.

**Scoring chart**	
**Total percentage**	**Score**
0-50	0
50-70	1
70-85	2
85-100	3
**Score for Standard 2.8.3.2**	

Annex 2.8c Types of Orthopedics Surgeries Available

S.N.	Minimum list of Orthopedic Surgeries	Score
1.	POP + Immobilization without anesthesia	
2.	POP + cast under anesthesia	
3.	Hip Spica cast	
4.	Joint aspiration	
5.	Intralesional steriod injection	
6.	Skin traction	

7.	Gallows traction	
8.	Skeletal Traction	
9.	Reduction of shoulder, elbow, small joints dislocation	
10.	Reduction of hip and knee dislocation	
11.	Extensor Tendon Repair	
12.	Flexor Tendon Repair	
13.	Amputation under LA and/or sedation	
14.	Simple implant removal	
15.	Soft tissue benign tumor excision	
16.	Trigger finger Release	
17.	DeQuervain's Release	
18.	Carpal Tunnel Release	
19.	Dupuytren's Contracture Surgery	
20.	CRPP (small joints)	
21.	CRPP (supracondylar fractures)	
22.	CRPP (distal radial fractures)	
23.	CRPP (proximal humerus)	
24.	Amputation (life-saving) under GA	
25.	Rush Nailing	
26.	Arthrotomy small joints	
27.	Arthrotomy large joints	
28.	ORIF Lateral condyle fracture	
29.	ORIF Supracondylar fracture	
30.	ORIF Short Long Bones	
31.	ORIF olecranon process/patella	
32.	ORIF Long Long Bones	
33.	ORIF ankle/knee/wrist/elbow/shoulder/hip	
34.	External Fixation	
35.	Radial head excision	
36.	IMIL Nailing Tibia/Femur	
37.	PFN	
38.	MIPO various bones	
39.	DHS/DCS/CCS hip	

40.	HRA	
41.	Arthroscopy	
42.	Discectomy	
43.	Fasciotomy	
44.	Decompression surgery for Acute osteomyelitis	
	**Total score**	
	**Total Percentage= Total score/44 x 100**	

Each row gets a score of 1 if all the required number is available otherwise 0.

**Scoring chart**	
**Total percentage**	**Score**
0-50	0
50-70	1
70-85	2
85-100	3
**Score for Standard 2.8.3.3**	

Annex 2.8d Types of ENT Surgeries Available

**Minimum list of ENT Surgeries Available**		**Score**
Emergency ENT surgeries		
1.	Ludwigs' Angina	
2.	Foreign Body removal (including bronchsocopy)	
Routine ENT surgeries		
3.	Myringoplasty /Tympanoplsty	
4.	Mastoid drill / Motor with burrs	
5.	Stapes surgery	
6.	Rigid oesophagoscopy	
7.	Tonsil adenoid	
8.	Endosopic sinus surgery and FESS	
9.	Septoplasty/ Rhinoplasty	
10.	Laryngeal surgery	
	**Total score**	
	**Total Percentage= Total score/10 x 100**	

Each row gets a score of 1 if all the required number is available otherwise 0.

Scoring chart	
**Total percentage**	**Score**
0-50	0
50-70	1
70-85	2
85-100	3
**Score for Standard 2.8.3.4**	

Annex 2.8e Types of Neurology Surgeries Available

**Minimum list of Neurology Surgeries Available**		**Score**
1.	Carniotomy	
2.	Head injury	
3.	Stroke	
4.	Chronic subdural	
5.	Hydrocephalus	
6.	Superficial tumors	
7.	Extra ventricular drain	
	**Total score**	
	**Total Percentage= Total score/7 x 100**	

Each row gets a score of 1 if all the required number is available otherwise 0.

Scoring chart	
**Total percentage**	**Score**
0-50	0
50-70	1
70-85	2
85-100	3
**Score for Standard 2.8.3.5**	

Annex 2.8f Types of Urology Surgeries Available

Minimum list of Urology Surgeries Available		Score
1.	Supra-pubic Catheterization	
2.	Transurethral resection of prostate	
3.	Testicular surgery	
4.	Penile Surgery (including repair of penile fracture)	
5.	Fournier’s Gangrene Debridement and Drainage	
6.	Open pyelolithotomy	
7.	Open ureterolithotomy	
8.	Open suprapubic cystolithotomy	
9.	Renal Surgery (nephrectomy, pyelopasty)	
	**Total score**	
	**Total Percentage= Total Score/9 x100**	

Each row gets a score of 1 if all the required number is available otherwise 0.

**Scoring chart**	
**Total percentage**	**Score**
0-50	0
50-70	1
70-85	2
85-100	3
**Score for Standard 2.8.3.6**	

Annex 2.9g Types of Burn/Plastic Surgeries Available

Minimum list of Burn/Plastic Surgeries Available		Score
1.	Skin Graft	
2.	Flap (Cutaneous, Musculo -cutaneous, Fasciocutaneous)	
3.	Contracture release	
4.	Hand reconstruction surgery	
5.	Ear reconstruction	
6.	Rhinoplasty	

7.	Eyelid reconstruction	
8.	Burn surgery	
	**Total score**	
	**Total Percentage= Total score/7x 100**	

Each row gets a score of 1 if all the required number is available otherwise 0.

**Scoring chart**	
**Total percentage**	**Score**
0-50	0
50-70	1
70-85	2
85-100	3
**Score for Standard 2.8.3.7**	

Annex 2.8h Physical Set Up for OT

SN	Physical Set Up	Score
	Separate room designated for OT with recovery room	
	Space designated for changing room for male and female staffs separately	
	Lockers for storage of the belongings of staffs	
	Separate shelves for storage of clean and dirty shoes at the entrance of the OT area demarked with red line	
	Space designated with sink facilitated with elbow tap for scrubbing	
	Designated space for tea room	
	Separate bathroom with at least one universal toilet for OT	
	Scrub basins with running water	
	Utility basins (at least 4)	
	**Total Score**	
	**Total percentage= Total Score/ 9 x 100**	

Each row gets a score of 1 if the mentioned test is available otherwise 0.

Scoring chart	
**Total percentage**	**Score**
0-50	0
50-70	1
70-85	2
85-100	3
**Score for Standard 2.8.7.1**	

Annex 2.8i Furniture, Equipment, Instruments and Supplies for each OT Room

SN	General Equipment and Instruments for OT	Standard Quantity for each OT Room	Score
1.	Wheel chair foldable, adult size	2	
2.	Stretcher	2	
3.	Patient trolley	2	
4.	Cupboards and cabinets for store	2	
5.	Working desk for anesthesia, nursing station, gowning	1 each	
6.	OT Table- universal type/ with wedge to position patient (*Radioluscent OT table with orthopedic attachment including C-arm for orthopedics)	1	
7.	Examining table	1	
8.	Mayo Stand with tray	At least 2	
9.	Operation theatre lights	At least 1	
10.	Ultra violet light source	At least 1	
11.	Medical Microscope for Surgeries (*for ENT, Neurosurgery)	At least 1 each*	
12.	Colposcope (*for Obstetrics and Gynecology Surgery)	At least 1 *	
13.	Central suction supply	Available	
14.	Central oxygen supply	Available	
15.	Electronic suction machine/ Foot-operated suction machine	At least 2	
16.	Oxygen concentrator/ Oxygen Cylinder	At least 2	

17.	Refrigerator / cold box	At least 1 each	
18.	Anesthesia machine with cardiac monitor	At least 1	
19.	Defibrillator	At least 1	
20.	Cautery/Diathermy machine	At least 1	
21.	Baby warmer	At least 1	
22.	Baby weight machine	At least 1	
23.	Anesthesia trolley	At least 2	
24.	Instrument trolley	At least 2	
25.	BP instrument with stethoscope	At least 1	
26.	Thermometer	At least 1	
27.	Steel Drum for gloves	At least 1	
28.	Steel Drum for Cotton/ Guaze	At least 1	
29.	Tourniquet, latex rubber, 75 cm	At least 2	
30.	Kidney tray (600cc)	At least 2	
31.	Covered instrument trays	At least 4	
32.	Mackintosh sheet	At least 1	
33.	Lead gown set	At least 4 sets	
34.	Bowl stand	At least 2	
35.	Cheatle forceps in jar	At least 2	
36.	Drapes for abdominal site<br>(laparotomy sheet, table cover, hook towel, mayo cover, plastic sheet, tetra)	As per need	
37.	Drapes for perineal region<br>(Laparotomy sheet, table cover, hook towel, mayo cover, plastic sheet, tetra, leggings)	As per need	
38.	Packing towel double wrapper	As per need	
39.	Sterile gloves (6,6.5,7,7.5,8)	5/5/5/5/5 each	
40.	Towels/ eye hole	As per need	
41.	Masks and caps	As per need	
42.	Torch light and batteries	At least 1 set	
43.	Foot steps	At least 2	
44.	Wall clock	At least 1	
45.	Waste bucket for scrub nurse	At least 1	

46.	IV stand	At least 2	
47.	Leak proof sharp container	At least 1	
48.	Generator back up for OT	At least 1	
49.	Flash autoclave (for orthopedics instruments)	At least 1	
50.	Color coded waste bins (based on HCWM guideline 2014 (MoHP))	At least 1 set	
		**Total Score**	
		**Total percentage= Total/ 50 x 100**	

Each row gets a score of 1 if the mentioned test is available otherwise 0.

**Scoring Chart**	
**Total percentage**	**Score**
0-50	0
50-70	1
70-85	2
85-100	3
**Score for Standard 2.8.7.2**	

Annex 2.8j Medicine and Supplies for OT

SN	Emergency Drugs (including neonates) for OT	Standard Quantity for 1 patient (for each OT room)	Score
1.	Midazolam Injection	5 vials	
2.	Hydrocortisone Powder for Injection	100ml 2 vial	
3.	Frusemide Injection	2 ampules	
4.	Dopamine Injection	5 vials	
5.	Transemic Acetate Injection	2 ampules	
6.	Hydralizine Injection	5 vials	
7.	Calcium Gluconate Injection	10ml X 2 ampules	
8.	Magnesium sulphate Injection	0.5 gms X 28	
9.	Oxytocin Injection	10 Ampules	
10.	Dextrose (25%) / (50%) Injection	2 ampules	

11.	Naloxone Injection	1 ampule	
12.	Aminophyline Injection	2 ampules	
13.	Chloropheniramine Injection	2 ampules	
14.	Mephentine Injection	1 vial	
15.	IV Fluids- Ringers Lactate / Normal Saline/ Dextrose 5% Normal Saline/ Dextrose 5%	6 bottles each	
16.	IV infusion Set	4	
17.	IV Canula 22G/20G/18G	4 each	
		**Total Score**	
		**Total Percentage = Total Score/17 X 100**	

Each row gets a score of 1 if all the required number is available otherwise 0

**Scoring Chart**	
**Total percentage**	**Score**
0-50	0
50-70	1
70-85	2
85-100	3
**Score for Standard 2.8.7.3**	

Annex 2.8k Minimum List of Surgical Sets

S.No.	Items	Required number	Score
1.	Catheter set	At least 5	
2.	Suture set	At least 5	
3.	Dressing set of different size (small, medium, large)	At least 2 each	
4.	Incision and drainage set	At least 5	
5.	Appendectomy set	At least 2	
6.	Caesarian section set	At least 5	
7.	Hernia repair set	At least 2	
8.	Laparotomy set	At least 2	
9.	Vasectomy set	At least 2	

10.	Minilap set	At least 2	
11.	Open cholecystectomy set	At least 2	
12.	Ileostomy / colostomy formation	At least 1	
13.	Open pyelolithotomy	At least 1	
14.	Open ureterolithotomy	At least 1	
15.	Open suprapubic cystolithotomy	At least 1	
16.	Oophorectomy	At least 1	
17.	Orthopedics Basic Surgical Set	At least 2	
18.	K wire set	At least 2 set	
19.	IM Nailing set	At least 2 set	
20.	Hemi-Replacement Arthoplasty set	At least 1 set	
21.	External fixation set	At least 1 set	
22.	Amputation set (bone saw, bone file)	At least 1 set	
23.	Bronchoscope	At least 1set	
24.	Myringoplasty /Tympanoplasty Set	At least 1 set	
25.	Mastoid drill / Motor with burrs	At least 1 set	
26.	Stapes surgery set	At least 1 set	
27.	Rigid oesophagoscope	At least 1 set	
28.	Tonsil adenoid set	At least 1 set	
29.	Endosopic sinus surgery and FESS set	At least 1 set	
30.	Septoplasty/ Rhinoplasty set	At least 1 set	
31.	Laryngealsurgery set	At least 1 set	
32.	Craniotomy set	At least 2 Set	
33.	Supra-pubic Catheterization Set	At least 3 set	
34.	Set for Transurethral resection of prostate	At least 2 set	
35.	Set for Testicular torsion	At least 2 set	

36.	Penile surgery set	At least 2 set	
37.	Renal stone removal set	At least 2 set	
38.	Nephrectomy Set	At least 2 set	
39.	Pyelopasty Set	At .least2 set	
40.	Hand Set	At least 2	
41.	Reconstruction set (eyelid, ear, nose)	At least 1 each	
42.	Basic Plastic Surgery Set	At least 2	
43.	Microsurgical set for grafting	At least 1	
44.	Mic-micrograft	At least 1	
		**Total Score**	
		**Total Percentage= Total Score/44x100**	

Each row gets a score of 1 if all the required number is available otherwise 0

**Scoring Chart**	
**Total percentage**	**Score**
0-50	0
50-70	1
70-85	2
85-100	3
**Score for Standard 2.8.7.4**	

Annex 2.8I Equipment, Instruments and Supplies for Anesthesia

**S.No.**	**List of equipment, instruments and supplies for anesthesia**	**Required Number**	**Score**
1.	Supply of oxygen<br>(e.g., oxygen concentrator, cylinders or pipeline) with regulator and flow meter	At least 2 oxygen concentrator	
2.	Oropharyngeal airways<br>(Size 000, 00, 0, 1, 2, 3, 4)	At least 2 each	
3.	Anesthesia face masks<br>(Size 0, 1, 2, 3, 4)	At least 2 each	

4.	Laryngoscope, Mc Coy's curved blade and Miller's straight blade<br>(small, medium and large sizes for both adult and pediatric patients)	At least two	
5.	Endotracheal tubes, cuffed, uncuffed, different sizes (Sizes 2.5 - 8.0 ID)	At least two of each size	
6.	Intubation aids<br>(Magillsforcep of small and large size, bougie, stylets of small and large size)	As per need	
7.	Suction device and suction catheters of different sizes (Size 8 -16 Fr)	As per need	
8.	Adult and pediatric self inflating bags<br>(Size 2L, 1L, 0.5L)	As per need	
9.	Bain's breathing circuit	At least 2	
10.	Pediatric breathing circuit: Ayre's T-piece	At least 2	
11.	Equipment for intravenous infusions and injection of medications for adult and pediatric patients<br>(IV stand, IV canula, fixing tapes, infusion sets, blood transfusion sets, burette sets, syringes, three-way stop cocks)	As per need	
12.	Equipment for spinal anesthesia or regional blocks<br>(e.g., a set of spinal needle 25/26 G, small bowl, 5-10ml syringe, sponge holding forceps, kidney tray, large eye towel, cotton pieces, gauze pieces)	As per need	
13.	Examination (non-sterile) gloves	As per need	
14.	Sterile gloves	As per need	
15.	Pulse oximeter	At least 2	
16.	Access to a defibrillator	At least 1	
17.	Stethoscope	At least 2	
18.	Sphygmomanometer with appropriate sized cuffs for adult and pediatric patients	As per need	
19.	Non-invasive blood pressure monitor with appropriate sized cuffs for adult and pediatric patients	As per need	
20.	Anesthesia machine with inspired oxygen concentration monitor, anti-hypoxia device to prevent delivery of a hypoxic gas mixture, system to prevent misconnection of gas sources (e.g., tank yokes, hose connectors), automated ventilator with disconnect alarm.	At least 1	
21.	Electrocardiogram - three leads	As per need	
22.	Temperature monitor (intermittent)	As per need	
		**Total Score**	
		**Total percentage = Total score/ 22 x 100**	

Each row gets a score of 1 if the mentioned test is available otherwise 0.

Scoring chart	
**Total percentage**	**Score**
0-50	0
50-70	1
70-85	2
85-100	3
**Score for Standard 2.8.8.2**	

Annex 2.8m Medicines for Anesthesia

S.No.	List of Medicines	Required Number	Score
Preoperative medications			
1.	Ranitidine Injection	5	
2.	Metoclopramide Injection	5	
3.	Aluminium hydroxide or magnesium trisilicate suspension	5	
4.	Atropine Injection	10	
5.	Diazepam Tablet	5	
Intraoperative medications			
6.	Ketamine Injection	3	
7.	Midazolam Injection	3	
8.	Opioid analgesics injections (Morphine, Pethidine, Fentanyl)	2 each	
9.	Lignocaine 2% Injection for IV infusion	2	
10.	Lignocaine Inj 1%, 2% with or without Adrenaline 1:200000	2	
11.	Thiopental Powder 500mg	As per need	
12.	Propofol Injection	As per need	
13.	Appropriate inhalational anesthetic (e.g., Halothane, Isoflurane, Sevoflurane)	As per need	
14.	Succinylcholine Injection	As per need	
15.	Appropriate non-depolarizing muscle relaxant (pancuronium, vecuronium, rocuronium, atracurium)	As per need	
16.	Neostigmine Injection	As per need	
17.	Atropine Injection / Glycopyrolate Injection	10/10	

18.	Bupivacaine Heavy 0.5%	5	
Intravenous fluids			
19.	Water for injection	As per need	
20.	Normal saline / Ringer's lactate	As per need	
21.	5% Dextrose / Dextrose normal saline	As per need	
22.	1/5Dextrose 1/3Normal saline	As per need	
23.	Mannitol 20% Injection	As per need	
24.	Haemaccel Injection / Gelafusine Injection / Voluven Injection	As per need	
Resuscitative medications			
25.	Dextrose 25%/ 50% Injection	5	
26.	Mephenteramine or Ephedrine Injection	5	
27.	Dopamine injection	5	
28.	Noradrenaline injection	5	
29.	Amiodarone injection	5	
30.	Hydrocortisone injection	5	
31.	Dexomethasone injection	5	
32.	Chlorpheniramine injection	5	
33.	Calcium gluconate injection	5	
34.	Beta-blockers (Metoprolol, Labetolol, Esmolol) Injection	As per need	
35.	Naloxone Injection	5	
Post-operative medications			
36.	Morphine Injection	As per need	
37.	Pethidine Injection	As per need	
38.	Tramadol Injection	As per need	
39.	Pentazocine Injection	As per need	
40.	Paracetamol Injection 1gm, Suppository 125mg	As per need	
41.	Diclofenac Injection	As per need	
42.	Ketorolac Injection	As per need	
43.	Promethazine Injection	As per need	
44.	Ondansetron Injection	As per need	
45.	Gabapentin Injection	As per need	

Other medications			
46.	Magnesium Injection	As per need	
47.	Salbutamol Injection (for inhalation)	As per need	
48.	Ipratropium bromide Injection (for inhalation)	As per need	
49.	Furosemide Injection	As per need	
50.	Glyceryl trinitrate/nitroglycerine Injection	As per need	
51.	Sodium nitroprusside Injection	As per need	
52.	Heparin Injection	As per need	
53.	Aminophylline Injection	As per need	
		**Total Score**	
		**Total percentage = Total score/ 53 x 100**	

Each row gets a score of 1 if the mentioned test is available otherwise 0.

**Scoring chart**	
**Total percentage**	**Score**
0-50	0
50-70	1
70-85	2
85-100	3
**Score for Standard 2.8.8.3**	

**Area**	**Code**	**Verification**		
**Hemodialysis service**	**2.9**			
**Components**		**Standards**	**Obtained Score**	**Maximum Score**
2.9.1 Time for patients	2.9.1.1	Hemodialysis service is available from 9 am to 5 pm and appointments of patient scheduled		1
	2.9.1.2	Emergency hemodialysis is available round the clock		1
	2.9.1.3	Under privilege citizen facilities as per norms of government of Nepal		1

2.9.2 Space for hemodialysis service	2.9.2.1	Separate block/rooms designated for hemodialysis with space for at least two hemodialysis beds and machines and a working.		1
	2.9.2.2	The dialysis station is easily accessible in time of emergency and have adequate space for resuscitation to be carried out.		1
	2.9.2.3	There is designated space for dialyzer reprocessing and proper cleaning and preparation of dialysers for reuse done based on protocol		1
	2.9.2.4	There is designated space for medicine preparation and storage of medicines, equipment, instruments and supplies in shelves with separate dry and wet storage shelves		1
	2.9.2.5	Separate bed is available for rest to patient after dialysis or to give injection on OPD as erythropoietin, cyclophosphamide, and others.		1
2.9.3 Staffing	2.9.3.1	There should be at least one haemodialysis trained medical officer with on call MD Internal Medicine or Nephrologist and one nurse per two dialysis machine per shift		1
	2.9.3.2	All hemodialysis staffs are trained on BLS, ACLS and basic maintenance of hemodialysis machine.		1
	2.9.3.3	Biomedical equipment trained technician is available any time in case of technical emergencies		1
2.9.4 Patient counseling	2.9.4.1	Counselling to the patient and attendant is done by multi-disciplinary team to adhere the patient to the treatment therapy including dietary counseling and follow up		1
	2.9.4.2	Proper care and monitoring of the Vascular access is done during treatment and patient counseled about its care		1
2.9.5 Equipment, instruments, drugs and consumables available	2.9.5.1	Dialysis machines is equipped with monitors and audio-visual alarms to ensure safe dialysis.		1
	2.9.5.2	Dedicated hemodialysis area and dedicated dialysis machines for patients with hepatitis B, Hepatitis C and PLHA		1
	2.9.5.3	Adequate medical equipment and instruments available. (See Annex 2.9aList of medical equipment and instruments for hemodialysis At the end of this standard)		3
	2.9.5.4	Adequate drugs and consumables available (See Annex 2.9b Medicines and supplies for hemodialysis At the end of this standard)		3

2.9.6 Physical Facility	2.9.6.1	Product water is free from harmful chemicals and bacterial contamination with reverse osmosis done		1
	2.9.6.2	Water used to prepare the dialysate has colony count of less than 100CFU/ml. (AAMI Standard)		1
	2.9.6.3	Recommended water quality by provision of water analysis for bacteria at least monthly and chemical at least six monthly.		1
	2.9.6.4	Endotoxin test of RO water and dialysate is performed in annual basis.		1
	2.9.6.5	Power back up supply is ensured in hemodialysis unit at least for reverse osmosis and dialysis machine		1
	2.9.6.6	Plumbing is installed in manner as to prevent back flow of the dialysate drainage		1
	2.9.6.7	There is adequate light, well ventilation and suitable temperature maintained		1
	2.9.6.8	Separate toilets for male and female staff with at least one universal toilet		1
2.9.7 Inventory maintained	2.9.7	Biomedical equipment log book is maintained along with preventive maintenance records		1
2.9.8 Handover and takeover of critical patients	2.9.8	There is provision of transporting patient for transfer/referral in safe manner accompanied by at least one mid-level health worker and if needed medical officer		1
2.9.9 Recording and reporting	2.9.9.1	Medical record is maintained in register and digital format.		1
	22.9.9.2	Dialysis schedule of the patient is maintained in board along with time.		1
2.9.10 Infection control measures followed in hemodialysis service	2.9.10.1	Personal protective equipment as gown, mask, face shield as per requirement.		1
	2.9.10.2	Dialyzers and AV blood lines of PLHA, Hepatitis B and Hepatitis C positive is discarded after each use.		1
	2.9.10.3	Rinsing of machine is done after each shift and disinfection is done as per protocol		1
	2.9.10.4	Dedicated shoes and gown is available in entrance of dialysis unit.		1
	2.9.10.5	Autoclave of infected waste before disposal		1
	2.9.10.6	There are colored bins for waste segregation and disposal as per HCWM guideline 2014 (MoHP)		1
	2.0.10.7	Hand-washing facility with running water and liquid soap is available for practitioners		1
	2.9.10.8	Needle cutter is used		1
	2.9.10.9	Chlorine solution is available and utilized for decontamination.		1
**Standard 2.9**		**Total Obtained Score**		42
		**Total Percentage (Total Obtained Score/ 42 x100)**		

Annex 2.9a Medical equipment and instrument for hemodialysis

S.N.	Medical equipment and instrument	Required No	Score
1	Hemodialysis machine	at least 2	
2	Blood Pressure Apparatus	2	
3	Stethoscope	2	
4	Thermometer(Patient and Refrigerator)	2	
5	Glucometer	2	
6	Hemodialysis set	2	
7	Curtain	as per need	
8	Wheel Chair	at least 2	
9	Stretcher	at least 1	
10	Bed	as least 2	
11	Bed side Tray	1 in each bed	
12	Standby Rechargeable light	1	
13	Hygrometer	1	
	Resuscitation set	at least 1	
14	Suction Apparatus	at least 1	
15	Oxygen cylinder with flow meter,nasal prongs and mask or central oxygen	at least 1/ available	
16	Defibrillator	at least 1	
17	ECG machine	at least 1	
18	Pulse oximeter	at least 1	
19	Nebulizer	at least 1	
20	Cardiac monitor	at least 1	
21	Torch light	at least 1	
		**Total score**	
		**Total percentage= Total score/21 x 100**	

Each row gets a score of 1 if all the required number is available otherwise 0.

**Scoring Chart**	
**Total Percentage**	**Score**
0-50	0
50-70	1
70-85	2
85-100	3
**Score for Standard 2.9.5.3**	

Annex 2.9b Medicines and Supplies for Hemodialysis

S.No	Medicines and supplies	Required No	Score
1	Diazepam Injection	5	
2	Frusemide Injection	5	
3	Ondansetron Injection	5	
4	Ranitidine Injection	5	
5	Noradrenaline Injection	5	
6	Phenytoin Injection	5	
7	Diclofenac sodium Injection	5	
8	Deriphylline Injection	5	
10	Hydrocortisone Injection	5	
11	Atropine Injection	5	
12	Adrenaline Injection	5	
13	Potassium Chloride (KCL) Injection	5	
14	Pheniramine Injection	5	
15	Sterile Water	5	
16	Soda bicarbonate Injection	5	
17	Dopamine Injection	5	
18	Calcium Gluconate Injection	5	
19	Dextrose 25 %/ 50% Injection	5each	
21	Tranxemic Acetate Injection	5	
22	Protamine Sulphate Injection	5	
23	Vitamin K Injection	5	
24	Tramadol Injection	5	
25	Hyoscine Butylbromide Injection	5	
26	Aspirin Tablet	1 strip	
27	Clopilet Tablet	1 strip	
28	Isodril Tablet	1 stirp	
29	Nefedipin 5 mg/10mg Cap	1 strip each	
30	Injection Heparin	as per need	
Dialysis Consumables			

31	Adhesive Tape	as per need	
32	Leukoband	as per need	
33	Paper Tape	as per need	
34	Betadine	as per need	
35	Spirit	as per need	
36	Dialyzer	as per need	
37	A/V Tubing	as per need	
38	Fistula Needle	as per need	
39	I/V Set	as per need	
40	I/V Cannula different size	as per need	
41	Transducer	as least 2	
42	Sub clavien Catheter	at least 2	
43	Femoral Catheter	at least2	
44	Guide wire	at least 2	
45	Normal Saline 1000ml	as per need	
46	Normal Saline 500ml	at least 5	
47	Disposable Syringe 20ml	as least 5	
48	Disposable Syringe 10ml	at least 5	
49	Disposable Syringe 5ml	at least 5	
50	Rubber Sheet	as per need	
**Total Score**			
**Total percentage= Total Score/50 x100**			

Each row gets a score of 1 if all the required number is available otherwise 0.

**Scoring Chart**	
**Total Percentage**	**Score**
0-50	0
50-70	1
70-85	2
85-100	3
**Score for Standard 2.9.5.4**	

Area	Code	Verification		
**Intensive Care Services**	**2.10**			
**Intensive care unit (ICU)**	**2.10.1**			
**Components**		**Standards**	**Obtained Score**	**Maximum Score**
2.10.1 ICU service for both inpatient and referral cases	2.10.1.1	ICU service is available for inpatients and referral critical cases round the clock		1
	2.10.1.2	Minimum number of ICU beds is 5% of total hospital beds		1
	2.10.1.3	One ICU set up has at least 8 ICU beds		1
Adequate physical facilities				
2.10.1.2 Strategic location of ICU in hospital	2.10.1.2.1	ICU must have easy access and connectivity with operation theatre complex, emergency department, radio-imaging and clinical lab.		1
	2.10.1.2.2	ICU must have a single entry and exit point to allow free access to heavy duty machines like mobile x-ray, bed and trolleys on wheels		1
	2.10.1.2.3	There must be at least two barriers to the entry of ICU		1
	2.10.1.2.3	Separate designated space in ICU for		
	2.10.1.2.4	Family waiting area with chairs at least one for each ICU bed		1
	2.10.1.2.5	Counselling room with working desk and chairs		1
	2.10.1.2.6	Central Nursing Station with working desk, centrally connected monitors display of each bed and at least three chairs		1
	2.10.1.2.7	Nurse's Room with tea room facility and reference books in shelves		1
	2.10.1.2.7	Doctor's Room with tea room facility and reference books in shelves		1
	2.10.1.2.8	Utility Room with separate dirty and clean linen storage		1
	2.10.1.2.9	Changing Room (Separate for male and female) with adequate number of lockers for staffs' belongings		1
	2.10.1.2.10	Wash room (Separate for male and female with at least one universal)		1
	2.10.11	Proper bed area allocated for each bed with supplies (See Annex 2.10.1a Proper Bed Area for ICU At the end of the standard)		3
	2.10.1.2.12	Lighting: Access to natural light		1
	2.10.1.2.13	ICU must have air conditioner to maintain the temperature, humidity and air flow and closed to outer environment		1
	2.10.1.2.14	High illumination spot lighting for procedures, like putting central lines etc.		1
	2.10.1.2.15	There must be proper fire extinguishing machines.		1

2.10.1.3 Staffing	2.10.1.3.1	ICU has staffing as per annex . (See Annex 2.10.1b Staffing of Intensive care services At the end of the standard)		3
	2.10.1.3.2	There must be one ICU in-charge (Nursing officer) with minimum of bachelor in critical care or trained in critical care for overall management of ICU		1
2.10.1.4 Equipment and instruments available and functioning	2.10.1.4	ICU has adequate equipment and instruments to function adequately (See Annex 2.10.1c Equipment and Instrument for intensive care services At the end of the standard)		3
2.10.1.5 Duty rosters	2.10.1.5	Duty roster for 24 hours is prepared and placed in visible area for all ICU staffs including doctors and nurses		1
2.10.1.6 ICU protocol in place and followed	2.10.1.6.1	ICU must practice given protocols on all given clinical conditions with all staffs in ICU trained in Basic Life Support, Advance Life Support, Critical Care Support, Mechanical Ventilation, Infection prevention, Therapeutic Feeding		1
	2.10.1.6.2	All ICUs must be designed to handle disasters both within ICU and outside the ICU		1
2.10.1.7 Recording and reporting	2.10.1.7.1	Separate sheet tailored for ICU set up is used for continuous monitoring of the patients and e-recording done in standard software approved by MoHP		1
	2.10.1.7.2	Handover and takeover of the patients from ER or other wards is done with patient being received in ICU accompanied by respective ward at least staff nurse or paramedics		1
	2.10.1.7.3	Complications, morbidity and mortality are recorded, reported and proceeded for clinical audit on regular basis (at least weekly)		1
2.10.1.8 Infection prevention	2.10.1.8.1	10% of beds (1 to 2) should be separated as isolation beds in each ICU		1
	2.10.1.8.2	Hand Hygiene protocol developed and followed between each ICU bed with alcohol hand rubs or sanitizer		1
	2.10.1.8.3	Separate space designated for scrub station with running water and liquid soap, elbow lever and hand drier available and functional		1
	2.10.1.8.4	Waste disposal as per HCWM guideline 2014 (MoHP)		1
	2.10.1.8.5	Gown and slippers for doctors, nurses, visitors		1
	2.10.1.8.6	Chlorine solution is available and utilized for decontamination		1
**Standard 2.10.1**		**Total obtained score**		40
		**Total percentage= Total obtained score/40 x 100**		

Neonatal Intensive care unit	2.10.2	Verification		
**Components**		**Standards**	**Obtained Score**	**Maximum Score**
2.10.2.1 NICU service for both inpatient and referral cases	2.10.2.1.1	NICU service is available for inpatients and referral critical cases round the clock		1
	2.10.2.1.2	Minimum number of NICU beds is 5% of total hospital beds		1
	2.10.2.1.3	One NICU set up has at least 5 NICU beds		1
2.10.2.2 Adequate physical facilities	2.10.2.2.1	NICU must have easy access and connectivity with operation theatre complex, emergency department, radio-imaging and clinical lab.		1
	2.10.2.2.2	NICU must have a single entry and exit point to allow free access to heavy duty machines like mobile x-ray, bed and trolleys on wheels		1
	2.10.2.2.3	There must be at least two barriers to the entry of NICU		1
	2.10.2.2.4	Separate designated space in NICU for		
	2.10.2.2.4.1	Family waiting area with chairs at least one for each NICU bed		1
	2.10.2.2.4.2	Counselling room with working desk and chairs		1
	2.10.2.2.4.3	Breast feeding room with comfortable chair and air conditioning for mother to feed or express breast milk		1
	2.10.2.2.4.4	Central Nursing Station with working desk, centrally connected monitors display of each bed and at least three chairs		1
	2.10.2.2.4.5	Nurse's Room with tea room facility and reference books in shelves		1
	2.10.2.2.4.6	Doctor's Room with tea room facility and reference books in shelves		1
	2.10.2.2.4.7	Utility Room with separate dirty and clean linen storage		1
	2.10.2.2.4.8	Changing Room (Separate for male and female) with adequate number of lockers for staffs' belongings		1
	2.10.2.2.4.9	Wash room (Separate for male and female with at least one universal)		1
	2.10.2.2.4.10	Lighting: Access to natural light		1
	2.10.2.2.4.11	NICU must have air conditioner to maintain the temperature, humidity and air flow and closed to outer environment		1
	2.10.2.2.4.12	High illumination spot lighting for procedures, like putting central lines etc.		1
	2.10.2.2.4.13	There must be proper fire extinguishing machines.		1
2.10.2.3 Staffing	2.10.2.3	NICU has staffing as per annex (See Annex 2.10.1b Staffing of Intensive care services At the end of this standard)		3

2.10.2.4 Equipment and instruments available and functioning	2.10.2.4	NICU has adequate equipment and instruments to function adequately (See Annex 2.10.1c Equipment and Instrument for intensive care services At the end of this standard)		3
2.10.2.5 Duty rosters	2.10.2.5	Duty roster for 24 hours is prepared and placed in visible area for all NICU staffs including doctors and nurses		1
2.10.2.6 NICU protocol in place and followed	2.10.2.6.1	NICU must practice given protocols on all given clinical conditions with all staffs in NICU trained in Basic Life Support, Pediatric Advance Life Support, Critical Care Support, Mechanical Ventilation, Infection prevention, Tube feeding, Incubators, Warmer, Phototherapy		1
	2.10.2.6.2	All NICUs must be designed to handle disasters both within NICU and outside the NICU		1
2.10.2.7 Recording and reporting	2.10.2.7.1	Separate sheet tailored for NICU set up is used for continuous monitoring of the patients and e-recording done in standard software approved by MoHP		1
	2.10.2.7.2	Handover and takeover of the patients from ER or other wards is done with patient being received in NICU accompanied by respective ward at least staff nurse or paramedics		1
	2.10.2.7.3	Complications, morbidity and mortality are recorded, reported and proceeded for clinical audit on regular basis (at least weekly)		1
2.10.2.8 Infection prevention	2.10.2.8.1	10% of beds (1 to 2) should be separated as isolation beds in each NICU		1
	2.10.2.8.2	Hand Hygiene protocol developed and followed between each NICU bed with alcohol hand rubs or sanitizer		1
	2.10.2.8.3	Separate space designated for scrub station with running water and liquid soap, elbow lever and hand drier available and functional		1
	2.10.2.8.4	Every neonate must be properly cleaned / wiped everyday by using approved solution.		1
	2.10.2.8.5	Use of personal protective equipment while caring each neonate to prevent cross infection.		1
	2.10.2.8.6	Waste disposal as per HCWM guideline 2014 (MoHP)		1
	2.10.2.8.7	Gown and slippers for doctors, nurses, visitors		1
	2.10.2.8.8	Chlorine solution is available and utilized for decontamination		1
**Standard 2.10.2**		**Total obtained score**		39
		**Total percentage= Total obtained score/39 x 100**		

Pediatric Intensive care unit	2.10.3	Verification		
**Components**		**Standards**	**Obtained Score**	**Maximum Score**
2.10.3.1 PICU service for both inpatient and referral cases	2.10.3.1.1	PICU service is available for inpatients and referral critical cases round the clock		1
	2.10.3.1.2	One PICU set up has at least 5 PICU beds		1
2.10.3.2 Adequate physical facilities	2.10.3.2.1	PICU must have easy access and connectivity with operation theatre complex, emergency department, radio-imaging and clinical lab.		1
	2.10.3.2.2	PICU must have a single entry and exit point to allow free access to heavy duty machines like mobile x-ray, bed and trolleys on wheels		1
	2.10.3.2.3	There must be at least two barriers to the entry of PICU		1
	2.10.3.2.4	Separate designated space in PICU for		
	2.10.3.2.5	Family waiting area with chairs at least one for each PICU bed		1
	2.10.3.2.6	Counselling room with working desk and chairs		1
	2.10.3.2.7	Central Nursing Station with working desk, centrally connected monitors display of each bed and at least three chairs		1
	2.10.3.2.8	Nurse's Room with tea room facility and reference books in shelves		1
	2.10.3.2.9	Doctor's Room with tea room facility and reference books in shelves		1
	2.10.3.2.10	Utility Room with separate dirty and clean linen storage		1
	2.10.3.2.11	Changing Room (Separate for male and female) with adequate number of lockers for staffs' belongings		1
	2.10.3.2.12	Wash room (Separate for male and female with at least one universal)		1
	2.10.3.2.13	Proper bed area allocated for each bed with supplies		1
	2.10.3.2.14	Lighting: Access to natural light		1
	2.10.3.2.15	PICU must have air conditioner to maintain the temperature, humidity and air flow and closed to outer environment		1
	2.10.3.2.16	High illumination spot lighting for procedures, like putting central lines etc.		1
	2.10.3.2.17	There must be proper fire extinguishing machines.		1
2.10.3.3 Staffing	2.10.3.3	PICU has staffing as per annex. (See Annex 2.10.1b Staffing of Intensive care services At the end of this standard)		3

2.10.3.4 Equipment and instruments available and functioning	2.10.3.4	PICU has adequate equipment and instruments to function adequately (See Annex 2.10.1c Equipment and Instrument for intensive care services At the end of this standard)		3
2.10.3.5 Duty rosters	2.10.3.5	Duty roster for 24 hours is prepared and placed in visible area for all PICU staffs including doctors and nurses		1
2.10.3.6 PICU protocol in place and followed	2.10.3.6.1	PICU must practice given protocols on all given clinical conditions with all staffs in PICU trained in Basic Life Support, Advance Life Support, Critical Care Support, Mechanical Ventilation, Infection prevention, Therapeutic Feeding		1
	2.10.3.6.2	All PICUs must be designed to handle disasters both within PICU and outside the PICU		1
2.10.3.7 Recording and reporting	2.10.3.7.1	Separate sheet tailored for PICU set up is used for continuous monitoring of the patients and e-recording done in standard software approved by MoHP		1
	2.10.3.7.2	Handover and takeover of the patients from ER or other wards is done with patient being received in PICU accompanied by respective ward at least staff nurse or paramedics		1
	2.10.3.7.3	Complications, morbidity and mortality are recorded, reported and proceeded for clinical audit on regular basis (at least weekly)		1
2.10.3.8 Infection prevention	2.10.3.8.1	10% of beds (1 to 2) should be separated as isolation beds in each PICU		1
	2.10.3.8.2	Hand hygiene protocol developed and followed between each PICU bed with alcohol hand rubs or sanitizer		1
	2.10.3.8.3	Separate space designated for scrub station with running water and liquid soap, elbow lever and hand drier available and functional		1
	2.10.3.8.4	Every neonate must be properly cleaned / wiped everyday by using approved solution.		1
	2.10.3.8.5	Use of personal protective equipment while caring each neonate to prevent cross infection.		1
	2.10.3.8.6	Waste disposal as per HCWM guideline 2014 (MoHP)		1
	2.10.3.8.7	Gown and slippers for doctors, nurses, visitors		1
	2.10.3.8.8	Chlorine solution is available and utilized for decontamination		1
**Standard 2.10.3**		**Total obtained score**		38
		**Total percentage= Total obtained score/38 x 100**		

Annex 2.10.1a Proper Bed Area for ICU

S.No.	Appropriate bed space and supplies	Score
1.	100 sq. ft per patient care area	
2.	Bed length: 7 ft; Bed width: 3.5 ft	
3.	The head end of the bed must be kept at least 2 ft from the wall to have adequate access for endotracheal intubation, resuscitation, and central venous catherization	
4.	The foot end of the bed must be kept at least 3 ft from the corridor or wall.	
5.	Space between the two adjacent beds: 5 ft	
6.	Wall or ceiling mounted pendants to reduce the space requirements and to provide hindrance free and smooth accessibility at the head end of the bed.	
7.	Utilities per bed in the pendant: 2 oxygen outlets with flow meters, 2 vaccuum, 8 universal electric outlets distributed on both sides of the bed.	
	**Total Score**	
	**Total percentage= Total Score/6 x 100**	

Each row gets a score of 1 if all the required number is available otherwise 0.

**Scoring Chart**	
**Total Percentage**	**Score**
0-50	0
50-70	1
70-85	2
85-100	3
**Score for Standard2.10.1.11**	

Annex 2.10.1b Staffing for Intensive Care Services

S.No.	Staffing of ICU	Required Number	ICU	NICU	PICU
1.	ICU Coordinator with at least MD Anesthesiology (dedicates 50% professional time for ICU)	1			
2.	NICU/ PICU Coordinator with at least MD Pediatrics (dedicates 50% professional time for NICU/PICU)				
3.	Admitting consultant on duty	1			
4.	One trained medical officer for each 5 bed	1 per shift			
5.	Nurse in-charge with 5 years' experience in ICU with at least Nursing officer (Bachelors' degree in critical care/ critical care trained)	1			
6.	Nurse: patient ventilated and multi-organ failure patients	1:1			
7.	Nurse: Patients ventilated or multi-organ failure patients	2:3			
8.	Nurse: Patients less seriously sick patients who do not require above modalities.	1:2			
9.	IP trained office assistants	1in each shift for 5 bedded ICU			
10.	Security staffs	2 in each shift			
11.	Biomedical technician/engineer on duty	At least one			
		**Total Score**			
		**Total percentage= Total Score/10 x 100**			

Each row gets a score of 1 if all the required number is available otherwise 0.

**Scoring Chart**	
**Total Percentage**	**Score**
0-50	0
50-70	1
70-85	2
85-100	3
**Score for Standard 2.10.1.3.1**	
**Score for Standard 2.10.2.2.5.1**	
**Score for Standard 2.10.3.4.1**	

Annex 2.10.1c Equipment and Instrument for Intensive Care Services

S.N.	Equipment, Instrument and Supplies for ICU	Required number	Score		
			ICU	NICU	PICU
1.	ICU bed (With mattress, two iv stands and all position possible: height adjustment, back section and leg section adjustment, tredelenberg and reverse trendelenberg position)	one per bed			
2.	Bedside Patient monitor (Modular - ECG, SPO2, NIBP, RR, Temp Probes with trays) upgradable to invasive BP monitoring	one per bed			
3.	Bedside Patient monitor (Modular - ECG, SPO2, NIBP, 2 Invasive BP, RR, Temp Probes with trays)	two out of five monitors			
4.	ICU Ventilator (With paediatric and adult provisions, graphics and Non-Invasive Modes, Humidifier, inbuilt nebulisation, turbine/air-compressor)	one per bed			
5.	BiPAP Machine	two for five beds			
6.	Defibrillator (manual and automated with transcutaneous pacing facility)	One			
7.	Syringe pumps	4 per bed			
8.	Infusion pumps	1 per bed			
9.	Over Bed Table	1 per bed			
10.	Bedside Cabinet	1 per bed			
11.	Handheld Pulse oximeter	2			
12.	ABG Machine	1			
13.	Hemodialysis Machine	1			
14.	Intermittent Leg Compressing Device to prevent DVT	1 per bed			
15.	Air mattress	1 per bed			
16.	Crash/Resuscitation trolley	1			
17.	Glucometer	2			
18.	Portable X-ray machine	1			
19.	Clinical Lab facility with lactate value, culture and sensitivity	Available			
20.	Warming devices: blanket, blower	As per need			
		**Total Score**			
		**Total percentage= Total Score/ 20x 100**			

Each row gets a score of 1 if all the required number is available otherwise 0.

Scoring Chart	
**Total Percentage**	**Score**
0-50	0
50-70	1
70-85	2
85-100	3
**Score for Standard 2.10.1.4**	
**Score for Standard 2.10.2.3**	
**Score for Standard 2.10.3.5**	

Area	Code	Verification		
**Diagnostics and laboratory**	**2.11**			
**Laboratory**	**2.11.1.1**			
**Components**		**Standards**	**Obtained Score**	**Maximum Score**
2.11.1.1.1Time for patients	2.11.1.1.1.1	Laboratory is open from 10 AM to 3 PM for routine services and separate emergency lab service available round the clock		1
	2.11.1.1.1.2	Basic investigations are available (See Annex 2.11.1a List of investigations for Laboratory At the end of this standard)		3
2.11.1.1.2 Staffing	2.11.1.1.2.1	Laboratory team is lead by pathologist (at least) 3 (one for histocytopathology and hematology, 1 for biochemistry and 1 for microbiology)		1
	2.11.1.1.2.2	At least 12 lab staffs (4 medical technologists, 4technicians, 2 assistants and 2 helpers are available during routine lab		1
	2.11.1.1.2.3	At least 3 staffs (1 Technician, 1 Assistant and 1 Helper) in each shift in emergency lab		1
	2.11.1.1.2.4	On call biomedical engineer available for maintenance of lab equipment		1
2.11.1.1.3 Instruments and equipment	2.11.1.1.3.1	Instruments and equipment to carry out all parameters of tests are available and functioning(See Annex 2.11.1.1b Equipment and Instrument for Lab At the end of this standard)		3
	2.11.1.1.3.2	Instrument are maintained and calibrated as per manufacturer instructions		1
	2.11.1.3.3	Quality control sera and standards are run regularly and record kept		1

2.11.1.1.4 Physical facilities	2.11.1.1.4.1	Separate space with working desk and chair designated for specific laboratory procedures like- hematology, biochemistry, microbiology, serology, histopathology and cytology		1
	2.11.1.1.4.2	Light and ventilation are adequately maintained.		1
	2.11.1.1.4.3	Designated area well labelled for reception of sample and dispatch of reports		1
	2.11.1.1.4.4	Power back up is available for the lab for preservation of sample and regents		1
2.11.1.1.5 Duty rosters	2.11.1.1.5	Duty rosters of lab are developed regularly and available in appropriate location.		1
2.11.1.1.6 Facilities for patients	2.11.1.1.6.1	Waiting space with sitting arrangement is available for at least 15 persons in waiting lobby.		1
	2.11.1.1.6.2	At least one each male, female and universal toilet for patients using laboratory services		1
	2.11.1.1.6.3	Safe drinking water is available in the waiting lobby throughout the day.		1
2.11.1.1.7 Recording and reporting	2.11.1.1.7.1	Laboratory Information System (LIS) available		1
	2.11.1.1.7.2	Major instruments are interfaced		
	2.11.1.1.7.3	Sample is adequately recorded with requisition form with detail information of patients		1
	2.11.1.1.7.4	Copy of computerized report is kept safe (hard and soft copies) for future use till 6 months and report is available to patient		1
	2.11.1.1.7.5	Report have adequate information of patient and checked by designated person before release		1
2.11.1.1.8 Supplies storage and stock	2.11.1.1.8.1	At least three months buffer stock of laboratory supplies is available.		1
	2.11.1.1.8.2	Reagents are stored at appropriate temperature in store and lab		1
2.11.1.1.9 Blood Bank within hospital premises	2.11.1.1.9	Blood bank should be available inside hospital premises either owned by hospital or Nepal Red Cross Society<br>(If hospital has its own blood bank refer to standard 2.11.1.2)		1

2.11.1.1.10 Infection prevention	2.11.1.1.10.1	Closed vacuum system is used for sample collection		1
	2.11.1.1.10.2	Biohazard signs and symbols are used at appropriate places visibly		1
	2.11.1.1.10.3	All staffs know how to respond in case of spillage and other incidents		1
	2.11.1.1.10.4	Masks and gloves are available		1
	2.11.1.1.10.5	There are colored bins for waste segregation based on HCWM guideline 2014 (MoHP) and infectious waste is sterilized using autoclave before disposal		1
	2.11.1.1.10.6	Hand-washing facility with running water and soap is available for practitioners		1
	2.11.1.1.10.7	Needle cutter is used		1
	2.11.1.1.10.8	Chlorine solution and bleach is available and utilized for decontamination		1
**Standard 2.11.1.1**		**Total Obtained Score**		36
		**Total Percentage (Total Obtained Score/ 36 x100)**		

Annex 2.11.1.1a List of Investigations for Laboratory

**SN**	**Test**	**Routinely available**
Hematology		
1.	Hb	
2.	Total Leucocyte count	
3.	Differential leucocyte count	
4.	ESR	
5.	Blood grouping for non transfusion	
6.	Blood grouping for transfusion	
7.	Bleeding time	
8.	PT	
9.	APTT	
10.	Platelet count	
11.	MCV	
12.	MCH	
13.	MCHC	

14.	Hematocrit (PCV)	
15.	Malaria RDT or microscopy	
16.	Absolute count	
17.	Reticulocyte	
18.	Peripheral smear examination	
19.	D dimer	
20.	Sickling test	
21.	Bone marrow for hematological diseases and LD bodies	
22.	Blood Sugar	
23.	Urea	
24.	Creatinine	
25.	Billirubin total	
26.	Billirubin direct	
27.	Serum Uric acid	
28.	Total Protein	
29.	Serum albumin	
30.	SGOT	
31.	SGPT	
32.	Alkaline phosphatase	
33.	Triglyceride	
34.	Total Cholesterol	
35.	HDL	
36.	LDL	
37.	Serum Sodium	
38.	Serum Potassium	
39.	HbA1c	
40.	Beta hCG	
41.	Urine microalbumin	
42.	Urine albumin creatinine ratio	
43.	Total CK	

44.	CK-MB	
45.	Troponin T/I	
46.	Amylase	
47.	Lipase	
48.	Thyroid function test	
49.	Anti TPO	
50.	LDH	
51.	CEA	
52.	CA125	
53.	CA19.9	
54.	Serum Beta HCG	
55.	LH	
56.	FSH	
57.	Estradiole	
58.	Serum ferritin	
59.	Serum Iron	
60.	TIBC	
61.	Vitamin D3	
Microbiology		
62.	Sputum AFB	
63.	KOH mount	
64.	Routine bacteriology culture (blood, urine, pus, body fluid, swab)	
65.	Antibiotic susceptibility	
66.	Gram stain	
Serology		
67.	RPR	
68.	Widal	
69.	ASO	
70.	RA factor	
71.	CRP	
72.	rK39 (kit)	

73.	Montoux test	
74.	TPHA (rapid)	
75.	HbsAg (rapid)	
76.	HCV(rapid)	
77.	HIV(rapid)	
78.	HIV (other than rapid)	
79.	HbsAg(other than rapid)	
80.	Anti HCV(other than rapid)	
81.	Anti HBc(other than rapid)	
82.	HBe antigen	
83.	ANA	
84.	Cryptococcal antigen test	
Miscellaneous		
85.	Urine routine and microscopy	
86.	Urine Pregnancy Test	
87.	Stool routine and microscopy	
88.	Stool for occult blood	
89.	Stool for reducing substance	
90.	Urine ketone bodies	
91.	CSF and body fluid examination (sugar, protein, total and differential count)	
92.	Semen analysis (total count and motility)	
Histo/cytopathology		
93.	Pap smear examination	
94.	Sputum cytology	
95.	Body fluid cytology	
96.	FNAC	
97.	Histopathology	
	**Total Score**	
	**Total Percentage = Total Score/ 97 x 100**	

Each row gets a score of 1 if all the required number is available otherwise 0.

Scoring chart	
**Total percentage**	**Score**
0-50	0
50-70	1
70-85	2
85-100	3
**Score for Standard 2.11.1.1.1.2**	

Annex 2.11.1.1b Equipment and Instrument for Laboratory

S.N.	Name of Instruments	Required Quantity	Score
1.	Microscope	3	
2.	Fully automated biochemistry analyser	1	
3.	Fully automated hematology analyser	1	
4.	CLIA/ECL	1	
5.	Incubator	1	
6.	Biosafety cabinet (for microbiology)	1	
7.	Chemical Balance	1	
8.	Electrolyte Analyzer	1	
9.	HBA1c measuring instrument (semiautomated/ automated)	1	
10.	Hot air Oven	1	
11.	Refrigerator	1-2	
12.	Centrifuge	1-2	
13.	Counting Chamber	1-2	
14.	DLC counter	1-2	
15.	Tissue Processor	1	
16.	Microtome	1	
17.	Liquid based cytology machine	1	
18.	Pipettes, Glassware/kits	As per need	
19.	Computer with printer	1	
20.	Water Bath	1	
21.	Tissue flotation bath	1	

22.	Disposable test tubes	As per need	
23.	Different Closed Vacuum set (for sample)- hematology, biochemistry	As per need	
24.	Histo/cyto staining boxes with stains	As per need	
25.	Autoclave for waste disposal (250 liter, pre-vacuum with horizontal outlet)	1	
		**Total Score**	
		**Total percentage = Total Score/ 25 x 100**	

Each row gets a score of 1 if all the required number is available otherwise 0.

**Scoring chart**	
**Total percentage**	**Score**
0-50	0
50-70	1
70-85	2
85-100	3
**Score for Standard 2.11.1.1.3.1**	

**Area**	**Code**	**Verification (*Applicable if hospital has its own blood bank)**		
**Blood Bank***	**2.11.1.2**			
**Components**		**Standards**	**Obtained Score**	**Maximum Score**
2.11.1.2.1 Time for patients	2.11.1.2.1	Blood bank is open / facility is available round the clock		1
2.11.1.2.2 Staffing	2.11.1.2.2	Adequate numbers of trained healthcare workers are available in blood bank (at least 2 blood bank staffs to cover shifts including ER)		1
2.11.1.2.3 Physical facilities	2.11.1.2.3.1	Adequate rooms and space for the staffs and patients are available (area of more than 10 meter squares)		1
	2.11.1.2.3.2	Light and ventilation are adequately maintained.		1
	2.11.1.2.3.3	The required furniture and supplies are available (See Annex 2.11.1.2a Blood Bank Furniture and Supplies At the end of the standard)		3
	2.11.1.2.3.4	Thermometers are attached to all equipment requiring temperature control and temperatures are recorded daily or temperature sensor based equipment		1

2.11.1.2.4 Instruments and equipment	2.11.1.2.4	Instruments and equipment are calibrated, available and functioning with record of smear kept (See Annex 2.11.1.2b Equipment and Instrument for Blood Bank At the end of the standard)		3
2.11.1.2.5 Duty rosters	2.11.1.2.5	Duty rosters of lab are developed regularly and available in appropriate location.		1
2.11.1.2.6 Facilities for patients	2.11.1.2.6.1	Comfortable waiting space with sitting arrangement is available for at least 10 persons in waiting lobby.		1
	2.11.1.2.6.2	Safe drinking water is available in the waiting lobby throughout the day.		1
2.11.1.2.7 Recording and reporting	2.11.1.2.7.1	Sample is adequately recorded with requisition form with detail information of patients		1
	2.11.1.2.7.2	Standard reporting sheets are being used and all reports are recorded in a standard register or NBBTS software and computerized bill available to patients		1
	2.11.1.2.7.3	Report have adequate information of patient and checked by designated person before release		1
	2.11.1.2.7.3	BTSC submits regular reports to NPHL/ NBBTS of provided proficiency panels related to TTIs		1
2.11.1.2.8 Supplies storage and stock	2.11.1.2.8.1	At least three months buffer stock of laboratory supplies is available.		1
	2.11.1.2.8.2	Blood bags, transfusion sets, blood and blood components, reagents are stored at appropriate temperature in store and lab		1
2.11.1.2.9 Infection prevention	2.11.1.2.9.1	Biohazard signs and symbols are used at appropriate places		1
	2.11.1.2.9.2	All staffs know how to respond in case of spillage and other incidents		1
	2.11.1.2.9.3	Masks and gloves are available		1
	2.11.1.2.9.4	Bio-waste disposal is done based on HCWM guideline 2014 (MoHP)		1
	2.11.1.2.9.5	Hand-washing facility with running water and soap is available for practitioners		1
	2.11.1.2.9.6	Needle cutter is used		1
	2.11.1.2.9.7	Chlorine solution and bleach is available and utilized for decontamination		1
**Standard 2.11.1.2**		**Total Obtained Score**		27
		**Total Percentage (Total Obtained Score/27x100)**		

Annex 2.11.1.2a Blood Bank Furniture and Supplies

S.N.	Furniture and supplies	Required Quantity	Score
1.	Working desk with two chairs	1 set	
2.	Patient chair for blood collection	1	
3.	Blood bag single and/or component	As per need	
4.	BP cuff	1	
5.	Stethoscope	1	
6.	Weighing machine (for patient and for blood)	As per need	
7.	Band aid, cotton and spirit	As per need	
8.	Needle cutter	as per need	
9.	Reagents Kits for ABO/Rh serology/ cross-matching requirement	as per need	
10.	Glass ware for blood grouping (ABO/ Rh)	as per need	
		**Total score**	
		**Total percentage= Total Score/ 11 x 100**	

Each row gets a score of 1 if the mentioned test is available otherwise 0.

Scoring chart	
**Total Percentage**	**Score**
0-50	0
50-70	1
70-85	2
85-100	3
Score 2.11.1.2.3.1	

Annex 2.11.1.2b Equipment and Instrument for Blood Bank

S.N.	Name of Instruments	Required Quantity	Score
1.	Blood bank refrigerator 2 to 4° C	2	
2.	Ordinary centrifuge	3	
3.	Deep freezer (-20°C to -30°C)	1	
4.	Deep freezer (-80°C)	1	
5.	Platelet Shaker	1	
6.	Autoclave	1	

7.	Computer with printer	1	
8.	Gamma radiation chamber	1 (optional*)	
9.	Microscope	1	
10.	Auto pipettes (20, 50, 100 µl)	2 each	
11.	Incubator	2	
12.	Water Bath	3	
13.	Hot Air Oven	2	
14.	Generator 60 KVA	as per need	
**Total score**			
**Total percentage= Total Score/ 14 x 100**			

Each row gets a score of 1 if the mentioned test is available otherwise 0.

**Scoring chart**	
**Total Percentage**	**Score**
0-50	0
50-70	1
70-85	2
85-100	3
Score 2.11.1.2.3.3	

**Area**	**Code**	**Verification**		
**X-ray**	**2.11.2**			
**Components**		**Standards**	**Obtained Score**	**Maximum Score**
2.11.2.1 Time for patients	2.11.2.1.1	X-ray service is open from 10 AM to 3 PM		1
	2.11.2.1.2	Emergency x-ray service is available round the clock		1
2.11.2.2 Staffing	2.11.2.2	Adequate numbers of trained healthcare workers are available in x-ray (at least 2 staffs to cover shifts including ER) with on duty radiologist		1
2.11.2.3 Patient counseling	2.11.2.3	Counseling is provided to patients about radiation hazard, site and position for x-ray		1
2.11.2.4 Information education and communication materials for patients	2.11.2.4	Radiation sign, appropriate Radiation awareness posters, leaflets are available in the department and OPD waiting area.		1

2.11.2.5 Instruments and equipment	2.11.2.5	General X ray unit (with minimum 125KV and 300ma X-ray machine) with floatation table top and vertical bucky		1
	2.11.2.6	Mobile X ray unit 1 for bed side radiography for inpatient is available and functioning.		1
	2.11.2.7	Complete CR system with CR cassette at least 5 of 14 x 17 inch and 3 of 10x12inch.		1
2.11.2.8 Physical facilities	2.11.2.8.1	X ray room of at least 4x4sqm with wall of at least 23cm of brick or 6cm RCC or 2mm lead equivalent.		1
	2.11.2.8.2	Light and ventilation are adequately maintained.		1
	2.11.2.8.3	The required furniture and supplies including radiation protective measures for patients, visitors and staffs are available including lead gown		1
2.11.2.9 Duty rosters	2.11.2.9	Duty rosters of X-ray are developed regularly and available in appropriate location.		1
2.11.2.10 Facilities for patients	2.11.2.10	Waiting space with sitting arrangement is available for at least 5 persons in waiting lobby.		1
2.11.2.11 Recording and reporting	2.11.2.11.1	X-ray is adequately recorded as per requisition form with detail information of patients, date of x-ray and site and view		1
	2.11.2.11.2	Report have adequate information of patient and checked by designated person before release		1
2.11.2.12 Information to patients	2.11.2.12	Biohazard signs and symbols are used at appropriate places		1
2.11.2.12 Infection prevention	2.11.2.12.1	Radiological waste is disposed based on HCWM guideline 2014 (MoHP)		1
	2.11.2.12.2	Hand-washing facility with running water and soap is available for practitioners		1
	2.11.2.12.3	Needle cutter is used		1
	2.11.2.12.4	Chlorine solution and bleach is available and utilized for decontamination		1
**Standard 2.11.2**		**Total Obtained Score**		20
		**Total Percentage (Total Obtained Score/ 20 x100)**		

Area	Code	Verification		
**Ultrasonography (USG)**	**2.11.3**			
**Components**		**Standards**	**Obtained Score**	**Maximum Score**
2.11.3.1 Time for patients	2.11.3.1	USG is open from 10 AM to 3 PM for obstetrics, abdominal, pelvic and superficial structure like testis, thyroid		1
2.11.3.2 Staffing	2.11.3.2	USG trained medical practitioner and mid-level health worker (preferably female) in each USG room		1
2.11.3.3 Patient counseling	2.11.3.3	Counseling is provided to patients about site and indication of USG		1
2.11.3.4 Maintaining patients' privacy	2.11.3.4	Appropriate techniques have been used to ensure the patient privacy (separate rooms, curtains hung, maintaining queuing of patients)		1
2.11.3.5 Instruments and equipment	2.11.3.5	USG machine (advanced) with different probes, computer and printer with USG papers , gel and wipes is available and functional		1
2.11.3.6 Physical facilities	2.11.3.6.1	Adequate space for practitioner and patient for USG with working table and examination bed one per each USG machine		1
	2.11.3.6.2	Proper light and ventilation maintained.		1
2.11.3.7 Facilities for patients	2.11.3.7	Comfortable waiting space with sitting arrangement is available for at least 15 persons in waiting lobby.		1
2.11.3.8 Recording and reporting	2.11.3.8.1	USG is adequately recorded as per requisition form with detail information of patients, date of USG		1
	2.11.3.8.2	Report have adequate information of patient, information of area of examination and radiological opinion, further referral and checked by designated person before release		1
2.11.3.9 Infection prevention	2.11.3.9.1	Hand-washing facility with running water and soap is available for practitioners		1
	2.11.3.9.2	Chlorine solution and bleach is available and utilized for decontamination		1
Standard 2.11.3		**Total Obtained Score**		12
		**Total Percentage (Total Obtained Score/ 12 x100)**		

Area	Code	Verification		
**Electrocardiogram (ECG)**	**2.11.4**			
**Components**		**Standards**	**Obtained Score**	**Maximum Score**
2.11.4.1 Service available	2.11.4.1	ECG service is available for patients in OPD, Emergency and Indoor		1
2.11.4.2 Space	2.11.4.2	Separate space is dedicated for ECG Service		1
2.11.4.3 Patient counseling	2.11.4.3	Counseling is provided to patients about procedure and indication of ECG		1
2.11.4.4 Maintaining patient privacy	2.11.4.4	Appropriate techniques have been used to ensure the patient privacy (separate rooms, curtains hung, maintaining queuing of patients)		1
2.11.4.5 Instruments, equipment and supplies	2.11.4.5	Functional ECG machine (12 lead with power back up), paper, gel, wipes and hand sanitizer are available in ECG trolley		1
2.11.4.6 Recording and reporting	2.11.4.6.1	ECG is adequately recorded as per requisition form with detail information of patients, date of ECG		1
	2.11.4.6.2	Reporting folder of ECG should have adequate information of patient, including analysis of 12 lead ECG with final impression of ECG diagnosis done by designated person before release		1
2.11.4.7 Infection prevention	2.11.4.7.1	Hand-washing facility with running water and liquid soap is available for practitioners		1
	2.11.4.7.2	Chlorine solution and bleach is available and utilized for decontamination		1
**Standard 2.11.4**		**Total Obtained Score**		9
		**Total Percentage (Total Obtained Score/ 9 x100)**		

**Echocardiogram (Echo)**	**2.11.6**	**Verification**		
**Components**		**Standards**	**Obtained Score**	**Maximum Score**
2.11.6.1 Adequate time for patients	2.11.6.1.1	Echo service is available from 10 AM to 3 PM		1
	2.11.6.1.2	Emergency Echo is available round the clock (optional)		
2.11.6.2 Adequate health workers	2.11.6.2	Cardiologist is available for Echo service with at least one mid-level health worker assigned in Echo		1
2.11.6.3 Patient counseling	2.11.6.3	Counseling is provided to patients about procedure and indication of Echo		1
2.11.6.4 Adequate instruments, equipment and supplies	2.11.6.4	Functional Echo machine (2D, M-mode, color doppler), computer and printer with Echo papers, gel, wipes and hand sanitizer are available		1

2.11.6.5 Adequate physical facilities	2.11.6.5.1	Separate space allocated for Echo with changing room and patients' gown		1
	2.11.6.5.2	Proper light and ventilation are adequately maintained		1
	2.11.6.5.3	Echo examination bed with mattress and bed cover and pillow with curtains for privacy of patients		1
2.11.6.6 Recording and reporting	2.11.6.6.1	Echo is adequately recorded as per requisition form with detail information of patients, date of echo and echo diagnosis		1
	2.11.6.6.2	Report have adequate information of patient and checked by designated person before release		1
2.11.6.7 Infection prevention	2.11.6.7.1	Hand-washing facility with running water and soap is available for practitioners		1
	2.11.6.7.2	Chlorine solution and bleach is available and utilized		1
**Standard 2.11.6**		**Total Obtained Score**		11
		**Total Percentage (Total Obtained Score/ 11 x100)**		

**Treadmill (TMT)**	**2.11.7**	**Verification**		
**Components**		**Standards**	**Obtained Score**	**Maximum Score**
2.11.7.1 Adequate time for patients	2.11.7.1	Treadmill (TMT) service is available from 10 AM to 3 PM		1
2.11.7.2 Adequate health workers	2.11.7.2	At least one trained medical officer / cardiologist and one mid-level health worker is allocated for TMT service		1
2.11.7.3 Patient counseling	2.11.7.3	Counseling is provided to patients about procedure, indication and anticipated complication during TMT as well as to modify the routine cardiac medications as per TMT protocol.		1
2.11.7.4 Adequate instruments, equipment, medicines and supplies	2.11.7.4.1	Functional TMT machine with display monitor and printer, paper, ECG lead, wipes and hand sanitizer are available		1
	2.11.7.4.2	Emergency trolley with emergency drugs and supplies readily available (See Annex 2.11.7a Emergency Trolley TMT At the end of this standard)		3
	2.11.7.4.3	Synchronized Defibrillator is available and functional in TMT room		1
	2.11.7.4.4	Injection Dobutamine available for Stress TMT		1

2.11.7.5 Adequate physical facilities	2.11.7.5.1	Separate room allocated for TMT with changing room, gown for patient and locker for patients' belongings		1
	2.11.7.5.2	Light and ventilation are adequately maintained.		1
2.11.7.6 Recording and reporting	2.11.7.6.1	TMT is adequately recorded as per requisition form with detail information of patients, date of TMT		1
	2.11.7.6.2	TMT report should have adequate information of patient, achievement of target heart rate, blood pressure response with exercise, any ECG changes, any complication observed during exercise and recovery period and checked by designated person before release		1
2.11.7.7 Infection prevention	2.11.7.7.1	Hand-washing facility with running water and liquid soap is available for practitioners		1
	2.11.7.7.2	Chlorine solution and bleach is available and utilized for decontamination		1
**Standard 2.11.7**		**Total Obtained Score**		15
		**Total Percentage = Total Obtained Score/ 15x100**		

Annex 2.11.7a Emergency Trolley TMT

SN	Name	Required No	Score
1.	Atropine Injection	10	
2.	Adrenaline Injection	3	
3.	Xylocaine 1% and 2% Injections with Adrenaline	2	
4.	Xylocaine 1% and 2 % Injections without Adrenaline	2	
5.	Xylocaine Gel	2	
6.	Diclofenac Injection	5	
7.	Hyoscine Butylbromide Injection	5	
8.	Diazepam injection	2	
9.	Morphine Injection / Injection Pethidine	2	
10.	Hydrocortisone Injection	4	
11.	Phenaramine Injection	4	
12.	Dexamethasone Injection	4	
13.	Ranitidine/Omeperazole Injection	4	
14.	Frusemide Injection	5	

15.	Dopamine injection	2	
16.	Noradrenaline injection	2	
17.	Digoxin injection	2	
18.	Verapamil injection	2	
19.	Amidarone injection	2	
20.	Glyceryltrinitrate Injection	1	
21.	Labetolol injection	1	
22.	Sodium bicarbonate injection	2	
23.	Ceftriaxone Injection	4	
24.	Metronidazole Injection	4	
25.	Dextrose 25% / 50% Injection	2 each	
26.	IV Infusion set (Adult/Pediatric)	2	
27.	IV Canula (18, 20, 22, 24 Gz)	2 each	
28.	Disposable syringes 1 ml, 3 ml, 5 ml, 10 ml, 20 ml, 50 ml	5 each	
29.	Disposable Gloves 6, 6.5, 7, 7.5	3 each	
30.	Distilled Water	3	
31.	Needle 18-25	As per need	
32.	Sodium chloride-15%w/v and Glycerin-15% w/v (for enema)	5	
		**Total Score**	
		**Total Percentage (Total Score/ 32x100)**	

Each row gets a score of 1 if the mentioned test is available otherwise 0.

**Scoring chart**	
**Total Percentage**	**Score**
0-50	0
50-70	1
70-85	2
85-100	3
**Score for Standard 2.11.7.4.2**	

Endoscopy	2.11.8	Verification		
**Components**		**Standards**	**Obtained Score**	**Maximum Score**
2.11.8.1 Adequate time for patients	2.11.8.1. 1	Endoscopy service is available from 10 AM to 3 PM		1
	2.11.8.1. 2	Emergency endoscopy is available round the clock		1
2.11.8.2 Adequate physical facilities	2.11.8.2.1	Separate room designated for endoscopy with adjustable patient bed, head adjustable recovery bed, working chair for practitioner and working table with at least two chairs		1
	2.11.8.2.2	Scopes hanging area for storage. (covered cupboards/ shelves)		1
	2.11.8.2.3	Proper light and ventilation maintained		1
2.11.8.3 Adequate staffing	2.11.8.3.1	Physician/ surgeons having endoscopic training or Gastroenterologist or hepatologist or gastrointestinal surgeons with at least 2 Trained endoscopic nurse/paramedic designated for endoscopy room		1
	2.11.8.3.2	One mid-level trained health worker for record keeping, booking, and report dispatch , counselling before procedure		1
2.11.8.4 Patient counseling	2.11.8.4	Counseling is provided to patients about procedure and indication of Endoscopy with possible complications		1
2.11.8.5 Adequate instruments, equipment, medicines and supplies	2.11.8.5.1	Functional Video-endoscopy machine with attachable gastroscope and colonscope; bronchoscope (optional) with cardiac monitor available		1
	2.11.8.5.2	Portable Diathermy machine with cautery wires for endoscopic procedure standard.		1
	2.11.8.5.3	Emergency trolley with emergency drugs and supplies readily available (See Annex 2.11.8a Emergency Trolley Endoscopy At the end of this standard)		3
2.11.8.6 Recording and reporting	2.11.8.6.1	Recording and printing system (computer, color printer with capture card and reporting software for UGI endoscopy, colonoscopy)		1
	2.11.8.6.2	Endoscopy report is adequately recorded as per requisition form with detail information of patients, date of endoscopy and pictures attached		1
	2.11.8.6.3	Report have adequate information of patient, visibly printed pictures captured during endoscopy, endoscopic observation of structure, diagnosis and relevant endoscopic procedures performed and complications observed if any and checked by designated person before release		1

2.11.8.7 Infection prevention	2.11.8.7.1	Hand-washing facility with running water and soap is available for practitioners		1
	2.11.8.7.2	Personal protective equipment including utility gloves and boots available and used		1
	2.11.8.7.3	Separate tubs for washing, disinfection and final cleaning of the scopes available and used		1
	2.11.8.7.4	Disinfectant solution (gluteraldehyde) and Citezyme solution for enzymatic cleaning of scopes available and used		1
	2.11.8.7.5	Chlorine solution and bleach is available and utilized for decontamination (*not for scopes)		1
**Standard 2.11.8**		**Total Obtained Score**		21
		**Total Percentage (Total Obatined Score/ 21 x100)**		

Annex 2.11.8a Emergency Trolley Endoscopy

SN	Name	Required No	Score
1.	Atropine Injection	10	
2.	Adrenaline Injection	3	
3.	Xylocaine 1% and 2% Injections with Adrenaline	2	
4.	Xylocaine 1% and 2 % Injections without Adrenaline	2	
5.	Xylocaine Gel	2	
6.	Diclofenac Injection	5	
7.	Hyoscine Butylbromide Injection	5	
8.	Diazepam injection	2	
9.	Morphine Injection / Pethidine Injection	2	
10.	Hydrocortisone Injection	4	
11.	Pheramine Injection	4	
12.	Dexamethasone Injection	4	
13.	Ranitidine/Omeperazole Injection	4	
14.	Frusemide Injection	5	
15.	Dopamine injection	2	
16.	Noradrenaline injection	2	
17.	Digoxin injection	2	
18.	Verapamil injection	2	

19.	Amidarone injection	2	
20.	Glyceryltrinitrate injection	1	
21.	Labetolol injection	1	
22.	Sodium bicarbonate injection	2	
23.	Ceftriaxone Injection	4	
24.	Metronidazole Injection	4	
25.	Dextrose 25%/50% ampoule	2	
26.	IV Infusion set (Adult/Pediatric)	2	
27.	IV Canula (16, 18, 20, 22, 24, 26 Gz)	2 each	
28.	Disposable syringes 1 ml, 3 ml, 5 ml, 10 ml, 20 ml, 50 ml	5 each	
29.	Disposable Gloves (Size 6, 6.5, 7, 7.5)	3 each	
30.	Distilled Water	3	
31.	Sodium chloride-15%w/v and Glycerin-15% w/v (for enema)	As per need	
		**Total Score**	
		**Total Percentage (Total Score/ 31x100)**	

Each row gets a score of 1 if the mentioned test is available otherwise 0.

**Scoring chart**	
**Total Percentage**	**Score**
0-50	0
50-70	1
70-85	2
85-100	3
**Score for Standard 2.11.8.6.3**	

**Audiometry**	**2.11.9**	**Verification**		
**Components**		**Standards**	**Obtained Score**	**Maximum Score**
2.11.9.1 Adequate time for patients	2.11.9.1	Audiometry service is available from 10 AM to 3 PM		1
2.11.9.2 Adequate health workers	2.11.9.2	ENT specialist is available for performing audiometry		1
2.11.9.3 Patient counseling	2.11.9.3	Counseling is provided to patients about procedure and indication of audiometry and explain patient about the examination booth		1

2.11.9.4 Adequate instruments, equipment and supplies	2.11.9.4	Functional Audiometer with power supply and response switch, headphones, earphones with audiometric calibration stand and 500g weight and audiometer patch cords available		1
2.11.9.5 Adequate physical facilities	2.11.9.5.1	Separate room allocated for audiometry with a special booth made of acoustic medium is available with custom-built triangular table with the audiometer on top and the computer tower beneath and an area outside booth for working area for technologist		1
	2.11.9.5.2	Sound dampening materials on the interior walls of the exam room and a rubber seal on the hallway door		1
	2.11.9.5.3	Light and ventilation are adequately maintained.		1
2.11.9.6 Recording and reporting	2.11.9.6.1	Computer available for recording and reporting of the test results		1
	2.11.9.6.2	Audiometry is adequately recorded as per requisition form with detail information of patients and diagnosis		1
	2.11.9.6.3	Report have adequate information of patient, results of audiometry and advices for further hearing related treatment and checked by designated person before release		1
2.11.9.7 Infection prevention	2.11.9.7.1	Hand-washing facility with running water and soap is available for practitioners		1
	2.11.9.7.2	Chlorine solution and bleach is available and utilized		1
**Standard 2.11.9**		**Total Obtained Score**		12
		**Total Percentage (Total Obtained Score/ 12x100)**		

**CT Scan**	**2.11.10**	**Verification**		
**Components**		**Standards**	**Obtained Score**	**Maximum Score**
2.11.10.1 Adequate time for patients	2.11.10.1.1	CT Scan service is open from 10 AM to 3 PM and appointments date given		1
	2.11.10.1.2	Emergency CT Scan service is available round the clock		1
2.11.10.2 Adequate staffing	2.11.10.2	Adequate numbers of trained healthcare workers are available in CT scan (at least 2 staffs to cover shifts including ER) under supervision of Radiologists		1
2.11.10.3 Patient counseling	2.11.10.3	Counseling is provided to patients about radiation hazard, site and position for CT Scan and assessed for claustrophobia		1

2.11.10.4 Information education and communication materials for patients	2.11.10.4	Radiation sign, appropriate Radiation awareness posters, leaflets are available in the department and OPD waiting area.		1
2.11.10.5 Adequate instruments and equipment	2.11.10.5	CT Scan Machine (at least 128 slice) available and functional at least 1		1
2.11.10.6 Adequate physical facilities	2.11.10.6.1	CT Scan room of at least 16x20 feet with wall of at least 23cm of brick or 6cm RCC or 2mm lead equivalent and control room of 10x 12 feet		1
	2.11.10.6.2	Light and ventilation are adequately maintained with help of air conditioner and exhaust fans.		1
	2.11.10.6.3	The required furniture and supplies including radiation protective measures for patients,, visitors and staffs are available including magnetic gown		1
2.11.10.7 Duty rosters	2.11.10.7	Duty rosters for CT Scan service are developed regularly and available in appropriate location.		1
2.11.10.8 Facilities for patients	2.11.10.8.1	Comfortable waiting space with sitting arrangement is available for at least 5 persons in waiting lobby.		1
	2.11.10.8.2	Safe drinking water is available in the waiting lobby throughout the day.		1
2.11.10.9 Recording and reporting	2.11.10.9.1	CT Scan is adequately recorded as per requisition form with detail information of patients, date of Ct Scan and site and view		1
	2.11.10.9.2	Report have adequate information of patient, radiological diagnosis of CT Scan and checked by designated person before release		1
2.11.10.10 Information to patients	2.11.10.10	Biohazard signs and symbols are used at appropriate places		1
2.11.10.11 Infection prevention	2.11.10.11.1	Radiological waste is disposed based on HCWM guideline 2014 (MoHP)		1
	2.11.10.11.2	Hand-washing facility with running water and soap is available for practitioners		1
	2.11.10.11.3	Needle cutter is used		1
	2.11.10.11.4	Chlorine solution and bleach is available and utilized for decontamination		1
**Standard 2.11.10**		**Total Obtained Score**		19
		**Total Percentage (Total Obtained Score/ 19 x100)**		

Area	Code	Verification		
**Postmortem**	**2.12.1**			
**Components**		**Service Standards**	**Obtained score**	**Maximum Score**
2.12.1.1 Physical facility	2.12.1.1.2	Designated area for mortuary room, changing room and store room and bathroom		1
	2.12.1.1.2	Body dissection table (at least one) is available and used		1
	2.12.1.1.3	Organ dissection table (at least one) is available and used		1
	2.12.1.1.4	Adequate ventilation and light and odor management		1
2.12.1.2 Availability of postmortem services	2.12.1.2	Examination of the dead body in any unnatural death and suspicious death (Post-mortem examination or autopsy) available from 9 am to 5pm		1
2.12.1.3 Staffing	2.12.1.3	At least one MD forensic and one trained medical officer for autopsy and clinical medico-legal services		1
2.12.1.4 Supplies and instruments	2.12.1.4	Adequate supplies and instruments for forensic services (See Annex 2.12.1a Supplies and instrument for post mortem At the end of this standard)		3
2.12.1.5 Mortuary van	2.12.1.5	Mortuary van is available 24 hours (at least one)		1
2.12.1.6 Recording and reporting	2.12.1.6	Standardized medico-legal examination formats available		1
2.12.1.7 Infection prevention	2.12.1.7.1	Staff wear mask and gloves at work.		1
	2.12.1.7.2	There are colored bins for waste segregation and disposal based on HCWM guideline 2014 (MoHP)		1
	2.12.1.7.3	Hand-washing facility with running water and soap is available and being practiced.		1
	2.7.11.4	Chlorine solution is available and utilized.		1
	2.12.1.7.5	Proper disposal of anatomical waste in placenta pit after autoclaving		1
**Standard 2.12.1**		**Total Obtained Score**		14
		**Total Percentage (Total Obtained Score/ 19 x100)**		

Annex 2.12.1a Supplies and instrument for post mortem services

S.No.	Supplies and instrument	Required Number	Score
1.	Refrigeration chamber or cool room for body preservation	8-10 bodies capacity	
2.	Dissection set of instruments for autopsy	2 sets	
3.	Magnifying lens; 20 and 40 times	1 each	
4.	Measuring tape	2	

5.	Weighing machine for organs and if possible for dead body	1	
6.	Camera for photography	1	
7.	Glass tubes for blood collection and tissue collection; reasonable numbers for regular use	as per need	
8.	Glass slides; reasonable number for regular use	as per need	
9.	EDTA	as per need	
10.	Sodium Floride -200 or 500 gm	As per need	
11.	Formalin solution	as per need	
12.	Plastic made wide mouth containers of 500 ml capacity ; reasonable numbers for regular need	as per need	
13.	Sodium chloride (table salt); reasonable amount for regular use	as per need	
14.	Autopsy gown	2 sets	
15.	Gum boots	2 pairs	
16.	Gloves and masks	as per need	
17.	Computer with printer for report preparation	1	
18.	Cleaning agents; soap, detergents	as per need	
19.	Sealing materials; specific seal tape or wax seal and seal print	as per need	
20.	Autopsy and skeletal remains SOP, Reference Manual	as per need	
		**Total score**	
		Percentage= **Total score**/20 x 100	

Each row gets a score of 1 if the mentioned test is available otherwise 0.

Scoring	
**Total Percentage**	**Score**
0-50	0
50-70	1
70-85	2
85-100	3
**Score for Standard 2.12.1.6.1**	

Area	Code	Verification		
**Medico-legal services**	**2.12.2**			
**Components**		**Standards**	**Obtained score**	**Maximum Score**
2.12.2.1 Physical facility	2.12.2.1	Designated area for medico-legal examination with examination bed and working desk with chair		1
2.12.2.2 Availability of medico-legal services	2.12.2.2.1	Medico-legal services are available 24 hours		1
	2.12.2.2.2	Injury examination and reporting in cases of physical assaults, attempted murder, sexual offenses (victim and accused), metal status examination, torture victim examination and drunkenness examination and at least one examination bed allocated for it		1
2.12.2.3 Staffing	2.12.2.3	At least one MD forensic and one trained medical officer for autopsy and clinical medico-legal services		1
2.12.2.4 Supplies and instruments	2.12.2.4.1	Adequate supplies and instruments for medico-legal services (See Annex 2.12.2a Supplies and instrument for medico legal services At the end of this standard)		3
	2.12.2.4.2	Preservation of sample ensured before dispatching for test		1
2.12.2.5 Patient counseling	2.12.2.5	Post-traumatic counseling is done to the victims of medico-legal issues like sexual offence		1
2.12.2.6 Recording and reporting	2.12.2.6	Standardized medico-legal examination formats available		1
2.12.2.7 Infection prevention	2.12.2.7.1	Staff wear mask and gloves at work.		1
	2.12.2.7.2	There are well labelled colored bins for waste segregation and disposal based on HCWM guideline 2014 (MoHP)		1
	2.12.2.7.3	Hand-washing facility with running water and soap is available and being practiced.		1
	2.12.2.7.4	Chlorine solution is available and utilized.		1
**Standard 2.12.2**		**Total Obtained Score**		14
		**Total Percentage (Total Obtained Score/ 14 x100)**		

Annex 2.12.2a Supplies and instrument for clinical medico-legal services

S.No.	Supplies and instrument	Required number	Score
1.	Weight machine and height scale	1 each	
2.	BP set, stethoscope and torch light	1 each	
3.	Examination kits; sexual offence cases (rape victim examination kit)	as per need	

4.	Gloves and masks	as per need	
5.	Magnifying lens; 20 and 40 times	1 each	
6.	Measuring tape	As per need	
7.	Camera for photography	1	
8.	Paper envelopes of different sizes for collection of samples and packing	as per need	
9.	Glass tubes for collection of blood urine; reasonable number for regular use	as per need	
10.	X ray plate view box	1	
11.	EDTA and Sodium floride 500 gm	As per need	
12.	Glass slides; reasonable number for regular use	as per need	
13.	Cupboards for store and necessary other furniture for examination room	as per need	
14.	Sealing materials as for autopsy room	as per need	
15.	Computer and printer for report preparation as in autopsy	1	
16.	SOPs and Reference Manuals for age estimation, sexual offence case examination, injury examination, drunkenness examination, mental state examination and torture victim examination.	1	
		**Total score**	
		Percentage= **Total score**/16 x 100	

Each row gets a score of 1 if the mentioned test is available otherwise 0.

Scoring	
**Total Percentage**	**Score**
0-50	0
50-70	1
70-85	2
85-100	3
**Score for Standard 2.12.2.6.2**	

Area	Code	Verification		
**One stop crisis management center**	**2.13**			
**Components**		**Service Standards**	**Obtained Score**	**Maximum Score**
2.13.1 OCMC coordination committee exists (multi-sectoral)	2.13.1.	Quarterly meeting minute of coordination committee		1
2.13.2 Functionality of case management committee	2.13.2	Monthly meeting minute of case management committee		1
2.13.3 Timely service for patients	2.13.3.1	Prioritized services for GBV victims/survivors exits		1
	2.13.3.2	Treatment for GBV survivors/affected by GBV is available 24 hours		1
2.13.4 Physical facilities for OCMC services	2.13.4	Separate space allocated for OCMC services with adequate physical facilities (See Annex 2.13a Physical facilities for OCMC At the end of this standard)		3
2.13.5 Staffing	2.13.5.1	At least one Medical officer working in the hospital trained in medico-legal issues is available		1
	2.13.5.2	At least two Staff nurse working in the hospital and 1 trained psycho social counselor		1
2.13.6 Timely examination from medico-legal aspects and treatment of GBV survivors/ affected by GBV	2.13.6.1	Health check-up, medico-legal examination including documentation (See Annex2.13b Instruments and supplies for treatment in OCMC At the end of this standard)		3
	2.13.6.2	Preservation of samples as legal evidence done for future use (See Annex 2.13.3c Instruments and supplies for evidence collection in OCMC At the end of this standard)		3
	2.13.6.3	Pregnancy test and emergency contraceptive services, tests for HIV/HBV available		1
2.13.7 Use of GBV clinical protocol	2.13.7.1	Whole site orientation on GBV clinical protocol conducted		1
	2.13.7.2	Availability and use of the treatment as per the protocol and OCMC guideline		1
2.13.8 Psycho-social counselling of GBV Survivors/ affected by GBV and rehabilitation	2.13.8.1	Mental health and psychosocial counselling services available		1
	2.13.8.2	If the female survivor requires to stay more days or requires advance psychosocial counseling including livelihood training, she/ he shall be referred to nearby appropriate safe home/ rehabilitation centers		1
2.13.9 Referral services in place	2.13.9.1	Provide required referral and other services (as per the health service guideline and protocol). (Beyond health: security, legal, shelter, rehabilitation)		1
	2.13.9.2	Health related referral services e.g. Safe abortion services		1

2.13.10 Information, education and empowerment for GBV survivors/ affected by GBV	2.13.10.1	Detailed information concerning the services being provided by OCMC to the survivors of GBV (Citizen charter, leaflets, community radio etc)		1
	2.13.10.2	Information is being given in an integrated manner (Safe home related, OCMC, Police women children service unit)		1
2.13.11Recording and reporting	2.13.11.1	Details of the events registered in the OCMC, services (health and non-health) being provided to the survivors, listing of the referred organizations shall be documented to be classified and analyzed in due course.		1
	2.13.11.2	Confidentiality shall be maintained at all stages of documentation.		1
	2.13.11.3	Report to concerned authority (DCC and MoHP) in monthly report service format		1
	2.13.11.4	Documentation of the current status of GBV survivors of at least last 1 year is done		1
2.13.12 Infection prevention	2.13.12.1	Masks and gloves are available and used		1
	2.13.12.2	There are colored bins for waste segregation and disposal based on HCWM guideline 2014 (MoHP)		1
	2.13.12.3	Hand-washing facility with running water and soap is available for practitioners.		1
	2.13.12.4	Needle cutter is used		1
	2.13.12.5	Chlorine solution is available and utilized.		1
**Standard 2.13**		**Total Obtained Score**		29
		**Total Percentage (Total Obtained Score/ 29 x100)**		

Annex 2.13a Physical Facilities for OCMC

SN	General Items	Required No.	Score
1	Rooms for treatment room/examination room, office and guard room	1 each	
2	Toilet allocated for OCMC services	as per need	
3	Curtains to maintain confidentiality during the forensic examination	as per need	
4	Examination table	1	
5	Desk	1	
6	Chairs	3	
7	Cupboard to keep clients' information with filing cabinet	1	
8	Movable table lamp	1	
9	toilet and bathroom for clients' use (water, bucket, mug, soap, towel)	as per need	
10	Hand washing facility for service provider	as per need	

11	Refrigerator and lockable cupboard for specimen store	1	
12	Telephone	1	
13	Computer and printer	1set	
14	Boiler (for tea)	1 set	
**Total score**			
Percentage= **Total score**/14 x 100			

Each row gets a score of 1 if the mentioned test is available otherwise 0l

Scoring	
**Total Percentage**	**Score**
0-50	0
50-70	1
70-85	2
85-100	3
**Score for Standard 2.13.4**	

Annex 2.13b Instruments and supplies for treatment in OCMC

SN	General Items	Required No.	Score
1	Sphygmomanometer (B.P. Instrument)	1	
2	Stethoscope	1	
3	Torch Light	1	
4	Tongue Depressor	as per need	
5	Tourniquet	1	
6	Sterilized Gloves as required	as per need	
7	Sterilized Syringe and Needles as required	as per need	
8	Cotton and Bandage as required	as per need	
9	Sterilized Vial for sample collection	as per need	
10	Different sized Reflecting Mirrors (big, medium and small)	1 each	
11	Sterilized Speculum	1	
12	Glutaradehylde solution for high level of infection prevention	as per need	
13	Chlorine powder to sterilize the used materials/tools	as per need	

14	Protoscope /Anscope	1	
15	Pregnancy Test Kit	as per need	
16	Specimen collection materials for communicable Sexually Transmitted Infections	as per need	
17	Lubricant, Clean Water, Normal Saline	as per need	
18	Tray for sharp instruments, such as scissors, knife etc.	as per need	
19	Height Measuring Scale	1	
20	Weight Measuring Scale	1	
		**Total score**	
		**Total Percentage = Total Score/ 20 x 100**	

Each row gets a score of 1 if the mentioned test is available otherwise 0.

**Scoring**	
**Total Percentage**	**Score**
0-50	0
50-70	1
70-85	2
85-100	3
**Score for Standard 2.13.6.1**	

Annex 2.13c Instruments and supplies for evidence collection in OCMC

SN	General Items	Required No.	Score
1	Cotton/material to collect sperm, Blood, Saliva etc. from survivor	as per need	
2	Container/vessel to keep the collected specimen	as per need	
3	Materials to swab	as per need	
4	Microscope slide	as per need	
5	Vials for blood collection	as per need	
6	Vials to collect urine for pregnancy test	as per need	
7	Paper or plastic seat	as per need	
8	Paper bag to hold clothes and other items	as per need	
9	Air spatula and slide for pap smear	as per need	
10	Fixing solutions: hair spray, alcohol etc	as per need	
11	Analgesic : Normal medications like Paracetamol, Ibuprofen etc. for pain relief	as per need	

12	Emergency Contraceptives: Pills and IUCD	as per need	
13	Thread for Suturing	as per need	
14	Immunization for Tetanus and Hepatitis	as per need	
15	STI Preventive	as per need	
16	Bed Sheet and Blankets for examination table	as per need	
17	Towel	as per need	
18	Clothes for Survivor (if her clothes are torn or stained).	as per need	
19	Gown to be worn during the examination	as per need	
20	Sanitary Pads and Tampons for internal use	as per need	
21	Documentation forms and recording forms	as per need	
22	Camera and Film for evidence collection	1	
24	Colposcope or Magnifying Glass	1	
		**Total score**	
		**Total Percentage = Total Score/ 24 x 100**	

Each row gets a score of 1 if the mentioned test is available otherwise 0.

**Scoring**	
**Total Percentage**	**Score**
0-50	0
50-70	1
70-85	2
85-100	3
**Score for Standard 2.13.6.2**	

**Area**	**Code**	**Verification**		
**Physiotherapy (Physical Rehabilitation)**	**2.14**			
**Components**		**Standards**	**Obtained Score**	**Maximum Score**
2.14.1 Space	2.14.1	Separate room for OPD physiotherapy with at least 15 physiotherapy beds with 8 exercise beds and 7 electric beds		1
2.14.2Time for patients	2.14.2.1	Physiotherapy OPD is open from 10 AM to 5 PM.		1
	2.14.2.2	Inpatient physiotherapy service is available based on the requisition		1

2.14.3 Staffing	2.14.3	At least 3 physiotherapist trained in Masters in Physiotherapy (MPT) (one orthopedics, one cardiac and one neurology specialty), 6 trained in Bachelors in Physiotherapy (BPT),and 3 Certificate in physiotherapy (CPT) or Diploma in physiotherapy (DPT) and 1 trained office assistant treating at least 40 patients per day on OPD basis		1
	2.14.3.1	Duty roster prepared for a month to cover 24 hours service		1
2.14.4 Maintaining patient privacy	2.14.4	Appropriate techniques have been used to ensure the patient privacy (separate rooms, curtains hung, maintaining queuing of patients).		1
2.14.5 Patient counseling	2.14.5	Counseling is provided to patients about the type of treatment being given and its consequences.		1
2.14.6 IEC/BCC materials	2.14.6	Appropriate IEC/BCC materials (posters, leaflets etc.) are available in the OPD waiting area.		1
2.14.7 Instruments and equipment	2.14.7	Instruments and equipment to carry out the Physiotherapy works are available and functioning (See Annex 2.14a Instruments and equipment physiotherapy At the end of this standard).		3
2.14.8 Physical facilities	2.14.8.1	Adequate rooms and space for the practitioners and patients are available.		1
	2.14.8.2	Light and ventilation are adequately maintained.		1
2.14.9 Duty rosters	2.14.9	Duty rosters of OPD are developed regularly and available in appropriate location.		1
2.14.10 Facilities for patients	2.14.10.1	Safe drinking water is available in the waiting lobby throughout the day.		1
	2.14.10.2	Hand-washing facilities are available for patients.		1
2.14.11 Recording and reporting	2.14.11.1	Recording and reporting throughout treatment and follow up is done		1
2.14.12 Infection prevention	2.14.12.1	Masks and gloves are available and used		1
	2.14.12.2	There are colored bins for waste segregation and disposal based on HCWM guideline 2014 (MoHP)		1
	2.14.12.3	Hand-washing facility with running water and soap is available for practitioners.		1
	2.14.12.4	Needle cutter is used		1
	2.14.12.5	Chlorine solution is available and utilized.		1
**Standard 2.14**		**Total Obtained Score**		**22**
		**Percentage = Total Obtained Score / 22 x 100**		

Annex 2.14a Instruments and equipment physiotherapy

SN	Instruments and equipment	Required No.	Score
1.	Traction unit	2	
2.	IFT(Interferential treatment)	4	
3.	Ultrasound(treatment) unit	4	
4.	TENS (Transcutaneous nerve stimulation)	4	
5.	Muscle stimulator	3	
6.	Parallel bar	1	
7.	Quadriceps Table	1	
8.	Therabands	5	
9.	Heel exerciser	1	
10.	CPM machine knee and elbow	1	
11.	Physio ball 55" 65" and 90"	3	
12.	Moist heat unit	1	
13.	Wax unit	1	
14.	Foot step	1	
15.	Shoulder wheel	2	
16.	Pulley Set	2	
17.	Static Cycle	1	
18.	Weight Cuffs set	2	
19.	Dumbell set	2	
20.	Shortwave diathermy	1	
21.	Tit table (electronic)	3	
22.	Microwave diathermy	1	
23.	Cryotherapy unit	1	
24.	Hand exercise table	1	
25.	Mobilization table / bed	2	
26.	Laser therapy unit	1	
27.	Shockwave Therapy	1	
28.	Ergometer	1	
29.	Multi-gym exercise set	1	
		**Total Score**	
		**Total Percentage= Total Score/29 x100**	

Each row gets a score of 1 if the mentioned test is available otherwise 0.

Scoring	
**Total Percentage**	**Score**
0-50	0
50-70	1
70-85	2
85-100	3
**Score for Standard 2.14.7**	

**Dietetics and Nutrition rehabilitation**	**2.15**	**Verification**		
**Components**		**Standards**	**Obtained Score**	**Maximum Score**
2.15.1 Adequate space	2.15.1	Separate space allocated for Dietetics and Nutrition rehabilitation		1
2.15.2 Adequate time and access for patients	2.15.2.1	Dietetics and Nutrition rehabilitation unit opens from 10 am to 3 pm		1
	2.15.2.2	Inpatients monitored for nutritional needs, rehabilitation and therapeutic diets prescribed where needed		1
2.15.3 Adequate health workers	2.15.3	1 Senior dietitian (Masters in Nutrition & Dietetics with hospital internship, or Bachelors in Nutrition & Dietetics with one year's hospital experience), 1 dietetic assistant and 1 mid-level health workers trained in nutrition rehabilitation available for the dietetics and nutrition rehabilitation service		1
2.15.4 Maintaining patient privacy	2.15.4	Appropriate techniques have been used to ensure the patient privacy (separate rooms, curtains hung, maintaining queuing of patients, etc).		1
2.15.5 Patient counseling	2.15.5	Counseling is provided to patients about their nutritional status, diet prescription/ nutritional rehabilitation, use of local food as sources of the diet required and follow up		1
2.15.6 Information education and communication materials for patients	2.15.6	Appropriate IEC materials (posters, leaflets etc.) are available in waiting area on balanced diet and identification of malnutrition specially among children		1
2.15.7 Adequate instruments and equipment	2.15.7	Instruments and equipment to carry out the OPD works are available and functioning		1

2.15.8 Inpatient stabilization center				
2.15.8.1 Adequate space	2.15.8.1	Separate space assigned for inpatient nutrition stabilization		1
2.15.8.2 Adequate staffing	2.15.8.2	Trained staffs assigned for inpatient nutrition stabilization		1
2.15.8.3 Adequate instrument, equipment, supplies	2.15.8.3	Instruments, equipment and supplies needed for inpatient stabilization center available and functional		1
2.15.8.4 Adequate physical facilities	2.15.8.4	Adequate rooms and space for the practitioners and patients are available.		1
	2.15.8.5	Light and ventilation are adequately maintained.		1
	2.15.8.6	The required furniture are available		1
2.15.9 Recording and reporting	2.15.9	Treatment, follow up and progress are recorded and reported		1
2.15.10 Facilities for patients	2.15.10.1	Safe drinking water is available in the waiting lobby throughout the day.		1
	2.15.10.2	Hand-washing facilities are available for patients.		1
2.15.11 Infection prevention	2.15.11.1	Masks and gloves are available.		1
	2.15.11.2	There are colored bins for waste segregation and disposal as per HCWM guideline 2014 (MoHP)		1
	2.15.11.3	Hand-washing facility with running water and soap is available for practitioners.		1
	2.15.11.4	Needle cutter is used		1
	2.15.11.5	Chlorine solution is available and utilized.		1
**Standard 2.15**		**Total Obtained Score**		**22**
		**Total Percentage (Total Obtained Score/ 22 x100)**		

Area	Code	Verification		
**Cardiac catheterization Laboratory**	2.16			
**Components**		**Standards**	**Obtained Score**	**Maximum Score**
2.16.1 Time for Cardiac catheterization Laboratory (Cath Lab)	2.161.1	Routine procedures available on scheduled days		1
	2.16.1.2	Emergency procedures available round the clock		1
	2.16.1.3	At least two functional operating tables		1

2.16.2 Staffing	2.16.2	For one cardiac intervention, at least a team is composed of: MD Internal Medicine trained in cardiac intervention or cardiologist with one trained medical officer, two trained nursing/paramedics, and one trained office assistant		1
2.16.3 Cath Lab services available	2.16.3	Cath lab has at least following services available		
	2.16.3.1	Coronary Angiography		1
	2.16.3.2	Percutaneous transluminal coronary angioplasty/ percutaneous coronary intervention(PTCA)		1
	2.16.3.3	Right heart catheterization		1
	2.16.3.4	Pigtail insertion (Pericardiocentesis)		1
2.16.4 Patient counseling	2.16.4.1	Indications and reviews the clinical history and physical examination is documented		1
	2.16.4.2	Informed consent is taken before intervention; patients and caretakers are given appropriate counseling about the procedure.		1
2.16.5 Patient preparation	2.16.5	Preoperative instructions for patient preparation are given and practiced with routine pre-anesthesia check up		1
2.16.6 Post-procedure care	2.16.6.1	Separate area designated for post-procedure care to stabilize the patient after procedure		1
	2.16.6.2	Staffs are specified for the post-procedure care including close monitoring of the vital signs and observation of patient for bleeding in intervention site		1
	2.16.6.3	Adequate information shared for patient care and patient followed by at least one mid-level health worker for hand over or transfer of patient within or outside the hospital		1
2.16.7 Cath Lab Set Up	2.16.7.1	Cath Lab has appropriate physical set up (See Annex 2.16a Physical Set Up for Cath Lab At the end of this standard)		3
	2.16.7.2	General equipment, instruments and supplies available (See Annex 2.16b Furniture, Equipment, Instruments and Supplies for Cath Lab At the end of this standard)		3
	2.16.7.3	Medicines and supplies available (See Annex 2.16c General Medicine and Supplies for Cath lab At the end of this standard)		3
	2.16.7.4	Surgical sets for minimum list of the interventions available (See Annex 2.16d Surgical sets for Minimum list of Interventions At the end of this standard)		3
2.16.8 Safe Surgery Checklist	2.16.8	The Safe Surgery Checklist is available in Cath Lab and used		1
2.16.9 Records	2.16.9	Recording is done for all surgeries including procedure, observation during surgical procedure and complications if any		1
2.16.10 Infection prevention protocol is strictly followed by all staffs in Cath Lab				

2.16.10.1 Hand hygiene	2.16.10.1	Hand-washing and scrubbing facility with running water and soap is available and being practiced with elbow tap		1
2.16.10.2 Appropriate PPE	2.16.10.2	Gowns and sterile drapes for patients and sterile gown for surgery team are available and used whenever required.		1
2.16.10.3 Fumigation	2.16.10.3	Fumigation is done at least once a week in the Cath Lab		1
2.16.10.4 Disinfection of instruments	2.16.10.4	High Level Disinfection (e.g. Cidex) facility is available and being practiced.		1
2.16.10.5 High Dusting	2.16.10.5	High dusting is done at least twice a week in Cath Lab		1
2.16.10.6 Appropriate segregation of waste	2.16.10.6	Separate colored waste bins based on HCWM guideline 2014 (MoHP) are available and used		1
2.16.10.7 Disposal of sharps	2.16.10.7	Needle cutter is used		1
2.16.10.8 Cleaning	2.16.10.8	Chlorine solution is available and utilized for decontamination.		1
**Standard 2.16**		**Total Obtained Score**		36
		**Total Percentage= Total Obtained Score/ 36 x 100**		

Annex 2.16a Physical Set Up for Cath Lab

SN	Physical Set Up	Score		
1.	Separate room designated for Cath Lab with recovery room and control room			
2.	Space designated for changing room for male and female staffs separately			
3.	Lockers for storage of the belongings of staffs			
4.	Separate shelves for storage of clean and dirty shoes at the entrance of the Cath Lab area demarked with red line			
5.	Space designated with sink facilitated with elbow tap for scrubbing			
6.	Designated space for tea room			
7.	Separate bathroom with at least one universal toilet for Cath Lab			
8.	Scrub basins with running water			
9.	Utility basins (at least 4)			
	**Total Score**			
	**Total percentage= Total Score/ 9 x 100**			

Each row gets a score of 1 if the mentioned test is available otherwise 0.

Scoring chart	
**Total percentage**	**Score**
0-50	0
50-70	1
70-85	2
85-100	3
**Score for Standard 2.16.7.1**	

Annex 2.16b Furniture, Equipment, Instruments and Supplies for Cath Lab

SN	General Equipment and Instruments for OT	Standard Quantity	Score
1.	Wheel chair foldable, adult size	1	
2.	Stretcher	1	
3.	Patient trolley	1	
4.	Cupboards and cabinets for store	1	
5.	Working desk for anesthesia, nursing station, gowning	1 each	
6.	OT Table- universal type/ with wedge to position patient	At least 2	
7.	Flurosope	At least 1	
8.	Cardiac Monitors	At least 2	
9.	Computer with display monitor and printer in control room	At least 1 set	
10.	Examining table	1	
11.	Mayo Stand with tray	2	
12.	Operation theatre lights	1	
13.	Ultra violet light source	1	
14.	Electronic suction machine/ Foot-operated suction machine	1/1	
15.	Refrigerator / cold box	1	
16.	Boiler/ Autoclave	1/1	
17.	Oxygen concentrator/ Oxygen Cylinder/ Central oxygen	1/1/ available	
18.	Instrument trolley	2	

19.	BP instrument with stethoscope	1	
20.	Thermometer	1	
21.	Steel Drum for gloves	1	
22.	Steel Drum for Cotton	1	
23.	Tourniquet, latex rubber, 75 cm	2	
24.	Kidney tray (600cc)	2	
25.	Covered instrument trays	4	
26.	Mackintosh sheet	1	
27.	Lead gown	2 sets	
28.	Bowl stand	2	
29.	Cheatle forceps in jar	2	
30.	Packing towel double wrapper	As per need	
31.	Sterile gloves (6,6.5,7,7.5,8)	5 each	
32.	Towels/ eye hole	As per need	
33.	Masks and caps	As per need	
34.	Torch light and batteries	1 set	
35.	Foot steps	2	
36.	Wall clock	1	
37.	Waste bucket for scrub nurse	1	
38.	IV stand	2	
39.	Big tray, Big bowl, small bowl, kidney tray, sponge holder	2 each	
40.	Lap Pack	At least 2	
41.	IV set and PMO line	At least 10 each	
42.	Leak proof sharp container	1	
43.	Color coded waste bins (based on HCWM guideline2014 (MoHP))	1 set per OT	
44.	Disposable syringes of different size, 3 way connector	As per need	
		**Total Score**	
		**Total percentage= Total/ 44 x 100**	

Each row gets a score of 1 if the mentioned test is available otherwise 0.

Scoring Chart	
**Total percentage**	**Score**
0-50	0
50-70	1
70-85	2
85-100	3
**Score for Standard 2.16.7.2**	

Annex 2.16c Medicine and Supplies for Cath Lab

SN	Emergency Drugs (including neonates) for OT	Standard Quantity for 1 patient	Score
1.	Verapamil Injection	4 ampules	
2.	Heparin Injection	2 vials	
3.	Midazolam Injection	5 vials	
4.	Hydrocortisone Powder for Injection	100ml 2 vial	
5.	Frusemide Injection	2 ampules	
6.	Dopamine Injection	5 vials	
7.	Ergometrine Injection	2 ampules	
8.	Hydralizine injection	5 vials	
9.	Calcium Gluconate Injection	10ml X 2 ampules	
10.	Dextrose (25%/50%) Injection	2 ampules each	
11.	Naloxone Injection	1 ampule	
12.	Aminophyline Injection	2 ampules	
13.	Chloropheniramine Injection	2 ampules	
14.	Mephentine Injection	1 vial	
15.	IV Fluids- Ringer Lactate / Normal Saline/ DNS/ D5%	6 bottles each	
16.	IV infusion Set	4	
17.	IV Canula 22G/20G/18G	4 each	
		**Total Score**	
		**Total Percentage = Total Score/17 X 100**	

Each row gets a score of 1 if all the required number is available otherwise 0

Scoring Chart	
**Total percentage**	**Score**
0-50	0
50-70	1
70-85	2
85-100	3
**Score for Standard 2.16.7.3**	

Annex 2.16d Minimum List of Surgical Sets for Intervention

S.No.	Items	Required number	Score
1.	Catheterization set	At least 5	
2.	Coronary angiography set	At least 2	
3.	PTCA kit	At least 2	
4.	TPI Set	At least 2	
5.	Swan Ganz Catheter	At least 2	
6.	Teurmo Wire J tip	At least 5	
7.	Radial/ femoral sheath 5 Fr	At least 10 each	
8.	Port manifold	At least 10	
9.	Ordinary wire	At least 5	
10.	Mersilk (2-0) cutting body	As per need	
11.	Pigtail 5/6 Fr	At least 5	
		**Total Score**	
		**Total Percentage = Total Score/11 x 100**	

Each row gets a score of 1 if all the required number is available otherwise 0.

Scoring Chart	
**Total Percentage**	**Score**
0-50	0
50-70	1
70-85	2
85-100	3
**Score for Standard 2.16.7.4**	

Area	Code	Verification		
**Diagnostics and laboratory**	**2.11**			
**Laboratory**	**2.11.1.1**			
**Components**		**Standards**	**Obtained Score**	**Maximum Score**
2.11.1.1.1Time for patients	2.11.1.1.1.1	Laboratory is open from 10 AM to 3 PM for routine services and separate emergency lab service available round the clock		1
	2.11.1.1.1.2	Basic investigations are available (See Annex 2.11.1a List of investigations for Laboratory At the end of this standard)		3
2.11.1.1.2 Staffing	2.11.1.1.2.1	Laboratory team is lead by pathologist (at least) 3 (one for histocytopathology and hematology, 1 for biochemistry and 1 for microbiology)		1
	2.11.1.2.2	At least 12 lab staffs (4 medical technologists, 4technicians, 2 assistants and 2 helpers are available during routine lab		1
	2.11.1.1.2.3	At least 3 staffs (1 Technician, 1 Assistant and 1 Helper) in each shift in emergency lab		1
	2.11.1.1.2.4	On call biomedical engineer available for maintenance of lab equipment		1
2.11.1.1.3 Instruments and equipment	2.11.1.1.3.1	Instruments and equipment to carry out all parameters of tests are available and functioning(See Annex 2.11.1.1b Equipment and Instrument for Lab At the end of this standard)		3
	2.11.1.1.3.2	Instrument are maintained and calibrated as per manufacturer instructions		1
	2.11.1.3.3	Quality control sera and standards are run regularly and record kept		1
2.11.1.1.4 Physical facilities	2.11.1.1.4.1	Separate space with working desk and chair designated for specific laboratory procedures like- hematology, biochemistry, microbiology, serology, histopathology and cytology		1
	2.11.1.1.4.2	Light and ventilation are adequately maintained.		1
	2.11.1.1.4.3	Designated area well labelled for reception of sample and dispatch of reports		1
	2.11.1.1.4.4	Power back up is available for the lab for preservation of sample and regents		1
2.11.1.1.5 Duty rosters	2.11.1.1.5	Duty rosters of lab are developed regularly and available in appropriate location.		1

2.11.1.1.6 Facilities for patients	2.11.1.1.6.1	Comfortable waiting space with sitting arrangement is available for at least 15 persons in waiting lobby.		1
	2.11.1.1.6.2	At least one each male, female and universal toilet for patients using laboratory services		1
	2.11.1.1.6.3	Safe drinking water is available in the waiting lobby throughout the day.		1
2.11.1.1.7 Recording and reporting	2.11.1.1.7.1	Laboratory Information System (LIS) available		1
	2.11.1.1.7.2	Major instruments are interfaced		1
	2.11.1.1.7.3	Sample is adequately recorded with requisition form with detail information of patients		1
	2.11.1.1.7.4	Standard reporting sheets are being used and all reports are recorded in a standard register (HMIS 9.4).		1
	2.11.1.1.7.5	Copy of computerized report is kept safe (hard and soft copies) for future use till 6 months and report is available to patient		1
	2.11.1.1.7.6	Report have adequate information of patient and checked by designated person before release		1
2.11.1.1.8 Supplies storage and stock	2.11.1.1.8.1	At least three months buffer stock of laboratory supplies is available.		1
	2.11.1.1.8.2	Reagents are stored at appropriate temperature in store and lab		1
2.11.1.1.9 Blood Bank within hospital premises	2.11.1.1.9	Blood bank should be available inside hospital premises either owned by hospital or Nepal Red Cross Society (If hospital has its own blood bank refer to standard 2.11.1.2)		1
2.11.1.1.10 Infection prevention	2.11.1.1.10.1	Closed vacuum system is used for sample collection		1
	2.11.1.1.10.2	Biohazard signs and symbols are used at appropriate places visibly		1
	2.11.1.1.10.3	All staffs know how to respond in case of spillage and other incidents		1
	2.11.1.1.10.4	Masks and gloves are available		1
	2.11.1.1.10.5	There are colored bins for waste segregation based on HCWM guideline 2014 (MoHP) and infectious waste is sterilized using autoclave before disposal		1
	2.11.1.1.10.6	Hand-washing facility with running water and soap is available for practitioners		1
	2.11.1.1.10.7	Needle cutter is used		1
	2.11.1.1.10.8	Chlorine solution and bleach is available and utilized for decontamination		1
**Standard 2.11.1.1**		**Total Score**		31
		**Total Percentage (Total Score/ 31x100)**		

Annex 2.11.1.1a List of Investigations for Laboratory

Each row gets a score of 1 if all the required number is available otherwise 0.

Scoring chart	
**Total percentage**	**Score**
0-50	0
50-70	1
70-85	2
85-100	3
**Score for Standard 2.11.1.1.1.2**	

Area	Code	Verification (*Applicable if hospital has its own blood bank)		
**Blood Bank***	**2.11.1.2**			
**Components**		**Service Standards**	**Obtained Score**	**Maximum Score**
2.11.1.2.1 Time for patients	2.11.1.2.1	Blood bank is open / facility is available round the clock		1
2.11.1.2.2 Staffing	2.11.1.2.2	Adequate numbers of trained healthcare workers are available in blood bank (at least 2 blood bank staffs to cover shifts including ER)		1
2.11.1.2.3 Physical facilities	2.11.1.2.3.1	Adequate rooms and space for the staffs and patients are available (area of more than 10 meter squares)		1
	2.11.1.2.3.2	Light and ventilation are adequately maintained.		1
	2.11.1.2.3.3	The required furniture and supplies are available (See Annex Blood Bank Furniture and Supplies At the end of this standard)		3
	2.11.1.2.3.4	Thermometers are attached to all equipment requiring temperature control and temperatures are recorded daily or temperature sensor based equipment		1
2.11.1.2.4 Instruments and equipment	2.11.1.2.4	Instruments and equipment are calibrated, available and functioning with record of smear kept (See Annex Equipment and Instrument for Blood Bank At the end of this standard)		3
2.11.1.2.5 Duty rosters	2.11.1.2.5	Duty rosters of lab are developed regularly and available in appropriate location.		1
2.11.1.2.6 Facilities for patients	2.11.1.2.6.1	Comfortable waiting space with sitting arrangement is available for at least 10 persons in waiting lobby.		1
	2.11.1.2.6.2	Safe drinking water is available in the waiting lobby throughout the day.		1

2.11.1.2.7 Recording and reporting	2.11.1.2.7.1	Sample is adequately recorded with requisition form with detail information of patients		1
	2.11.1.2.7.2	Standard reporting sheets are being used and all reports are recorded in a standard register or NBBTS software and computerized bill available to patients		1
	2.11.1.2.7.3	Report have adequate information of patient and checked by designated person before release		1
	2.11.1.2.7.3	BTSC submits regular reports to NPHL/ NBBTS of provided proficiency panels related to TTIs		1
2.11.1.2.8 Supplies storage and stock	2.11.1.2.8.1	At least three months buffer stock of laboratory supplies is available.		1
	2.11.1.2.8.2	Blood bags, transfusion sets, blood and blood components, reagents are stored at appropriate temperature in store and lab		1
2.11.1.2.9 Infection prevention	2.11.1.2.9.1	Biohazard signs and symbols are used at appropriate places		1
	2.11.1.2.9.2	All staffs know how to respond in case of spillage and other incidents		1
	2.11.1.2.9.3	Masks and gloves are available		1
	2.11.1.2.9.4	Bio-waste disposal is done based on HCWM guideline 2014 (MoHP)		1
	2.11.1.2.9.5	Hand-washing facility with running water and soap is available for practitioners		1
	2.11.1.2.9.6	Needle cutter is used		1
	2.11.1.2.9.7	Chlorine solution and bleach is available and utilized for decontamination		1
**Standard 2.11.1.2**		**Total Score**		27
		**Total Percentage (Total Score/27x100)**		

Annex 2.11.1.2a Blood Bank Furniture and Supplies

S.N.	Furniture and supplies	Required Quantity	Score
1.	Working desk with two chairs	1 set	
2.	Patient chair for blood collection	1	
3.	Blood bag single and/or component	As per need	
4.	BP cuff	1	
5.	Stethoscope	1	
6.	Weighing machine (for patient and for blood)	As per need	
7.	Band aid, cotton and spirit	As per need	
8.	Needle cutter	as per need	
9.	Reagents Kits for ABO/Rh serology/ cross-matching requirement	as per need	
10.	Glass ware for blood grouping (ABO/ Rh)	as per need	
**Total score**			
**Total percentage= Total Score/ 11 x 100**			

Each row gets a score of 1 if the mentioned test is available otherwise 0.

Scoring chart	
**Total Percentage**	**Score**
0-50	0
50-70	1
70-85	2
85-100	3
Score 2.11.1.2.3.1	

Annex 2.11.1.2b Equipment and Instrument for Blood Bank

S.N.	Name of Instruments	Required Quantity	Score
1.	Blood bank refrigerator 2 to 4° C	2	
2.	Ordinary centrifuge	3	
3.	Deep freezer (-20°C to -30°C)	1	
4.	Deep freezer (-80°C)	1	
5.	Platelet Shaker	1	
6.	Autoclave	1	
7.	Computer with printer	1	
8.	Gamma radiation chamber	1 (optional*)	
9.	Microscope	1	
10.	Auto pipettes (20, 50, 100 µl)	2 each	
11.	Incubator	2	
12.	Water Bath	3	
13.	Hot Air Oven	2	
14.	Generator 60 KVA	as per need	
**Total score**			
**Total percentage= Total Score/ 14 x 100**			

Each row gets a score of 1 if the mentioned test is available otherwise 0.

Scoring chart	
**Total Percentage**	**Score**
0-50	0
50-70	1
70-85	2
85-100	3
Score 2.11.1.2.3.3	

Area	Code	Verification		
**X-ray**	**2.11.2**			
**Components**		**Service Standards**	**Obtained Score**	**Maximum Score**
2.11.2.1 Time for patients	2.11.2.1.1	X-ray service is open from 10 AM to 3 PM		1
	2.11.2.1.2	Emergency x-ray service is available round the clock		1
2.11.2.2 Staffing	2.11.2.2	Adequate numbers of trained healthcare workers are available in x-ray (at least 2 staffs to cover shifts including ER)		1
2.11.2.3 Patient counseling	2.11.2.3	Counseling is provided to patients about radiation hazard, site and position for x-ray		1
2.11.2.4 Information education and communication materials for patients	2.11.2.4	Radiation sign, appropriate Radiation awareness posters, leaflets are available in the department and OPD waiting area.		1
2.11.2.5 Instruments and equipment	2.11.2.5	General X ray unit (with minimum 125KV and 300ma X-ray machine) with floatation table top and vertical bucky		1
	2.11.2.6	Mobile X ray unit 1 for bed side radiography for inpatient is available and functioning.		1
	2.11.2.7	Complete CR system with CR cassette at least 5 of 14 x 17 inch and 3 of 10x12inch.		1
2.11.2.8 Physical facilities	2.11.2.8.1	X ray room of at least 4x4sqm with wall of at least 23cm of brick or 6cm RCC or 2mm lead equivalent.		1
	2.11.2.8.2	Light and ventilation are adequately maintained.		1
	2.11.2.8.3	The required furniture and supplies including radiation protective measures for patients, visitors and staffs are available including lead gown		1
2.11.2.9 Duty rosters	2.11.2.9	Duty rosters of X-ray are developed regularly and available in appropriate location.		1
2.11.2.10 Facilities for patients	2.11.2.10	Waiting space with sitting arrangement is available for at least 5 persons in waiting lobby.		1
2.11.2.11 Recording and reporting	2.11.2.11.1	X-ray is adequately recorded as per requisition form with detail information of patients, date of x-ray and site and view		1
	2.11.2.11.2	Report have adequate information of patient and checked by designated person before release		1
2.11.2.12 Information to patients	2.11.2.12	Biohazard signs and symbols are used at appropriate places		1

2.11.2.12 Infection prevention	2.11.2.12.1	Radiological waste is disposed based on HCWM guideline 2014 (MoHP)		1
	2.11.2.12.2	Hand-washing facility with running water and soap is available for practitioners		1
	2.11.2.12.3	Needle cutter is used		1
	2.11.2.12.4	Chlorine solution and bleach is available and utilized for decontamination		1
**Standard 2.11.2**		**Total Score**		20
		**Total Percentage (Total Score/ 20 x100)**		

**Area**	**Code**	**Verification**		
**Ultrasonography (USG)**	**2.11.3**			
**Components**		**Service Standards**	**Obtained Score**	**Maximum Score**
2.11.3.1 Time for patients	2.11.3.1	USG is open from 10 AM to 3 PM for obstetrics, abdominal, pelvic and superficial structure like testis, thyroid		1
2.11.3.2 Staffing	2.11.3.2	USG trained medical practitioner and mid-level health worker in each USG room		1
2.11.3.3 Patient counseling	2.11.3.3	Counseling is provided to patients about site and indication of USG		1
2.11.3.4 Maintaining patients' privacy	2.11.3.4	Appropriate techniques have been used to ensure the patient privacy (separate rooms, curtains hung, maintaining queuing of patients)		1
2.11.3.5 Instruments and equipment	2.11.3.5	USG machine (advanced) with different probes, computer and printer with USG papers , gel and wipes is available and functional		1
2.11.3.6 Physical facilities	2.11.3.6.1	Adequate space for practitioner and patient for USG with working table and examination bed one per each USG machine		1
	2.11.3.6.2	Proper light and ventilation maintained.		1
2.11.3.7 Facilities for patients	2.11.3.7	Comfortable waiting space with sitting arrangement is available for at least 15 persons in waiting lobby.		1
2.11.3.8 Recording and reporting	2.11.3.8.1	USG is adequately recorded as per requisition form with detail information of patients, date of USG		1
	2.11.3.8.2	Report have adequate information of patient, information of area of examination and radiological opinion, further referral and checked by designated person before release		1

2.11.3.9 Infection prevention	2.11.3.9.1	Hand-washing facility with running water and soap is available for practitioners		1
	2.11.3.9.2	Chlorine solution and bleach is available and utilized for decontamination		1
**Standard 2.11.3**		**Total Score**		12
		**Total Percentage (Total Score/ 12 x100)**		

Area	Code	Verification		
**Electrocardiogram (ECG)**	**2.11.4**			
**Components**		**Service Standards**	**Obtained Score**	**Maximum Score**
2.11.4.1 Service available	2.11.4.1	ECG service is available for patients in OPD, Emergency and Indoor		1
2.11.4.2 Space	2.11.4.2	Separate space is dedicated for ECG Service		1
2.11.4.3 Patient counseling	2.11.4.3	Counseling is provided to patients about procedure and indication of ECG		1
2.11.4.4 Maintaining patient privacy	2.11.4.4	Appropriate techniques have been used to ensure the patient privacy (separate rooms, curtains hung, maintaining queuing of patients)		1
2.11.4.5 Instruments, equipment and supplies	2.11.4.5	Functional ECG machine (12 lead with power back up), paper, gel, wipes and hand sanitizer are available in ECG trolley		1
2.11.4.6 Recording and reporting	2.11.4.6.1	ECG is adequately recorded as per requisition form with detail information of patients, date of ECG		1
	2.11.4.6.2	Reporting folder of ECG should have adequate information of patient, including analysis of 12 lead ECG with final impression of ECG diagnosis done by designated person before release		1
2.11.4.7 Infection prevention	2.11.4.7.1	Hand-washing facility with running water and liquid soap is available for practitioners		1
	2.11.4.7.2	Chlorine solution and bleach is available and utilized for decontamination		1
Standard 2.11.4		**Total Score**		9
		**Total Percentage (Total Score/ 9 x100)**		

**Echocardiogram (Echo)**	**2.11.6**	**Verification**		
**Components**		**Service Standards**	**Obtained Score**	**Maximum Score**
2.11.6.1 Adequate time for patients	2.11.6.1.1	Echo service is available from 10 AM to 3 PM		1
	2.11.6.1.2	Emergency Echo is available round the clock (optional)		
2.11.6.2 Adequate health workers	2.11.6.2	Cardiologist is available for Echo service with at least one mid-level health worker assigned in Echo		1
2.11.6.3 Patient counseling	2.11.6.3	Counseling is provided to patients about procedure and indication of Echo		1
2.11.6.4 Adequate instruments, equipment and supplies	2.11.6.4	Functional Echo machine (2D, M-mode, color doppler), computer and printer with Echo papers, gel, wipes and hand sanitizer are available		1
2.11.6.5 Adequate physical facilities	2.11.6.5.1	Separate space allocated for Echo with changing room and patients' gown		1
	2.11.6.5.2	Proper light and ventilation are adequately maintained		1
	2.11.6.5.3	Echo examination bed with mattress and bed cover and pillow with curtains for privacy of patients		1
2.11.6.6 Recording and reporting	2.11.6.6.1	Echo is adequately recorded as per requisition form with detail information of patients, date of echo and echo diagnosis		1
	2.11.6.6.2	Report have adequate information of patient and checked by designated person before release		1
2.11.6.7 Infection prevention	2.11.6.7.1	Hand-washing facility with running water and soap is available for practitioners		1
	2.11.6.7.2	Chlorine solution and bleach is available and utilized		1
**Standard 2.11.6**		**Total Score**		11
		**Total Percentage (Total Score/ 11 x100)**		

**Treadmill (TMT)**	2.11.7	Verification		
Components		Service Standards	Obtained Score	Maximum Score
2.11.7.1 Adequate time for patients	2.11.7.1	Treadmill (TMT) service is available from 10 AM to 3 PM		1
2.11.7.2 Adequate health workers	2.11.7.2	At least one trained medical officer / cardiologist and one mid-level health worker is allocated for TMT service		1

2.11.7.3 Patient counseling	2.11.7.3	Counseling is provided to patients about procedure, indication and anticipated complication during TMT as well as to modify the routine cardiac medications as per TMT protocol.		1
2.11.7.4 Adequate instruments, equipment, medicines and supplies	2.11.7.4.1	Functional TMT machine with display monitor and printer, paper, ECG lead, wipes and hand sanitizer are available		1
	2.11.7.4.2	Emergency trolley with emergency drugs and supplies readily available (See Annex 2.11.7a Emergency Trolley TMT At the end of this standard)		3
	2.11.7.4.3	Synchronized Defibrillator is available and functional in TMT room		1
	2.11.7.4.4	Injection Dobutamine available for Stress TMT		1
2.11.7.5 Adequate physical facilities	2.11.7.5.1	Separate room allocated for TMT with changing room, gown for patient and locker for patients' belongings		1
	2.11.7.5.2	Light and ventilation are adequately maintained.		1
2.11.7.6 Recording and reporting	2.11.7.6.1	TMT is adequately recorded as per requisition form with detail information of patients, date of TMT		1
	2.11.7.6.2	TMT report should have adequate information of patient, achievement of target heart rate, blood pressure response with exercise, any ECG changes, any complication observed during exercise and recovery period and checked by designated person before release		1
2.11.7.7 Infection prevention	2.11.7.7.1	Hand-washing facility with running water and liquid soap is available for practitioners		1
	2.11.7.7.2	Chlorine solution and bleach is available and utilized for decontamination		1
**Standard 2.11.7**		**Total Score**		15
		**Total Percentage = Total Score/ 15x100**		

Annex 2.11.7a Emergency Trolley TMT

SN	Name	Required No	Score
1.	Atropine Injection	10	
2.	Adrenaline Injection	3	
3.	Xylocaine 1% and 2% Injections with Adrenaline	2	
4.	Xylocaine 1% and 2 % Injections without Adrenaline	2	
5.	Xylocaine Gel	2	
6.	Diclofenac Injection	5	
7.	Hyoscine Butylbromide Injection	5	

8.	Diazepam injection	2	
9.	Morphine Injection / Injection Pethidine	2	
10.	Hydrocortisone Injection	4	
11.	Antihistamine Injection	4	
12.	Dexamethasone Injection	4	
13.	Ranitidine/Omeperazole Injection	4	
14.	Frusemide Injection	5	
15.	Dopamine injection	2	
16.	Noradrenaline injection	2	
17.	Digoxin injection	2	
18.	Verapamil injection	2	
19.	Amidarone injection	2	
20.	GTN	1	
21.	Labetolol injection	1	
22.	Sodium bicarbonate injection	2	
23.	Injection Cefixim (Antibiotic)	4	
24.	Injection Metronidazole	4	
25.	D25% ampoule	2	
26.	IV Infusion set (Adult/Pediatric)	2	
27.	IV Catheters 18, 20, 22, 24, 26	2 each	
28.	Disposable syringes 1 ml, 3 ml, 5 ml, 10 ml, 20 ml, 50 ml	5 each	
29.	Disposable Gloves 6, 6.5, 7, 7.5	3 each	
30.	Distilled Water	3	
31.	Needle 18-25	As per need	
32.	Ezi vac (for enema) or Enema Can Set	5/1	
		**Total Score**	
		**Total Percentage (Total Score/ 32x100)**	

Each row gets a score of 1 if the mentioned test is available otherwise 0.

**Scoring chart**	
**Total Percentage**	**Score**
0-50	0
50-70	1
70-85	2
85-100	3
Score 2.11.7.4.2	

Endoscopy	2.11.8	Verification		
**Components**		**Service Standards**	**Obtained Score**	**Maximum Score**
2.11.8.1 Adequate time for patients	2.11.8.1. 1	Endoscopy service is available from 10 AM to 3 PM		1
	2.11.8.1. 2	Emergency endoscopy is available round the clock		1
2.11.8.2 Adequate physical facilities	2.11.8.2.1	Separate room designated for endoscopy with adjustable patient bed, head adjustable recovery bed, working chair for practitioner and working table with at least two chairs		1
	2.11.8.2.2	Scopes hanging area for storage. (covered cupboards/ shelves)		1
	2.11.8.2.3	Proper light and ventilation maintained		1
2.11.8.3 Adequate staffing	2.11.8.3.1	Physician/ surgeons having endoscopic training or Gastroenterologist or hepatologist or gastrointestinal surgeons with at least 2 Trained endoscopic nurse/paramedic designated for endoscopy room		1
	2.11.8.3.2	One mid-level trained health worker for record keeping, booking, and report dispatch , counselling before procedure		1
2.11.8.4 Patient counseling	2.11.8.4	Counseling is provided to patients about procedure and indication of Endoscopy with possible complications		1
2.11.8.5 Adequate instruments, equipment, medicines and supplies	2.11.8.5.1	Functional Video-endoscopy machine with attachable gastroscope and colonscope; bronchoscope (optional) with cardiac monitor available		1
	2.11.8.5.2	Portable Diathermy machine with cautery wires for endoscopic procedure standard.		1
	2.11.8.5.3	Emergency trolley with emergency drugs and supplies readily available (See Annex 2.11.8a Emergency Trolley Endoscopy At the end of this standard)		3
2.11.8.6 Recording and reporting	2.11.8.6.1	Recording and printing system (computer, color printer with capture card and reporting software for UGI endoscopy, colonoscopy)		1
	2.11.8.6.2	Endoscopy report is adequately recorded as per requisition form with detail information of patients, date of endoscopy and pictures attached		1
	2.11.8.6.3	Report have adequate information of patient, visibly printed pictures captured during endoscopy, endoscopic observation of structure, diagnosis and relevant endoscopic procedures performed and complications observed if any and checked by designated person before release		1

2.11.8.7 Infection prevention	2.11.8.7.1	Hand-washing facility with running water and soap is available for practitioners		1
	2.11.8.7.2	Personal protective equipment including utility gloves and boots available and used		1
	2.11.8.7.3	Separate tubs for washing, disinfection and final cleaning of the scopes available and used		1
	2.11.8.7.4	Disinfectant solution (gluteraldehyde) and Citezyme solution for enzymatic cleaning of scopes available and used		1
	2.11.8.7.5	Chlorine solution and bleach is available and utilized for decontamination (*not for scopes)		1
**Standard 2.11.8**		**Total Score**		21
		**Total Percentage (Total Score/ 21 x100)**		

Annex 2.11.8a Emergency Trolley Endoscopy

SN	Name	Required No	Score
1.	Atropine Injection	10	
2.	Adrenaline Injection	3	
3.	Xylocaine 1% and 2% Injections with Adrenaline	2	
4.	Xylocaine 1% and 2% Injections without Adrenaline	2	
5.	Xylocaine Gel	2	
6.	Diclofenac Injection	5	
7.	Hyoscine Butylbromide Injection	5	
8.	Diazepam injection	2	
9.	Morphine Injection / Injection Pethidine	2	
10.	Hydrocortisone Injection	4	
11.	Antihistamine Injection	4	
12.	Dexamethasone Injection	4	
13.	Ranitidine/Omeperazole Injection	4	
14.	Frusemide Injection	5	
15.	Dopamine injection	2	
16.	Noradrenaline injection	2	
17.	Digoxin injection	2	
18.	Verapamil injection	2	

19.	Amidarone injection	2	
20.	GTN	1	
21.	Labetolol injection	1	
22.	Sodium bicarbonate injection	2	
23.	Injection Cefixim (Antibiotic)	4	
24.	Injection Metronidazole	4	
25.	D25% ampoule	2	
26.	IV Infusion set (Adult/Pediatric)	2	
27.	IV Catheters 18, 20, 22, 24, 26	2 each	
28.	Disposable syringes 1 ml, 3 ml, 5 ml, 10 ml, 20 ml, 50 ml	5 each	
29.	Disposable Gloves 6, 6.5, 7, 7.5	3 each	
30.	Distilled Water	3	
31.	Needle 18-25	As per need	
32.	Ezi vac (for enema) or Enema can set	5/ 1 set	
		**Total Score**	
		**Total Percentage (Total Score/ 32x100)**	

Each row gets a score of 1 if the mentioned test is available otherwise 0.

**Scoring chart**	
**Total Percentage**	**Score**
0-50	0
50-70	1
70-85	2
85-100	3
**Score for Standard 2.11.8.6.3**	

**Audiometry**	**2.11.9**	**Verification**		
**Components**		**Service Standards**	**Obtained Score**	**Maximum Score**
2.11.9.1 Adequate time for patients	2.11.9.1	Audiometry service is available from 10 AM to 3 PM		1
2.11.9.2 Adequate health workers	2.11.9.2	ENT specialist is available for performing audiometry		1

2.11.9.3 Patient counseling	2.11.9.3	Counseling is provided to patients about procedure and indication of audiometry and explain patient about the examination booth		1
2.11.9.4 Adequate instruments, equipment and supplies	2.11.9.4	Functional Audiometer with power supply and response switch, headphones, earphones with audiometric calibration stand and 500g weight and audiometer patch cords available		1
2.11.9.5 Adequate physical facilities	2.11.9.5.1	Separate room allocated for audiometry with a special booth made of acoustic medium is available with custom-built triangular table with the audiometer on top and the computer tower beneath and an area outside booth for working area for technologist		1
	2.11.9.5.2	Sound dampening materials on the interior walls of the exam room and a rubber seal on the hallway door		1
	2.11.9.5.3	Light and ventilation are adequately maintained.		1
2.11.9.6 Recording and reporting	2.11.9.6.1	Computer available for recording and reporting of the test results		1
	2.11.9.6.2	Audiometry is adequately recorded as per requisition form with detail information of patients and diagnosis		1
	2.11.9.6.3	Report have adequate information of patient, results of audiometry and advices for further hearing related treatment and checked by designated person before release		1
2.11.9.7 Infection prevention	2.11.9.7.1	Hand-washing facility with running water and soap is available for practitioners		1
	2.11.9.7.2	Chlorine solution and bleach is available and utilized		1
**Standard 2.11.9**		**Total Score**		12
		**Total Percentage (Total Score/ 12x100)**		

**CT Scan**	**2.11.10**			
**Components**		**Verification Criteria**	**Obtained Score**	**Maximum Score**
2.11.10.1 Adequate time for patients	2.11.10.1.1	CT Scan service is open from 10 AM to 3 PM and appointments date given		1
	2.11.10.1.2	Emergency CT Scan service is available round the clock		1
2.11.10.2 Adequate staffing	2.11.10.2	Adequate numbers of trained healthcare workers are available in CT scan (at least 2 staffs to cover shifts including ER) under supervision of Radiologists		1
2.11.10.3 Patient counseling	2.11.10.3	Counseling is provided to patients about radiation hazard, site and position for CT Scan and assessed for claustrophobia		1

2.11.10.4 Information education and communication materials for patients	2.11.10.4	Radiation sign, appropriate Radiation awareness posters, leaflets are available in the department and OPD waiting area.		1
2.11.10.5 Adequate instruments and equipment	2.11.10.5	CT Scan Machine (at least 128 slice) available and functional at least 1		1
2.11.10.6 Adequate physical facilities	2.11.10.6.1	CT Scan room of at least 16x20 feet with wall of at least 23cm of brick or 6cm RCC or 2mm lead equivalent and control room of 10x 12 feet		1
	2.11.10.6.2	Light and ventilation are adequately maintained with help of air conditioner and exhaust fans.		1
	2.11.10.6.3	The required furniture and supplies including radiation protective measures for patients,, visitors and staffs are available including magnetic gown		1
2.11.10.7 Duty rosters	2.11.10.7	Duty rosters for CT Scan service are developed regularly and available in appropriate location.		1
2.11.10.8 Facilities for patients	2.11.10.8.1	Comfortable waiting space with sitting arrangement is available for at least 5 persons in waiting lobby.		1
	2.11.10.8.2	Safe drinking water is available in the waiting lobby throughout the day.		1
2.11.10.9 Recording and reporting	2.11.10.9.1	CT Scan is adequately recorded as per requisition form with detail information of patients, date of Ct Scan and site and view		1
	2.11.10.9.2	Report have adequate information of patient, radiological diagnosis of CT Scan and checked by designated person before release		1
2.11.10.10 Information to patients	2.11.10.10	Biohazard signs and symbols are used at appropriate places		1
2.11.10.11 Infection prevention	2.11.10.11.1	Radiological waste is disposed based on HCWM guideline 2014 (MoHP)		1
	2.11.10.11.2	Hand-washing facility with running water and soap is available for practitioners		1
	2.11.10.11.3	Needle cutter is used		1
	2.11.10.11.4	Chlorine solution and bleach is available and utilized for decontamination		1
**Standard 2.11.10**		**Total Score**		19
		**Total Percentage (Total Score/ 19 x100)**		

Area	Code	Verification		
**Postmortem**	**2.12.1**			
**Components**		**Service Standards**	**Obtained score**	**Maximum Score**
2.12.1.1 Physical facility	2.12.1.1.2	Designated area for mortuary room, changing room and store room and bathroom		1
	2.12.1.1.2	Body dissection table (at least one) is available and used		1
	2.12.1.1.3	Organ dissection table (at least one) is available and used		1
	2.12.1.1.4	Adequate ventilation and light and odor management		1
2.12.1.2 Availability of postmortem services	2.12.1.2	Examination of the dead body in any unnatural death and suspicious death (Post-mortem examination or autopsy) available from 9 am to 5pm		1
2.12.1.3 Staffing	2.12.1.3	At least one MD forensic and one trained medical officer for autopsy and clinical medico-legal services		1
2.12.1.4 Supplies and instruments	2.12.1.4	Adequate supplies and instruments for forensic services (See Annex 2.12.1a Supplies and instrument for post mortem At the end of this standard)		3
2.12.1.5 Mortuary van	2.12.1.5	Mortuary van is available 24 hours (at least one)		1
2.12.1.6 Recording and reporting	2.12.1.6	Standardized medico-legal examination formats available		1
2.12.1.7 Infection prevention	2.12.1.7.1	Staff wear mask and gloves at work.		1
	2.12.1.7.2	There are colored bins for waste segregation and disposal based on HCWM guideline 2014 (MoHP)		1
	2.12.1.7.3	Hand-washing facility with running water and soap is available and being practiced.		1
	2.7.11.4	Chlorine solution is available and utilized.		1
	2.12.1.7.5	Proper disposal of anatomical waste in placenta pit after autoclaving		1
**Standard 2.12.1**		**Total Score**		14
		**Total Percentage (Total Score/ 19 x100)**		

Annex 2.12.1a Supplies and instrument for post mortem services

S.No.	Supplies and instrument	Required Number	Score
1.	Refrigeration chamber or cool room for body preservation	8-10 bodies capacity	
2.	Dissection set of instruments for autopsy	2 sets	
3.	Magnifying lens; 20 and 40 times	1 each	
4.	Measuring tape	2	
5.	Weighing machine for organs and if possible for dead body	1	
6.	Camera for photography	1	
7.	Glass tubes for blood collection and tissue collection; reasonable numbers for regular use	as per need	
8.	Glass slides; reasonable number for regular use	as per need	
9.	EDTA	as per need	
10.	Sodium Floride -200 or 500 gm	As per need	
11.	Formalin solution	as per need	
12.	Plastic made wide mouth containers of 500 ml capacity ; reasonable numbers for regular need	as per need	
13.	Sodium chloride (table salt); reasonable amount for regular use	as per need	
14.	Autopsy gown	2 sets	
15.	Gum boots	2 pairs	
16.	Gloves and masks	as per need	
17.	Computer with printer for report preparation	1	
18.	Cleaning agents; soap, detergents	as per need	
19.	Sealing materials; specific seal tape or wax seal and seal print	as per need	
20.	Autopsy and skeletal remains SOP, Reference Manual	as per need	
		**Total score**	
		Percentage= **Total score**/20 x 100	

Each row gets a score of 1 if the mentioned test is available otherwise 0.

**Scoring**	
**Total Percentage**	**Score**
0-50	0
50-70	1
70-85	2
85-100	3
**Score for Standard 2.12.1.6.1**	

Area	Code	Verification		
**Medico-legal services**	**2.12.2**			
**Components**		**Service Standards**	**Obtained score**	**Maximum Score**
2.12.2.1 Physical facility	2.12.2.1	Designated area for medico-legal examination with examination bed and working desk with chair		1
2.12.2.2 Availability of medico-legal services	2.12.2.2.1	Medico-legal services are available 24 hours		1
	2.12.2.2.2	Injury examination and reporting in cases of physical assaults, attempted murder, sexual offenses (victim and accused), metal status examination, torture victim examination and drunkenness examination and at least one examination bed allocated for it		1
2.12.2.3 Staffing	2.12.2.3	At least one MD forensic and one trained medical officer for autopsy and clinical medico-legal services		1
2.12.2.4 Supplies and instruments	2.12.2.4.1	Adequate supplies and instruments for medico-legal services (See Annex 2.12.2a Supplies and instrument for medico legal services At the end of this standard)		3
	2.12.2.4.2	Preservation of sample ensured before dispatching for test		1
2.12.2.5 Patient counseling	2.12.2.5	Post-traumatic counseling is done to the victims of medico-legal issues like sexual offence		1
2.12.2.6 Recording and reporting	2.12.2.6	Standardized medico-legal examination formats available		1
2.12.2.7 Infection prevention	2.12.2.7.1	Staff wear mask and gloves at work.		1
	2.12.2.7.2	There are well labelled colored bins for waste segregation and disposal based on HCWM guideline 2014 (MoHP)		1
	2.12.2.7.3	Hand-washing facility with running water and soap is available and being practiced.		1
	2.12.2.7.4	Chlorine solution is available and utilized.		1
**Standard 2.12.2**		**Total Score**		14
		**Total Percentage (Total Score/ 14 x100)**		

Annex 2.12.2a Supplies and instrument for clinical medico-legal services

S.No.	Supplies and instrument	Required number	Score
1.	Weight machine and height scale	1 each	
2.	BP set, stethoscope and torch light	1 each	
3.	Examination kits; sexual offence cases (rape victim examination kit)	as per need	
4.	Gloves and masks	as per need	
5.	Magnifying lens; 20 and 40 times	1 each	
6.	Measuring tape	As per need	
7.	Camera for photography	1	
8.	Paper envelopes of different sizes for collection of samples and packing	as per need	
9.	Glass tubes for collection of blood urine; reasonable number for regular use	as per need	
10.	X ray plate view box	1	
11.	EDTA and Sodium floride 500 gm	As per need	
12.	Glass slides; reasonable number for regular use	as per need	
13.	Cupboards for store and necessary other furniture for examination room	as per need	
14.	Sealing materials as for autopsy room	as per need	
15.	Computer and printer for report preparation as in autopsy	1	
16.	SOPs and Reference Manuals for age estimation, sexual offence case examination, injury examination, drunkenness examination, mental state examination and torture victim examination.	1	
		**Total score**	
		Percentage= **Total score**/16 x 100	

Each row gets a score of 1 if the mentioned test is available otherwise 0.

**Scoring**	
**Total Percentage**	**Score**
0-50	0
50-70	1
70-85	2
85-100	3
**Score for Standard 2.12.2.6.2**	

Area	Code	Verification		
**One stop crisis management center (OCMC)**	**2.13**			
**Components**		**Service Standards**	**Obtained Score**	**Maximum Score**
2.13.1 OCMC coordination committee exists (multi-sectoral)	2.13.1.	Quarterly meeting minute of coordination committee		1
2.13.2 Functionality of case management committee	2.13.2	Monthly meeting minute of case management committee		1
2.13.3 Timely service for patients	2.13.3.1	Prioritized services for GBV victims/survivors exits		1
	2.13.3.2	Treatment for GBV survivors/affected by GBV is available 24 hours		1
2.13.4 Physical facilities for OCMC services	2.13.4	Separate space allocated for OCMC services with adequate physical facilities (See Annex 2.13a Physical facilities for OCMC At the end of this standard)		3
2.13.5 Staffing	2.13.5.1	At least one Medical officer working in the hospital trained in medico-legal issues is available		1
	2.13.5.2	At least two Staff nurse working in the hospital and 1 trained psycho social counselor		1
2.13.6 Timely examination from medico-legal aspects and treatment of GBV survivors/ affected by GBV	2.13.6.1	Health check-up,medico-legal examination including documentation (See Annex2.13b Instruments and supplies for treatment in OCMC At the end of this standard)		3
	2.13.6.2	Preservation of samples as legal evidence done for future use (See Annex 2.13.3c Instruments and supplies for evidence collection in OCMC At the end of this standard)		3
	2.13.6.3	Pregnancy test and emergency contraceptive services, tests for HIV/HBV available		1
2.13.7 Use of GBV clinical protocol	2.13.7.1	Whole site orientation on GBV clinical protocol conducted		1
	2.13.7.2	Availability and use of the treatment as per the protocol and OCMC guideline		1

2.13.8 Psycho-social counselling of GBV Survivors/ affected by GBV and rehabilitation	2.13.8.1	Mental health and psychosocial counselling services available		1
	2.13.8.2	If the female survivor requires to stay more days or requires advance psychosocial counseling including livelihood training, she/ he shall be referred to nearby appropriate safe home/ rehabilitation centers		1
2.13.9 Referral services in place	2.13.9.1	Provide required referral and other services (as per the health service guideline and protocol). (Beyond health: security, legal, shelter, rehabilitation)		1
	2.13.9.2	Health related referral services e.g. Safe abortion services		1
2.13.10 Information, education and empowerment for GBV survivors/ affected by GBV	2.13.10.1	Detailed information concerning the services being provided by OCMC to the survivors of GBV (Citizen charter, leaflets, community radio etc)		1
	2.13.10.2	Information is being given in an integrated manner (Safe home related, OCMC, Police women children service unit)		1
2.13.11 Recording and reporting	2.13.11.1	Details of the events registered in the OCMC, services (health and non-health) being provided to the survivors, listing of the referred organizations shall be documented to be classified and analyzed in due course.		1
	2.13.11.2	Confidentiality shall be maintained at all stages of documentation.		1
	2.13.11.3	Report to concerned authority (DCC and MoHP) in monthly report service format		1
	2.13.11.4	Documentation of the current status of GBV survivors of at least last 1 year is done		1
2.13.12 Infection prevention	2.13.12.1	Masks and gloves are available and used		1
	2.13.12.2	There are colored bins for waste segregation and disposal based on HCWM guideline 2014 (MoHP)		1
	2.13.12.3	Hand-washing facility with running water and soap is available for practitioners.		1
	2.13.12.4	Needle cutter is used		1
	2.13.12.5	Chlorine solution is available and utilized.		1
**Standard 2.13**		**Total Score**		29
		**Total Percentage (Total Score/ 29 x100)**		

Annex 2.13a Physical Facilities for OCMC

SN	General Items	Required No.	Score
1	Rooms for treatment room/examination room, office and guard room	1 each	
2	Toilet allocated for OCMC services	as per need	
3	Curtains to maintain confidentiality during the forensic examination	as per need	
4	Examination table	1	
5	Desk	1	
6	Chairs	3	
7	Cupboard to keep clients' information with filing cabinet	1	
8	Movable table lamp	1	
9	toilet and bathroom for clients' use (water, bucket, mug, soap, towel)	as per need	
10	Hand washing facility for service provider	as per need	
11	Refrigerator and lockable cupboard for specimen store	1	
12	Telephone	1	
13	Computer and printer	1set	
14	Boiler (for tea)	1 set	
		**Total score**	
		**Percentage= Total score/14 x 100**	

Each row gets a score of **1** if the mentioned test is available otherwise **0**.

**Scoring**	
**Total Percentage**	**Score**
0-50	0
50-70	1
70-85	2
85-100	3
**Score for Standard 2.13.4**	

Annex 2.13b Instruments and supplies for treatment in OCMC

SN	General Items	Required No.	Score
1	Sphygmomanometer (B.P. Instrument)	1	
2	Stethoscope	1	
3	Torch Light	1	
4	Tongue Depressor	as per need	
5	Tourniquet	1	
6	Sterilized Gloves as required	as per need	
7	Sterilized Syringe and Needles as required	as per need	
8	Cotton and Bandage as required	as per need	
9	Sterilized Vial for sample collection	as per need	
10	Different sized Reflecting Mirrors (big, medium and small)	1 each	
11	Sterilized Speculum	1	
12	Glutaradehylde solution for high level of infection prevention	as per need	
13	Chlorine powder to sterilize the used materials/tools	as per need	
14	Protoscope /Anscope	1	
15	Pregnancy Test Kit	as per need	
16	Specimen collection materials for communicable Sexually Transmitted Infections	as per need	
17	Lubricant, Clean Water, Normal Saline	as per need	
18	Tray for sharp instruments, such as scissors, knife etc.	as per need	
19	Height Measuring Scale	1	
20	Weight Measuring Scale	1	
		**Total score**	
		**Total Percentage = Total Score/ 20 x 100**	

Each row gets a score of **1** if the mentioned test is available otherwise **0**.

**Scoring**	
**Total Percentage**	**Score**
0-50	0
50-70	1
70-85	2
85-100	3
**Score for Standard 2.13b**	

Annex 2.13c Instruments and supplies for evidence collection in OCMC

SN	General Items	Required No.	Score
1	Cotton/material to collect sperm, Blood, Saliva etc. from survivor	as per need	
2	Container/vessel to keep the collected specimen	as per need	
3	Materials to swab	as per need	
4	Microscope slide	as per need	
5	Vials for blood collection	as per need	
6	Vials to collect urine for pregnancy test	as per need	
7	Paper or plastic seat	as per need	
8	Paper bag to hold clothes and other items	as per need	
9	Air spatula and slide for pap smear	as per need	
10	Fixing solutions: hair spray, alcohol etc	as per need	
11	Analgesic : Normal medications like Paracetamol, Ibuprofen etc. for pain relief	as per need	
12	Emergency Contraceptives: Pills and IUCD	as per need	
13	Thread for Suturing	as per need	
14	Immunization for Tetanus and Hepatitis	as per need	
15	STI Preventive	as per need	
16	Bed Sheet and Blankets for examination table	as per need	
17	Towel	as per need	
18	Clothes for Survivor (if her clothes are torn or stained).	as per need	
19	Gown to be worn during the examination	as per need	
20	Sanitary Pads and Tampons for internal use	as per need	
21	Documentation forms and recording forms	as per need	
22	Camera and Film for evidence collection	1	
24	Colposcope or Magnifying Glass	1	
**Total score**			
**Total Percentage = Total Score/ 24 x 100**			

Each row gets a score of **1** if the mentioned test is available otherwise **0**.

**Scoring**	
**Total Percentage**	**Score**
0-50	0
50-70	1
70-85	2
85-100	3
**Score for Standard 2.13.6.2**	

Area	Code	Verification		
**Physiotherapy (Physical Rehabilitation)**	**2.14**			
**Components**		**Service Standards**	**Obtained Score**	**Maximum Score**
2.14.1 Space	2.14.1	Separate room for OPD physiotherapy with at least 3 physiotherapy beds and physiotherapy hall		1
2.14.2Time for patients	2.14.2.1	Physiotherapy OPD is open from 10 AM to 5 PM.		1
	2.14.2.2	Inpatient physiotherapy service is available based on the requisition		1
2.14.3 Staffing	2.14.3	At least 1 physiotherapist trained in		1
2.14.4 Maintaining patient privacy	2.14.4	Appropriate techniques have been used to ensure the patient privacy (separate rooms, curtains hung, maintaining queuing of patients).		1
2.14.5 Patient counseling	2.14.5	Counseling is provided to patients about the type of treatment being given and its consequences.		1
2.14.6 IEC/BCC materials	2.14.6	Appropriate IEC/BCC materials (posters, leaflets etc.) are available in the OPD waiting area.		1
2.14.7 Instruments and equipment	2.14.7	Instruments and equipment to carry out the Physiotherapy works are available and functioning (See Annex 2.14a Instruments and equipment physiotherapy At the end of this standard)		3
2.14.8 Physical facilities	2.14.8.1	Adequate rooms and space for the practitioners and patients are available.		1
	2.14.8.2	Light and ventilation are adequately maintained.		1
2.14.9 Duty rosters	2.14.9	Duty rosters of OPD are developed regularly and available in appropriate location.		1
2.14.10 Facilities for patients	2.14.10.1	Safe drinking water is available in the waiting lobby throughout the day.		1
	2.14.10.2	Hand-washing facilities are available for patients.		1
2.14.11 Recording and reporting	2.14.11.1	Recording and reporting throughout treatment and follow up is done		1
2.14.12 Infection prevention	2.14.12.1	Masks and gloves are available and used		1
	2.14.12.2	There are colored bins for waste segregation and disposal based on HCWM guideline 2014 (MoHP)		1
	2.14.12.3	Hand-washing facility with running water and soap is available for practitioners.		1
	2.14.12.4	Needle cutter is used		1
	2.14.12.5	Chlorine solution is available and utilized.		1
**Standard 2.14**		**Total Score**		21
		Percentage = **Total Score** / 21 x 100		

Annex 2.14a Instruments and equipment physiotherapy

SN	Instruments and equipment	Required No.	Score
1	Traction unit	2	
2	IFT(Interferential treatment)	4	
3	Ultrasound(treatment) unit	4	
4	TENS (Transcutaneous nerve stimulation)	4	
5	Muscle stimulator	3	
6	Parallel bar	1	
7	Quadriceps Table	1	
8	Therabands	5	
9	Heel exerciser	1	
10	CPM machine knee and elbow	1	
12	Physio ball 55" 65" and 90"	3	
12	Moist heat unit	1	
13	Wax unit	1	
14	Foot step	1	
		**Total Score**	
		**Total Percentage= Total Score/14 x100**	

Each row gets a score of **1** if the mentioned test is available otherwise **0**.

**Scoring**	
**Total Percentage**	**Score**
0-50	0
50-70	1
70-85	2
85-100	3
**Score for Standard 2.14.7**	

**Dietetics and Nutrition rehabilitation**	**2.15**	**Verification**		
**Components**		**Standards**	**Obtained Score**	**Maximum Score**
2.15.1 Adequate space	2.15.1	Separate space allocated for Dietetics and Nutrition rehabilitation		1
2.15.2 Adequate time and access for patients	2.15.2.1	Dietetics and Nutrition rehabilitation unit opens from 10 am to 3 pm		1
	2.15.2.2	Inpatients monitored for nutritional needs, rehabilitation and therapeutic diets prescribed where needed		1
2.15.3 Adequate health workers	2.15.3	1 Senior dietitian (Masters in Nutrition & Dietetics with hospital internship, or Bachelors in Nutrition & Dietetics with one year's hospital experience), 1 dietetic assistant and 1 mid-level health workers trained in nutrition rehabilitation available for the dietetics and nutrition rehabilitation service		1
2.15.4 Maintaining patient privacy	2.15.4	Appropriate techniques have been used to ensure the patient privacy (separate rooms, curtains hung, maintaining queuing of patients, etc).		1
2.15.5 Patient counseling	2.15.5	Counseling is provided to patients about their nutritional status, diet prescription/ nutritional rehabilitation, use of local food as sources of the diet required and follow up		1
2.15.6 Information education and communication materials for patients	2.15.6	Appropriate IEC materials (posters, leaflets etc.) are available in waiting area on balanced diet and identification of malnutrition specially among children		1
2.15.7 Adequate instruments and equipment	2.15.7	Instruments and equipment to carry out the OPD works are available and functioning		1
2.15.8 Inpatient stabilization center for severe undernourished children with complications				
2.15.8.1 Adequate space	2.15.8.1	Separate space assigned for inpatient nutrition stabilization		1
2.15.8.2 Adequate staffing	2.15.8.2	Trained staffs assigned for inpatient nutrition stabilization		1
2.15.8.3 Adequate instrument, equipment, supplies	2.15.8.3	Instruments, equipment and supplies needed for inpatient stabilization center available and functional		1
2.15.8.4 Adequate physical facilities	2.15.8.4	Adequate rooms and space for the practitioners and patients are available.		1
	2.15.8.5	Light and ventilation are adequately maintained.		1
	2.15.8.6	The required furniture are available		1

2.15.9 Recording and reporting	2.15.9	Treatment, follow up and progress are recorded and reported		1
2.15.10 Facilities for patients	2.15.10.1	Safe drinking water is available in the waiting lobby throughout the day.		1
	2.15.10.2	Hand-washing facilities are available for patients.		1
2.15.11 Infection prevention	2.15.11.1	Masks and gloves are available.		1
	2.15.11.2	There are colored bins for waste segregation and disposal as per HCWM guideline 2014 (MoHP)		1
	2.15.11.3	Hand-washing facility with running water and soap is available for practitioners.		1
	2.15.11.4	Needle cutter is used		1
	2.15.11.5	Chlorine solution is available and utilized.		1
**Standard 2.15**		**Total Obtained Score**		**22**
		**Total Percentage (Total Obtained Score/ 22 x100)**		

**Area**	**Code**	**Verification**		
**Cardiac catheterization lab (Cath Lab)**	**2.16**			
**Components**		**Service Standards**	**Obtained Score**	**Maximum Score**
2.16.1 Time for Cath Lab	2.161.1	Routine procedures available on scheduled days		1
	2.16.1.2	Emergency procedures available round the clock		1
	2.16.1.3	At least two functional operating tables		1
2.16.2 Staffing	2.16.2	For one cardiac intervention, at least a team is composed of: MD Internal Medicine trained in cardiac intervention or cardiologist with one trained medical officer, two trained nursing/paramedics, and one trained office assistant		1
2.16.3 Cath Lab services available	2.16.3	Cath lab has at least following services available		
	2.16.3.1	Coronary Angiography		1
	2.16.3.2	Percutaneous transluminal coronary angioplasty/ percutaneous coronary intervention(PTCA)		1
	2.16.3.3	Right heart catheterization		1
	2.16.3.4	Pigtail insertion (Pericardiocentesis)		1

2.16.4 Patient counselling	2.16.4.1	Indications and reviews the clinical history and physical examination is documented		1
	2.16.4.2	Informed consent is taken before intervention; patients and caretakers are given appropriate counselling about the procedure.		1
2.16.5 Patient preparation	2.16.5	Preoperative instructions for patient preparation are given and practiced with routine pre-anesthesia check up		1
2.16.6 Post-procedure care	2.16.6.1	Separate area designated for post-procedure care to stabilize the patient after procedure		1
	2.16.6.2	Staffs are specified for the post-procedure care including close monitoring of the vital signs and observation of patient for bleeding in intervention site		1
	2.16.6.3	Adequate information shared for patient care and patient followed by at least one mid-level health worker for hand over or transfer of patient within or outside the hospital		1
2.16.7 Cath Lab	2.16.7.1	Cath Lab has appropriate physical set up (See Annex 2.16a Physical Set Up for Cath Lab At the end of this standard)		3
	2.16.7.2	General equipment, instruments and supplies available (See Annex 2.16b Furniture, Equipment, Instruments and Supplies for Cath Lab At the end of this standard)		3
	2.16.7.3	Medicines and supplies available (See Annex 2.16c General Medicine and Supplies for Cath lab At the end of this standard)		3
	2.16.7.4	Surgical sets for minimum list of the interventions available (See Annex 2.16d Surgical sets for Minimum list of Interventions At the end of this standard)		3
2.16.8 Safe Surgery Checklist	2.16.8	The Safe Surgery Checklist is available in Cath Lab and used		1
2.16.9 Records	2.16.9	Recording is done for all surgeries including procedure, observation during surgical procedure and complications if any		1
2.16.10 Infection prevention protocol is strictly followed by all staffs in Cath Lab				
2.16.10.1 Hand hygiene	2.16.10.1	Hand-washing and scrubbing facility with running water and soap is available and being practiced with elbow tap		1
2.16.10.2 Appropriate PPE	2.16.10.2	Gowns and sterile drapes for patients and sterile gown for surgery team are available and used whenever required.		1
2.16.10.3 Fumigation	2.16.10.3	Fumigation is done at least once a week in the Cath Lab		1
2.16.10.4 Disinfection of instruments	2.16.10.4	High Level Disinfection (e.g. Cidex) facility is available and being practiced.		1

2.16.10.5 High Dusting	2.16.10.5	High dusting is done at least twice a week in Cath Lab		1
2.16.10.6 Appropriate segregation of waste	2.16.10.6	Separate colored waste bins based on HCWM guideline 2014 (MoHP) are available and used		1
2.16.10.7 Disposal of sharps	2.16.10.7	Needle cutter is used		1
2.16.10.8 Cleaning	2.16.10.8	Chlorine solution is available and utilized for decontamination.		1
		**Total Score**		36
		**Total Percentage= Total Score/ 36 x 100**		

Annex 2.16a Physical Set Up for Cath Lab

SN	Physical Set Up	Score		
1.	Separate room designated for Cath Lab with recovery room and control room			
2.	Space designated for changing room for male and female staffs separately			
3.	Lockers for storage of the belongings of staffs			
4.	Separate shelves for storage of clean and dirty shoes at the entrance of the Cath Lab area demarked with red line			
5.	Space designated with sink facilitated with elbow tap for scrubbing			
6.	Designated space for tea room			
7.	Separate bathroom with at least one universal toilet for Cath Lab			
8.	Scrub basins with running water			
9.	Utility basins (at least 4)			
	**Total Score**			
	**Total percentage= Total Score/ 9 x 100**			

Each row gets a score of 1 if the mentioned test is available otherwise 0.

**Scoring chart**	
**Total percentage**	**Score**
0-50	0
50-70	1
70-85	2
85-100	3
**Score for Standard 2.15.7.1**	

Annex 2.16b Furniture, Equipment, Instruments and Supplies for Cath Lab			
**SN**	**General Equipment and Instruments for OT**	**Standard Quantity**	**Score**
1.	Wheel chair foldable, adult size	1	
2.	Stretcher	1	
3.	Patient trolley	1	
4.	Cupboards and cabinets for store	1	
5.	Working desk for anesthesia, nursing station, gowning	1 each	
6.	OT Table- universal type/ with wedge to position patient	At least 2	
7.	Flurosope	At least 1	
8.	Cardiac Monitors	At least 2	
9.	Computer with display monitor and printer in control room	At least 1 set	
10.	Examining table	1	
11.	Mayo Stand with tray	2	
12.	Operation theatre lights	1	
13.	Ultra violet light source	1	
14.	Electronic suction machine/ Foot-operated suction machine	1/1	
15.	Refrigerator / cold box	1	
16.	Boiler/ Autoclave	1/1	
17.	Oxygen concentrator/ Oxygen Cylinder/ Central oxygen	1/1/ available	
18.	Instrument trolley	2	
19.	BP instrument with stethoscope	1	
20.	Thermometer	1	
21.	Steel Drum for gloves	1	
22.	Steel Drum for Cotton	1	
23.	Tourniquet, latex rubber, 75 cm	2	
24.	Kidney tray (600cc)	2	
25.	Covered instrument trays	4	
26.	Mackintosh sheet	1	
27.	Lead gown	2 sets	

28.	Bowl stand	2	
29.	Cheatle forceps in jar	2	
30.	Packing towel double wrapper	As per need	
31.	Sterile gloves (6,6.5,7,7.5,8)	5/5/5/5/5/5	
32.	Towels/ eye hole	As per need	
33.	Masks and caps	As per need	
34.	Torch light and batteries	1 set	
35.	Foot steps	2	
36.	Wall clock	1	
37.	Waste bucket for scrub nurse	1	
38.	IV stand	2	
39.	Big tray, Big bowl, small bowl, kidney tray, sponge holder	2 each	
40.	Lap Pack	At least 2	
41.	IV set and PMO line	At least 10 each	
42.	Leak proof sharp container	1	
43.	Color coded waste bins (based on HCWM guideline 2014 (MoHP))	1 set per OT	
44.	Disposable syringes of different size, 3 way connector	As per need	
		**Total Score**	
		**Total percentage= Total/ 44 x 100**	

Each row gets a score of 1 if the mentioned test is available otherwise 0.

**Scoring Chart**	
**Total percentage**	**Score**
0-50	0
50-70	1
70-85	2
85-100	3
**Score for Standard 2.16.7.2**	

Annex 2.16c Medicine and Supplies for Cath Lab

SN	Emergency Drugs (including neonates) for OT	Standard Quantity for 1 patient	Score
1.	Verapamil (inj)	4 ampules	
2.	Heparin (inj)	2 vials	
3.	Midazolam (Inj)	5 vials	
4.	Hydrocortisone	100ml 2 vial	
5.	Frusemide (inj)	2 ampules	
6.	Dopamine	5 vials	
7.	Ergometrine (inj)	2 ampules	
8.	Hydralizine injection	5 vials	
9.	Calcium Gluconate (inj)	10ml X 2 ampules	
10.	Dextrose (25%) (inj)	2 ampules	
11.	Naloxone (inj)	1 ampule	
12.	Aminophyline (inj)	2 ampules	
13.	Chloropheniramine	2 ampules	
14.	Mephentine (inj)	1 vial	
15.	IV Fluids- Ringer Lactate / Normal Saline/ DNS/ D5%	6 bottles each	
16.	IV infusion Set	4	
17.	IV Canula 22G/20G/18G	4 each	
		**Total Score**	
		**Total Percentage = Total Score/17 X 100**	

Each row gets a score of 1 if all the required number is available otherwise 0

**Scoring Chart**	
**Total percentage**	**Score**
0-50	0
50-70	1
70-85	2
85-100	3
Score 2.16.7.3	

Annex 2.16d Minimum List of Surgical Sets for Intervention

S.No.	Items	Required number	Score
1.	Catheterization set	At least 5	
2.	Coronary angiography set	At least 2	
3.	PTCA kit	At least 2	
4.	TPI Set	At least 2	
5.	Swan Ganz Catheter	At least 2	
6.	Teurmo Wire J tip	At least 5	
7.	Radial/ femoral sheath 5 Fr	At least 10 each	
8.	Port manifold	At least 10	
9.	Ordinary wire	At least 5	
10.	Mersilk (2-0) cutting body	As per need	
11.	Pigtail 5/6 Fr	At least 5	
		**Total Score**	
		**Total Percentage = Total Score/11 x 100**	

Each row gets a score of 1 if all the required number is available otherwise 0.

**Scoring Chart**	
**Total Percentage**	**Score**
0-50	0
50-70	1
70-85	2
85-100	3
**Score for Standard 2.16.7.4**	

Section III: Hospital Support Services Standards

Summary Sheet for Standards and Scores

Area	Total Number of Standards	Total Score	Total Obtained Score (Percentage)
Central Supply Sterile Department (CSSD)	17	19	
Laundry	17	19	
Housekeeping	13	15	
Repair, Maintenance and Power System	12	12	
Water Supply	4	4	
Hospital Waste Management	17	17	
Safety and Security	15	17	
Transportation and Communication	8	8	
Store (Medical and Logistics)	7	7	
Hospital Canteen	16	16	
Social Service Unit (SSU)	12	14	
Total	138	148	
Score of Section III<br><br>(Average of the percentage obtained = Sum of percentage obtained in each sub-section/ Number of sub-section (11))			

Area	Code	Verification		
**CSSD**	**3.1**			
**Components**		**Standards**	**Obtained Score**	**Maximum Score**
3.1.1 Space	3.1.1.1	Separate central supply sterile department (CSSD) is available with running water facility		1
	3.1.1.2	There are separate rooms designated for dirty utility, cleaning, washing and drying and sterile area for sterilizing, packaging and storage		1
3.1.2 Staffing	3.1.2	Separate staffs assigned for CSSD and is led by CSSD trained personal		1
3.1.3 Equipment and supplies for CSSD	3.1.3	Equipment and supplies for sterilization available and functional round the clock (See Annex 3.1a CSSD Equipment and Supplies At the end of this standard)		3
3.1.4 Preparing consumables	3.1.4	Wrapper, gauze, cotton balls, bandages are prepared.		1
3.1.5 Preparing for sterilization	3.1.5.1	All used instruments are cleaned using brush chemical/detergents in a separate room.		1
	3.1.5.2	All instruments and equipment are dried in a separate place		1
	3.1.5.3	All instruments are packed in double wrappers		1
3.1.6 Sterilization	3.1.6	All wrapped instruments are indicated with thermal indicator and autoclaved in a separate room.		1
3.1.7 Storage	3.1.7	All sterile packs with sticker of sterilization date are stored in separate cupboards		1
3.1.8 Collection and Distribution	3.1.8.1	System for single door collection and different route for distribution of the sterile supply exist and is practiced		1
	3.1.8.2	Sterile supplies are distributed using basket supply system or on-demand supply system		1
3.1.9 Inventory	3.1.9	All instruments and wrappers are recorded and inventory maintained		1
3.1.10 Infection prevention	3.1.10.1	Staffs use personal protective equipment at work		1
	3.1.10.2	There are well labelled colored bins for waste segregation and disposal based on HCWM[7] guideline 2014 (MoHP)		1
	3.1.10.3	Hand-washing facility with running water and liquid soap is available and being practiced.		1
	3.1.10.4	Chlorine solution is available and utilized for decontamination		1
**Standard 3.1**		**Total Obtained Score**		**19**
		**Percentage = Total Obtained Score / 19 x 100**		

7 HCWM: Health Care Waste Management

Annex 3.1a CSSD Equipment and Supplies

SN	Items	Required No.	Score
1.	Working Table	3	
2.	Trolley for Transportation	2	
3.	Steel Drums	10	
4.	Storage Shelves	2	
5.	Autoclave Machine (250 liter, pre-vacuum, with horizontal outlet)	2	
6.	Double Wrappers	As per need	
7.	Timer	2	
8.	Thermal Indicator Tape	As per need	
9.	Cap, Mask, Gown, Apron	As per need	
10.	Gloves	1 box	
11.	Cotton Rolls	As per need	
12.	Cotton Gauze	As per need	
13.	Scissors	2	
14.	Gauze cutter	2	
15.	Buckets	5	
16.	Scrub Brush	As per need	
17.	Hamper bag (cloth sack for collection of wrappers)	As per need	
		**Total Score**	
		**Total Percentage = Total Score/17 X 100**	

Each row gets a score of 1 if all the required number is available otherwise 0.

**Scoring Chart**	
**Total Percentage**	**Score**
0-50	0
50-70	1
70-85	2
85-100	3
**Score for Standard 3.1.3**	

Area	Code	Verification		
**Laundry**	**3.2**			
**Components**		**Standards**	**Obtained Score**	**Maximum Score**
3.2.1 Space	3.2.1.1	Separate laundry room is available.		1
	3.2.1.2	Separate space allocated for clean and dirty linens		1
3.2.2 Staffing	3.2.2	There is a special schedule for collection and distribution of linens with visible duty roster for staffs		1
3.2.3 Equipment/ Supplies	3.2.3	Adequate equipment and supplies are available for laundry (See Annex 3.2a Equipment and Supplies for Laundry At the end of this standard)).		3
3.2.4 Segregation and decontamination of linens	3.2.4.1	Linens are segregated (soiled, unsoiled, colorful, white, blood stained) before wash		1
	3.2.4.2	Separated linens are decontaminated before wash		1
3.2.5 Cleaning	3.2.5	All linens are washed using a washing machine.		1
3.2.6 Drying	3.2.6.1	Space available for drying linens like blankets in direct sunlight.		1
	3.2.6.2	Linen dryer is available and used		1
3.2.7 Packing	3.2.7	All linens are ironed and packed properly.		1
3.2.8 Storage	3.2.8	Linens are properly stored in separate cupboard.		1
3.2.9 Distribution	3.2.9	All linens are distributed using a proper method (basket supply system and on-demand supply system).		1
3.2.10 Inventory	3.2.10	All linens are recorded and inventory maintained.		1
3.2.11 Infection prevention	3.2.11.1	Staff wear mask and gloves at work.		1
	3.2.11.2	There are well labelled colored bins for waste segregation and disposal based on HCWM[8] guideline 2014 (MoHP)		1
	3.2.11.3	Hand-washing facility with running water and soap is available and being practiced.		1
	3.2.11.4	Chlorine solution is available and utilized for decontamination		1
**Standard 3.2**		**Total Obtained Score**		**19**
		**Percentage = Total Obtained Score/ 19 x 100**		

8 HCWM: Health Care Waste Management

Annex 3.2a Equipment and Supplies for Laundry

SN	List of equipment and supplies	Required No.	Score
1.	Working table	1	
2.	Ironing Table	1	
3.	Storage Shelves	2	
4.	Trolley for Transportation	2	
5.	Washing Machine (at least 10 kg capacity with semi/full dryer)	2	
6.	Iron Machine	1	
7.	Buckets/ Basins	5	
8.	Stirrer (wooden)	2	
9.	Boots	2 pairs	
10.	Cap, Mask, Gowns	As per need	
11.	Ropes (for drying)	As per need	
12.	Scrub Brush	As per need	
13.	House/ Utility Gloves	As per need	
14.	Washing Powder	As per need	
15.	Chlorine Liquid/ Powder	As per need	
		**Total Obtained Score**	
		**Total Percentage = Total Obtained Score/15 X 100**	
Each row gets a score of 1 if all the required number is available otherwise 0.			

**Scoring Chart**	
**Total Percentage**	**Score**
0-50	0
50-70	1
70-85	2
85-100	3
**Score for Standard 3.2.3**	

Area	Code	Verification		
**Housekeeping**	**3.3**			
**Components**		**Standards**	**Obtained Score**	**Maximum Score**
3.3.1 Space for storage	3.3.1	Separate space is allocated for storage of the housekeeping basic supplies		1
3.3.2 Staffing	3.3.2.1	Allocation of the staff for cleaning with visible duty roster		1
	3.3.2.2	There is checklist of cleaning in each department with contact number of assigned working personnel		1
3.3.3 Basic Supplies	3.3.3	Basic supplies are available (See Annex 3.3a Housekeeping Basic Supplies At the end of this standard)).		3
3.3.4 Cleaning	3.3.4.1.1	The hospital premises are visibly clean and dust free		1
	3.3.4.1.2.1	All hospital toilets are clean with no offensive smell		1
	3.3.4.1.2.2	All toilets are cleaned at least three times a day		1
	3.3.4.3	All doors and windows of hospital are dust-free and cleaned once a day.		1
	3.3.4.4	All floors of the hospital are clean and cleaned at least twice a day (like- before registration in morning and after OPD closes)		1
	3.3.4.5	All walls of the hospital are clean and are tiled or painted with enamel up to 4 feet		1
	3.3.4.6	Every ward/unit must have high wash twice a month and fumigation as per need		1
3.3.5 Drainage of chlorine solution	3.3.5	Separate drainage system or pit is maintained for drainage of chlorine solution		1
3.3.6 Garden	3.3.6	Garden and trees should cover at least 25% of the hospital premises		1
**Standard 3.3**		**Total Obtained Score**		**15**
		**Percentage = Total Obtained Score / 15 x 100**		

Annex 3.3a Housekeeping Basic Supplies

SN	General Items	Required No.	Score
1.	Working Table and Chair	1	
2.	Telephone	1	
3.	Housekeeping Storage Room	1	
4.	Shelves	2	
5.	Cupboards	2	

6.	Log Book for Records	1	
7.	Vacuum Cleaner	1	
8.	Sickle	As per need	
9.	Spade	As per need	
10.	Shovel	As per need	
11.	Ropes	As per need	
12.	Scrub Brush	As per need	
13.	Broom	As per need	
14.	Buckets	As per need	
15.	Jars	As per need	
16.	Sprinkle Pipe	As per need	
17.	Soaps	As per need	
18.	Washing Powder	As per need	
19.	Additional Bed Covers for Replacement	As per need	
20.	Additional Pillow	As per need	
21.	Pillow cover	As per need	
22.	Blankets	As per need	
23.	Personal Protective Items	As per need	
24.	Window screens (jaali)	In all windows	
25.	Mosquito nets	As per need	
26.	Flower Pots	As per need	
		**Total Score**	
		**Total Percentage = Total Score/26 X 100**	

Each row gets a score of 1 if all the required number is available otherwise 0.

**Scoring Chart**	
**Total Percentage**	**Score**
0-50	0
50-70	1
70-85	2
85-100	3
**Score 3.3.3**	

Area	Code	Verification		
**Repair, Maintenance and Power system**	**3.4**			
**Components**		**Standards**	**Obtained Score**	**Maximum Score**
3.4.1 Staffing	3.4.1.1	Human resource trained in biomedical engineer is designated for repair and maintenance		1
	3.4.1.2	Staffs assigned to cover 24 hours shift with visible duty roster for staffs.		1
3.4.2 Preventive Maintenance	3.4.2.1	Hospital has regular preventive maintenance practices (calibration, servicing of equipment) and corrective maintenance)		1
	3.4.2.2	Biomedical equipment inventory of all equipment and instrument is updated		1
	3.4.2.3	Separate room for storage of repairing tools and instrument		1
	3.4.2.4	Availability of spare parts for repair and maintenance of biomedical equipment and instruments		1
	3.4.2.5	Record keeping of repair and maintenance of biomedical equipment and instruments		1
	3.4.2.6	Specification of annual maintenance cost of major equipment		1
3.4.3 Availability of power sources	3.4.3.1	Hospital has main-grid power supply with three-phase line		1
	3.4.3.2	Hospital has alternate power generator capable of running x-ray and other hospital equipment		1
	3.4.3.3	Proper inventory of fuel is maintained.		1
	3.4.3.4	Hospital has solar system installed (at least for essential clinical services and administrative function).		1
**Standard 3.4**		**Total Obtained Score**		12
		**Percentage = Total Obtained Score / 12 x 100**		

Area	Code	Verification		
**Water supply**	**3.5**			
**Component**		**Standards**	**Obtained Score**	**Maximum Score**
3.5.1 Water supply	3.5.1	There is regular water supply system – boring or well or from drinking water supply dedicated for hospital		1
3.5.2 Water Storage	3.5.2.1	Water storage tank is covered to prevent contamination and cleaned on a regular basis		1
	3.5.2.2	Water storage tank has the reserve capacity to supply water for two full days in case of interruptions in main water supply		1
3.5.3 Water quality	3.5.3	Water quality test is done every year and report is available as per Nepal Drinking Water Quality Standards, 2005		1
**Standard 3.5**		**Total Obtained Score**		**4**
		**Percentage = Total Obtained Score / 4 x 100**		

Area	Code	Verification		
**Hospital Waste Management**	**3.6**			
**Components**		**Standards**	**Obtained Score**	**Maximum Score**
3.6.1 Staffing	3.6.1.1	There is allocation of staff for HCWM from segregation to final disposal		1
	3.6.1.2	Whole site coaching/ orientation on health care waste management is done		1
3.6.2 Space	3.6.2	There is separate area/space designated for waste storage and management with functional hand washing facility		1
3.6.3 Segregation of waste from source to final disposal	3.6.3	Different colored bins (for risk and non-risk waste) based on HCWM guideline 2014 (MoHP) are used from source to final disposal		1
3.6.4 Personal protection	3.6.4	Staff use cap, mask, gloves, boot, and gown while collecting waste.		1
3.6.5 Public information	3.6.5.	Information regarding proper use of waste bins is displayed publicly and basic information of HCWM is displayed in hospital premises		1
3.6.6 Medication trolley with waste segregation buckets	3.6.6	Medication trolley has well labeled buckets for segregation of waste during procedures		1

3.6.7 Transportation of waste within the hospital	3.6.7	Hospital uses transportation trolleys separate for risk and non-risk waste		1
3.6.8 Disposal and recycle/reuse of waste	3.6.8.1	Infectious waste is sterilized using autoclave before disposal		1
	3.6.8.2	Collection of recyclable/reusable items such as plastic bottles, paper, decontaminated sharps is practiced and sold to vendor/municipality		1
	3.6.8.3	Composting of bio-degradable waste is practiced		1
	3.6.8.4	Collection of waste by the local municipality/ rural municipality after sterilization /decontamination		1
	3.6.8.5	Placenta pit used for disposal of human anatomical waste such as placenta, human tissue		1
	3.6.8.6	Biogas plant in place and energy generated used for hospital support services		1
3.6.9 Pharmaceutical and radiological waste management	3.6.9	Pharmaceutical waste and radiological waste treated and disposed based on the HCWM guideline 2014 (MoHP)		1
3.6.10 Liquid waste management	3.6.10	Hospital liquid waste management is done		1
3.6.11 Waste management of electronic goods and products	3.6.11	Hospital has IT personnel to coordinate to manage waste related to electronic goods and products		1
**Standard 3.6**		**Total Obtained Score**		**17**
		**Percentage = Total Obtained Score / 17 x 100**		

**Area**	**Code**			
**Safety and Security**	**3.7**	**Verification**		
**Component**		**Standards**	**Obtained Score**	**Maximum Score**
3.7.1 Staffing of security personnel	3.7.1.1	Hospital has trained security personnel round the clock.		1
	3.7.1.2	All security staffs are oriented with hospital codes like 001- call for help for crashing patients, 007- call for disaster in ER		1
	3.7.1.3	All security staffs have participated in emergency drills		1
3.7.2 Office space allocated for security personnel	3.7.2	A separate office for security with communication system is available		1

3.7.3 Amenities	3.7.3	Basic amenities for safety and security are available (See Annex 3.7a Safety and Security Basic Amenities At the end of this standard)		3
3.7.4 Patient safety	3.7.4	The hospital has replaced all mercury apparatus with other appropriate technologies.		1
3.7.5 Continuous surveillance of hospital premises	3.7.5	CCTV coverage of major areas and control under Medical Superintendent and security in-charge		1
3.7.6 Hospital has disaster mitigation system	3.7.6.1	The hospital has fire extinguisher in all blocks including the fire extinguishing system		1
	3.7.6.2	The hospital has installed safety alarm system including smoke detector		1
	3.7.6.3	The hospital has prevented lightening by ensuring earthing system in electrification.		1
	3.7.6.4	Disaster preparedness orientation has been given to all staff at least every six months.		1
	3.7.6.5	Exit signs are displayed to escape during disaster in all departments and wards		1
	3.7.6.6	An assembly zone has been specified for disaster		1
	3.7.6.7	Hospital has functional rapid response team		1
	3.7.6.8	Medicine stock for post disaster response is available		1
**Standard 3.7**		**Total Obtained Score**		**17**
		**Percentage = Total Obtained Score / 17 x 100**		

Annex 3.7a Safety and Security Basic Amenities

SN	General Items	Score
1	Flash light	1
2	Whistle	1
3	List of Important Phone Numbers	1
4	Key Box	1
5	Emergency Alarm	1
6	Fire extinguisher at least one in each block	1
	**Obtained Score**	
	**Total Percentage = Total Score/6 X 100**	

Each row gets a score of 1 if all the mentioned items are available otherwise 0.

**Scoring Chart**	
**Total Percentage**	**Score**
0-50	0
50-70	1
70-85	2
85-100	3
**Score for Standard 3.7.4**	

Area	Code	Verification		
**Transportation and Communication**	**3.8**			
**Components**		**Standards**	**Obtained Score**	**Maximum Score**
3.8.1 Transportation	3.8.1.1	24-hour ambulance service is available.		1
	3.8.1.2	Hospital has its own well-equipped ambulance at least 2		1
	3.8.1.3	The hospital has access to utility van		1
3.8.2 Communication	3.8.2.1	The hospital has telephone with intercom (EPABX) network.		1
	3.8.2.2	Internal communication (paging) system has been installed in all major service stations.		1
	3.8.2.3	A notice board is available and being utilized.		1
	3.8.2.4	List of important phone numbers including emergency contacts like ambulance, fire brigade, blood banks, hospital administration, hospital staffs is available in the reception, emergency and administration office		1
	3.8.2.5	There should be a public contact or information center in prime location of hospital with 24 hours staff availability		1
**Standard 3.8**		**Total Obtained Score**		**8**
		**Percentage = Total Obtained Score / 8 x 100**		

Area	Code	Verification		
**Store (Medical and Logistics)**	**3.9**			
**Components**		**Standards**	**Obtained Score**	**Maximum Score**
3.9.1 Space	3.9.1	Separate space allocated for store for hospital- medicine and logistics		1
3.9.2 Buffer stock in medical store	3.9.2.1	A separate hospital medical store with 3 months' buffer stock is available		1
	3.9.2.3	Minimum and Maximum stock levels for each item are calculated and used when re-ordering stock		1
3.9.3 Inventory	3.9.3.1	Electronic database system is used in the hospital medical store.		1
	3.9.3.2	Hospital submits quarterly reports to LMIS utilizing either paper report or web-based (eLMIS-7)		1

3.9.4 Disposal of expired medicine	3.9.4	Disposal of expired medicine as per HCWM guideline 2014 (MoHP) is practiced in every six month		1
3.9.5 Auction of logistics	3.9.5	Auction of identified old logistics is done annually		1
**Standard 3.9**		**Total Obtained Score**		**7**
		**Percentage = Total Obtained Score / 7 x 100**		

Area	Code	Verification		
**Hospital Canteen and Dietetics**	**3.10**			
**Components**		**Standards**	**Obtained Score**	**Maximum Score**
3.10.1 Time for patients/ visitors and staff	3.10.1	Hospital has canteen in its premises with 24 hours service		1
3.10.2 Information to patients/ visitors and staffs	3.10.2	A list of food items with price list approved by Hospital Management Committee is available		1
3.10.3 Physical facilities	3.10.3.1	Visibly clean floors and space allocated for cooking, cleaning and storage of stock		1
	3.10.3.2	Light and ventilation are adequately maintained.		1
	3.10.3.3	All walls of the canteen are clean and are tiled or painted with enamel up to 4 feet		1
	3.10.3.4	Safe drinking water is available 24 hours		1
3.10.4 Uniform for canteen staffs	3.10.4	Dress code is maintained		1
3.10.5 Food for inpatients under supervision of dietetics	3.10.5.1	Staffs assigned for inpatient diet including the intensive care units and patient needing hemodialysis:<br>Trained dietetic staff assigned for inpatient nutrition care : one Senior Dietitian (Masters qualification in Nutrition & Dietetics including hospital internship or Bachelors in Nutrition & Dietetics with 1 years hospital experience) plus one dietetic assistant per hundred general beds, plus one office assistant<br><br>Additionally, 1 senior dietitian per 25 beds for all specialized services, including ICU, NICU, PICU, nephrology/hemodialysis,		1
	3.10.5.2	The inpatients who are identified needy or covered by SSU are provided with food at least four times a day and the food contains carbohydrate, fats, proteins and at least one vegetable item		1
3.10.6 IEC/ BCC materials	3.10.6	Appropriate IEC/ BCC materials (posters, leaflets, television) are available in the canteen for balanced diet		1

3.10.7 Facilities for staffs, patients and visitors	3.10.7	Comfortable space with sitting arrangement is available for at least 50 people		1
3.10.8 Infection prevention and food hygiene	3.10.8.1	Separate area designated for washing dishes and visibly clean.		1
	3.10.8.2	There are colored bins for waste segregation and disposal based on HCWM guideline 2014 (MoHP)		1
	3.10.8.3	Hand-washing facility with running water and soap is available		1
	3.10.8.4	Mesh/ net to cover food and refrigerator used to store food used to cover food		1
	3.10.8.5	Rat proofing and daily scrubbing of the canteen is done		1
**Standard 3.10**		**Total Obtained Score**		**16**
		**Percentage = Total Obtained Score / 16x 100**		

**Area**	**Code**	**Verification**		
**Social Service Unit**	**3.11**			
**Components**		**Standards**	**Obtained Score**	**Maximum Score**
3.11.1 Time for patients	3.11.1.1	SSU open from 8am to 7pm		1
	3.11.1.2	Management committee to manage 24 hours SSU services for patients		1
3.11.2 Physical facilities for SSU services	3.11.2	Separate space allocated for SSU is accessible to patients (See Annex 3.11a Physical Facilities SSU At the end of this standard)		3
3.11.3 Staffing	3.11.3	Allocation of staffs for SSU under unit chief a team of 2 to 10 facilitators		1
3.11.4 Identify and display target group and services covered	3.11.4	Refer to ‘deprived citizen treatment fund guideline 2071’ to identify target group, and display target group and services covered		1
3.11.5 Referral mechanism in place	3.11.5.1	Referral of patients based on treatment protocol		1
	3.11.5.2	Documentation of referral based on ‘deprived citizen treatment fund guideline 2071’		1
	3.11.5.3	SSU allocates fund for transportation for referral		1

3.11.6 Recording and reporting	3.11.6.1	Meetings of SSU every two months to review and discuss problems		1
	3.11.6.2	Daily display of names of persons receiving free and partially free services from the unit		1
	3.11.6.3	Record information on free and partially free service recipients on the formats to records section of the concerned hospital		1
	3.11.6.4	Prepare and submit monthly, trimester and annual report to concerned authority		1
**Standard 3.11**		**Total Obtained Score**		**14**
		**Percentage = Total Obtained Score / 14 x 100**		

Annex 3.11a Physical Facilities SSU

SN	General Items	Required No.	Score
1.	Separate space for SSU	Available	
2.	Desk	2	
3.	Chairs	3	
4.	Cupboard to keep clients' information with filing cabinet	1	
5.	Recording and reporting forms	as per need	
6.	Telephone	1	
7.	Computer and printer	1	
		**Total Score**	
		**Percentage= Total Score / 7x 100**	

Each row gets a score of 1 if all the mentioned items are available otherwise 0.

**Scoring Chart**	
**Total Percentage**	**Score**
0-50	0
50-70	1
70-85	2
85-100	3
**Score for Standard 3.11.2**	

Annex I: List of Subject Experts

- Roshani Laxmi Tuitui, Chief, Hospital Nursing Administrator, MoHP
- Dr Roshan Neupane, Chief Medical Superintendent, Myagdi District Hospital
- Dr Chuman Lal Das, Chief Medical Superintendent, Sagarmatha Zonal Hospital
- Ramkrishna Lamichhane, Under-Secretary, CSD/ MoHP
- Sangita Shah, Senior Drug Administrator, QARD, MoHP
- Krishna Subedi, Section Officer, CSD/MoHP
- Shrijana Shrestha, Chief, Environmental Health and HCWM Section, MD, DoHS
- Dr Runa Jha, Pathologist, National Public Health Laboratory, MoHP
- Uma Kumari Rijal, Nursing Officer, MoHP
- Bijaya Kranti Shakya, Sr. PHO, QARD, MoHP
- Kopila Shrestha Palikhey, Nursing Director, TUTH
- Dr Asha Thapa, Dental Surgeon, NAMS
- Dr Bhaskar M. M. Kayastha, Dermatologist, NAMS
- Dr Dhundi Paudel, ENT/ Audiologist, NAMS
- Dr Ravi Ram Shrestha, Chief Consultant Anesthesiologist, NAMS
- Dr Rajiv Jha, Neurosurgeon, NAMS
- Dr Saroj Sharma, Consultant Radiologist, NAMS
- Dr Peeyush Dahal, Consultant Burn/Plastic Surgeon, NAMS
- Dr Ashish Shrestha, Consultant Physician, PAHS
- Dr Rajesh Gangol, Palliative care specialist, PAHS
- Dr Ramesh Kandel, Consultant Geriatrician, PAHS
- Dr Nabees Man Singh Pradhan, Consultant Orthopedics,PAHS
- Dr Sanjay Paudel, Consultant Surgeon, PAHS
- Dr Anil Baral, Consultant Nephrologist, NAMS
- Dr Shanta Bir Maharjan, Consultant Surgeon, PAHS
- Mohan Poudel, Housekeeping and Laundary Incharge, PAHS
- Devi Shah, CSSD Incharge, PAHS
- Dr Ananta Adhikari, Psychiatrist, Patan Mental Hospital
- Dr Jageshowor Gautam, Director, Paropakar Maternity and Women's Hospital
- Asha Laxmi Prajapati, Nurse, Paropakar Maternity and Women's Hospital
- Dr Archana Amatya, Obstetrician and Gynecologist, TUTH/IOM
- Dr Harihar Wasti, Medico-legal Expert, TUTH/IOM
- Dr Ramesh Kumar Maharjan, Emergency Physician, TUTH/IOM
- Dr Ratna Mani Gajurel, Cardiologist, Manmohan Cardiovacular and Throcic Centre, TUTH/ IOM
- Dr Rakesh Verma, Urologist, Human Organ Transplant Center
- Subhadra Regmi, Hemodialysis Nurse, Human Organ Transplant Center
- Dr Amir Neupane, Physiotherapist, AASHAH Health Care
- Dr Ben Limbu, Ophthalmologist, Til Ganga Eye Hospital
- Amit Kumar Shah, Radiographer, District Hospital Dhankuta
- Ambika Thapa, Technical Coordinator for MSS, MoHP, WHO Nepal/NSI/NHSSP

Annex II: List of Reviewers

- Dr Krishna Kumar Rai, Technical Advisor to Minister of Health and Population
- Dr Sushil Nath Pyakurel, Chief Specialist, MoHP
- Dr Guna Raj Lohani, DG, DoHS
- Narayan Prasad Dahal, DG, DDA
- Dr Vasudev Upadhyaya, DG, DoA
- Santa Bahadur Shrestha, Former Secretary of MoHP
- Dr Taranath Poudel, Medical Generalist, MoHP
- Dr Dipendra Raman Singh, Chief, QARD, MoHP (Chief, The Then CSD, MoHP)
- Dr Bhim Singh Tinkari, Chief, The then PHAMED, MoHP
- Mohammad Daud, Chief, Federalism Implementation Unit, MoHP
- Dr Bikash Devkota, Chief, Management Division, DoHS, MoHP
- Dr Madan Kumar Upadhyaya, Executive Director, Health Insurance Board
- Mahendra Shrestha, Advocacy Officer to Minster of Health and Population
- Roshani Laxmi Tuitui, Chief, Hospital Nursing Administrator, MoHP
- Dr Roshan Neupane, Chief Medical Superintendent, Myagdi District Hospital
- Dr Chuman Lal Das, Chief Medical Superintendent, Sagarmatha Zonal Hospital
- Ramkrishna Lamichhane, Under-Secretary, The Then CSD/ MoHP
- Sangita Shah, Senior Drug Administrator,QARD, MoHP
- Krishna Subedi, Section Officer, The Then CSD/MoHP
- Shrijana Shrestha, Chief, Environmental Health and HCWM Section, MD, DoHS
- Dr Arjun Sapkota, Chief, The then Quality Section, MD, DoHS
- Dr Basudev Pandey, Director NCASC, DoHS
- Dr Runa Jha, Pathologist, National Public Health Laboratory, MoHP
- Dr Punya Paudel, The then FHD, DoHS
- Uma Kumari Rijal, Nursing Officer, MoHP
- Bijaya Kranti Shakya, Sr. PHO, QARD, MoHP
- Dipak Raj Bhatta, PHI, DoHS
- Krishna Shrestha, NHTC, DoHS
- Kopila Shrestha Palikhey, Nursing Director, TUTH
- Dr Asha Thapa, Dental Surgeon, NAMS
- Dr Bhaskar M. M. Kayastha, Dermatologist, NAMS
- Dr Dhundi Paudel, ENT/ Audiologist, NAMS
- Dr Ravi Ram Shrestha, Chief Consultant Anesthesiologist, NAMS
- Dr Rajiv Jha, Neurosurgeon, NAMS
- Dr Saroj Sharma, Consultant Radiologist, NAMS
- Dr Bhupendra Basnet, Director, Bir Hospital
- Dr Ganesh Rai, Director, Kanti Children's Hospital
- Dr Dilip Sharma, Registrar, Nepal Medical Council
- Dr Ganesh Shah, Consultant Pediatrician, PAHS
- Dr Ashish Shrestha, Consultant Physician, PAHS
- Dr Bishnu Prashad Sharma, Director, PAHS
- Dr Arjun Acharya, Director, WRH, Pokhara
- Dr Rajesh Gangol, Palliative care specialist, PAHS
- Dr Ramesh Kandel, Consultant Geriatrician, PAHS
- Dr Nabees Man Singh Pradhan, Consultant Orthopedics, PAHS
- Dr Sanjay Paudel, Consultant Surgeon, PAHS
- Dr Shanta Bir Maharjan, Consultant Surgeon, PAHS
- Mohan Poudel, Housekeeping and Laundary Incharge, PAHS
- Devi Shah, CSSD Incharge, PAHS
- Dr Ananta Adhikari, Psychiatrist, Patan Mental Hospital
- Dr Jageshowor Gautam, Director, Paropakar Maternity and Women's Hospital
- Asha Laxmi Prajapati, Nurse, Paropakar Maternity and Women's Hospital

- Dr Archana Amatya, Obstetrician and Gynecologist, TUTH/IOM
- Dr Harihar Wasti, Medico-legal Expert, TUTH/IOM
- Dr Ramesh Kumar Maharjan, Emergency Physician, TUTH/IOM
- Dr Ratna Mani Gajurel, Cardiologist, Manmohan Cardiovacular and Throcic Centre, TUTH/ IOM
- Dr Rakesh Verma, Urologist, Human Organ Transplant Center
- Subhadra Regmi, Hemodialysis Nurse, Human Organ Transplant Center
- Dr Amir Neupane, Physiotherapist, AASHAH Health Care
- Dr Ben Limbu, Ophthalmologist, Til Ganga Eye Hospital
- Dr Mukti Shrestha, Chairperson, Nepal Medical Association
- Dr Prakash Budhathoky, Sr. Dental Surgeon, NAMS; Treasurer, Nepal Medical Association
- Raj Kumari Gyawali, Nursing Association of Nepal
- Amit Kumar Shah, Radiographer, District Hospital Dhankuta
- Dr Amrit Pokhrel, Medical Superintendent, Syanjga District Hospital
- Dr Shilu Aryal, Obgyn Expert
- Kabiraj Khanal, NHSSP
- Dr Indira Basnet, NHSSP
- Dr Sushil Chandra Baral, NHSSP
- Dr Kishori Mahat, NHSSP
- Ghanshyam Gautam, NHSSP
- Pradeep Paudel, NHSSP
- Maureen, NHSSP
- Kamala Shrestha, NHSSP
- Dr Anil Bahadur Shrestha, Executive Director, NSI
- Dr Kashim Shah, NSI
- Rita Pokhrel, NSI
- Ian Chandwell, NSI
- Balsundar Chashi, NSI
- Bijay Dhakal, NSI
- Janardhan Pathak, NSI
- Jot Narayan Patel, NSI
- Sushma Lama , NSI
- Sharada Shah , NSI
- Prashanta Vikram Shahi, NSI
- Dr Meera Upadhayaya, WHO Nepal
- Kimat Adhikari, WHO Nepal
- Anjana Rai, WHO Nepal
- Ambika Thapa, Technical Coordinator for MSS, MoHP, WHO Nepal/NSI/NHSSP

Print supported by UKaid
